# Discovery of a Reversible Sub‐Picomolar Thrombin Inhibitor Using DCC

**DOI:** 10.1002/anie.5778436

**Published:** 2026-07-15

**Authors:** Millicent Dockerill, Richard J. Payne, Nicolas Winssinger

**Affiliations:** ^1^ Department of Organic Chemistry Faculty of Sciences University of Geneva Geneva Switzerland; ^2^ School of Chemistry The University of Sydney Sydney New South Wales Australia; ^3^ Australian Research Council Centre of Excellence for Innovations in Peptide and Protein Science The University of Sydney Sydney New South Wales Australia

**Keywords:** active site, chemistry, combinatorial chemistry, ligand, mass spectrometry, mass spectrometry imaging, nucleic acid, peptide nucleic acid, thrombin

## Abstract

Dynamic combinatorial chemistry (DCC) offers a powerful yet underutilized strategy for ligand discovery, largely limited by heterogeneous kinetic constraints in exchange reactions and analytical challenges. Here we report a peptide nucleic acid (PNA)‐templated trivalent DCC platform that enables rapid, target‐guided exploration of 125 000 assemblies to identify ultrahigh‐affinity and reversible thrombin inhibitors. Short hybridization handles allow unbiased equilibration of a three‐fragment library, and size‐exclusion filtration combined with matrix‐assisted laser desorption/ionization mass spectrometry (MALDI‐MS) provides a complete selection–readout cycle in under 1 h. From this library, thrombin amplifies synergistic fragment combinations engaging the active site and both exosites, yielding a trivalent inhibitor with apparent sub‐picomolar affinity (*K*_D_ ≈ 84 fM) and near‐stoichiometric inhibition. Despite its extreme potency, inhibition remains fully reversible: addition of a single‐stranded toehold antidote rapidly disassembles the complex and restores activity in buffer and in plasma. These results establish hybridization‐guided DCC as a fast, scalable route to programmable multivalent therapeutics with on‐demand reversibility.

## Graphical abstract

An ultrapotent yet fully reversible trivalent thrombin inhibitor discovered through screening a large dynamic combinatorial library (DCL) of 125 000 members of self‐assembled fragments. The innovative approach leverages size‐exclusion‐based affinity selection, coupled with MALDI‐TOF mass spectrometry readout, enabling the full workflow to be completed in under 1 h.

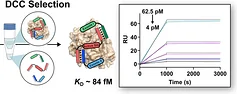

## 1 Introduction

**FIGURE 1:**
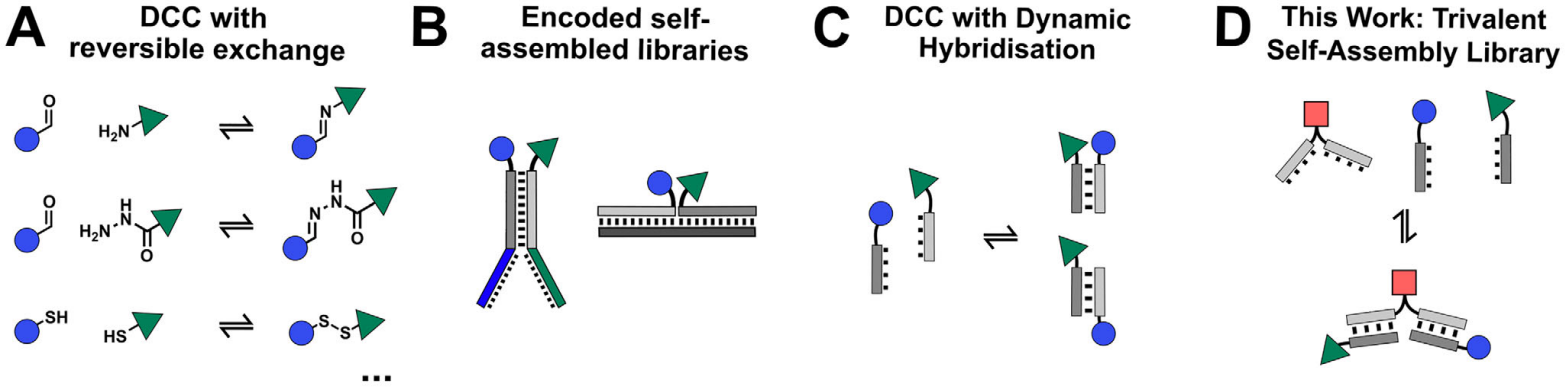
Dynamic Combinatorial Chemistry (DCC). (A) DCC with reversible covalent bond exchange. (B) DEL encoded self‐assembled chemical libraries (ESAC). (C) DCC with dynamic hybridization. (D) This work: trivalent library targeting thrombin.

High‐throughput screening (HTS) of large compound libraries is historically the most effective strategy for ligand discovery but is often constrained by the time‐ and resource‐intensive nature of library preparation and the screen itself. Fragment‐based approaches provide an attractive alternative, yet identifying suitable chemistries to link fragments with the correct spatial geometry remains a major bottleneck [1, 2, 3, 4]. Dynamic combinatorial chemistry (DCC) effectively bridges these two paradigms: fragments exchange dynamically through reversible covalent bonds, allowing the target itself to select the optimal fragment combination from a virtual library in dynamic equilibrium [5, 6]. Since its introduction by Lehn and Sanders in the late 1990s, DCC has been applied to a wide range of molecular recognition challenges, including enzyme inhibition, catalyst discovery, and the modulation of protein–protein interactions (Figure 1) [7, 8, 9, 10, 11, 12, 13]. Despite its conceptual elegance, DCC remains underutilized in drug discovery, primarily due to technical limitations. These include the scarcity of reversible chemistries compatible with biological conditions, the intrinsic biases in equilibrium composition arising from fragment reactivity or agonistic/antagonistic effects in the selection, and the analytical challenges associated with deconvoluting complex library equilibria [14, 15, 16, 17, 18, 19]. DNA‐encoding strategies have greatly simplified the analysis of selection outcomes [20, 21, 22, 23, 24, 25, 26, 27], and the use of hybridization‐based dynamic exchange—via constant DNA or peptide nucleic acid (PNA) scaffolds—provides a homogeneous and well‐defined framework for library equilibration [28, 29].

We recently reported a potent thrombin inhibitor exhibiting on‐demand reversibility, assembled from two fragments held together through short PNA hybridization [30]. Cooperative stabilization between target engagement and duplex formation drives the formation of the assembly at concentrations far below the *K*_D_ of the individual fragments, but inhibition remains reversible upon addition of a competing PNA. Consistent with the synergy between hybridization and target binding, competition‐driven hybridization displacement by an oligonucleotide was relatively slow, despite the short hybridization sequence employed. By introducing a short toehold, we were able to accelerate strand displacement and thereby drive the equilibrium toward fragment dissociation, enabling rapid and controlled deactivation of inhibition. This earlier work drew inspiration from peptide fragments derived from blood‐feeding organisms. Hirudin, isolated from the salivary glands of the medicinal leech, is a historically important bivalent thrombin inhibitor that binds both the active site and exosite I (fibrinogen binding site) to achieve potent inhibition [31]. This protein has inspired the development of clinically approved drugs such as bivalirudin (ATC: B01AE06), used in patients with heparin‐induced thrombocytopenia (HIT) and in percutaneous coronary interventions (PCI). Several other anticoagulant proteins from blood‐feeding organisms have been characterized [32, 33, 34, 35], and recent rational design efforts have yielded engineered ultrapotent chimeric inhibitors that engage three distinct sites on thrombin (the active site, exosite I, and exosite II) [36].

We reasoned that reversible thrombin inhibitors are particularly well‐suited to a DCC approach: the PNA exchange kinetics enable unbiased equilibration of library composition, while exchange of thrombin‐bound inhibitors remains slow. This feature allows the target itself to amplify the best binders from an otherwise equilibrated pool and should allow a screen of unprecedented scope. We designed a three‐component DCC system to explore optimal fragment combinations for engaging the active site, exosite I, and exosite II, incorporating both peptide and small‐molecule fragments. Using a set of 50 fragments for each position, the resulting virtual library samples a combinatorial space of 125 000 unique assemblies.

## 2 Results and Discussion

### 2.1 Screen Validation

**FIGURE 2:**
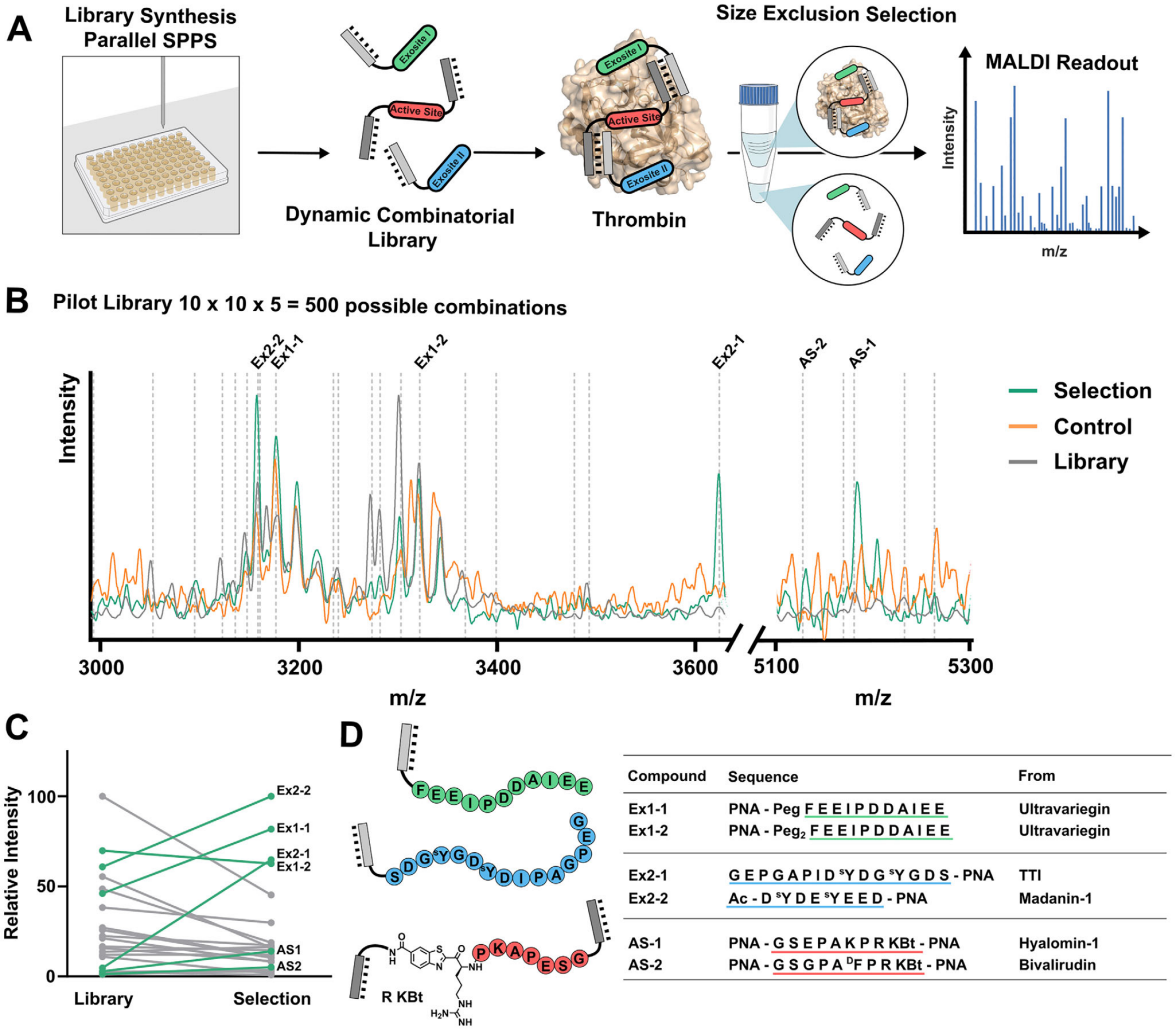
Selection of pilot library. (A) Schematic workflow used. (B) MALDI spectrum for library (grey), blank selection (red), and thrombin selection (red). Dotted lines represent individual members. (C) Processed results showing relative abundance of fragments following the size exclusion selection. (D) Schematic representation of one known fragment for each binding site, with the full sequence shown in the table. *KBt: keto benzothiazole, ^s^Y: sulfo tyrosine mimic, ^D^F: d‐phenyl alanine*.

Expanding from a two‐fragment to a three‐fragment assembly markedly increases the combinatorial diversity of a dynamic library, from scaling with the square to scaling with the cube of the number of fragments, while increasing the synthetic and analytical workload by only ∼50%. For example, using 100 variants per fragment yields 10 000 possible combinations in a two‐fragment system, but 1 000 000 in a three‐fragment design. To validate this concept before constructing the full library, we designed a pilot 10 × 10 × 5 library targeting thrombin's exosite I, exosite II, and active site, respectively. The active site fragment is functionalized with two identical PNAs such that it can bridge the assembly of the three fragments. Each sublibrary incorporated known binders for its respective site while the remaining sequences contained point mutations at key positions expected in some cases to impair binding, serving as negative controls (Figure 2): exosite II fragments inspired by the tsetse fly thrombin inhibitor (TTI) and Madanin‐1 [37, 38, 39] as well as a known exosite I binding fragment, termed ultravariegin [40]. This pilot library comprised 500 total combinations, of which eight could be anticipated to yield functional assemblies.

Modifications were required to render the active site fragment amenable to hybridization with both exosite I and exosite II fragments. A new ketobenzothiazole warhead (KBt) with an additional vector on the 6‐position of the benzothiazole was synthesized (Figure 2D, see Supporting Information for exact structures of all compounds used in this study). Our prior work had shown improvement when the PNA was lengthened from 6‐ to 8‐mer. However, a 6‐mer PNA was preferred for the library in order to favor dynamic assembly and shuffling between the fragments. Keen to retain the simplicity of the assemblies and their synthesis, we decided to readout our library members post‐selection via mass spectrometry. Despite generating numerous assemblies, the library retains discrete molecular fragments, each identifiable by its unique, pre‐defined molecular weight.

Traditional affinity‐selection protocols often rely on immobilizing the target protein on solid supports to capture binders from solution, followed by sequential washing and elution steps. However, thrombin—an active serine protease—is ill‐suited for covalent immobilization due to the risk of inactivating or perturbing the active site. As an alternative, we adopted a solution‐phase selection approach based on size‐exclusion filtration, chosen for its simplicity and speed, combined with mass spectrometry readout.

Initial trials using conventional size exclusion high‐performance liquid chromatography (SEC‐HPLC) proved impractical due to sample dilution and high protein requirements. We therefore employed size‐exclusion spin filters to enrich thrombin–binder complexes while simultaneously concentrating the samples for analysis. Given the molecular weights of thrombin (∼36 kDa) and the assembled inhibitors (∼11 kDa), a 30 kDa molecular‐weight cutoff (MWCO) filter was selected. The library members were incubated for 15 min with thrombin at a 1:1 ratio (500 nM thrombin and 500 nM per fragment, 15 µL) to ensure selection of only the tightest binders. Following dilution with PBS to 18 nM (400 µL added), the solution was spun for 15 min at 14 000 *g*. After a single centrifugation, the retained solution was spotted on matrix assisted laser desorption/ionization (MALDI) and the relative intensity of each peak was analyzed relative to a control performed without thrombin. The whole selection process required < 1 h (Figure 2). Using the relative intensity of each peak, a slope of enrichment was calculated with values ranging from ‐55 (negative selection) to 60. Gratifyingly, all fragments with a positive slope are part of positive controls with established affinity to thrombin (Figure 2C). While the activity of all other fragments is not known, **Ex1‐7** bearing the most mutations at a structurally validated hotspot was strongly negatively selected, confirming the integrity of the selection method. Throughout this study, fragments are denoted **Ex1‐X** for exosite I, **Ex2‐X** for exosite II, and **AS‐X** for active‐site fragments. The entire selection and readout process required less than 1 h (Figure 2).

### 2.2 Screen of 125 000 Library

**FIGURE 3:**
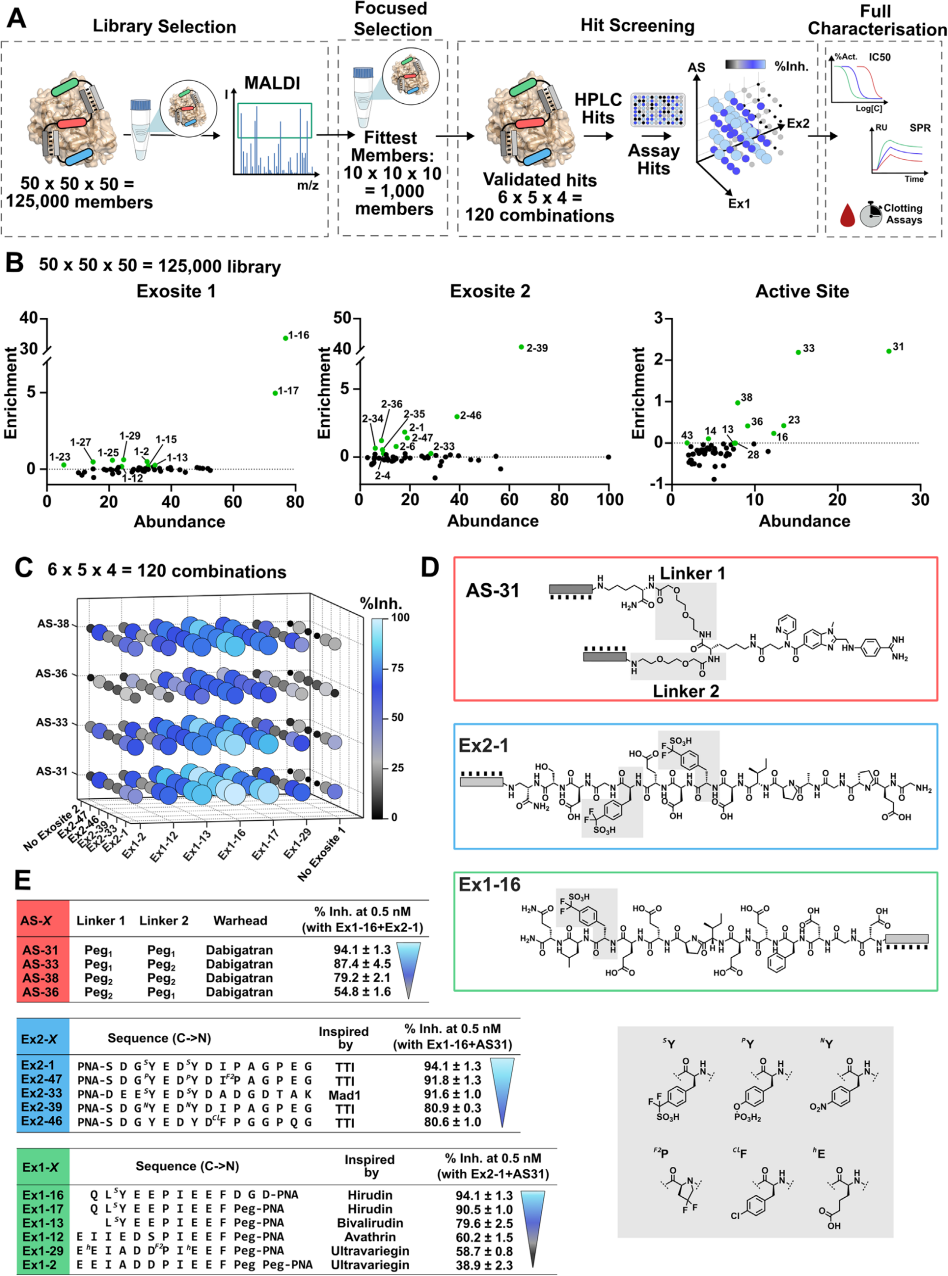
125 000 library and validation. (A) Schematic workflow of library selection and hit validation. (B) Processed results of 125 000‐member library selection. (C) Assay‐based hit validation of the best fragments of the focused library (*n* = 2 replicates). (D) Schematic representation of one known fragment for each binding site with hotspot modifications in grey (^s^Y and *^p^* Y will be in ionic form under the assay conditions). (E) Full sequences of the hits with their % inhibition (*n* = 2 replicates, with data points presented as mean values ± s.d.). Structures and code of unnatural building blocks found in hits are shown in the grey box.

Following the success of the pilot library, a 50 × 50 × 50 = 125 000‐member combinatorial library was designed incorporating a diverse range of chemical space with improved stability, which included known binders, small molecule inhibitors targeting the active site, linear peptides, cyclized peptides whilst incorporating many unnatural amino acids (see Tables S1–S3, for the structures of each fragment and Figure S1 for sequence alignment of known binders). Each member was designed to have a unique molecular weight and could be tuned by incorporating tags (Arg, Lys, PEG, Gly, Ala, Ac) of different masses at the end of the PNA. This simple design provided a minimum mass separation of 4 Da, which was deemed important considering isotopic distributions of the constructs. As the members are prepared via solid‐phase peptide synthesis (SPPS) without purification, they were easily prepared in parallel on an automatic synthesizer (Figure 3A). The selection process mentioned above was performed, and the starting library, thrombin selection, and blank selection were analyzed by MALDI‐TOF spectrometry. The selection results were analyzed by corrected enrichment (enrichment in selection minus enrichment in control selection) versus abundance (intensity) in order to identify inaccurate enrichment factors caused by low abundance, grouping each fragment by binding site (Figure 3B). Gratifyingly, enrichments exceeding 30‐fold were observed for members of both exosites, while the active site library contained more clustered results; this was expected since many fragments contain a suitable active pharmacophore (arginine in position P1). Notably, **AS‐50**, a negative control in the library with no arginine in the P1 position, had the strongest negative enrichment. Importantly, the MALDI spectra from biological replicates of selection were nearly identical, despite the complexity of the experiment. It is also important to point out that the fittest combination cannot be ascertained from the result at this stage. The top 10 fragments of each subset were used for a second round of selection, which is intrinsically more stringent since all fragments are putative binders. A practical constraint of the present MALDI‐based deconvolution strategy is that each library member must be uniquely identifiable by mass. Accordingly, the scalability of direct mass‐based decoding is ultimately limited by the accessible mass window and by the extent of spectral overlap that can be tolerated. The use of focused sublibraries, however, provides an opportunity to accommodate a greater degree of mass redundancy by enabling recursive deconvolution at the focused‐library stage. Alternatively, tandem mass spectrometry (MS/MS) could be employed for peptide‐based fragments to further expand the scope of deconvolution.

From this focused selection (10 × 10 × 10 = 1000 combinations), six exosite I fragments, five exosite II fragments, and four active site fragments were significantly enriched with adequate abundance (Figure S3). Whilst self‐assembled libraries and MALDI readout is beneficial for its simplicity and its ease of synthesis, this affinity‐based selection may not correlate directly to inhibitory activity. To retrieve this information, the best combinations must be tested in an enzymatic assay using a fluorogenic substrate. The hits from the focused library yielded 120 possible combinations (6 × 5 × 4) which can easily be tested in 96‐well plate format. To this end, each combination was tested, in duplicate, for thrombin inhibition at a single concentration (0.5 nM) (Figure 3C,E). A low concentration was chosen in order to differentiate the best assemblies from the good assemblies therefore validating the combinations yielding the strongest inhibition. At this concentration, a clear differentiation was observed between monovalent (<5% inhibition for **AS‐31**, the fittest active site fragment), bivalent (78 and 42% inhibition for **AS‐31+Ex1‐16** and **AS‐31+Ex2‐1**, respectively) and trivalent (94% inhibition **AS‐31+Ex1‐16+Ex2‐1**). The library identified new combinations: the active site fragment hits all incorporated a small molecule drug named dabigatran (Figure 3D), whilst the best exosite II fragments were inspired by peptides from Hematophagous Arthropods [TTI (Tsetse fly Glossina: **Ex1‐2**, **Ex2‐47**, **Ex2‐39** and **Ex2‐46** and madanin‐1 (the bush tick Haemaphysalis): **Ex2‐33**]. Many of these natural anticoagulants have sulfated tyrosine residues which increase their affinity [35, 41, 42]. Interestingly, some of the hits include other negatively charged residues including phosphate (**Ex1‐47**) and nitro (**Ex2‐39**) groups. The exosite I hits showed more variability in potency with sequences inspired by hirudin being preferred (**Ex1‐16**, **Ex1‐17** and **Ex1‐13**). The combinatorial output of this library has provided a wide range of structure activity relationships (SAR) and identified potent trivalent assemblies with strong cooperative binding of all three fragments. The best hit combination **Ex1‐16+Ex2‐1+AS‐31** shows almost stoichiometric inhibition of thrombin (>90% inhibition at 0.5 nM assembly for 0.2 nM thrombin).

**FIGURE 4:**
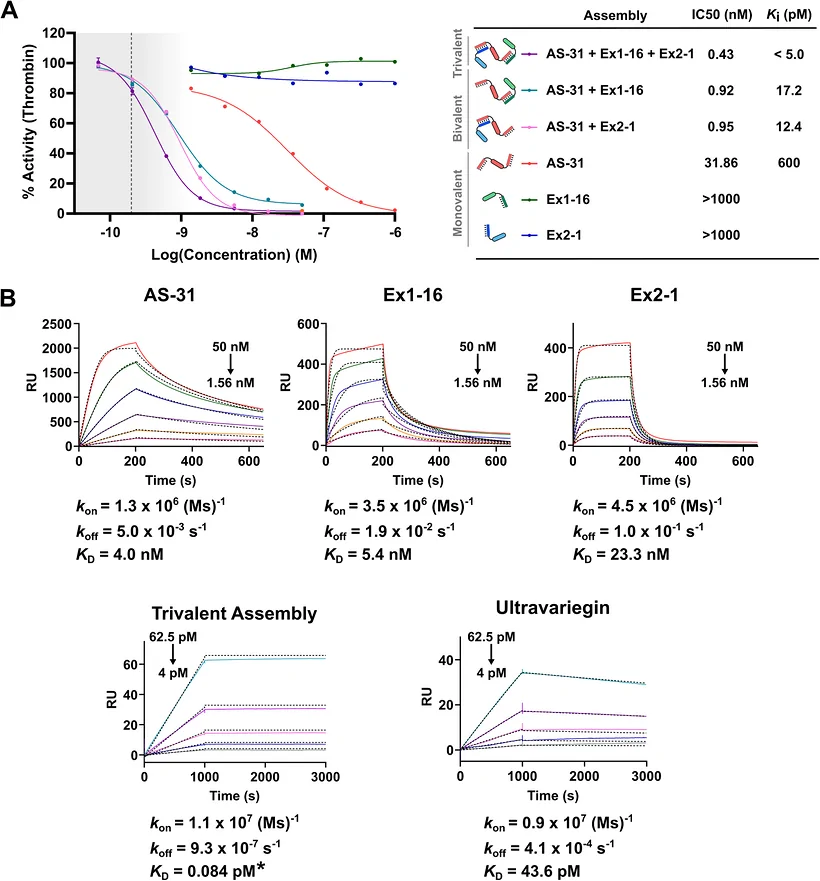
Full characterization. (A) IC_50_ curves of the best trivalent inhibitor (**AS31** + **Ex1‐16** + **Ex2‐1**) with the bivalent and monovalent assemblies as controls (*n* = 2 replicates, with individual data points presented as mean values ± s.d.). Vertical dotted line in the IC_50_ plot marks the concentration of thrombin. (B) SPR curves and binding constants of individual fragments, trivalent assembly, and Ultravariegin. *The binding constants of the trivalent assembly cannot be accurately determined due to limited dissociation.

The best‐performing assembly (**Ex1‐16+Ex2‐1+AS‐31**) was fully characterized in an inhibition assay, with the trivalent assembly yielding an apparent *K*_i_ of 4.6 pM when analyzed using the Morrison tight‐binding model. However, given that the enzyme concentration used was 200 pM, the [*E*]_t_/ *K*_i_ ≈ 50, lies well beyond the reliable limits of the model, suggesting a near‐stoichiometric interaction. When considered through the lens of the Cheng–Prusoff relationship [*K*_i_ = IC_50_/(1+([S]/*K*_M_)), using the enzyme concentration as the lowest possible value of IC_50_ sets the boundary of accurate measurement to 4.0 ± 0.6 pM, below which the inhibitor is sub‐stoichiometric relative to the enzyme. We therefore regard any value below ∼5 pM as beyond the range of reasonable experimental determination. Nevertheless, the IC_50_ curves of either bivalent assembly (**AS‐31**+**Ex1‐16** or **AS‐31**+**Ex2‐1**) are clearly right‐shifted relative to the trivalent construct, yielding extrapolated *K_i_* of 17.2 and 12.4 pM, respectively (Figure 4A). The active site fragment alone (**AS‐31**) has a *K_i_* of 600 pM, clearly pointing to the fact that full dissociation of the fragment would abolish inhibitory activity, even at the IC_90_ concentration of the trimeric assembly. Interestingly, it seems that the exosite I binder (**Ex1‐16**) activates thrombin activity, which yields a double‐edged sword when implemented into an assembly. This is not totally surprising, as exosite I is where the natural substrate, fibrinogen, binds, resulting in increased enzymatic activity as observed in the fluorogenic substrate assay used. Collectively, the data support the effectiveness of this selection process to guide the assembly of the strongest binder, as a trivalent assembly. It should be noted that, for simplicity, the active site fragment is conjugated to two units of the same PNA sequence in order to form a duplex with exosite I and II fragments, which have the same PNA sequence. Thus, the virtual library also contains assemblies composed of Ex1:AS:Ex1 and Ex2:AS:Ex2 in addition to the sought Ex1:AS:Ex2 assemblies. Given that such assemblies would not likely capture the benefit of interactions at the three sites, they were anticipated to yield lesser affinity and were not considered in the calculation of the virtual library size (500 000 possible combinations taking these assemblies into consideration).

Considering the limitations of *K*_i_ determination by enzymatic assays, we turned to surface plasmon resonance (SPR) to directly measure binding affinities. Following the same rationale as in the selection, thrombin was kept in solution, and the fragments were immobilized via a biotin modification on the *N*‐terminus of the PNA or via PNA hybridization with a PNA on a chip (see Supporting Information Section S10 for further details). As shown in Figure 4B, affinities of 4.0, 5.4, and 23.3 nM were measured for the **AS‐31**, **Ex1‐16** and **Ex2‐1**, respectively. All the fragments have comparable association kinetics (*k*_on_ = 1.3–4.5 × 10^6^ (Ms)^−1^) but broader dissociation kinetics ranging from 0.5–10 × 10^−2^ s^−1^. However, when the three fragments were combined, the calculated *K*_D_ decreased dramatically to 84 fM, with dissociation kinetics that approaching the limitation of the instrument accuracy (*k*_off_ = 9.3 × 10^−7^ s^−1^). As a comparison, ultravariegin had a *K*_D_ of 44 pM, compared to an extrapolated *K*_i_ of 4 pM using the tight‐fitting model of enzymatic kinetic measurements (assays performed at 0.83 nM of thrombin) [40]. The binding affinity of this trimeric assembly measured by SPR is within the range of hirudin (20 fM *K*_i_ calculated using extrapolation from tight‐binding model [43]). This places the affinity of the trimeric assembly in the realm of therapeutic proteins and computationally designed miniproteins [44, 45]. It is also interesting to note that in the absence of thrombin, the duplex dissociation (*k*_off_ = 2.2 × 10^−4^ s^−1^) is >200 times faster while the slowest dissociation fragment has a *k*_off_ = 0.5 × 10^−2^ s^−1^, >1000 times faster. These results underscore the cooperativity between the dynamic interaction of each fragment with thrombin and the hybridization. The rebinding or rehybridization events occur faster than dissociation, producing an apparent covalent‐like stability. It is conceivable that the double stranded PNA (dsPNA) scaffold participates in fortuitous interactions with the protein surface, thereby contributing to the high degree of synergy observed between fragment pairs. Regrettably, our efforts to obtain structural information from co‐crystal structures have thus far been unsuccessful, including in earlier studies involving related PNA–peptide conjugates [46, 47]. The present work employs L‐𝛄‐modified PNA that form a right‐handed helix a duplex and it is possible that a different helical conformation has an impact [48, 49, 50]. More broadly, if productive interactions between the protein surface and the dsPNA scaffold do occur, chemically modified PNA scaffolds could in principle be used to optimize them. Consistent with this possibility, a previous study on related conjugates showed that the nature of the nucleic acid backbone (arginine‐modified PNA versus anionic DNA) influenced binding affinity [51], supporting the idea that the duplex scaffold can contribute productively to protein recognition.

**FIGURE 5:**
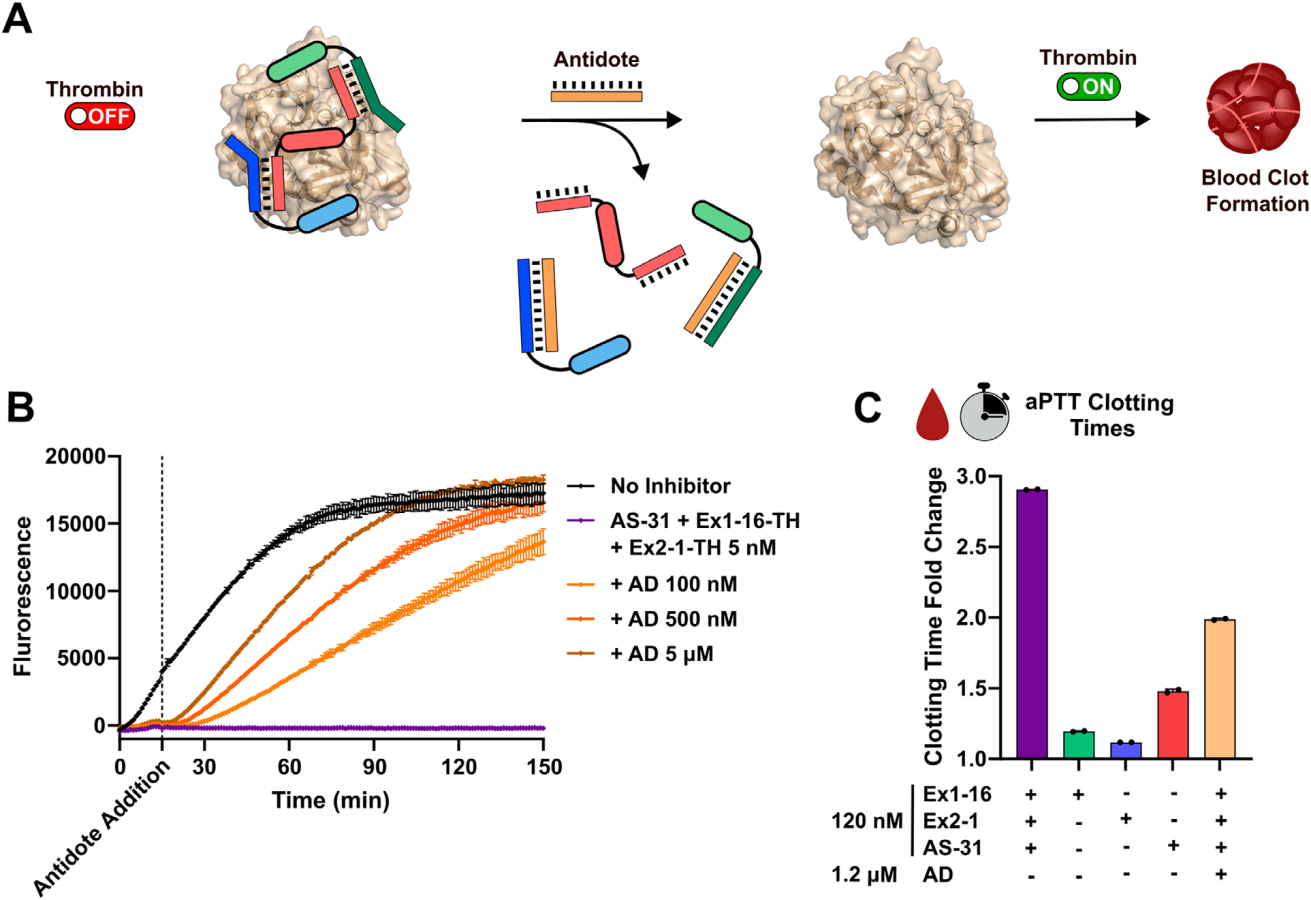
Reversal of inhibition in vitro and in blood. (A) Mechanism of the reversal scheme. (B) Reversal of inhibition of the trivalent inhibitor with additional toehold (TH) sequences on the exosite PNAs (**AS31** + **Ex1‐16‐TH** + **Ex2‐1‐TH**), monitored by fluorescence recovery upon antidote (**AD**) addition after 15 min (*n* = 2 replicates, with individual data points presented as mean values ± s.d.). (C) Prolongation of clotting times with **AS31**+**Ex1‐16‐TH**+**Ex2‐1‐TH** with and without antidote and the individual fragments in human plasma (aPTT assay) (*n* = 2 replicates, with individual data points presented as mean values ± s.d.).

Importantly, this inhibition remains reversible. As shown in Figure 5A, introducing a four‐residue toehold to each exosite‐binding PNA, and leveraging their identical nucleotide sequences, enables a single complementary PNA strand to displace the hybridization within the trimeric assembly–thrombin complex, thereby restoring enzymatic activity. Addition of 5 µM of antidote (AD) restores 97% activity within 10 min (Figure 5B, Figure S5, and Table S1) while 500 nM restores 73% activity within 15 min. While on a molar equivalent basis, this represents 1000 and 100 equiv of antidote. The apparent need for a large excess of antidote is driven by the kinetics of a second‐order rate constant of the strand displacement. The observed reversal rates are consistent with a strand displacement rate expected for a four‐nucleotide overhang (*k* = 10^3^–10^4^ M^−1^s^−1^).

We next turned to the activated partial thromboplastin time (aPTT) assay to assess both the efficacy of the inhibitor and its reversibility in a clinically relevant setting. While anticoagulants are indispensable for preventing thrombotic events and have contributed substantially to increased life expectancy in at‐risk patients, their use carries a significant risk of hemorrhage in case of trauma or other adverse event and adds significant complications to unplanned surgical interventions [52, 53]. The capacity to rapidly reverse anticoagulation therefore represents a major therapeutic advantage. As shown in Figure 5C, 120 nM of inhibitor was sufficient to produce a nearly three‐fold prolongation in clotting time, which represents the upper limit sought in a clinical setting. Addition of 10 equiv of antidote (5 functional equivalents since full disassembly requires two equivalents) brought the clotting time down to twofold, consistent with a reversal of activity. It should be noted that at this high inhibitor concentration, the active site alone yields a 1.5‐fold extension in the clotting time.

## 3 Conclusion

Our results demonstrate that dynamic combinatorial chemistry (DCC) guided by short hybridization tags enables rapid exploration of a vast chemical diversity space and yields hits inherently suited for on‐demand reversible pharmacology. Target‐templated amplification within a 125 000‐member library, while modest in scale compared to current DNA‐encoded libraries, is offset by the exceptional speed and simplicity of the MALDI‐MS readout, allowing screening and decoding to be completed within hours. The identified hits exhibit an affinity comparable to that of rationally designed miniproteins or even monoclonal antibodies. Remarkably, despite its sub‐nanomolar binding, activity reversal remains possible and operates efficiently in plasma, as demonstrated by the aPTT assay. Although the present study does not address fragment serum stability or pharmacokinetic behavior, the clinical success of GLP‐1 agonists provides an important precedent, offering solutions to challenges that historically limited the development of peptides as therapeutics.

## Author Contributions

M.D. conceived and performed the synthesis of all compounds reported and biochemical experiments. R.P. and N.W. conceived the work; NW supervised the work. The manuscript was written by M.D., R.P., and N.W.

## Conflicts of Interest

The authors declare no conflicts of interest.

## Supporting information



Materials and methods, including information on chemicals, synthesis procedures, analytical results (MS, HPLC, NMR), selection data and thrombin assays. The raw data of analyses is available on www.zenodo.org, https://doi.org/10.5281/zenodo.19555395 **Supporting File**: anie73632‐sup‐0001‐SuppMat.pdf.

## Data Availability

The data that support the findings of this study are openly available in Zenodo at https://zenodo.org/, reference number 10.5281/zenodo.19555395.
