# Supplementary material for: Discovery of a Reversible Sub‐Picomolar Thrombin Inhibitor Using DCC
Source: Angew Chem Int Ed Engl. 2026 Jul 15;65(38):e5778436. doi: 10.1002/anie.5778436 (PMC13573113; doi:10.1002/anie.5778436)
Supplement: Supplementary file 1 [file ANIE-65-e5778436-s001.pdf]

Discovery of a Reversible Sub-Picomolar Thrombin Inhibitor using DCC

Supplementary Information

Table of Contents

1. **Acronyms** 3
2. **Supplementary Figures** 4
3. **General methods** 6
4. **Synthesis of compounds** 7
5. **Synthesis of Arg(Pbf)-Benzothiazole-N_3** 10
6. **Selection Procedure** 14
7. **10x10x5 Pilot Library** 15
8. **50x50x50 Duplicate Spectrums** 15
9. **Thrombin Inhibition Assay** 16
10. **SPR Experiments** 18
11. **aPTT Experiments** 19
12. **Library Members and characterisation** 20
13. **Final compound characterisation** 53
14. **References** 64

1. Acronyms

CuAAC	Copper(I)-catalyzed azide-alkyne cycloaddition
DAP	Diaminopropionic acid
DCE	1,2-dichloroethane
DCM	Dichloromethane
DHB	2,5-Dihydroxybenzoic acid
DIPEA	N,N-Diisopropylethylamine
DMF	Dimethylformamide
DMSO	Dimethyl sulfoxide
EDC	1-Ethyl-3-(3-dimethylaminopropyl)carbodiimide
ESI	Electrospray ionization
Fmoc	Fluorenylmethoxycarbonyl
HATU	Hexafluorophosphate Azabenzotriazole Tetramethyl Uronium
HIFP	Hexafluoroisopropanol
HOBt	Hydroxybenzotriazole
HPLC	High performance liquid chromatography
HRMS	High-resolution mass spectra
LCMS	Liquid chromatography-mass spectrometry
MALDI	Matrix-assisted laser desorption/ionization
MS	Mass spectrometry
NaAsc	Sodium ascorbate
NMP	N-Methyl-2-pyrrolidone
PEG	Polyethylene glycol
PNA	Peptide Nucleic Acid
TBTA	Tris((1-benzyl-4-triazolyl)methyl)amine
TFA	Trifluoroacetic acid
UHPLC	Ultra High performance liquid chromatography

2. Supplementary Figures

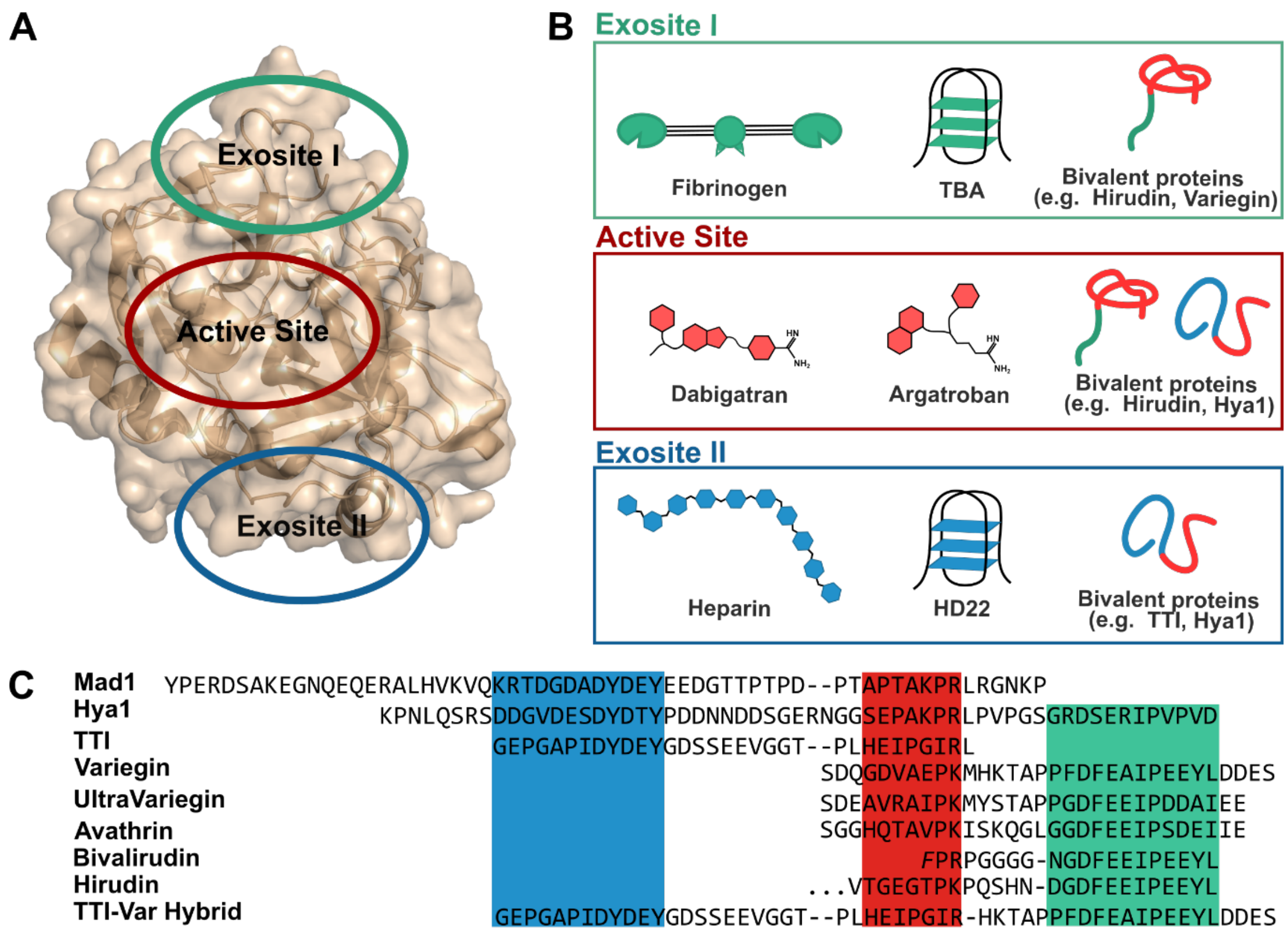

Supplementary Figure S1. Known structures that bind to specific binding pockets of thrombin. **A**. Schematic representation of thrombin's three binding sites. **B.** Exemplar ligands to each binding site. **C.** Sequence alignment of proteins/peptides from blood sucking insects or inspired by them.

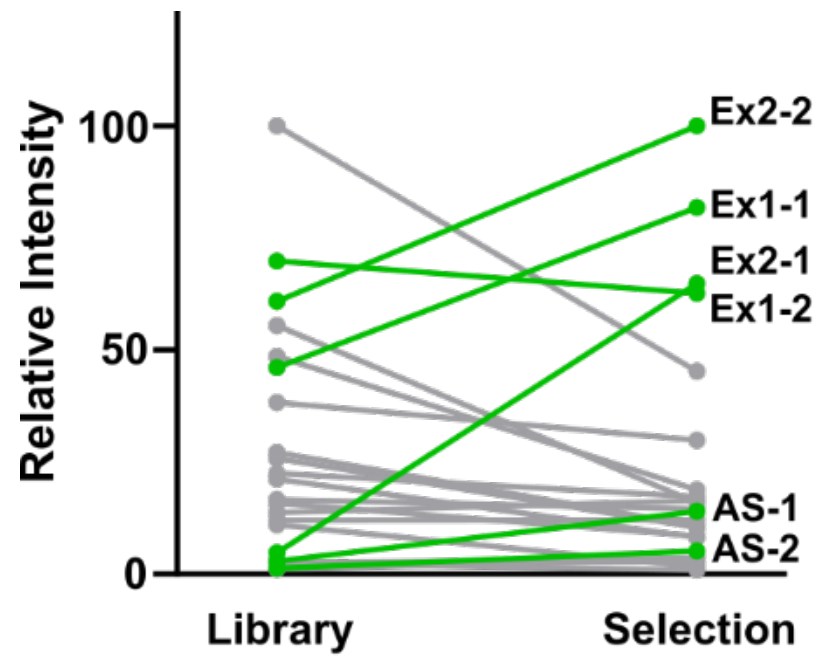

AS#	Molecular Weight	Slope	Intensity
AS-1	5181.23	11.10	13.96
AS-2	5128.17	3.83	5.11
AS-3	5233.26	-1.45	0.85
AS-4	5264.28	0.37	2.77
AS-5	5170.25	2.32	3.41

Ex1-#	Molecular Weight	Slope	Intensity
Ex1-1	3177.20	35.69	81.78
Ex1-2	3322.36	-7.20	62.62
Ex1-3	3148.17	-8.49	29.80
Ex1-4	3095.29	2.70	16.38
Ex1-5	3240.44	-5.20	17.22
Ex1-6	3235.52	0.15	12.18
Ex1-7	3302.51	-54.72	45.28
Ex1-8	3161.30	ND	ND
Ex1-9	3274.42	-5.20	15.87
Ex1-10	3136.34	-2.10	14.54

Ex2-#	Molecular Weight	Slope	Intensity
Ex2-1	3624.49	60.13	64.86
Ex2-2	3158.94	39.07	100.00
Ex2-3	3398.64	-9.42	1.61
Ex2-4	2993.19	0.74	3.25
Ex2-5	3368.48	-12.99	8.21
Ex2-6	3492.59	-7.38	8.44
Ex2-7	3282.49	-29.83	18.81
Ex2-8	3053.17	-15.41	10.35
Ex2-9	3123.26	-16.46	10.65
Ex2-10	3477.75	1.96	3.69

Supplementary Figure S2. 10x10x5 Pilot Library. Data used to produce **Figure 2C** with the molecular weight of each member, the slope and their intensity.

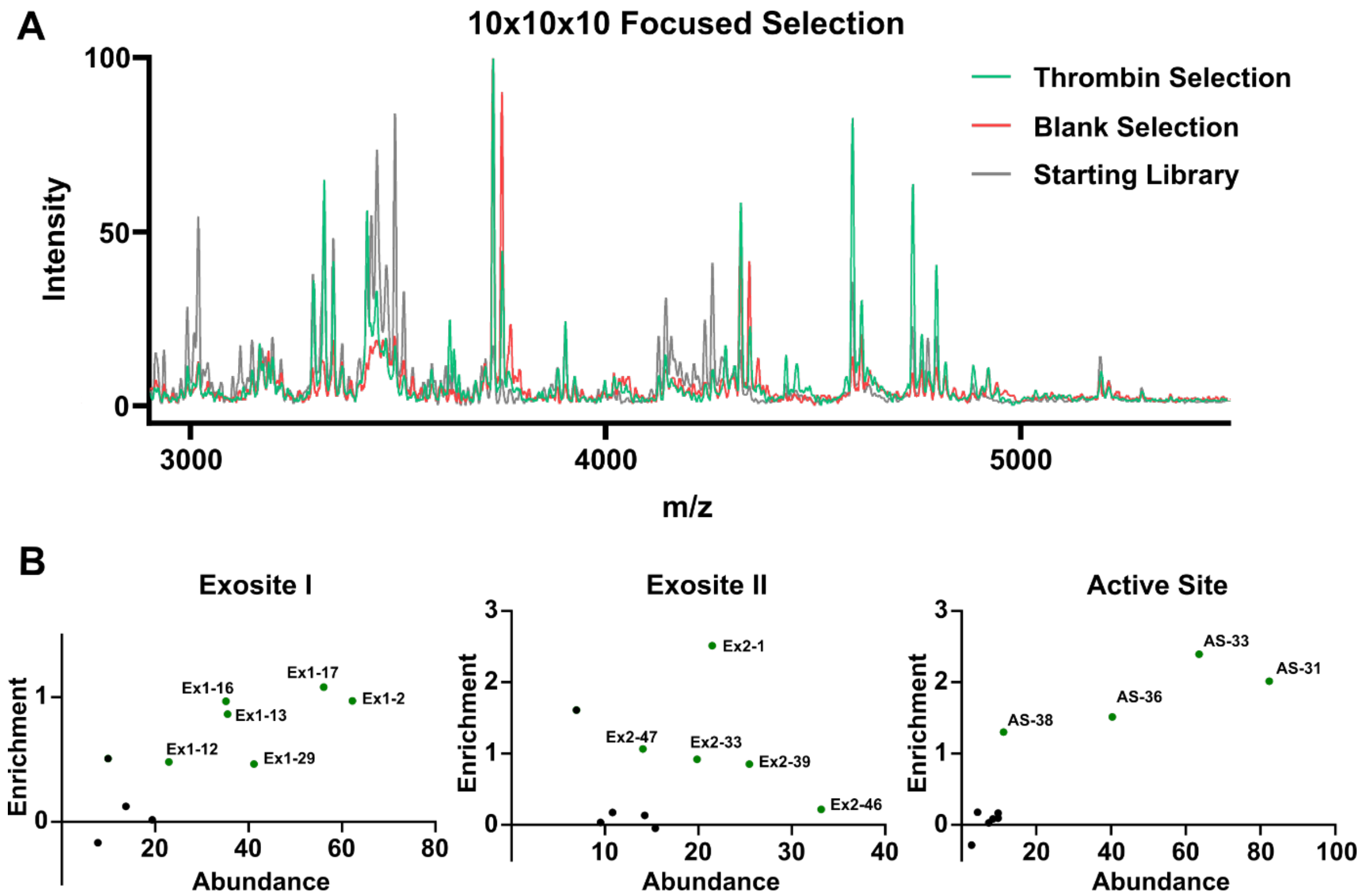

Supplementary Figure S3. 10 x 10 x 10 focused selection results and analysis

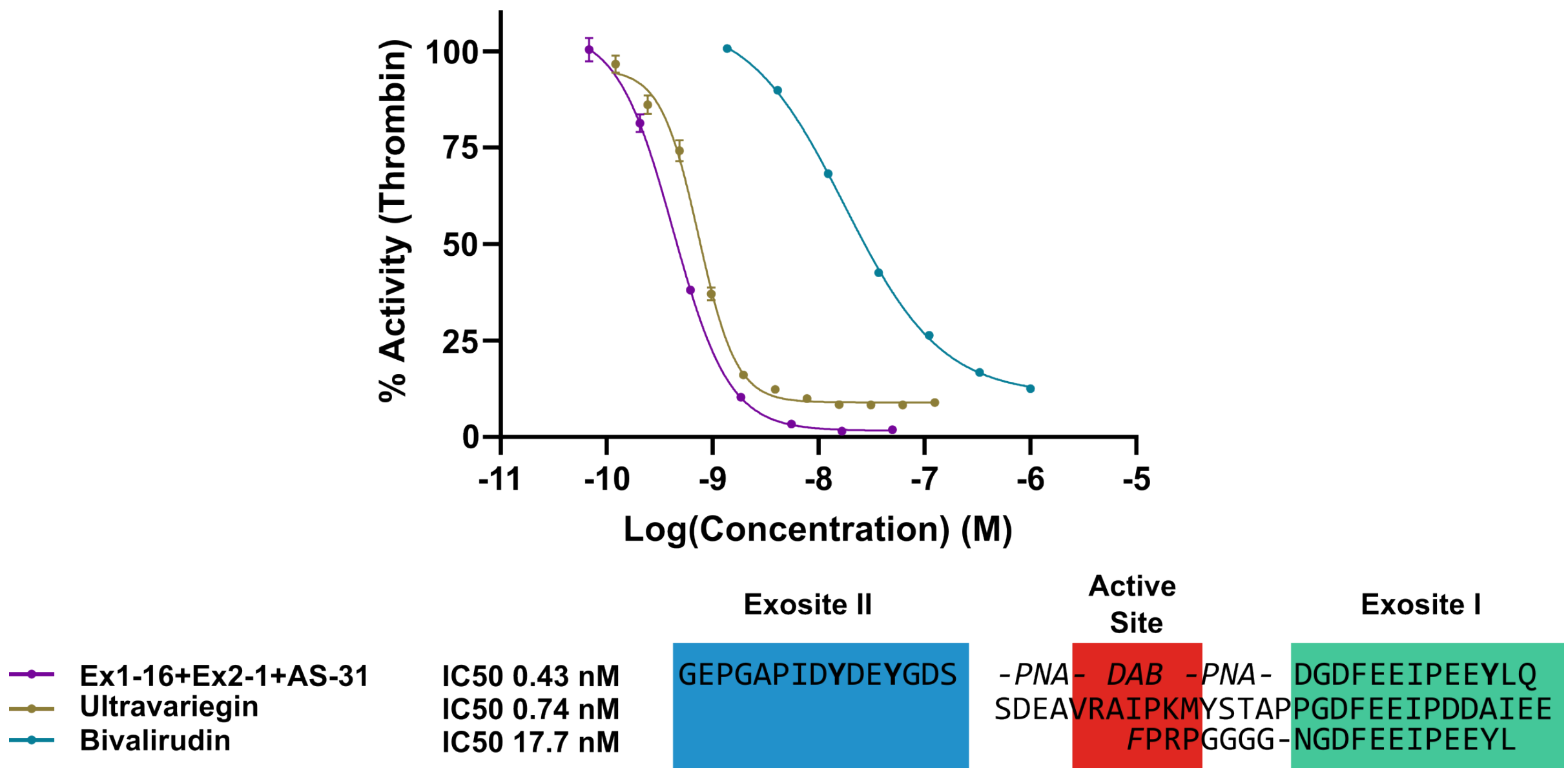

SI Figure S4: Benchmarking against ultravariegin and bivalirudin. *DAB:* Dabigatran; *PNA:* peptide nucleic acid; The sulfated tyrosines are shown in bold.

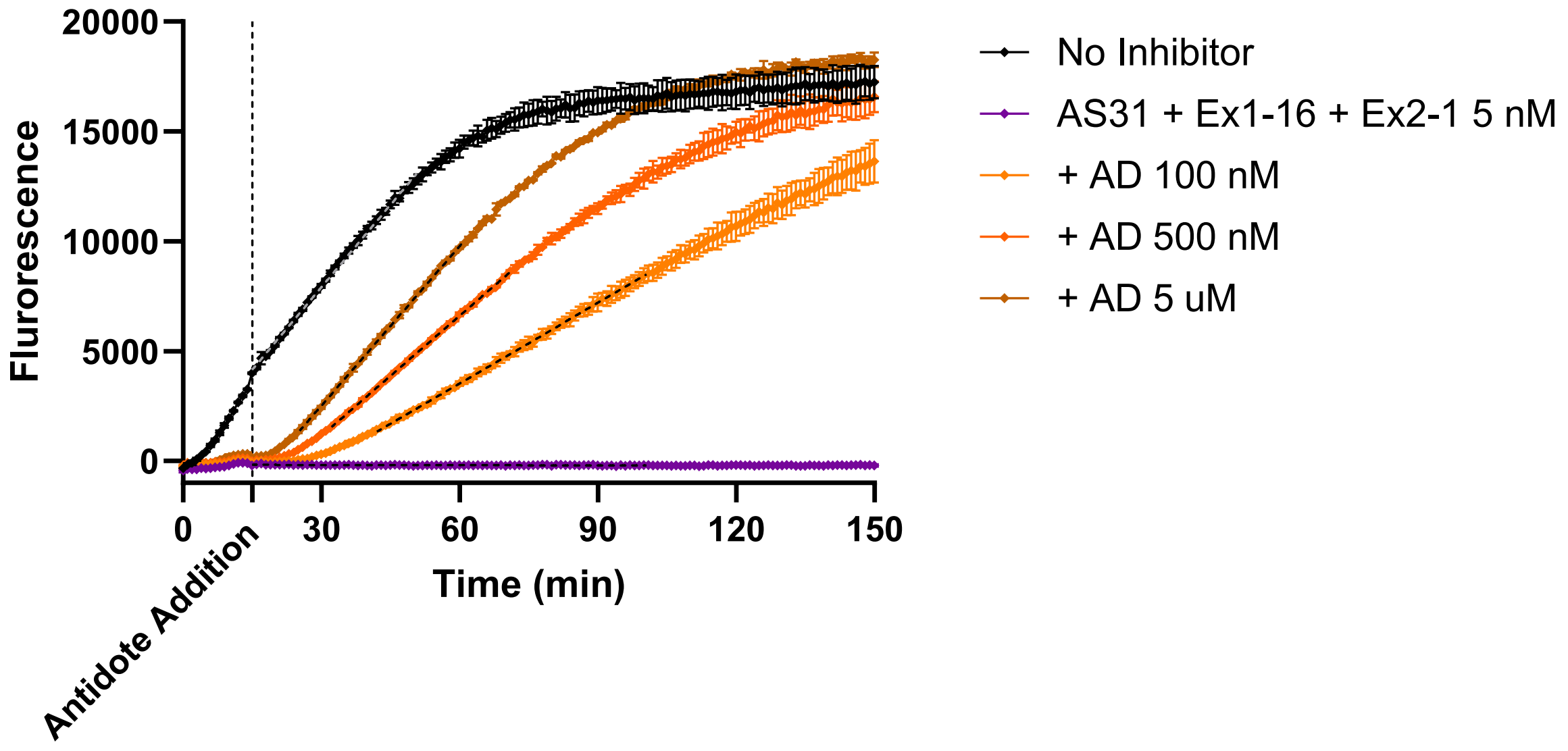

SI Figure S5: Reversal of inhibition of the trivalent inhibitor with additional toehold (TH) sequences on the exosite PNAs (**AS31** + **Ex1-16-TH** + **Ex2-1-TH**), monitored by fluorescence recovery upon antidote (**AD**) addition after 15 minutes (*n* = 2 replicates, with individual data points presented as mean values ± s.d.). Thrombin activity is relative to the slope and the recovery of activity upon antidote addition is estimated by the linear sections of the curves shown in dotted lines.

SI Table S1: % Reversal as estimated from SI Figure S5.

	% Reversal	Slope	Linear Range (after AD addition)	R^2
No Inhibitor	NA	250.3 ± 1.88	0 – 35 minutes	0.996
AS31 + Ex1-16 + Ex2-1	0 %	-0.33 ± 0.08	0 – 85 minutes	NA
+ AD 100 nM	49 %	122.2 ± 0.85	25 – 85 minutes	0.994
+ AD 500 nM	73 %	182.1 ± 0.50	15 – 55 minutes	0.999
+ AD 5 µM	97 %	242.2 ± 0.79	10 – 45 minutes	0.999

3. General methods

All reagents and solvents for the organic synthesis were purchased from commercial sources and were used without further purification. HPLC purification was performed with an Agilent Technologies 1260 infinity HPLC using a COSMOSIL 5C8-MS column (120Å, 5 µm, 10.0 x 150 mm). LC-MS spectra were recorded on a DIONEX Ultimate 3000 UHPLC with a Thermo LCQ Fleet Mass Spectrometer System using PINNACLE DB C18 column (1.9 µm, 50 x 2.1 mm) operated in positive mode. All the LC-MS spectra were measured by ESI. MALDI-TOF Mass spectra were measured using a Bruker Daltonics Autoflex spectrometer operated in positive mode. High-resolution mass spectra (HRMS) were obtained on a Xevo G2 Tof spectrometer (Ionization mode: ESI positive polarity; Mobile phase: MeOH 100 µl/min). Automated solid-phase synthesis was carried out on an Intavis AG Multipep RS instrument. All amino acids, natural and unnatural were purchased from commercial unless mentioned otherwise.

4. Synthesis of compounds

5.0 mg of resin was swollen in DCM for 10 minutes and washed twice with DMF. Iterative cycles of amide coupling (**Procedure 1**), capping of the resin (**Procedure 4**), and deprotection of the protecting group (**Procedure 2 or 3**) were done to synthesize the PNA probes. The compounds were deprotected and cleaved from the resin using **Procedure 5** and finally purified using HPLC.

2-Chlorotrityl Chloride Resin Loading

2-Chlorotrityl chloride resin (1.46 mmol/g loading) was swollen in dry DCM for 30 min, followed by washing with DCM+1% DIPEA (1 x 3 mL) and DCM (10x 3 mL). A solution of Fmoc-Xaa-OH (0.7 mmol/g resin) and DIPEA (4 eq. relative to resin functionalization) in DCM (final concentration 0.125 M of amino acid) was added to the resin, which was shaken at room temperature for 16 h. The resin was then washed with DCM (5x 3 mL), DMF (5x 3 mL) and DCM (5x 3 mL). The resin was then capped via treatment with 17:2:1 v/v/v DCM:MeOH:DIPEA (5 mL) for 40 mins at room temperature. The resin was then washed again with DCM (5x 3 mL), DMF (5x 3 mL) and DCM (5x 3 mL) prior to further use.

Rink Amide Resin Loading

Nova PEG® Rink amide resin (0.44mmol/g, NovaBiochem) was swollen in DCM for 10 minutes and washed twice with DMF. Standard amide coupling (**Procedure 1**) was performed, followed by capping of the resin (**Procedure 4**). The resin was then washed again with DCM (5x 3 mL), DMF (5x 3 mL) and DCM (5x 3 mL) prior to further use.

Procedure 1 (P1): Amide coupling.

The corresponding Fmoc protected PNA monomer, or amino acid (4.0 equiv., 0.2M in NMP) was incubated for 5 minutes with HATU (3.5 equiv., 0.5M in NMP) and base solution [DIPEA, 1.2M (4.0 equiv.) and 2,6-lutidine 1.8M (6.0 equiv.) in NMP]. The mixture was then added to the corresponding resin. After 20 minutes, the mixture was filtered, the resin was washed with DMF, and a new premixed reaction solution was added to the resin and let react for another 20 minutes. Finally, the resin was washed with 2x DMF, 2x DCM, and 2x DMF.

Procedure 2 (P2): Fmoc deprotection.

A solution of 20% (v/v) piperidine in DMF was added to the resin and allowed to react for 5 minutes. The mixture was then filtered, the resin washed with DMF, and the sequence repeated for another 5 minutes. Finally, the resin was washed with 2x DMF, 2x DCM, and 2x DMF.

Procedure 3 (P3): Mtt deprotection.

A solution (made from 244 mg of HOBt in 10 mL HFIP and 10 mL DCE) was added to the prewashed resin to reach a volume of 10 mL/g of resin and allowed to react for 5 minutes. The solution was flushed, the resin washed with DCM, and the sequence repeated for another 5 minutes. Finally, the resin was washed with 2x DCM and 2x DMF.

Procedure 4 (P4): Capping.

The resin was treated with a capping mixture (0.92 mL acetic anhydride and 1.3 mL 2,6 lutidine in 18 mL DMF: 10 mL of solution/g of resin) for 5 minutes. After flushing the solution, the resin was washed with 2x DMF, 2x DCM, and 2x DMF.

Procedure 5 (P5): Cleavage from the resin and final deprotection.

Resin (5.0 mg, 1.0 µmol) was treated with 125 µL of a mixture of TFA and scavengers (440 µL TFA + 25 mg phenol + 25 µL water + 10 µL triisopropylsilane) for 2 hours. The resin was filtered, washed with TFA (50 µL), and the collected fractions of cleavage product precipitated in cold ether (1.5 mL). After centrifugation, the pellet was vortexed again with cold Et_2O (1.5 mL) and centrifuged (14k rpm). The pellet was dissolved in H_2O/CH_3CN (3:1, 1.5 mL) and lyophilized to obtain a white powder.

Procedure 6 (P6): Microcleavage for quality control.

The minimum number of beads were picked up with a pipette plastic tip and transferred to 50 µL of TFA. The solution was left for 1 hour and transferred to 1.0 mL of ether. The ether solution was kept for 5 minutes at -20 °C and then centrifuged for 5 minutes at 14k rpm. The ether supernatant was removed, and the pellet dissolved in 20 µL 1:1 acetonitrile/water, which was then used to analyze by MALDI and/or LC-MS.

Procedure 7 (P7): On-resin Copper(I)-catalyzed azide-alkyne cycloaddition (CuAAC).

To TBTA (2.0 mg) in 20 µL DMF was added 15 µL $CuSO_4$ (64 mg/mL in H_2O) followed by 50 µL of NaAsc (396 mg/mL in H_2O). Azide-containing peptide (2 eq. in 60 µL DMF) was added to the mixture which was mixed prior to the addition to 5.0 mg alkyne derivatized rink amide resin (0.0022 mmol). After 16 hours of shaking, the mixture was filtered and the resin was washed with 6x 250 µL of sodium diethyl dithiocarbamate 0.02 M in DMF, 6x 250 µL of DMF, 6x MeOH and 6x DCM.

Procedure 8 (P8): Coupling of Fmoc-L-F_2Smp(nP)-OH

Fmoc-L-F_2Smp(nP)-OH was prepared following the procedure described by Dowman *et al.*[2] A mixture of Fmoc-L-F_2Smp(nP)-OH (0.003 mmol, 1.5 equiv.), HOBt (0.003 mmol, 1.5 equiv.) and DIC (0.003 mmol, 1.5 equiv.) were added to the corresponding resin and shaken overnight. The mixture was filtered and the resin was washed with 2x DMF, 2x DCM, and 2x DMF.

Procedure 9 (P9): Coupling of Arg(Pbf)-Benzothiazole-N_3

To 5.0 mg of resin (0.0022 mmol), Arg(Pbf)-Benzothiazole-N_3 (0.0044 mmol, 2 equiv.) and HATU (0.0034 mmol, 1.5 equiv.) in NMP (100 µL) were added followed by DIPEA (0.012 mmol, 6 equiv.). The reaction was shaken for 2 hours. The mixture was filtered, and the resin was washed with 2x DMF, 2x DCM, and 2x DMF.

Procedure 10 (P10): Neopentyl deprotection

The precipitate collected after cleavage and ether precipitation was lyophilized. The remaining solid was dissolved in a solution 1M NH_4Ac and 6M GnHCl and shaken at 37 °C for 2 hours. The solution was then diluted with H_2O/ACN (50:50) and purified by HPLC.

Procedure 11 (P11): Preparation of di-clicked AS members

The PNA-alkyne and peptide-$(N_3)_2$ were prepared as previously described on resin. On-resin CuAAC (**P7**) with the peptide-$(N_3)_2$ in solution was initially attempted and di-clicked product was observed – however the yields were poor and variability between sequences were observed due to equivalence and resin loading being of high importance to obtain the di-clicked product (bridging of two alkyne-PNA on resin). Solution click was therefore favored. Both resins were cleaved, ether precipitated as described above followed by lyophilization. CuSO4 (see amounts below) was added to PNA-alkyne in H_2O, followed by THPTA and NaAsc. The solution was added to peptide-$(N_3)_2$ and shaken at 37°C for 2 hours. The reaction was diluted with H_2O:ACN (1:1, 140 µL) and purified by HPLC directly.

***Supplementary Table S2**: Volumes and concentrations of in-solution CuAAC*

Component	Stock	Volume in Reaction	Final Concentration
Peptide-$(N_3)_2$	5 mM (in DMF)	8 µL	1 mM
PNA-Alkyne	10 mM (in DMF)	10 µL	4 mM
$CuSO_4$	100 mM (in H_2O)	4 µL	10 mM
NaAsc	150 mM (in H_2O)	4 µL	15 mM
THPTA	90 mM (in H_2O)	6.8 µL	15 mM
H_2O	NA	7.2 µL	(55%)
		**40 µL Total Volume**	

Procedure 12 (P12): Cyclisation with diglycolic anhydride

Diglycolic Anhydride (1.5 equiv, 0.003 mmol) and DIPEA (3 equiv, 0.006 mmol) are added to the, previously Fmoc-deprotected, resin (5 mg, 0.002 mmol) in NMP (120 µL). The mixture is agitated for 30 minutes. The resin is washed with DMF and the procedure is repeated. Finally, the resin is washed with 2x DMF, 2x CH2Cl2, 2x DMF. For the cyclisation, HATU (1.5 equiv, 0.003 mmol) and DIPEA (2 equiv, 0.004 mmol) in NMP (120 µL) are added to the resin (5 mg, 0.002 mmol) containing the peptide with

a free amine and carboxylic acid. The mixture is agitated for 1 hour. The resin is washed with 2x DMF, 2x DCM, 2x DMF.

Characterization of PNA-peptide conjugates.

Characterization of the PNA-peptide conjugates was done by MALDI (Bruker Daltonics Autoflex spectrometer with Flex control 3.4 software and analysis with FlexAnalysis 3.4) and/or LC-MS (DIONEX Ultimate 3000 UHPLC with a Thermo LCQ Fleet Mass Spectrometer System using PINNACLE DB C18 column (1.9 µm, 50 x 2.1 mm) with Thermo Xcalibur 2.2.SP1.48 software and analysis with Thermo Xcalibur Qual Browser 2.2.Sp1.48). For MALDI analysis, 1.0 µL of the sample (in either water or water/acetonitrile 1:1) was mixed with 1.0 µL of DHB matrix solution (30 mg of DHB in 1.0 mL of 70:30:0.01 water/acetonitrile/TFA), and the mixture spotted on a MALDI plate. The measurements were done in a positive linear mode. For LC-MS analysis, 20 µL of sample in water or water/acetonitrile 1:1 was injected on the LC and further analyzed by MS on a positive mode.

5. Synthesis of Arg(Pbf)-Benzothiazole-N_3

2-(N2-(tert-butoxycarbonyl)-Nw-((2,2,4,6,7-pentamethyl-2,3-dihydrobenzofuran-5-yl)sulfonyl)-L-arginyl)benzo[d]thiazole-6-carboxylic acid (Boc-Arg(Pbf)-Benzothiazole-COOH)

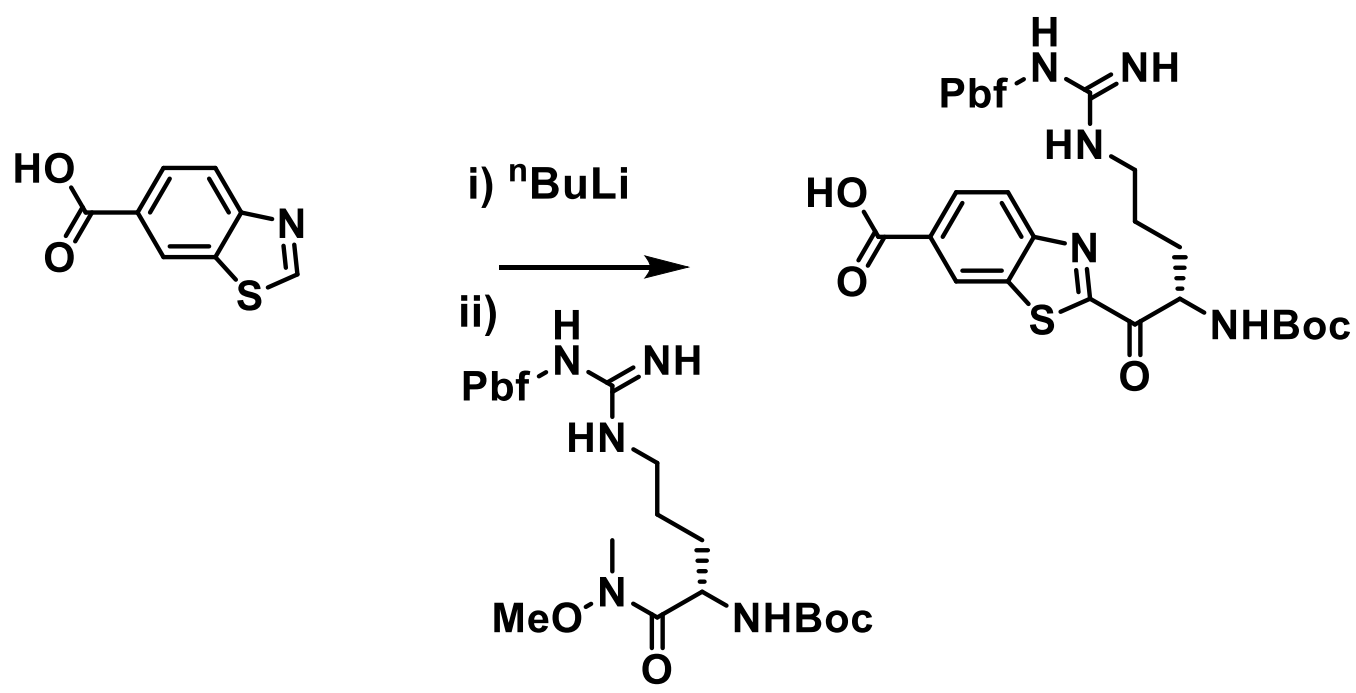

Chemical Formula: $C_{32}H_{41}N_5O_8S_2$, **Exact Mass:** 687.24, **Molecular Weight:** 687.83

benzothiazole-6-carboxylic acid (8.77 mmol, 10 equiv., 1.57g) was added to dry THF (150 mL) under N_2 and cooled to -78 °C. n-BuLi in hexane (19.29 mmol, 22 equiv.) was added dropwise over 10 minutes and stirred for 30 minutes at -78 °C. A solution of Weinreb amide, reported previously,[1] (0.877 mmol, 500 mg) in THF (17.5 mL) was added dropwise at -78 °C and stirred for 30 minutes. The reaction was then stirred at 0°C for 1 hour and 30 minutes. The reaction was quenched with 10 mL of saturated NH_4Cl and warmed to room temperature. The mixture was extracted with EtOAc (3x), the organic layers were washed with H_2O (2x), Brine (1x) and dried over Na_2SO_4 and concentrated. The crude product was purified by flash chromatography (5% MeOH in DCM) to yield Boc-Arg(Pbf)-Benzothiazole-COOH as a colourless film (340 mg, 0.494 mmol, 56%).

LCMS (ESI); RT= 2.81, $[M+1H]^{1+}$: 687.97

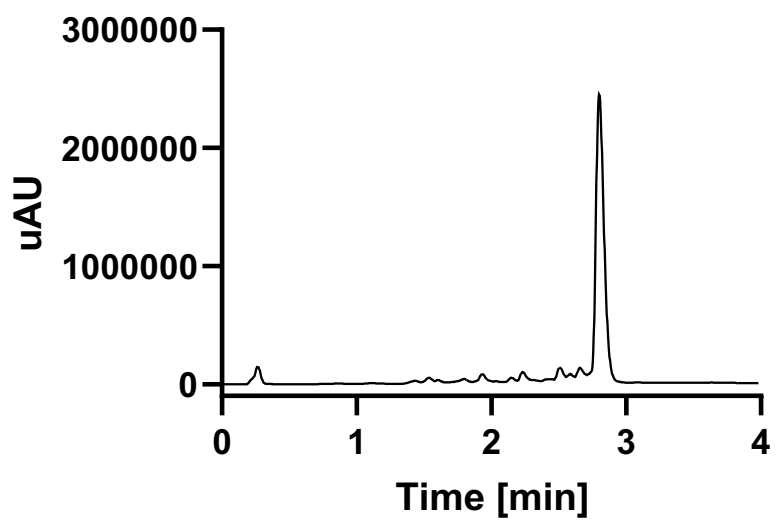

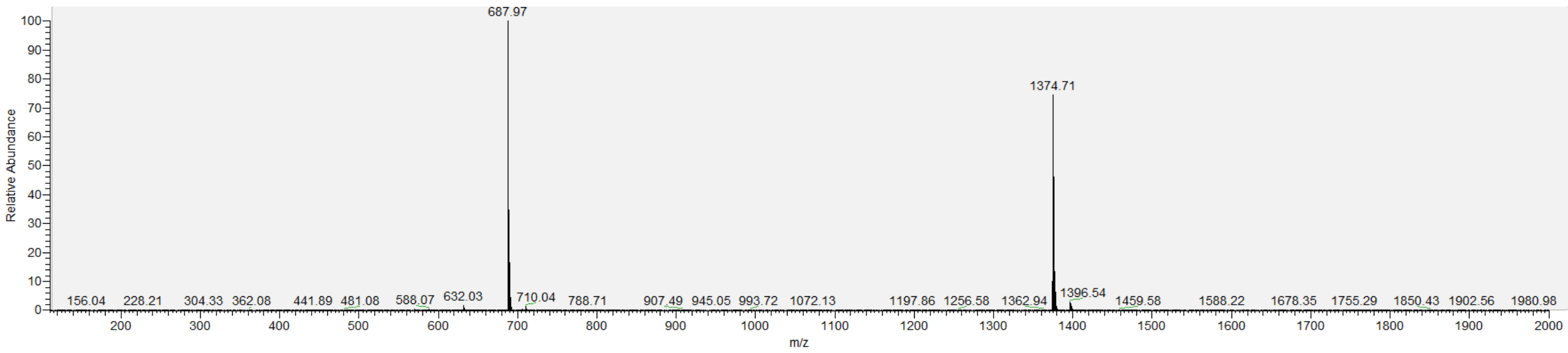

NMR; ^{1}H NMR (400 MHz, DMSO) δ 8.88 (s, 1H), 8.28 (d, *J* = 8.6 Hz, 1H), 8.13 (d, *J* = 8.7, 1.7 Hz, 1H), 7.52 (d, *J* = 7.0 Hz, 1H), 5.17 (t, 1H), 3.04 (d, *J* = 6.5 Hz, 2H), 2.88 (s, 2H), 2.41 (s, 3H), 2.35 (s, 3H), 1.93 (s, 3H), 1.65 – 1.53 (m, 2H), 1.53 – 1.44 (m, 2H), 1.37 (d, *J* = 2.7 Hz, 6H), 1.33 (s, 9H).

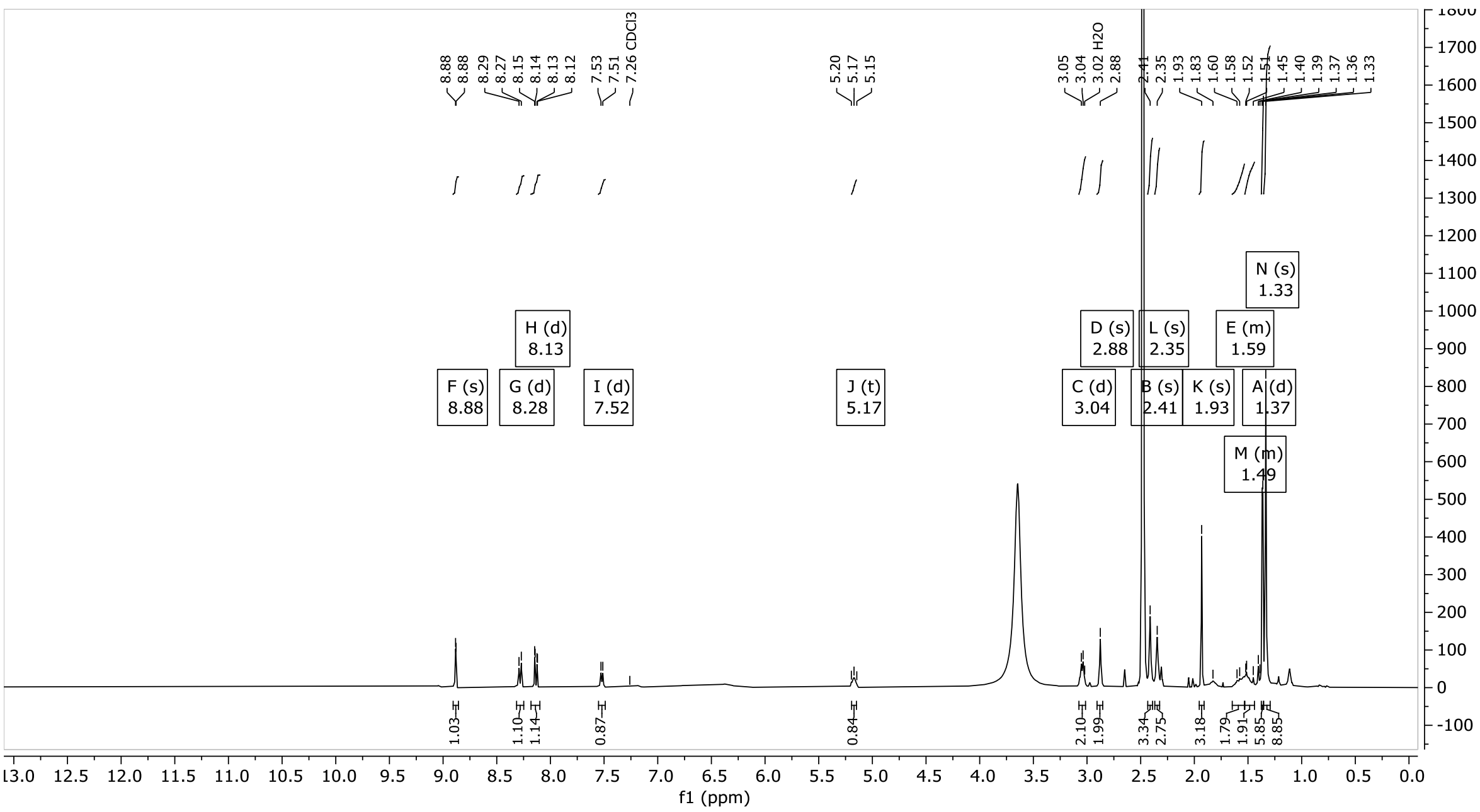

tert-butyl (S)-(1-(6-((2-azidoethyl)carbamoyl)benzo[d]thiazol-2-yl)-1-oxo-5-(3-((2,2,4,6,7-pentamethyl-2,3-dihydrobenzofuran-5-yl)sulfonyl)guanidino)pentan-2-yl)carbamate (Boc-Arg(Pbf)-Benzothiazole-N_3)

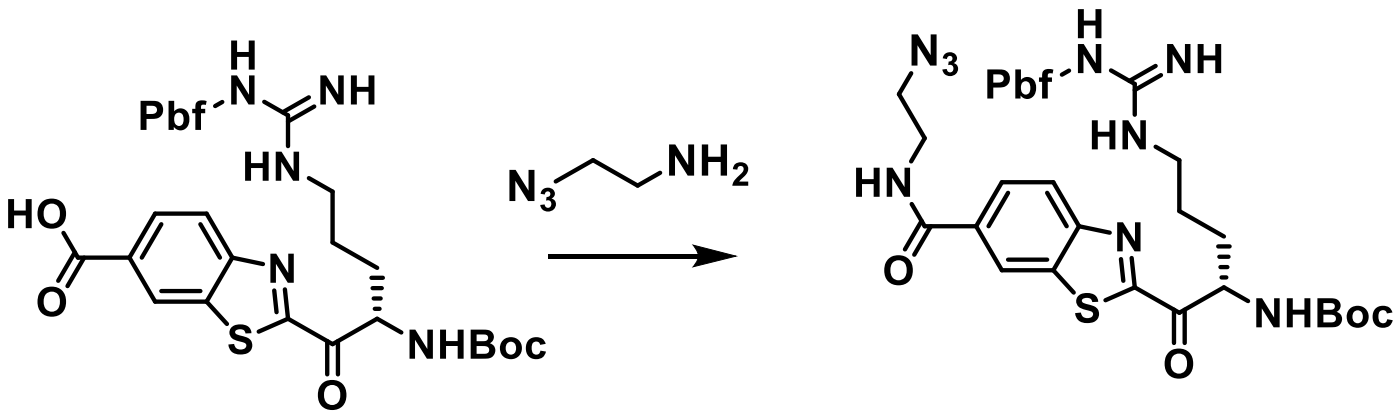

Boc-Arg(Pbf)-Benzothiazole-COOH (280 mg, 0.41 mmol) was dissolved in DMF (7.2 mL) and HATU (1.1 eq., 0.451 mmol, 172 mg) and DIPEA (5 eq., 2.05 mmol, 356 µL) were added. 2-azidoethan-1-amine (1.5 eq., 0.61 mmol, 53 mg) in DMF (1 mL) was added and the reaction was stirred for 1.5 hours at room temperature. Pentane (20 mL) was added and the solution was concentrated under *vacuo*. Water was added and extracted with DCM (3x). The organic layers were washed with brine (2x), dried with $NaSO_4$ and concentrated under *vacuo*. The crude material was purified by flash chromatography (15% pentane in ethyl acetate) to yield Boc-Arg(Pbf)-Benzothiazole-N_3 (175 mg, 56%).

Chemical Formula: $C_{34}H_{45}N_9O_7S_2$, **Exact Mass:** 755.29, **Molecular Weight:** 755.91

LCMS (ESI); RT= 2.88, $[M+1H]^{1+}$: 756.00

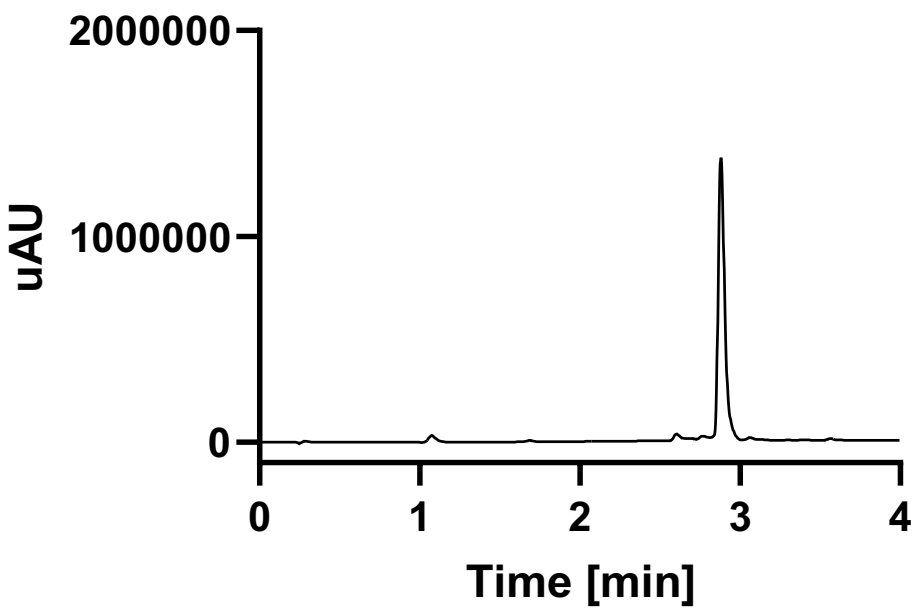

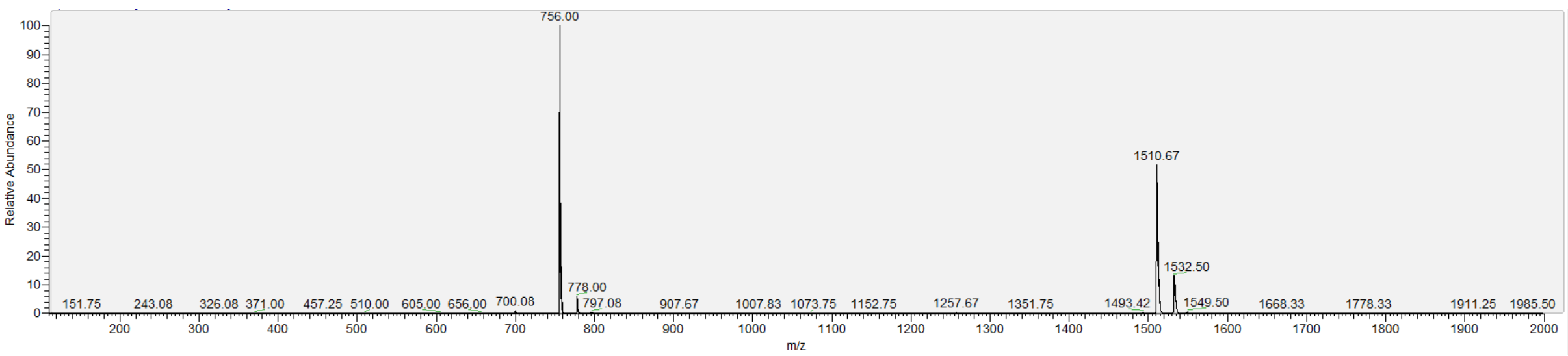

NMR; ^1H NMR (400 MHz, $CDCl_3$) δ 8.45 (s, 1H), 8.19 (dd, *J* = 8.7, 1.8 Hz, 1H), 7.94 (dd, *J* = 8.6, 1.7 Hz, 1H), 5.77 (d, 1H), 5.55 (s, 1H), 3.71 – 3.65 (m, 2H), 3.60 (t, *J* = 5.6 Hz, 2H), 3.35 (s, 1H), 2.95 (s, 2H), 2.80 (s, 3H), 2.56 (s, 3H), 2.50 (s, 3H), 2.08 (s, 3H), 1.83 – 1.72 (m, 2H), 1.47 (s, 6H), 1.44 – 1.42 (m, 4H).

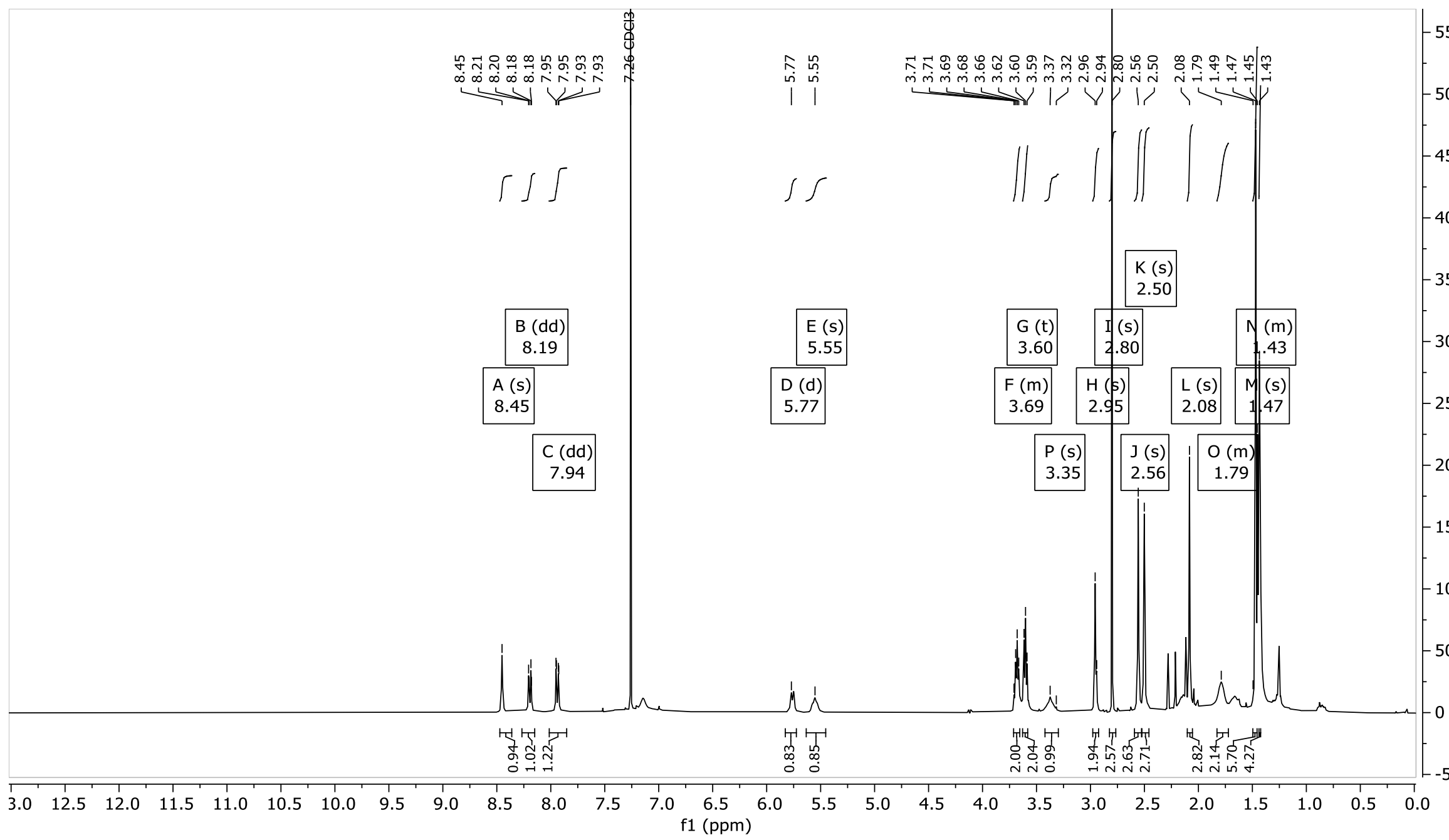

N-(2-azidoethyl)-2-(Nw-((2,2,4,6,7-pentamethyl-2,3-dihydrobenzofuran-5-yl)sulfonyl)-L-arginyl)benzo[d]thiazole-6-carboxamide (Arg(Pbf)-Benzothiazole-N$_3$)

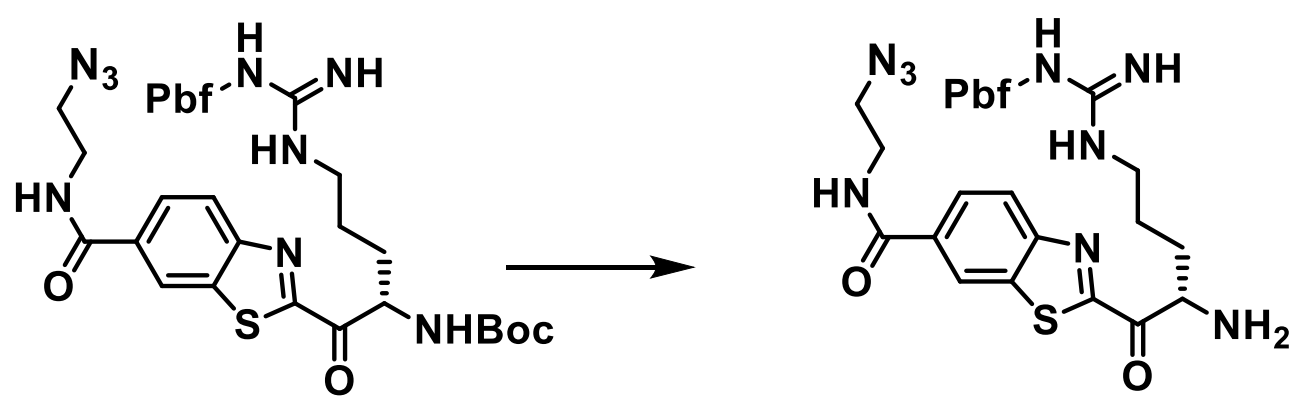

Chemical Formula: Chemical Formula: $C_{29}H_{37}N_9O_5S_2$, **Exact Mass**: 655.24, **Molecular Weight**: 655.79.

Boc-Arg(Pbf)-Benzothiazole-N$_3$ (100 mg, 0.132 mmol) was dissolved in dioxane (1.85 mL) and cooled to 0 °C. 4M HCl in dioxane (1.122) was added and the mixture was stirred for 2 hours at room temperature. The mixture was concentrated under *vacuo* to yield Arg(Pbf)-Benzothiazole-N$_3$ as a brown solid (86 mg, quant.). The compound was used without further purification.

LCMS (ESI); RT= 2.06, $[M+1H]^{1+}$: 656.08

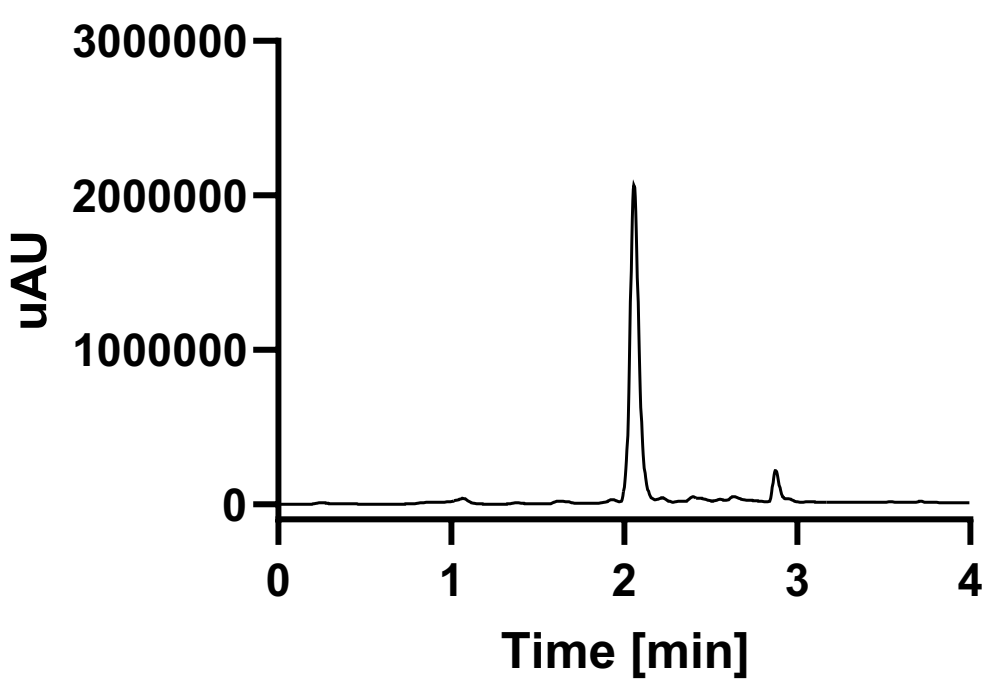

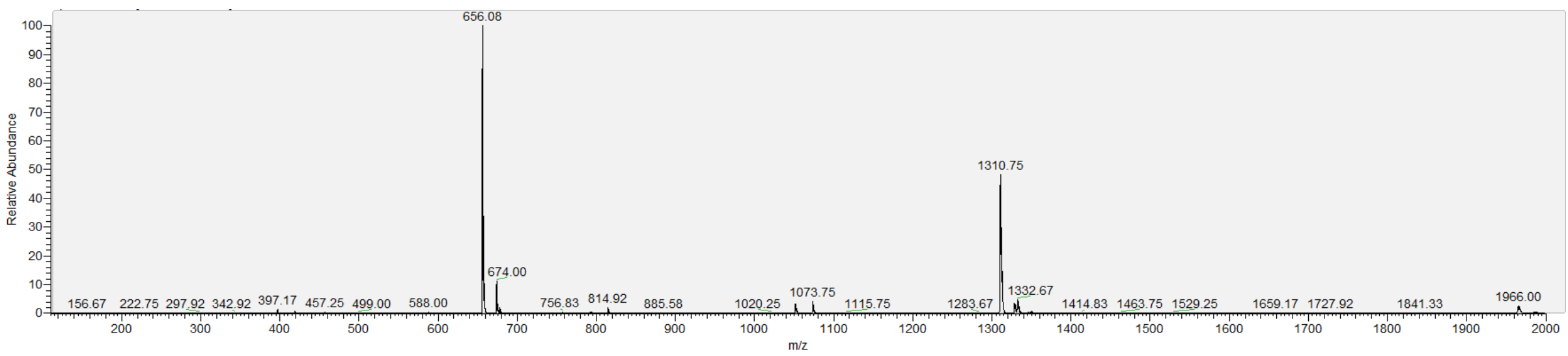

6. Selection Procedure

Library Preparation: Each crude library member was dissolved in DMSO (15 µL). 1 µL of the stock was then diluted 400-fold with water and the concentration was quantified by nanodrop (at 260 nm). The individual compounds were combined into active site, exosite I and exosite II libraries (5 µM/compound, total concentration 250 µM). The three libraries were then combined to obtain the final library (with 1 µM/compound, total concentration: 50 µM of each fragment and 150 µM of members).

Selection: 7.5 µL of library (1 µM/per compounds – final concentration 500 nM) was incubated with 7.5 µL of Thrombin (1 µM– final concentration 500 nM) (‘*Thrombin Selection*’) or Buffer (PBS) (‘*No Thrombin Selection*’) for 5 minutes. 1 µL of ‘*No Thrombin Selection*’ was spotted on MALDI for Pre-Selection library. 400 µL PBS was added to each selection and incubated at room temperature for 15 minutes. Each solution was transferred to an Amicon® Ultra Centrifugal Filter (30 kDa MWCO, UFC8030) and centrifuged for 15 minutes at 18 400 *g*. 1 µL of the selections were spotted on MALDI for analysis.

Each selection was performed in duplicate on different days – see duplicates below.

MALDI: 1 µL of sample + 1 µL DHB Matrix. Once dried another layer of DHB (1 µL) was added. The MALDI was calibrated using standard methods on the FlexControl Software using 5 compounds from the library which range from 2500 to 5000 g/mol.

Analysis: The MALDI spectrum were exported to Excel for automated peak picking. To do so, the m/z values were added to column A and the corresponding intensities for (‘*Library*’, ‘*Thrombin Selection*’ and ‘No *Thrombin Selection*’) were added to column B. To compensate for a small variation in m/z values due to calibration, the following formula (to find the peak within +/- 0.5 g/mol) was used to output the intensity corresponding to each member:

=INDEX(B:B, MATCH(1, (A:A >= I2 - 0.5) * (A:A <= I2 + 0.5), 0))

In order to remove the background from size exclusion spin filters, the enrichment was calculated as:

$$E_i = E_{i,s} - E_{i,c} = \frac{I_{i,s}}{I_{i,l}} - \frac{I_{i,c}}{I_{i,l}}$$

With E_i: Enrichment of compound i; $E_{i,s}$: Enrichment of compound i in selection; $E_{i,c}$: Enrichment of compound i in control; $I_{i,l}$: Intensity of compound I in library; $I_{i,s}$: Intensity of compound i in selection; $I_{i,c}$: Intensity of compound i in control

7. 10x10x5 Pilot Library

A 10x10x5 pilot library was prepared and selected as per the experimental procedure in section 4.2. The MALDI spectrum were normalised to the highest peak and peak intensities were compared between library and selection. This data shows enrichment of positive controls and no change or decreased intensity for negative controls. This data validates the selection procedure.

8. 50x50x50 Duplicate Spectrums

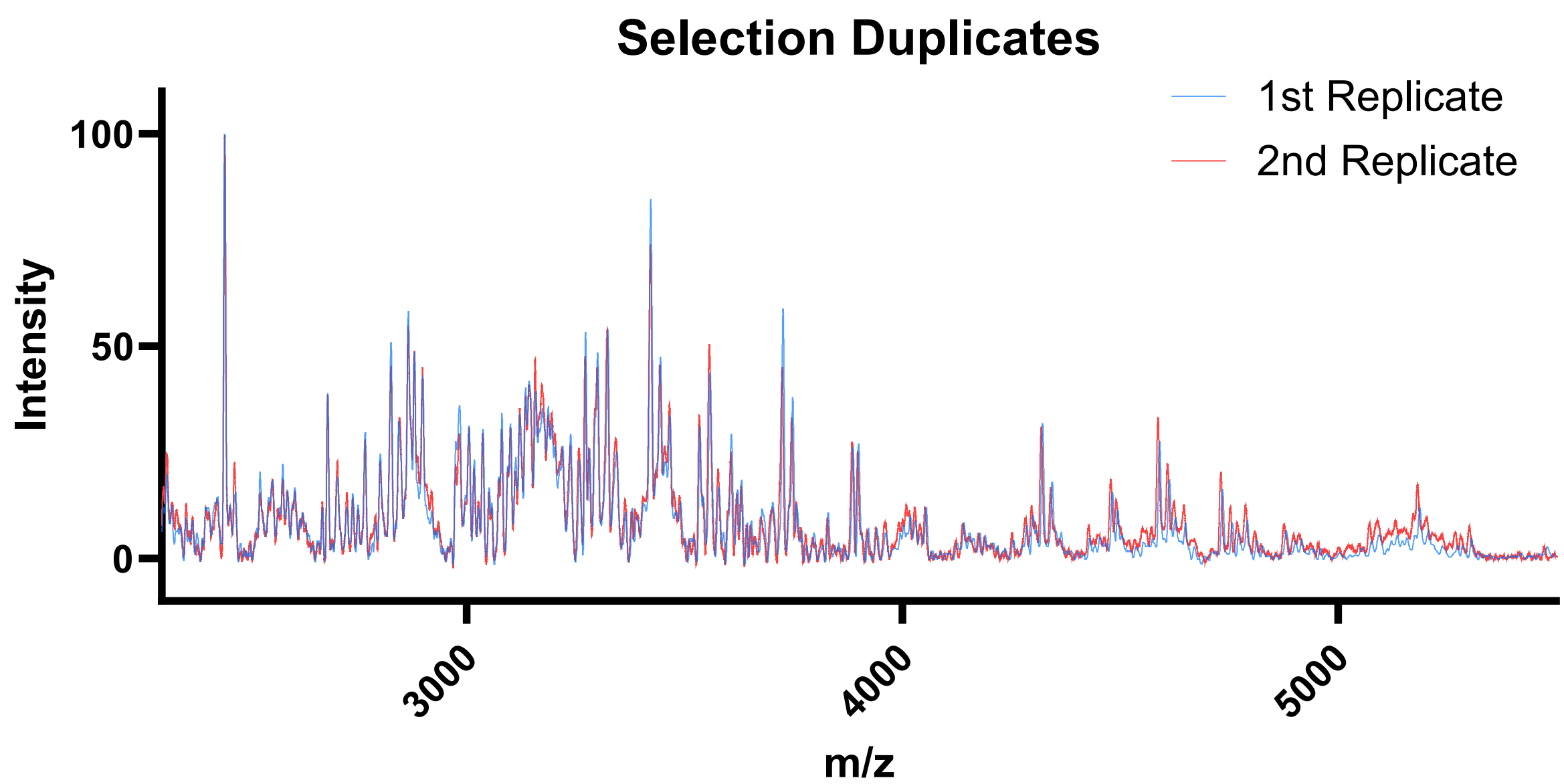

SI Figure S6*: Raw MALDI spectrum of 50x50x50 selection. Both replicates (2 independent selection experiments) are shown.*

10x10x10 Duplicate Spectrums

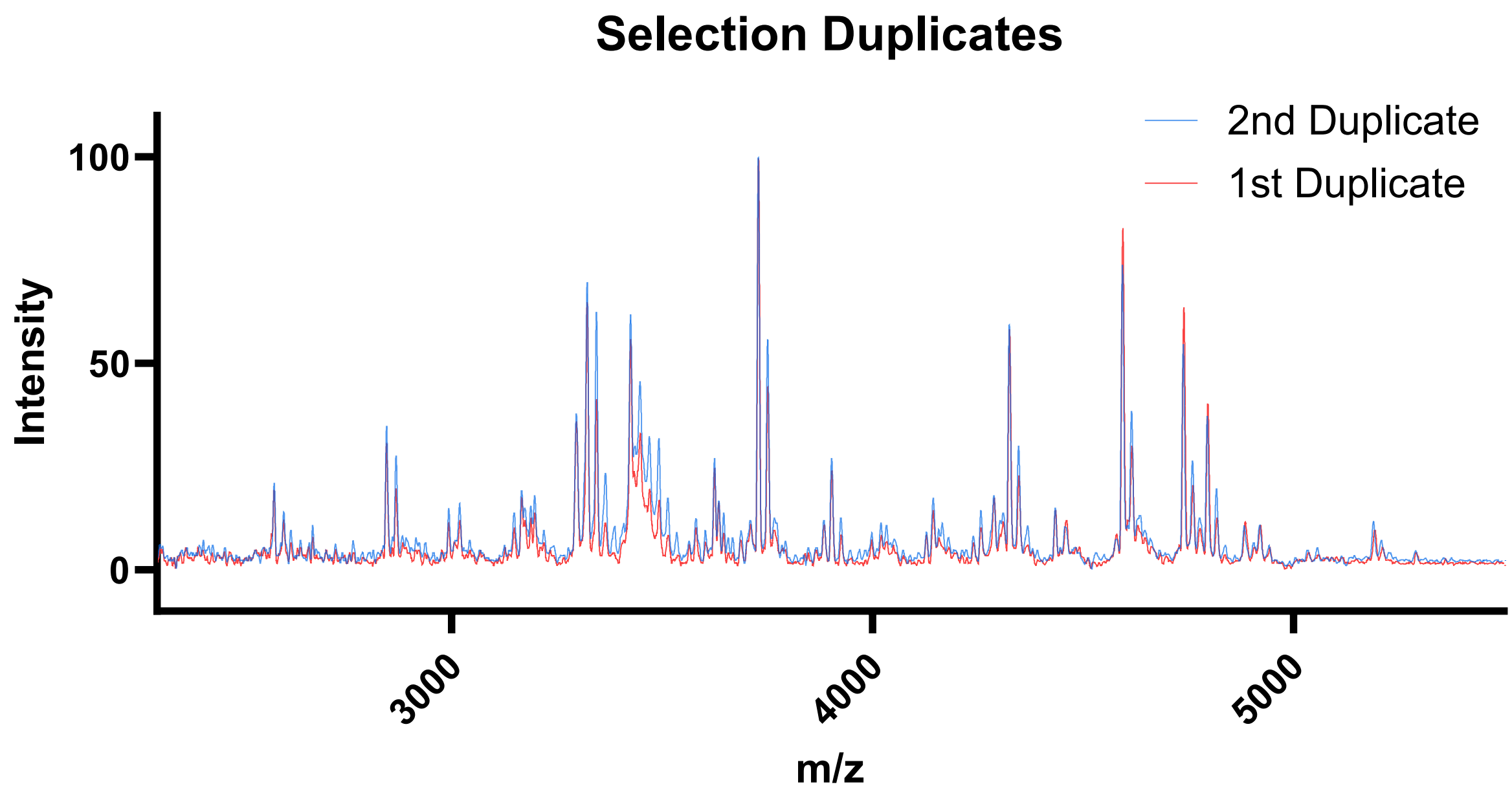

SI Figure S7*: Raw MALDI spectrum of 10x10x10 selection. Both replicates (2 independent selection experiments) are shown.*

9. Thrombin Inhibition Assay

In vitro Inhibition of Human α-Thrombin. The inhibition of the activity of human α-thrombin (Haematologic Technologies, HCT0020) was followed spectrophotometrically using phe-Pro-Arg-Coumarin (synthesised as previously reported[1]) as chromogenic substrate.

Inhibition assays were performed using 0.2 nM enzyme, 20 µM substrate, and increasing concentrations of inhibitor. The concentration of each inhibitor variant was determined using the absorbance of the PNA at 260 nm, measured by nanodrop. All reactions were carried out at 37 °C in 50 mM Tris-HCl pH 8.0, 50 mM NaCl, 1 mg/mL BSA in black 96-well microtiter plates (ref. 267342, ThermoFisher). Reaction progress was monitored by excitation at 339 nm and emission at 439 nm using a Tecan Spark Plate Reader.

In the assays in Figure 4, 100 µM substrate is used instead of 20 µM as the IC50s are very low. Number of flashes and gain of the plate are subsequently adjusted.

In the case of reversal (Figure 4B), 5 µL of antidote or buffer is added after 15 minutes and the fluorescence monitoring is resumed.

For each inhibitor, the reactions were performed in duplicate, together with control reactions in the absence of enzyme. The initial velocity was calculated from the slope of the first 10 minutes of the assay. The curves were normalised to the well without inhibitor, where the initial velocity was set to

100% activity. The IC50 curves (Figure 4) were fitted using GraphPad 10.2.0 ‘Log(Inhibitor) vs. response’ curves and the Ki values were determined with ‘Morrison K_i’ model for tight binding.

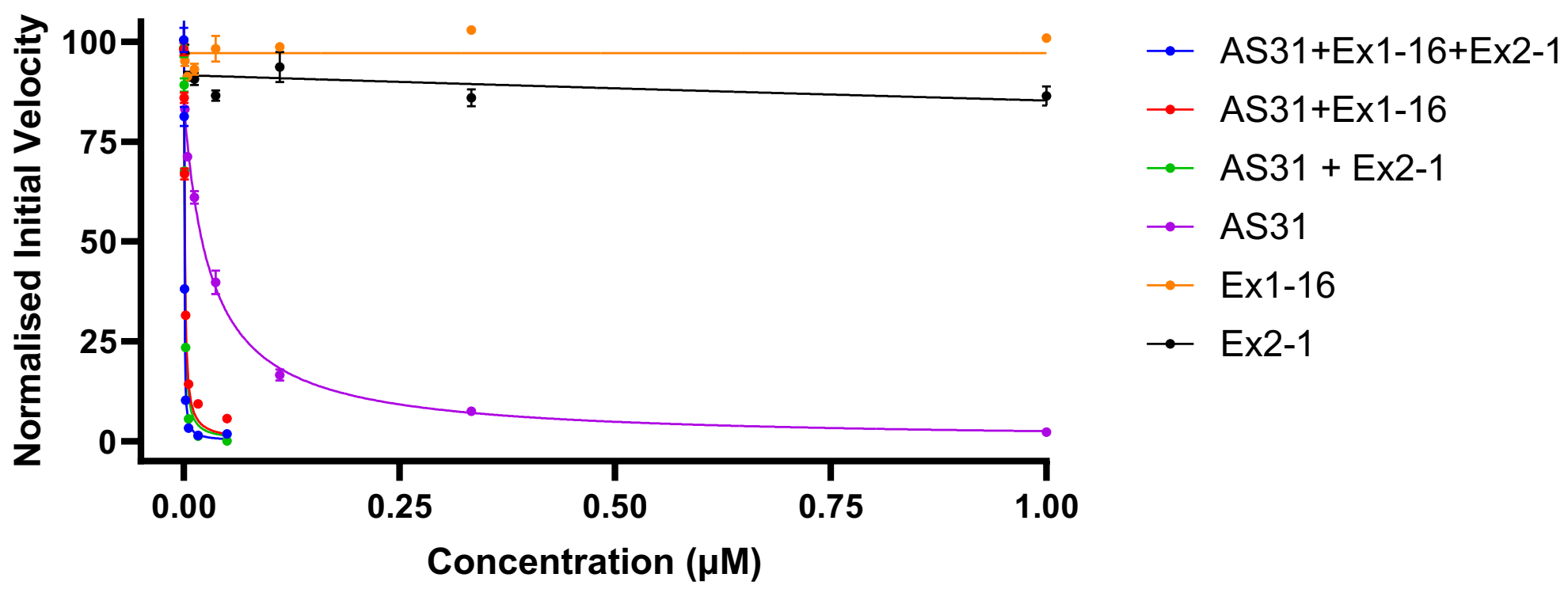

SI Figure S8: *Concentration-response plot for a tight binder – used to determine K_i values.*

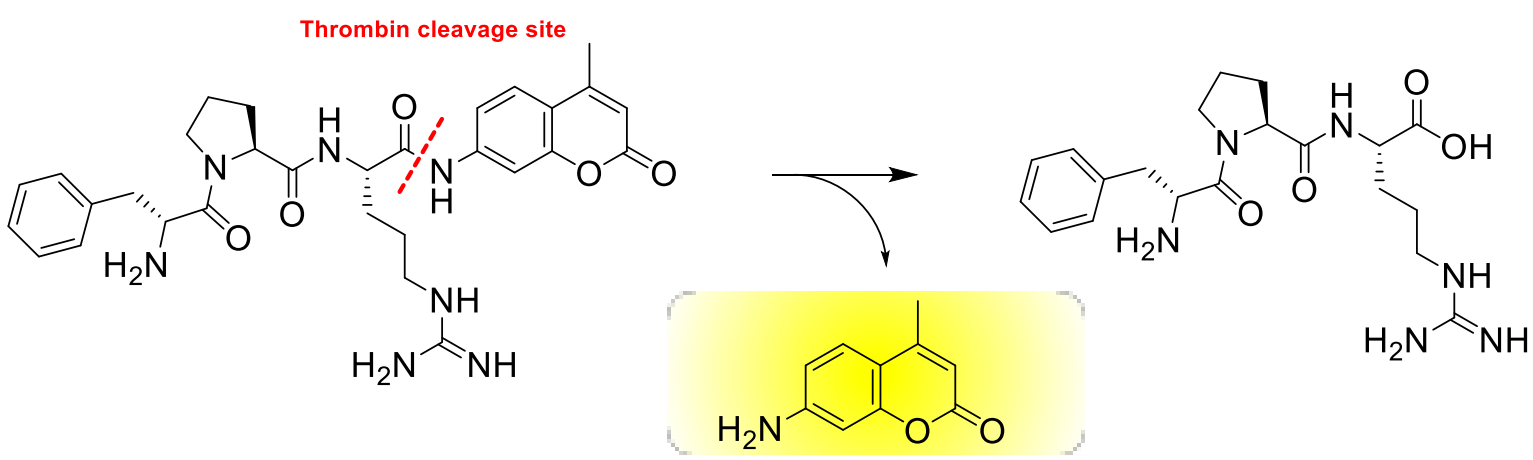

SI Figure S9: *Thrombin Fluorogenic Assay*

To convert IC50 values to K_i values, K_M was determined: The assay described above was performed without inhibitor but with variation of [substrate]. The Michaelis Menten plot was obtained, and K_M and Vmax were determined to be 2.041 µM and 620.8 respectively (R^2: 0.98).

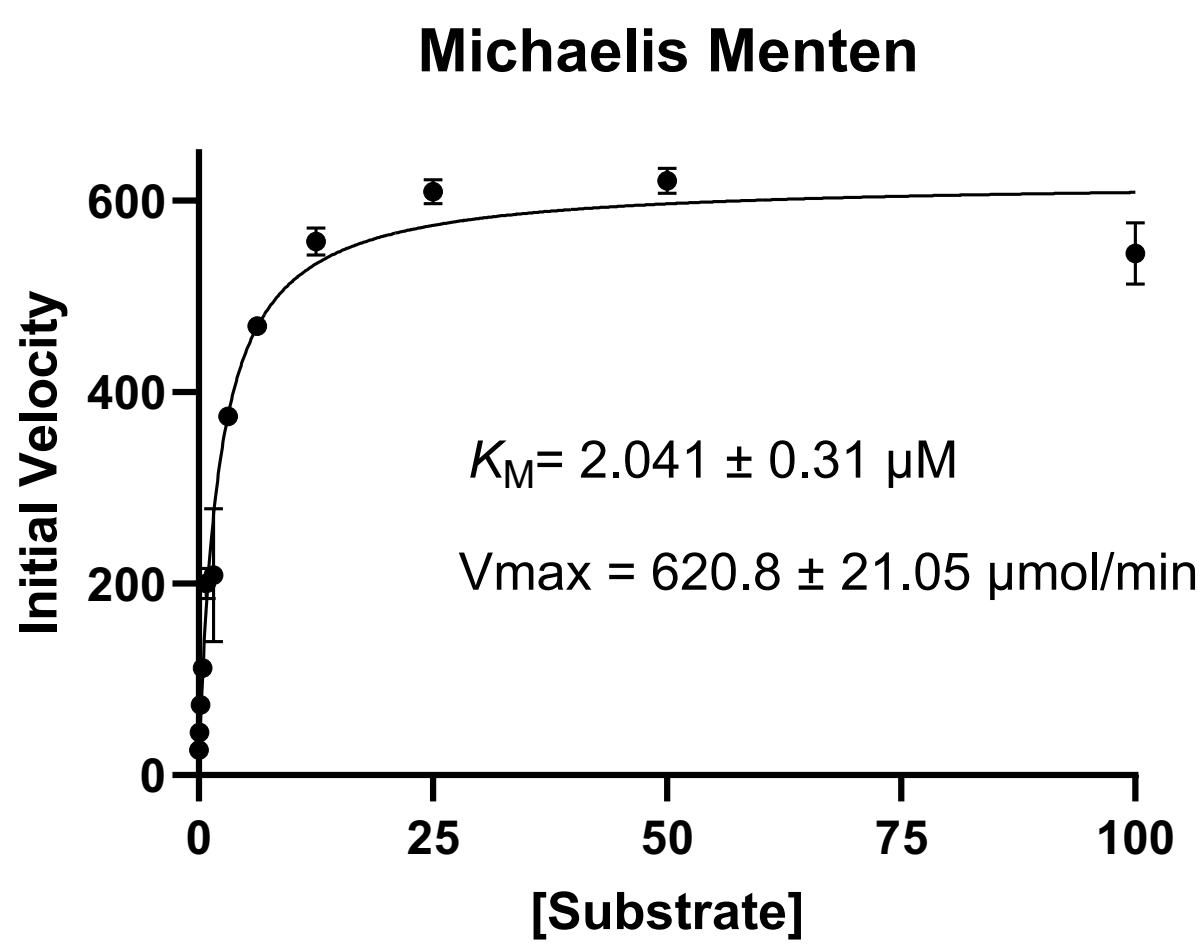

SI Figure S10: *Michaelis Menten Plot*

10. SPR Experiments

SPR experiments were performed on a Biacore T200 instrument (GE Healthcare) at 25 °C in PBS-P+ buffer (10x stock from Cytiva Life Sciences, 28995084). Thrombin was used in solution to avoid inactivating or perturbing the active site. The ligands were immobilized either via biotin capture or PNA hybridisation (to biotinylated PNA that was previously captured) – see Table S3 for details for each measurement. The PNA hybridisation method enables capture of a range of PNA-compounds whilst biotin-capture requires one chip per compound.

Immobilisation - Biotin-loading on the chip

Streptavidin was chemically immobilized via NHS/EDC coupling on a CM5 sensor chip (Cytiva Life Sciences, 29104988). Immobilization was performed following the standard Cytiva protocol (amine coupling of ligands to Biacore sensor chips) using streptavidin at 20 µg/mL, contact time of 600 s and flow rate of 10 µL/min. Subsequently, Ligand-Biotin was independently immobilized on the previous streptavidin coated chip. Prior to immobilization, the two channels were conditioned with 1 M NaCl in 50 mM NaOH. After stabilization, the biotinylated compound was flowed over one of the flow cells of the sensor chip at a concentration of 50 nM in PBS-P+ at a flow rate of 10 µL/min with a targeted response level of 150 RUs. The system (not including the flow cells) was washed with 50% isopropanol in 1 M NaCl and 50 mM NaOH after each ligand injection.

Capture - PNA-hybridisation on the chip

Some of the compounds were captured on to the chip via hybridisation, which consisted of ligand capture of PNA-compound (X nM, X s injection, flow rate X µL/min, stabilisation X s) at the beginning of each cycle, prior to kinetic measurements.

Kinetic measurements

Kinetic measurements consisted of injections (association, dissociation, flow rate: 30 µL/min) of decreasing concentrations of thrombin (2-fold cascade dilutions from the starting concentration). The chip was regenerated between cycles by one injection of regeneration solution (50 mM NaOH) for 10 seconds at a flow rate of 20 µL/min, followed by a 10 second stabilization period. Binding was measured as resonance units over time after blank subtraction, and the data interpreted using the Biacore T200 software, version 3.2. The KD values were calculated based on steady-state affinity (1:1 binding).

Supplementary Table S3*: SPR workflow for each compound*

Measurement	Compound used	Immobilisation method	Ligand-Biotin on chip	Association, dissociation times	Highest concentration tested (thrombin)
**AS31 Fragment**	AS31-8mer1	PNA Hybridisation	PNA-Biotin(Exosite sequence)	200, 450s	50 nM
**Ex1-16 Fragment**	Ex1-16-Biotin	Biotin Capture	Ex1-16-Biotin	200, 450s	50 nM
**Ex2-1 Fragment**	Ex2-1-8mer	PNA Hybridisation	PNA-Biotin(Active site sequence)	200, 450s	50 nM
**Trivalent Assembly (AS31+Ex1-16+Ex2-1)**	Ex1-16-Biotin, Ex2-1, AS31	PNA Hybridisation	Ex1-16-Biotin	1000, 2000s	62.5 pM
**Ultravariegin**	Ultravariegin-Biotin	Biotin Capture	Ultravariegin-Biotin	1000, 2000s	62.5 pM

11. aPTT Experiments

500 µL of Pooled Normal Human plasma (Precision Biologic, ref. CCN-10 (80x1ml)) were spiked with the compound(s) (100X stock) and analyzed using Siemens CN-3000. In the case of the antidote, the active inhibitor fragments were added to the plasma followed by antidote and the analysis was performed after 30 minutes.

Dade® Actin® FSL Activated PTT Reagent (ref. B4219-2) and $CaCl_2$ (ref. ORHO37) were acquired from Siemens.

12. Library Members and characterisation

Supplementary Table S4*: Exosite I Library Members*

	Chemical Formula	MW	m/z	Structure	MALDI
**Ex1-1**	$C_{132}H_{182}N_{48}O_{46}$	3177.198	3176.341	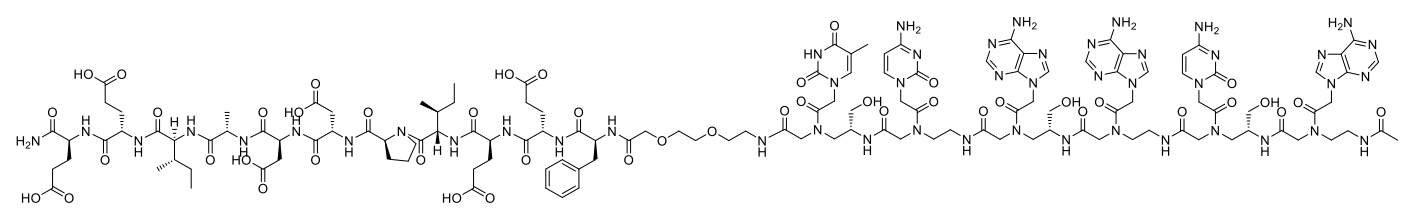	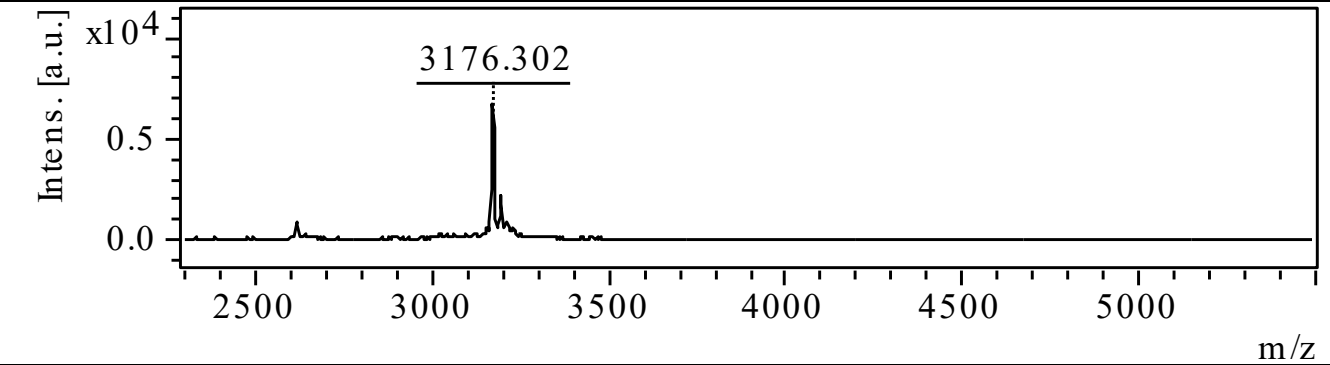
**Ex1-2**	$C_{138}H_{193}N_{49}O_{49}$	3322.356	3321.415	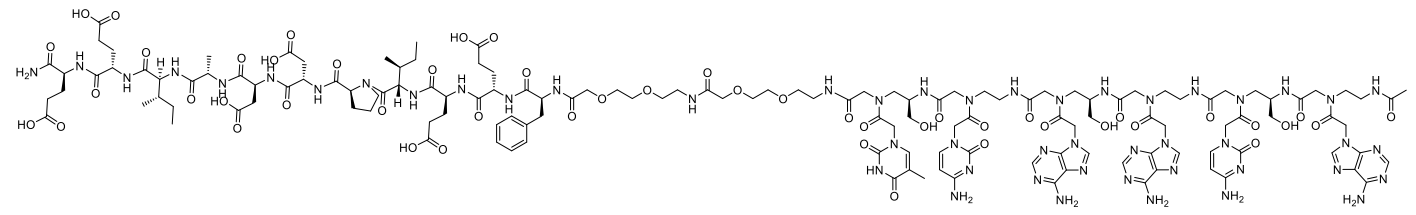	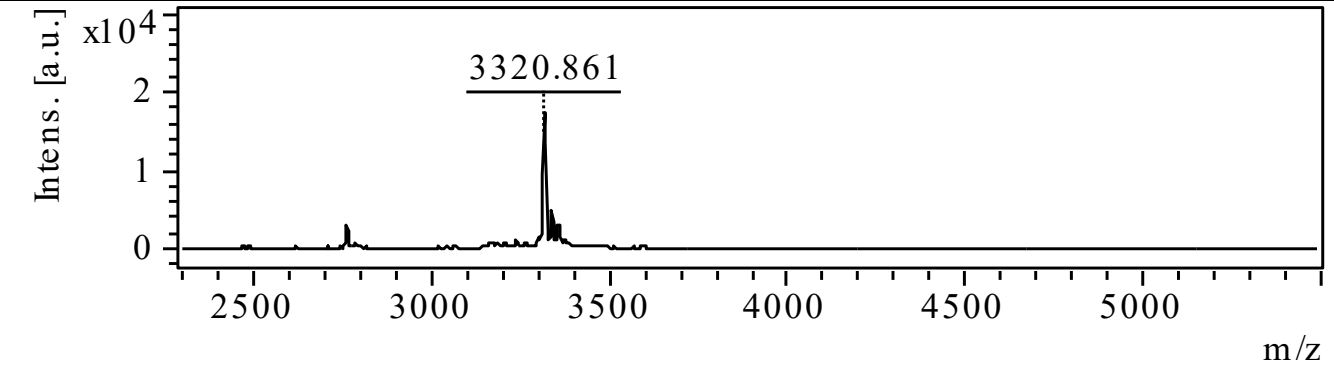
**Ex1-3**	$C_{132}H_{175}N_{51}O_{42}$	3148.167	3147.316	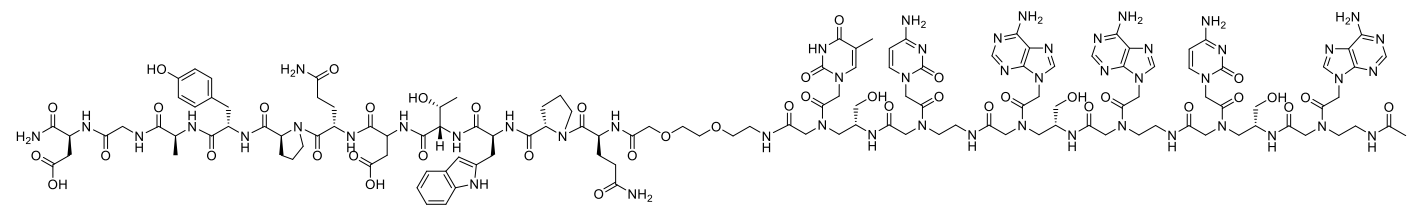	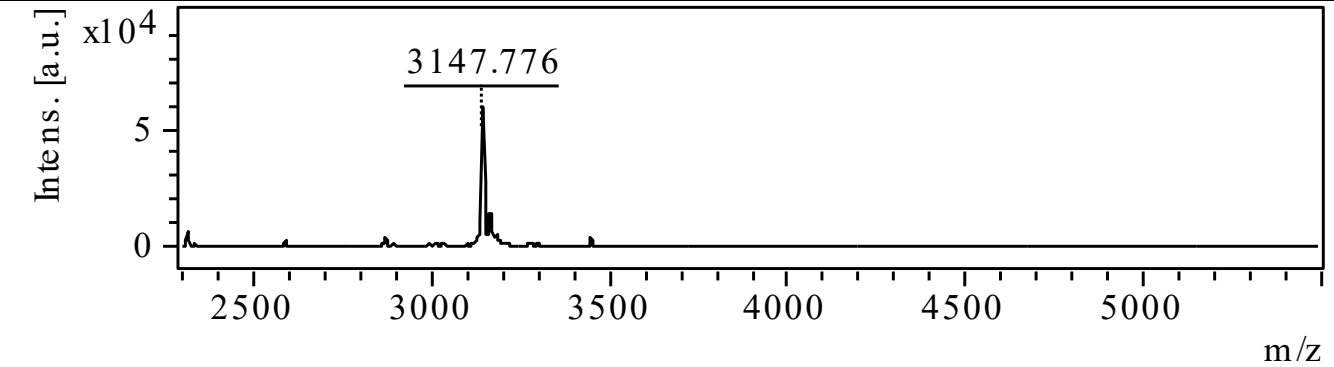
**Ex1-4**	$C_{135}H_{184}N_{52}O_{35}$	3095.286	3094.425	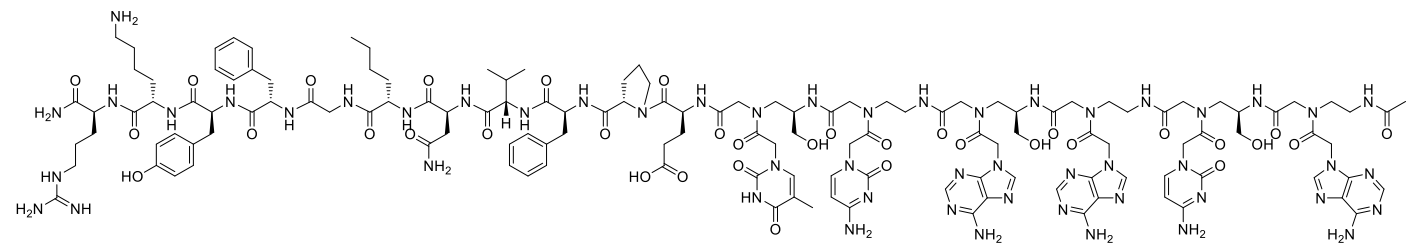	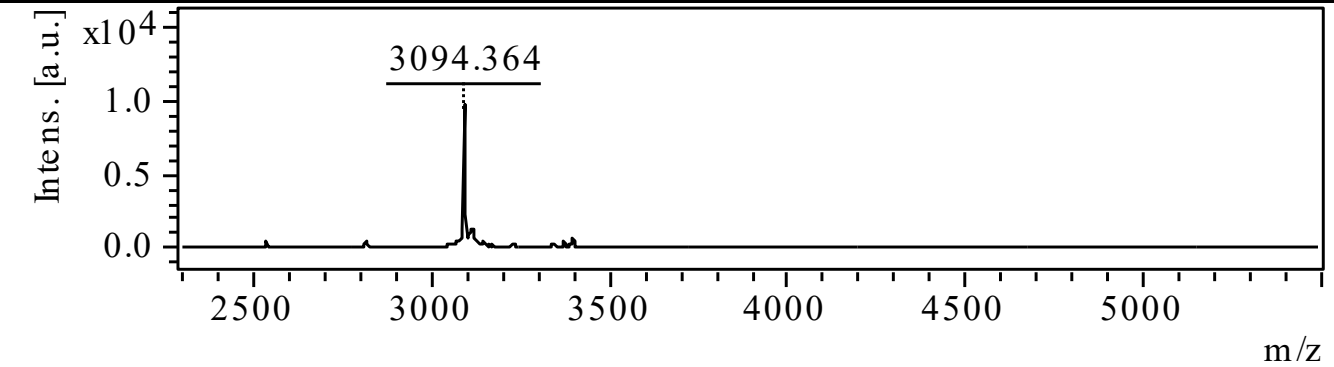
**Ex1-5**	$C_{141}H_{195}N_{53}O_{38}$	3240.444	3239.499	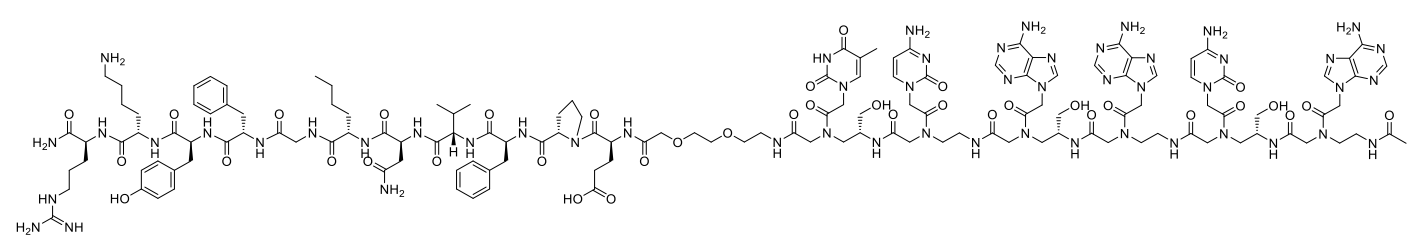	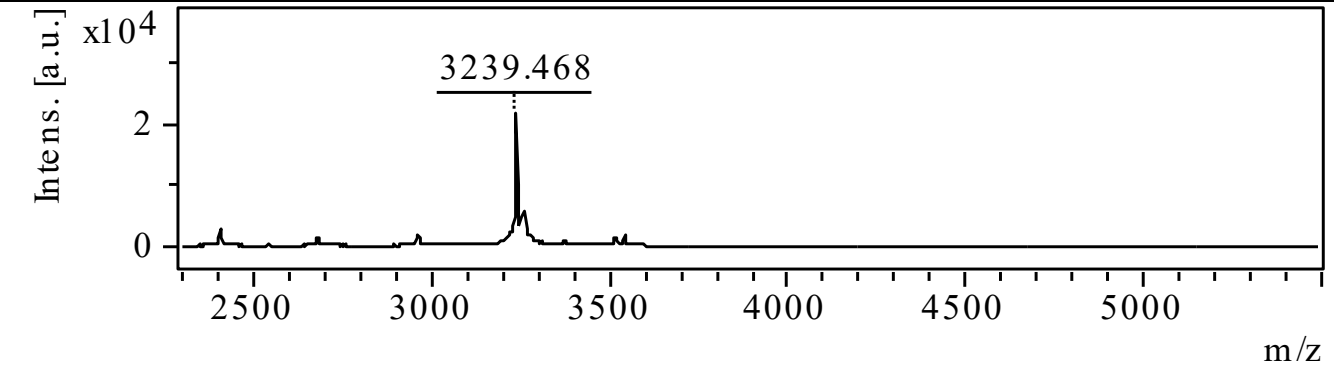

**Ex1-6**	$C_{142}H_{200}N_{56}O_{34}$	3235.52	3234.568	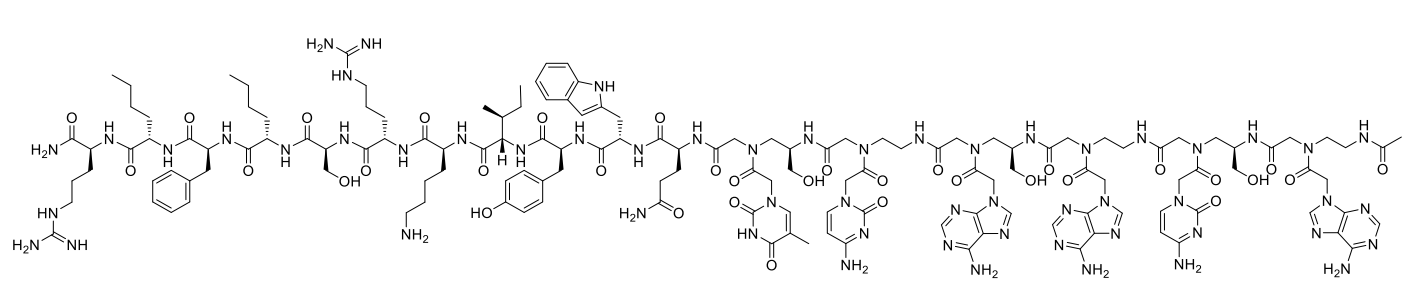	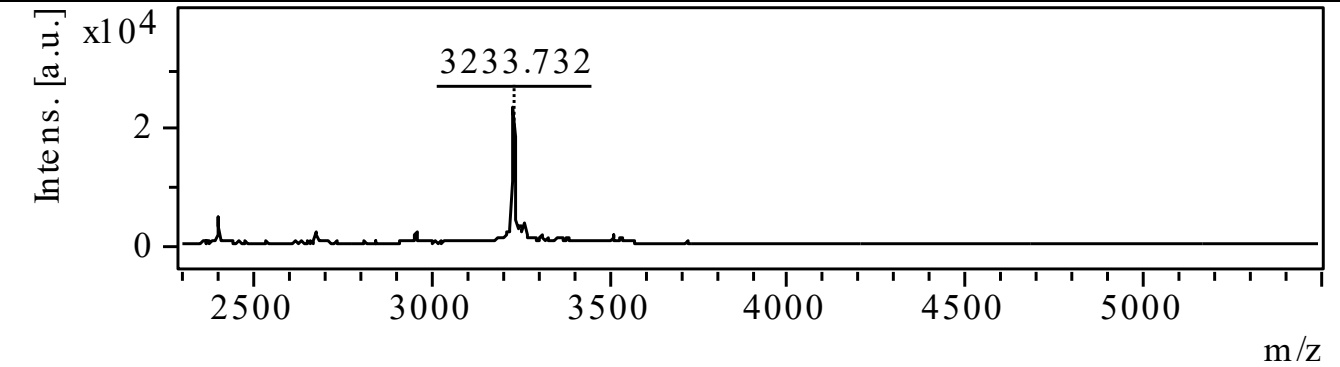
**Ex1-7**	$C_{140}H_{205}N_{53}O_{42}$	3302.509	3301.557	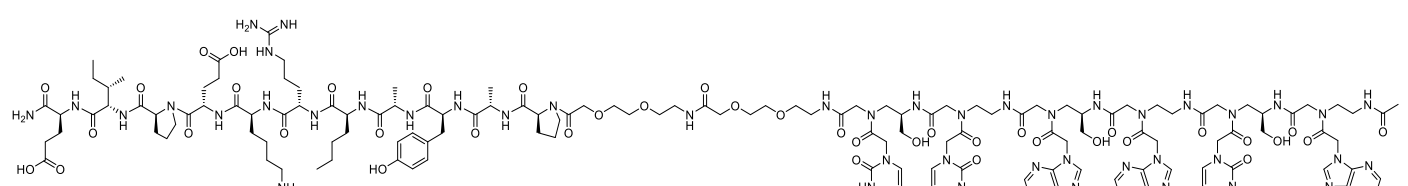	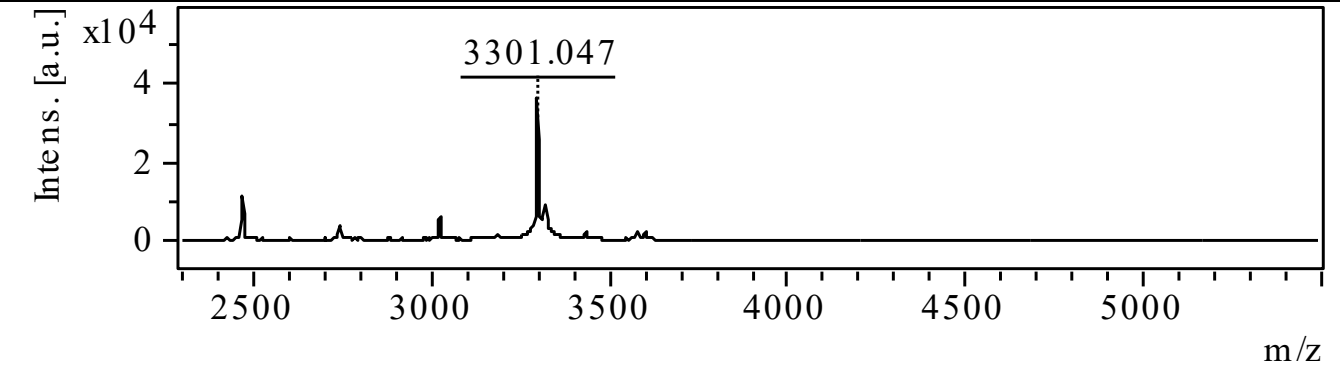
**Ex1-8**	$C_{134}H_{186}N_{54}O_{38}$	3161.302	3160.432	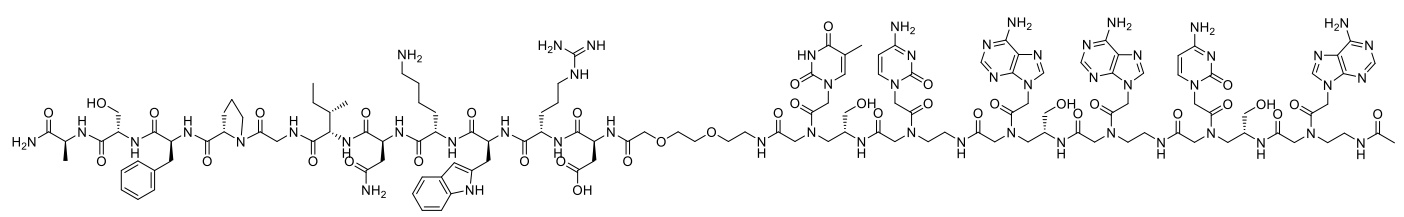	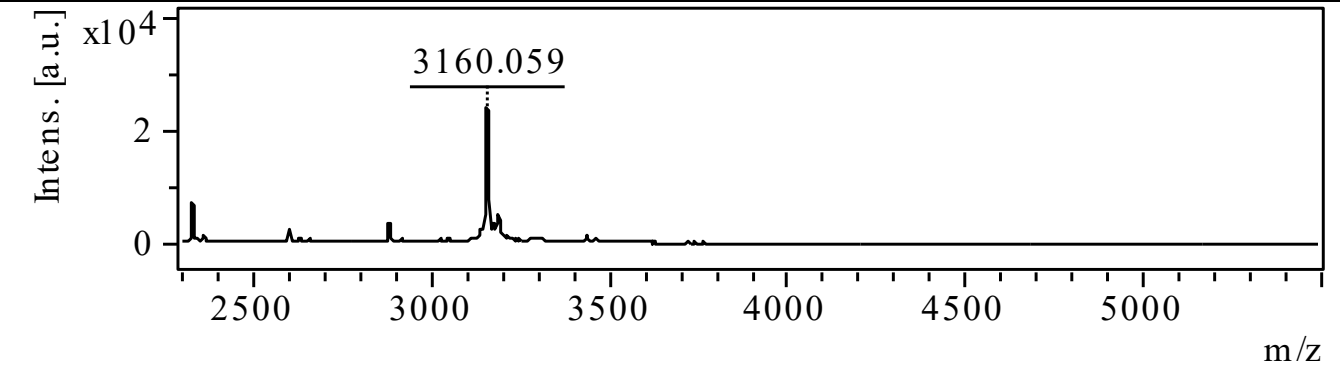
**Ex1-9**	$C_{136}H_{197}N_{55}O_{42}$	3274.415	3273.500	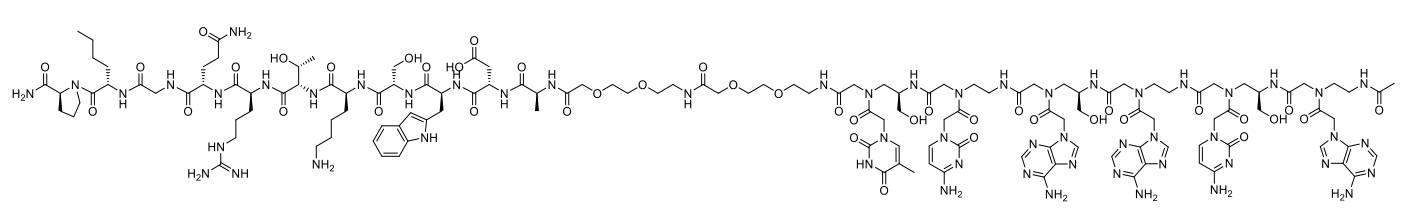	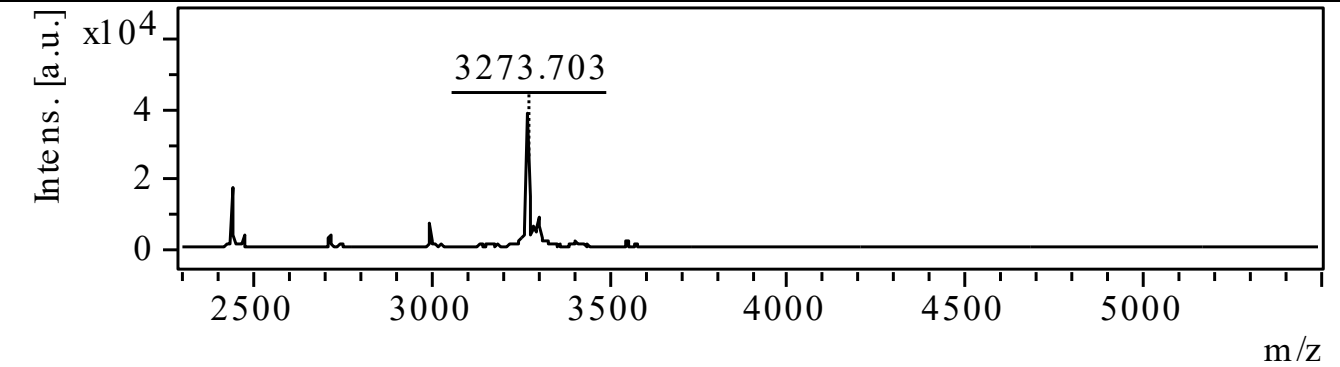
**Ex1-10**	$C_{137}H_{187}N_{53}O_{35}$	3136.339	3135.452	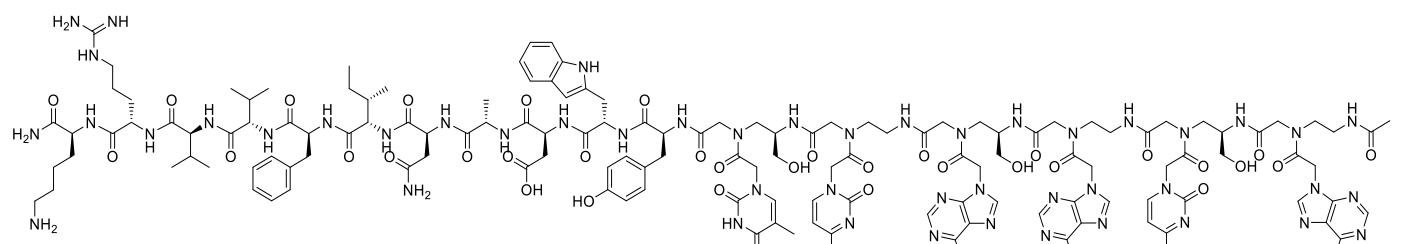	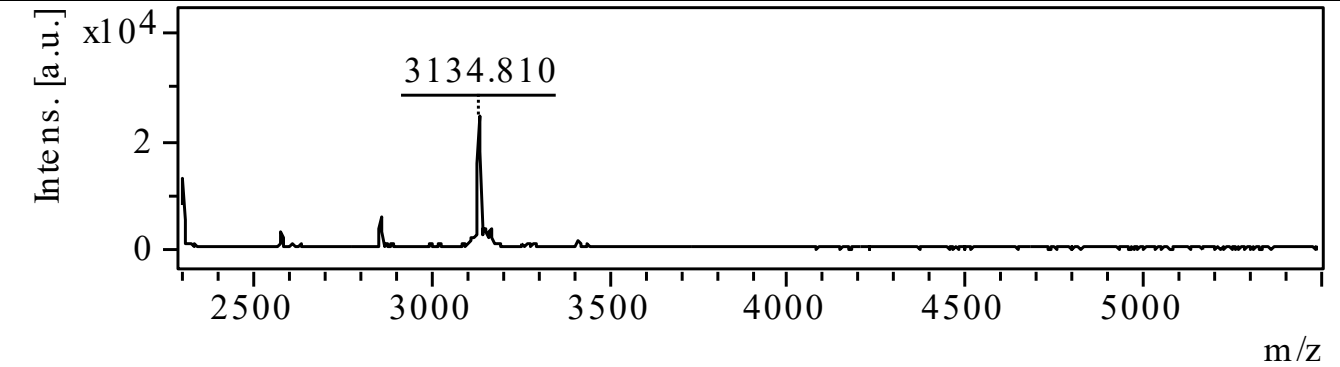
**Ex1-11**	$C_{151}H_{207}F_2N_{51}O_{55}S$	3686.676	3685.469	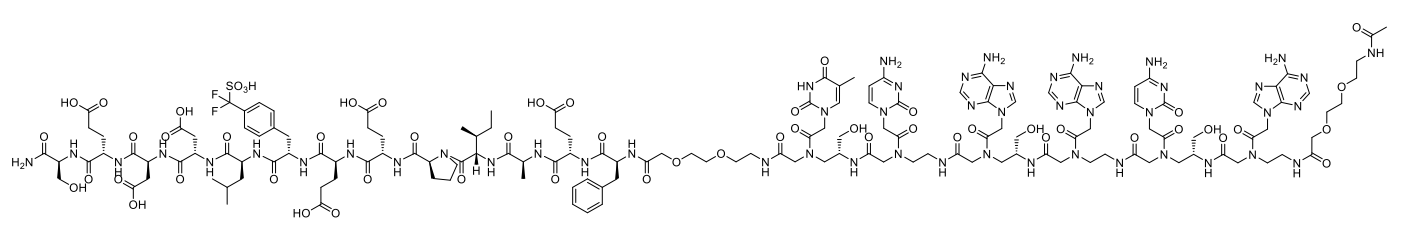	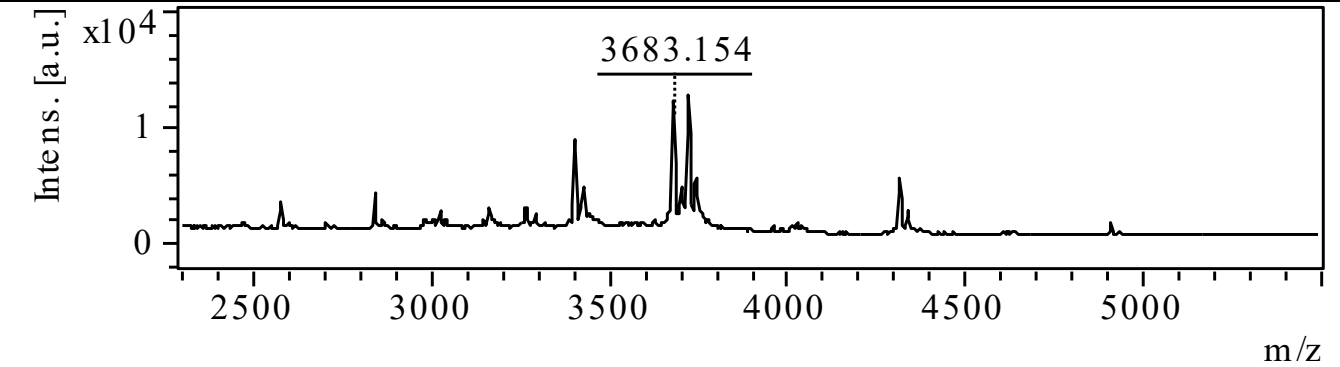

**Ex1-12**	$C_{138}H_{197}N_{49}O_{47}$	3294.39	3293.456	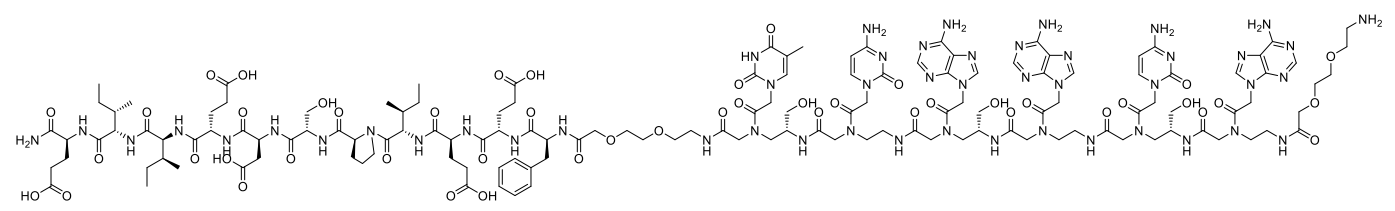	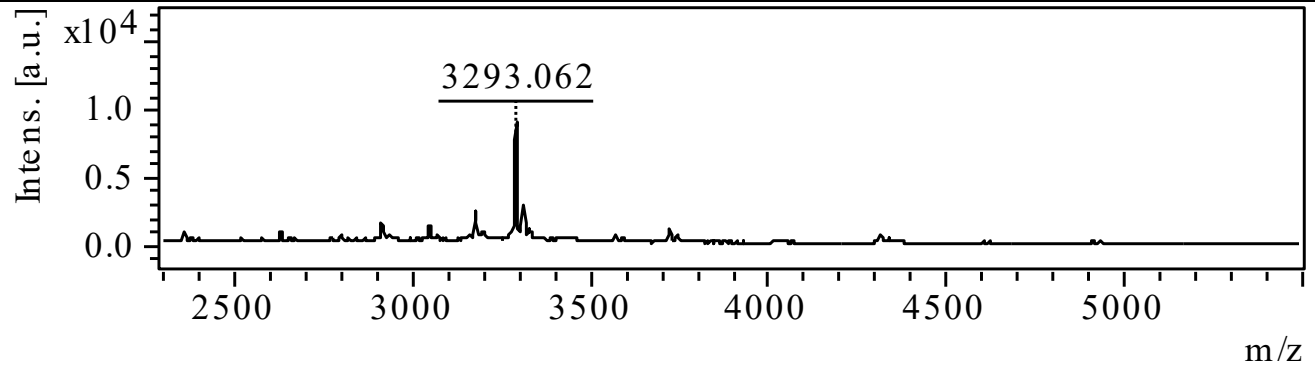
**Ex1-13**	$C_{137}H_{187}F_2N_{47}O_{46}S$	3298.343	3297.346	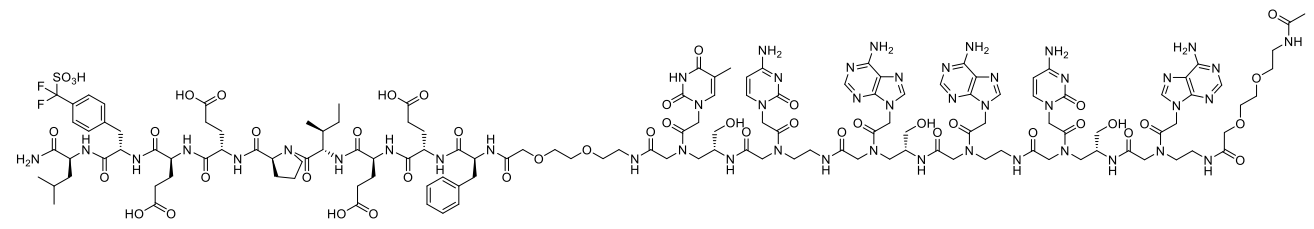	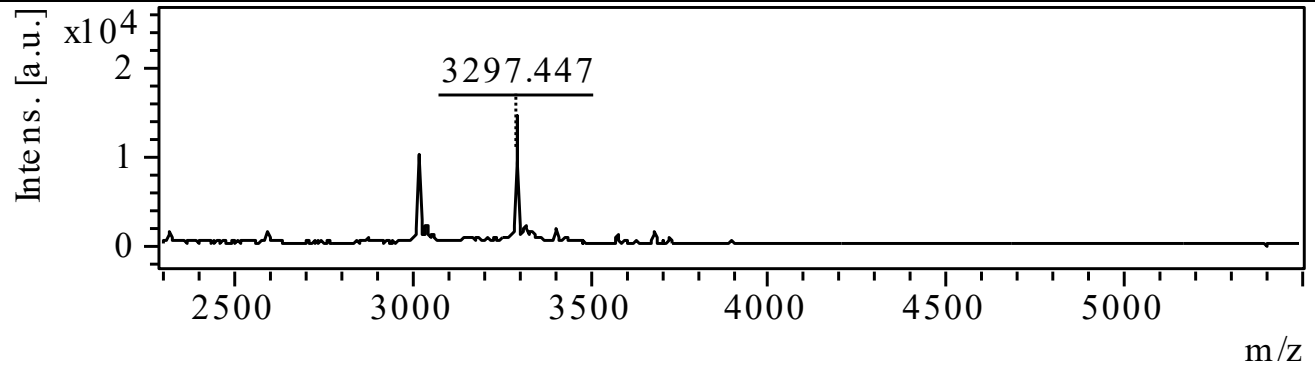
**Ex1-14**	$C_{145}H_{193}F_2N_{53}O_{50}S$	3548.517	3547.391	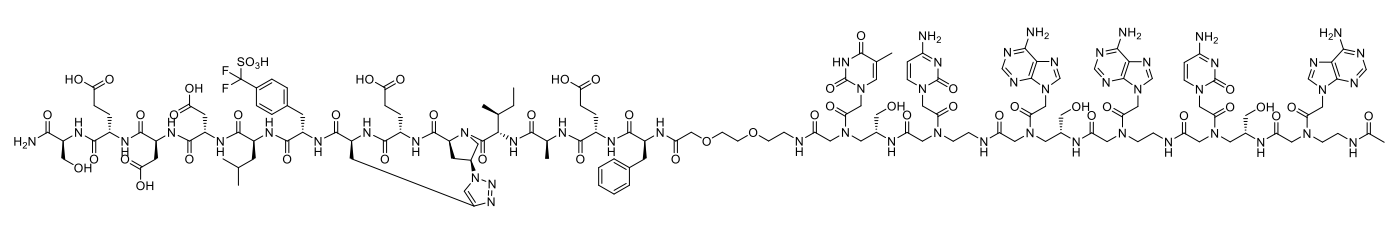	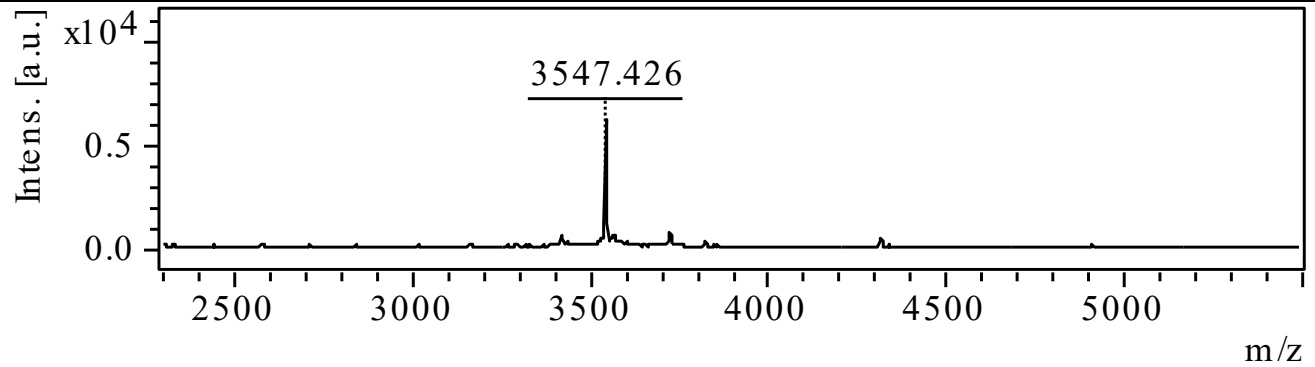
**Ex1-15**	$C_{133}H_{181}N_{51}O_{44}$	3198.224	3197.353	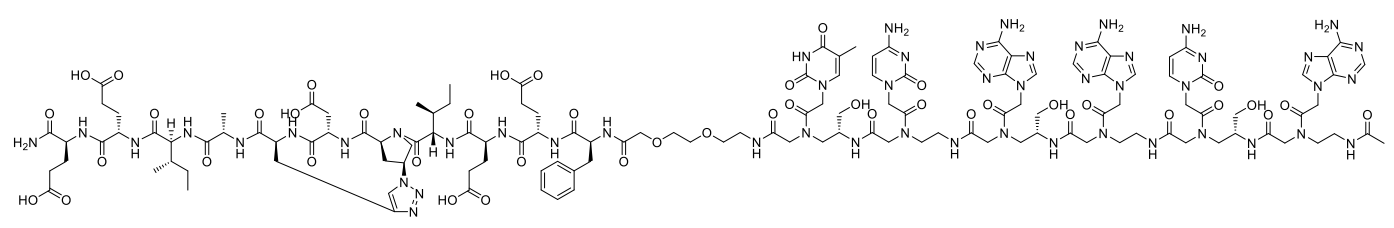	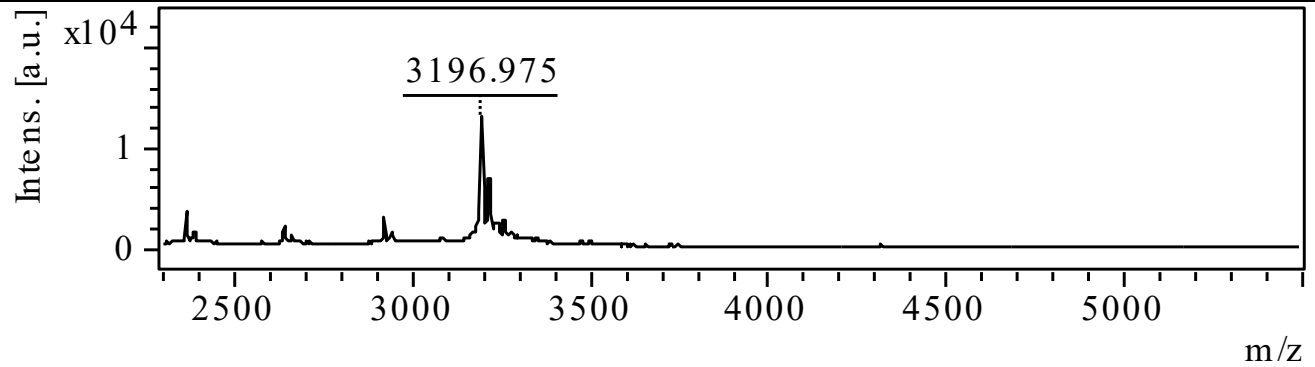
**Ex1-16**	$C_{140}H_{186}F_2N_{50}O_{49}S$	3423.386	3422.332	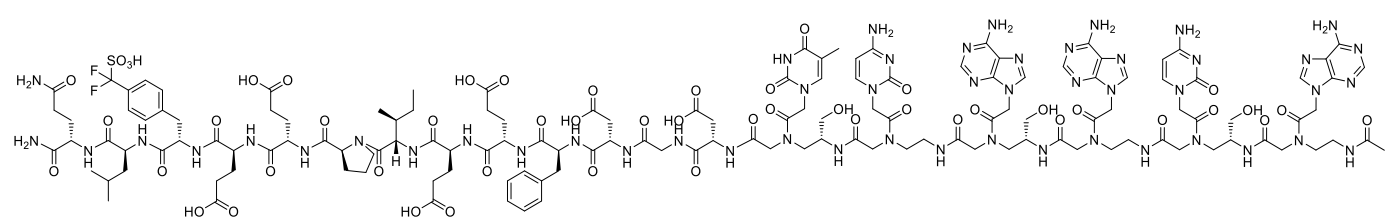	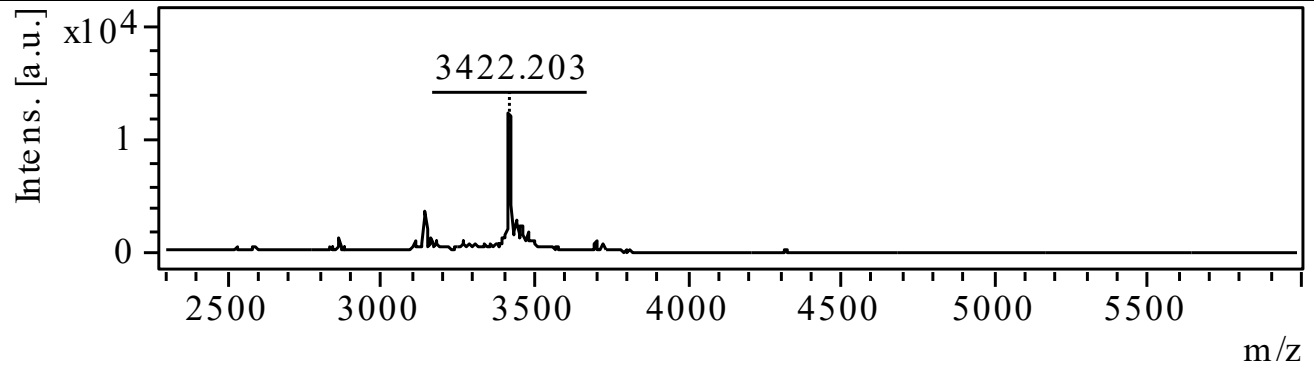
**Ex1-17**	$C_{142}H_{195}F_2N_{49}O_{48}S$	3426.474	3425.405	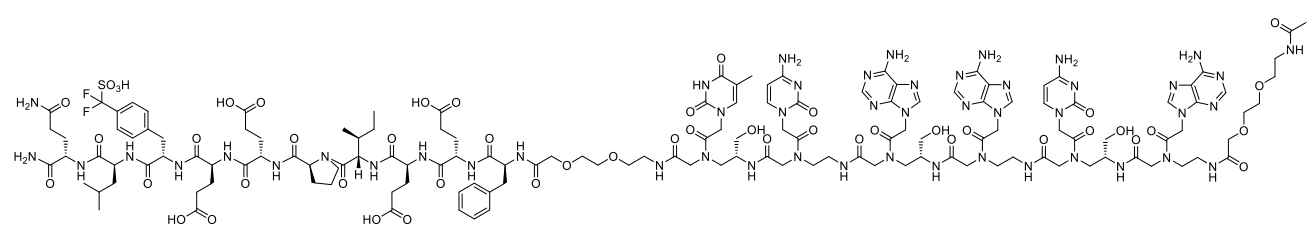	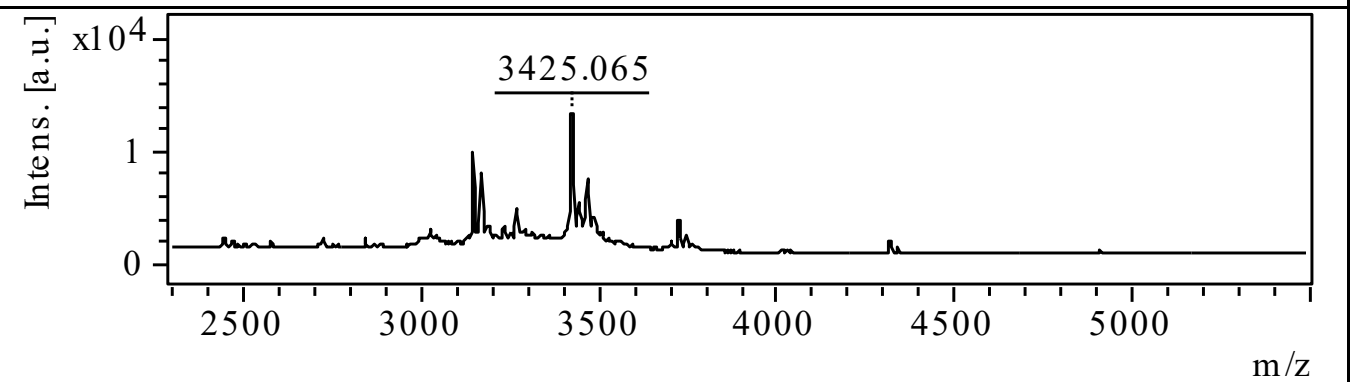

**Ex1-18**	$C_{143}H_{200}N_{53}O_{52}P$	3524.466	3523.441	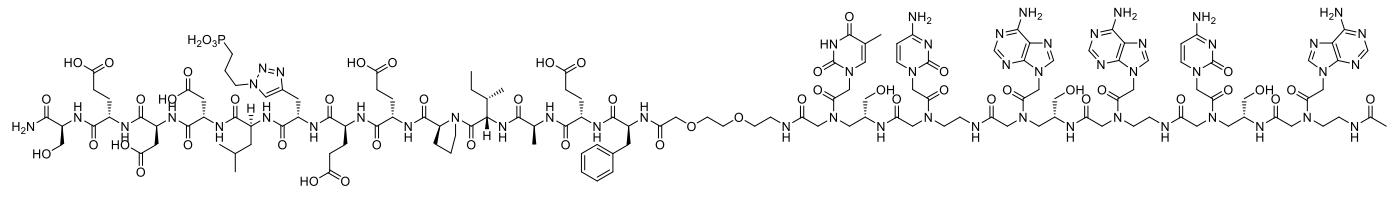	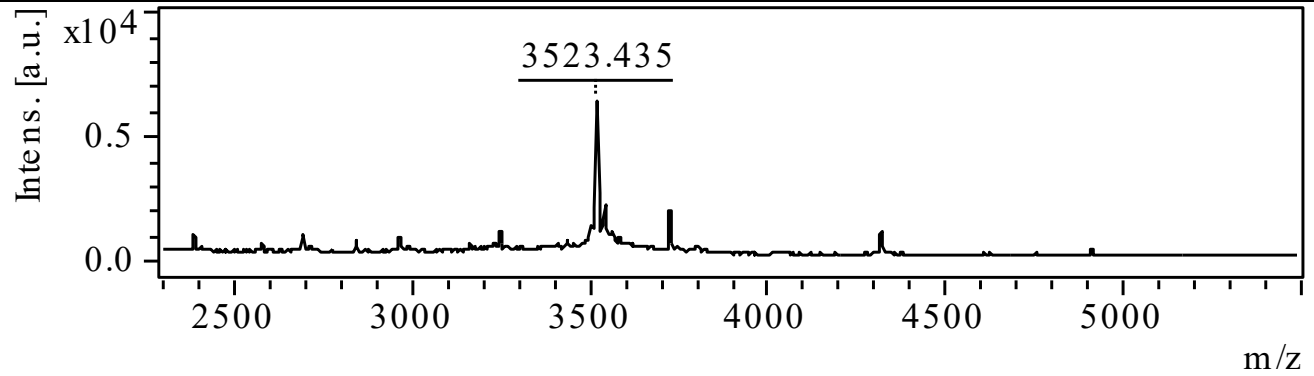
**Ex1-19**	$C_{145}H_{197}N_{51}O_{53}S$	3534.535	3533.404	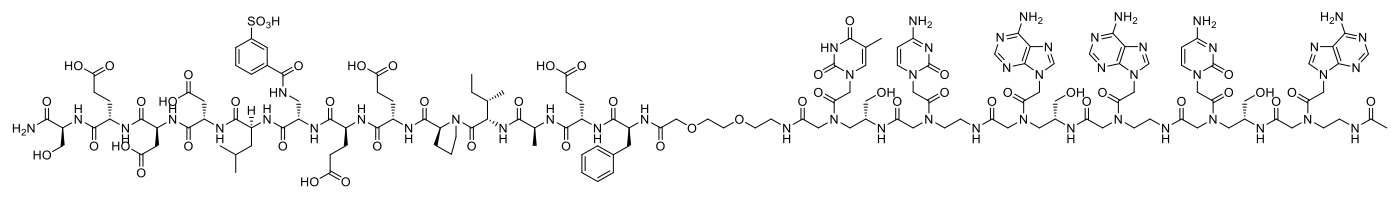	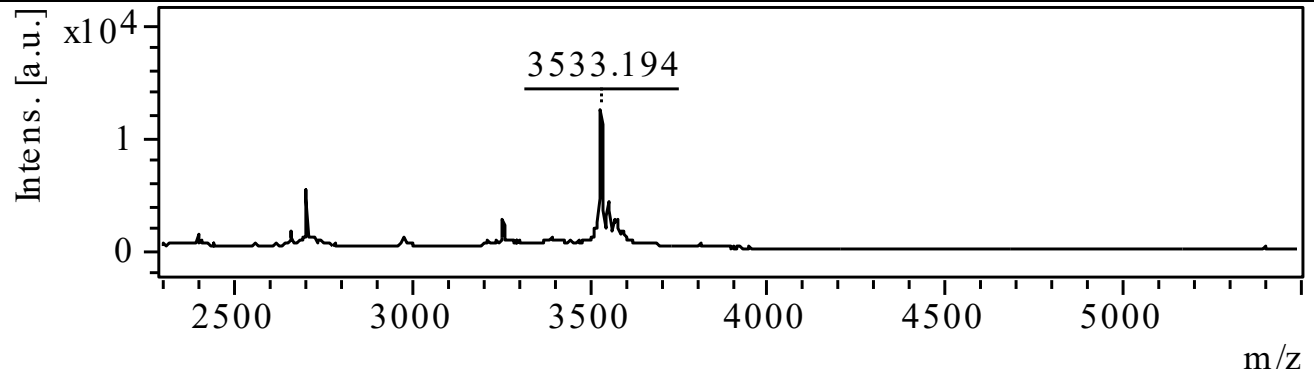
**Ex1-20**	$C_{151}H_{208}N_{52}O_{56}S$	3679.693	3678.478	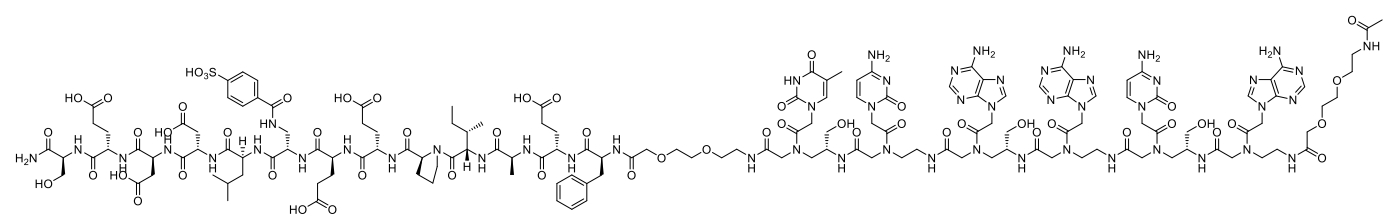	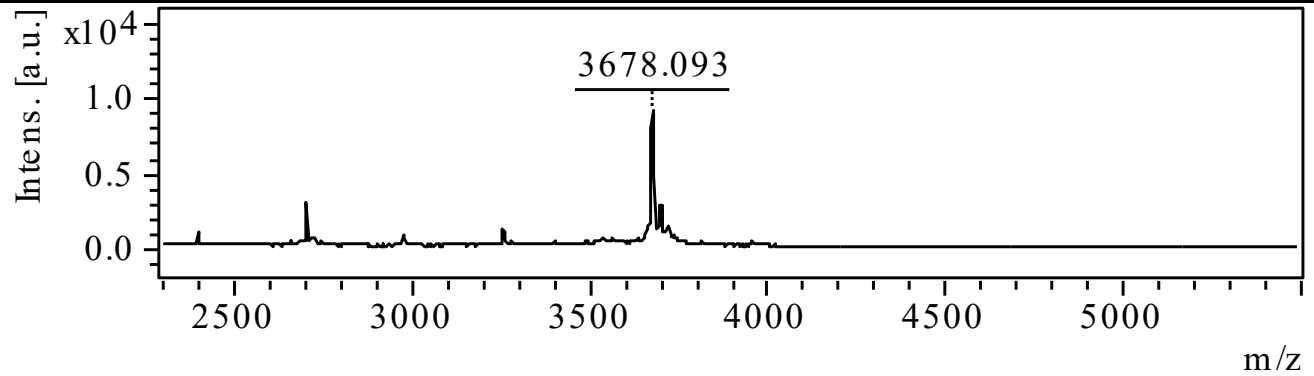
**Ex1-21**	$C_{142}H_{195}N_{53}O_{51}$	3460.442	3459.433	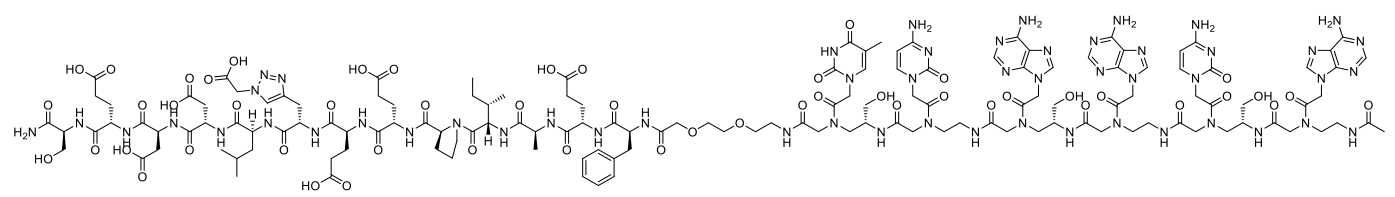	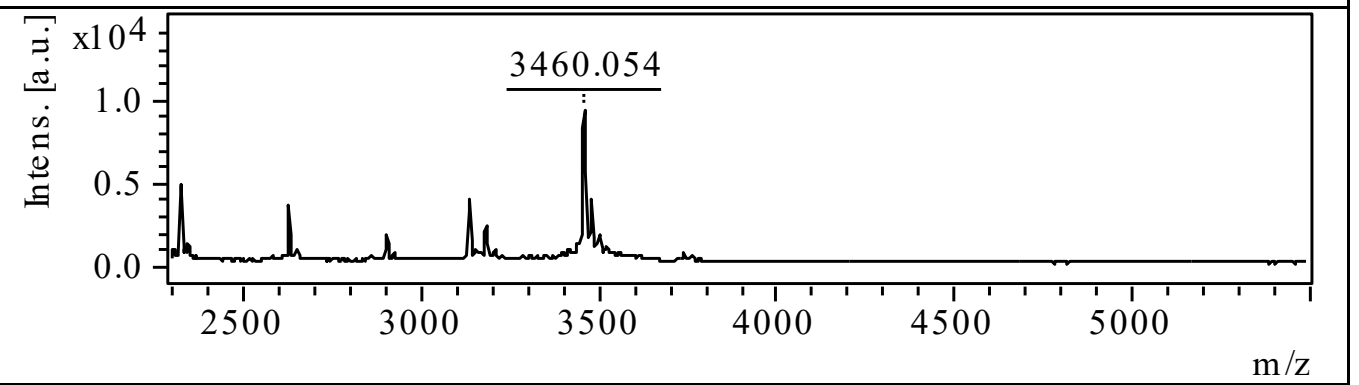
**Ex1-22**	$C_{135}H_{188}N_{50}O_{47}$	3263.292	3262.389	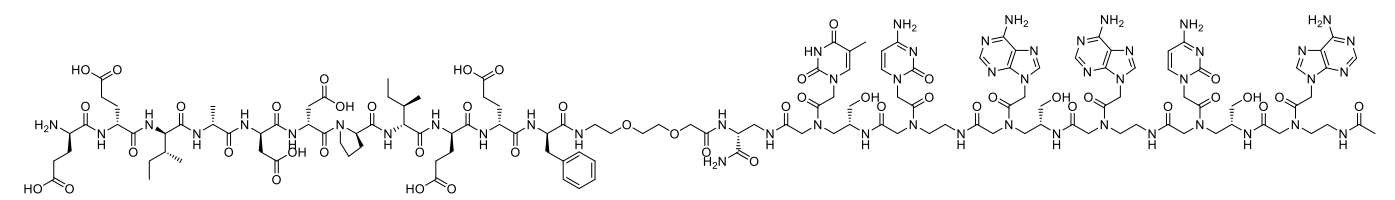	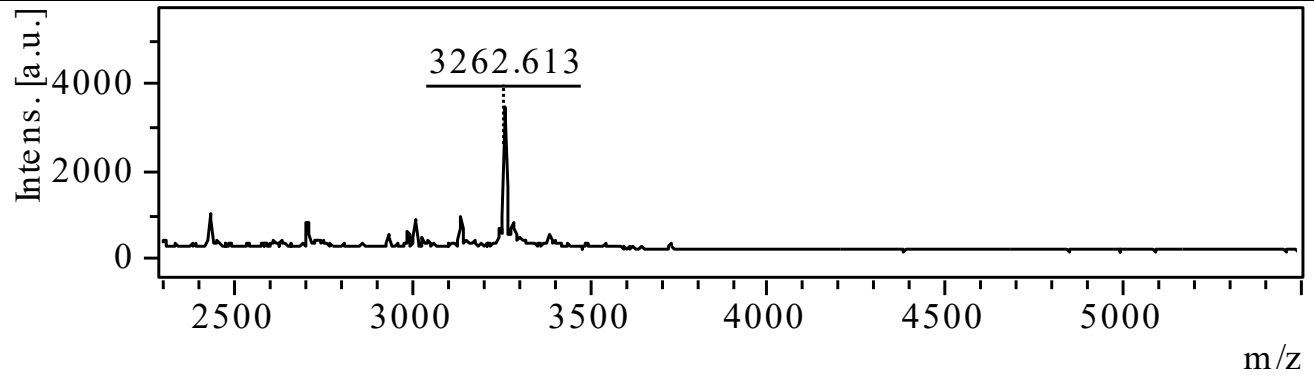
**Ex1-23**	$C_{147}H_{202}N_{52}O_{51}$	3513.546	3512.485	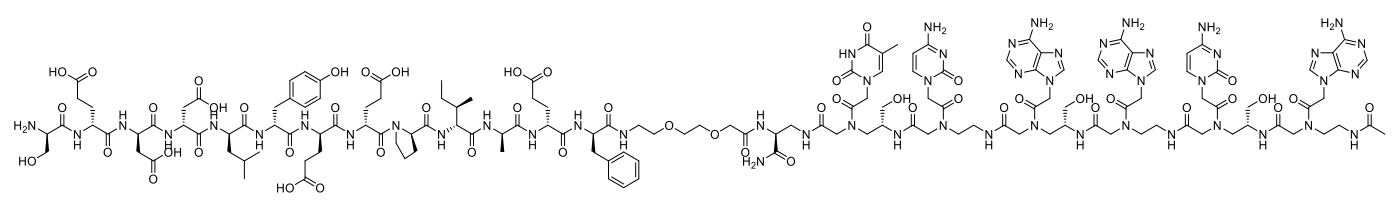	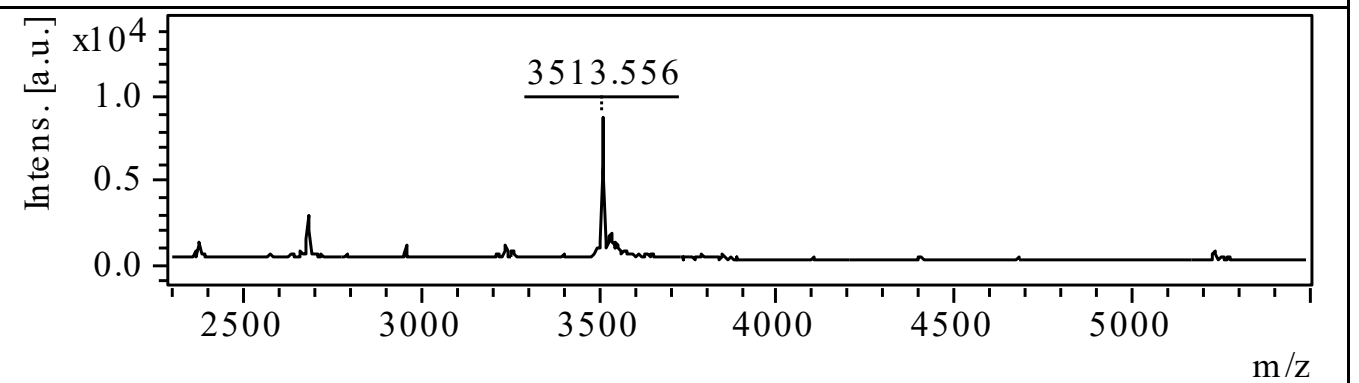

**Ex1-24**	$C_{131}H_{178}N_{48}O_{44}$	3129.157	3128.320	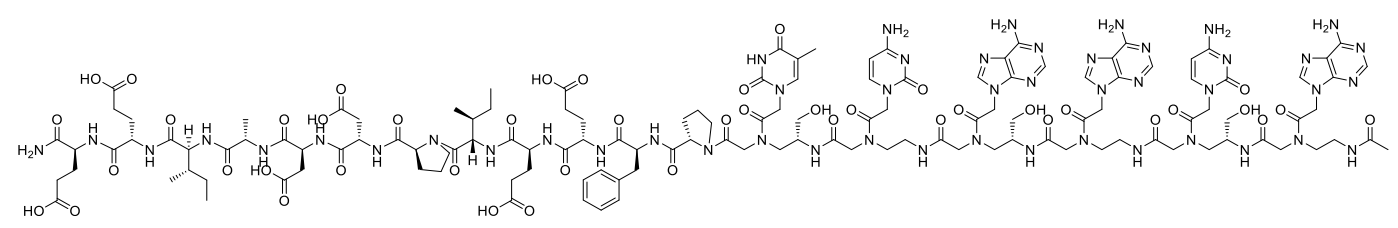	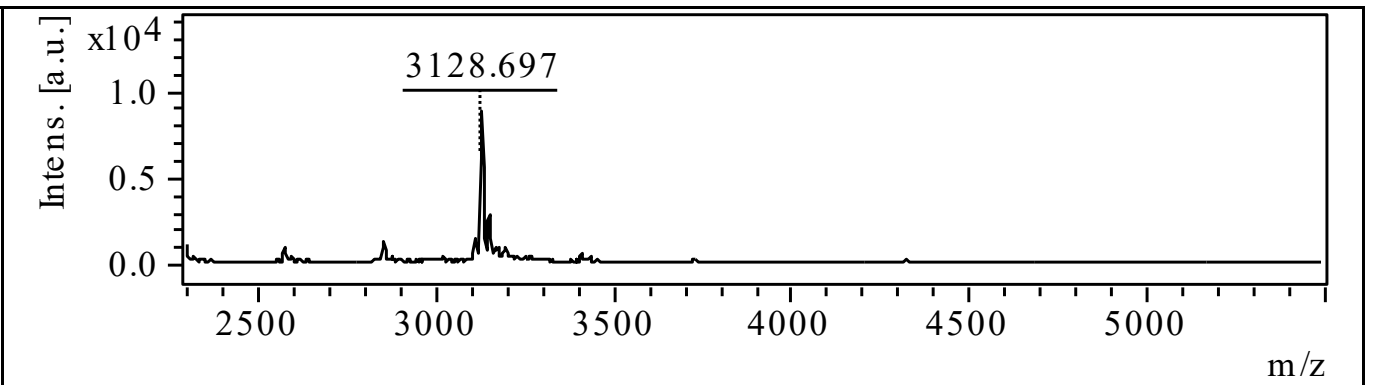
**Ex1-25**	$C_{145}H_{197}N_{51}O_{51}$	3470.477	3469.442 *3,453.443 (Deg. of NO_2 on MALDI)	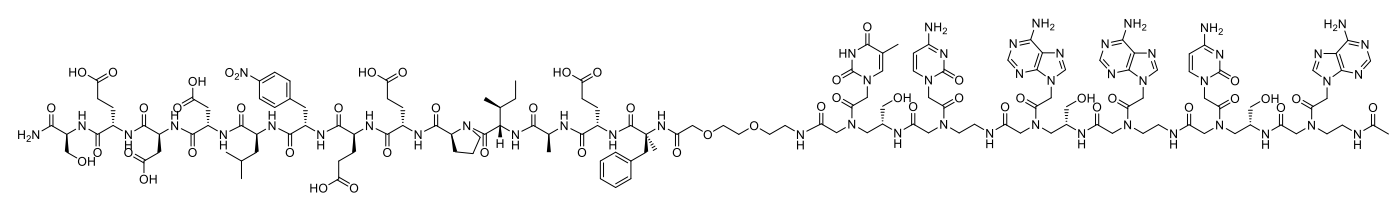	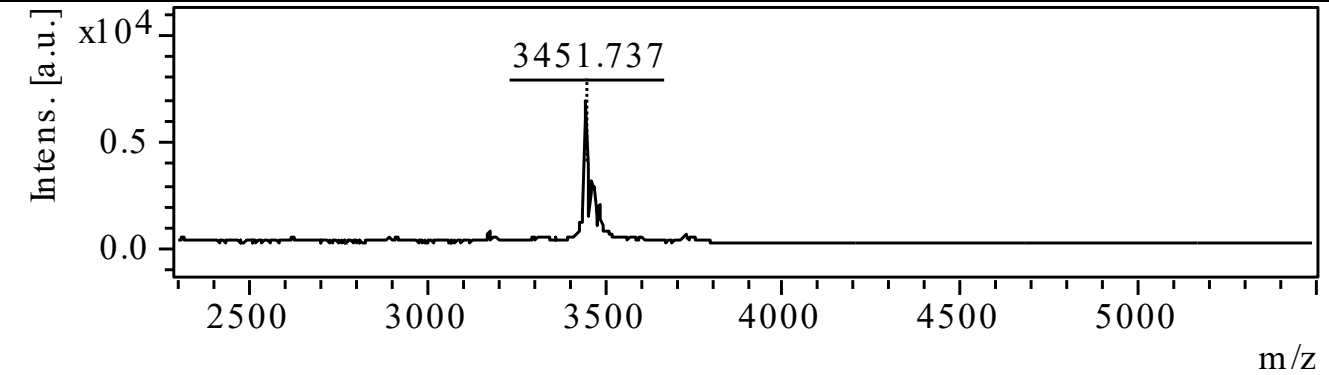
**Ex1-26**	C146H199 N52O54P	3577.482	3576.420 *3,560.421 (Deg. of NO_2 on MALDI)	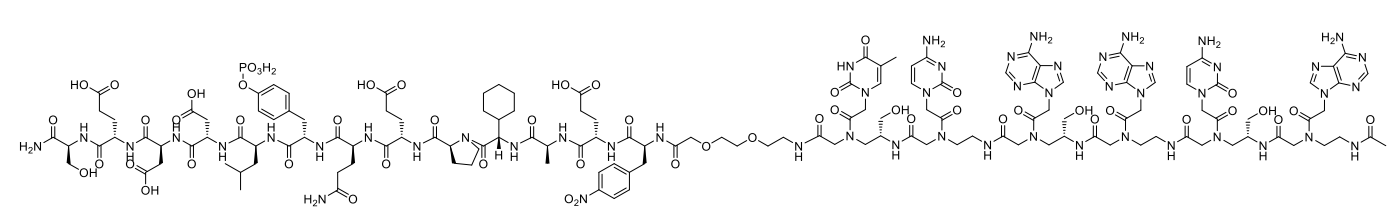	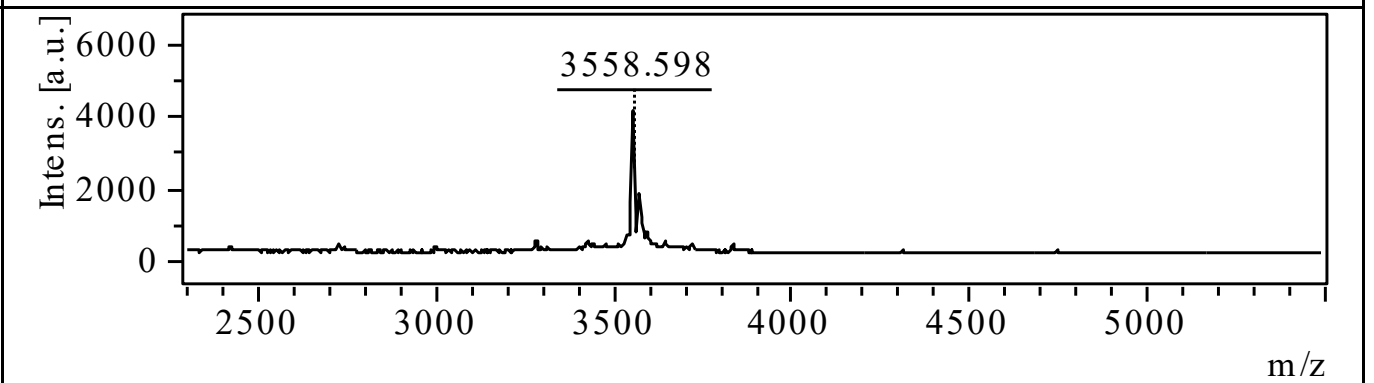
**Ex1-27**	$C_{137}H_{189}F_2N_{52}O_{49}P$	3417.305	3416.364	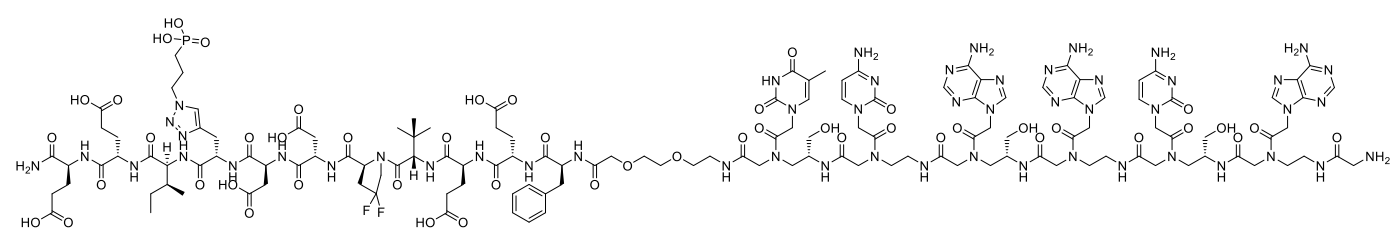	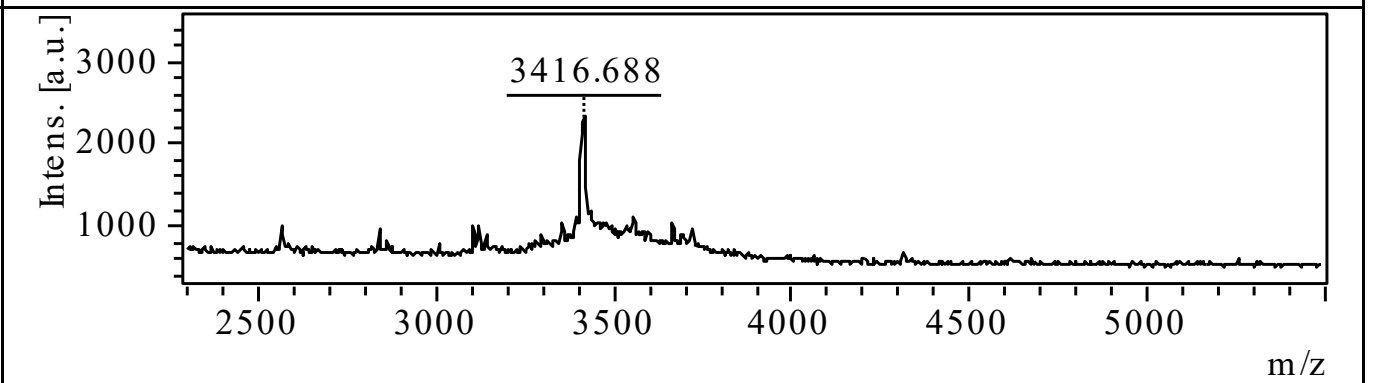
**Ex1-28**	$C_{129}H_{176}F_2N_{49}O_{43}P$	3170.098	3169.283	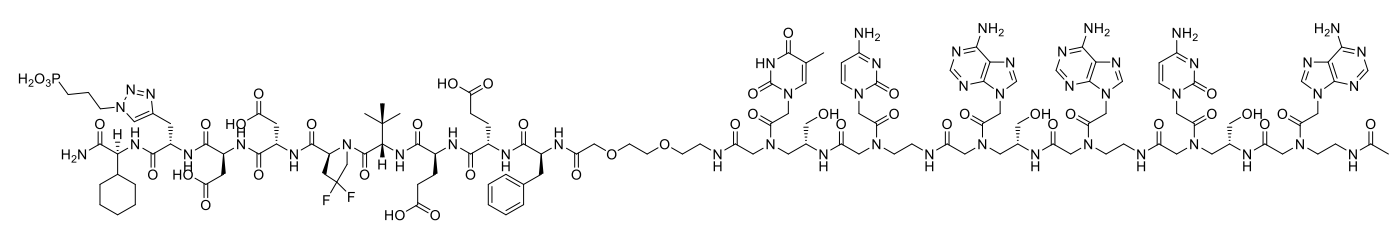	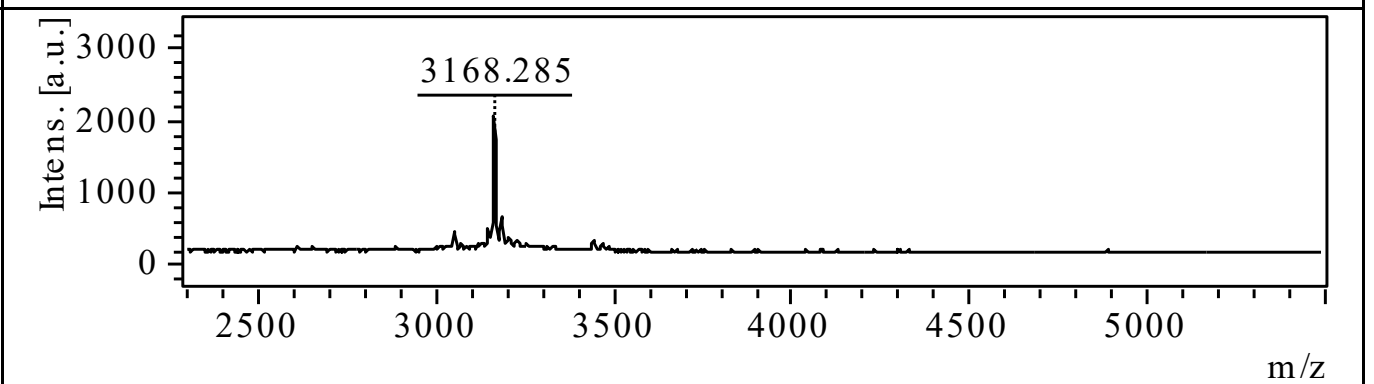
**Ex1-29**	$C_{138}H_{193}F_2N_{49}O_{48}$	3344.354	3343.417	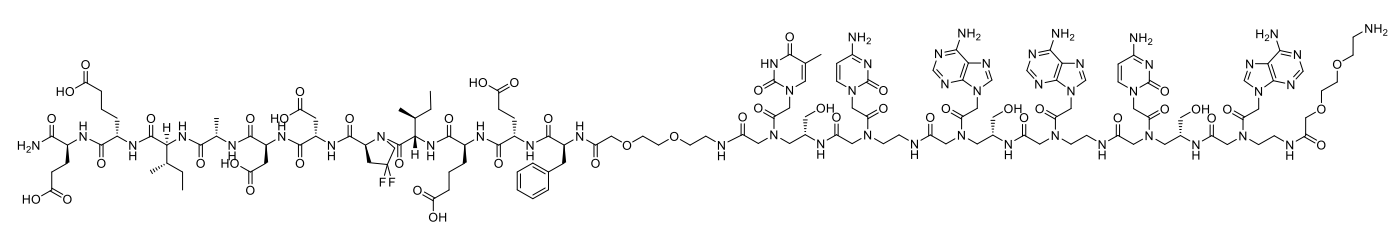	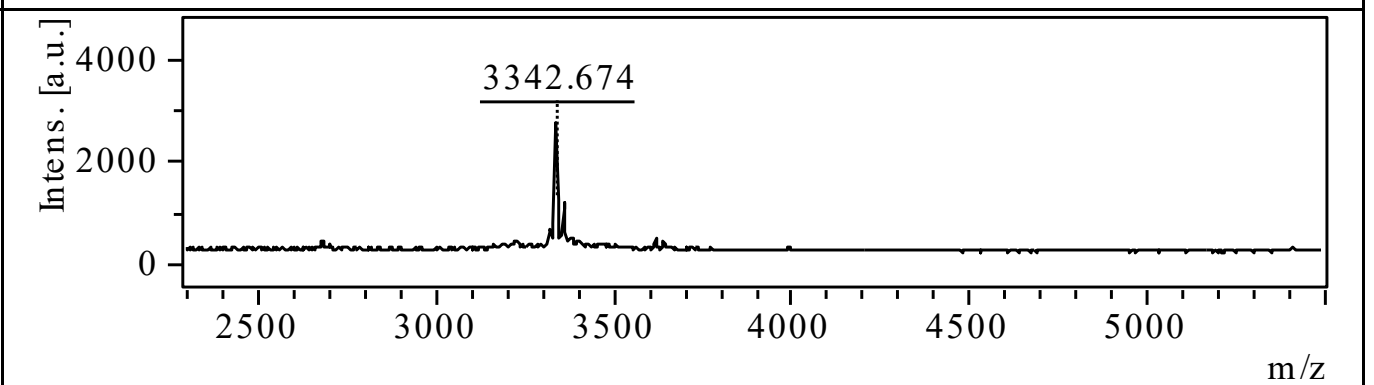

**Ex1-30**	$C_{133}H_{182}N_{48}O_{47}$	3205.208	3204.336	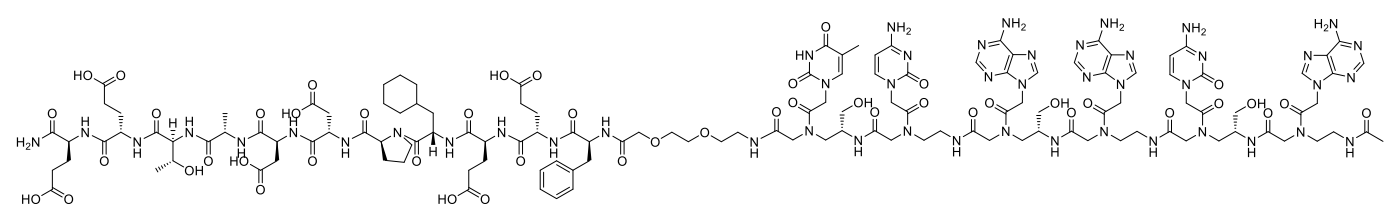	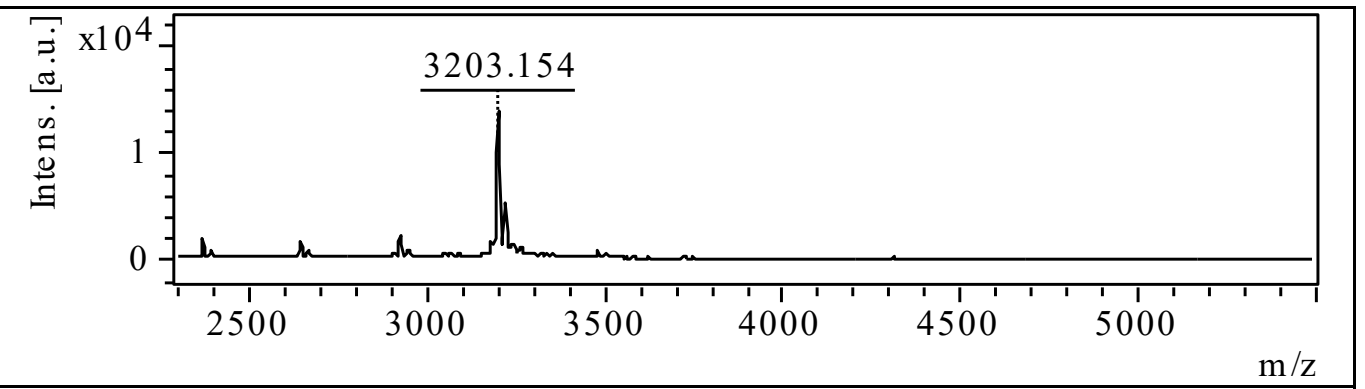
**Ex1-31**	$C_{135}H_{184}N_{48}O_{46}$	3215.247	3214.357	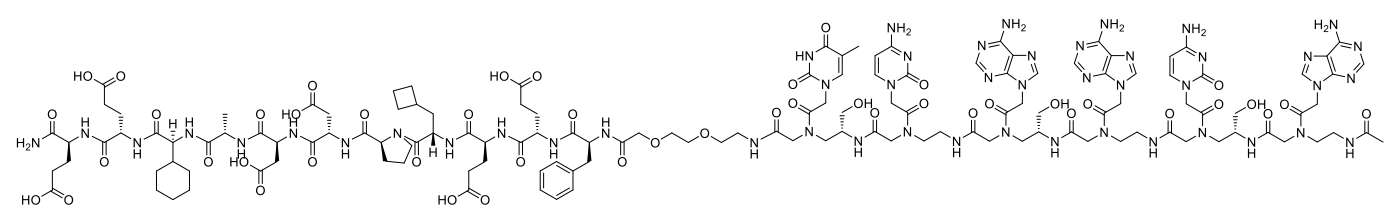	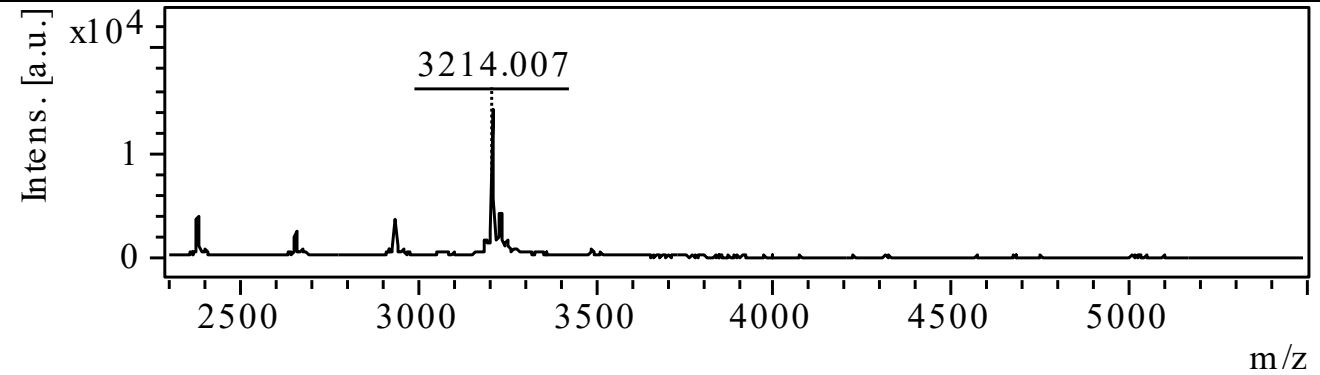
**Ex1-32**	$C_{117}H_{169}N_{51}O_{36}$	2865.960	2865.299	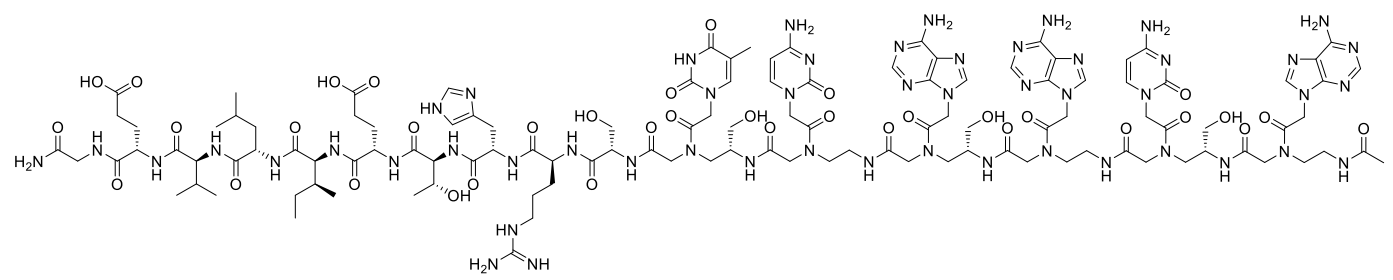	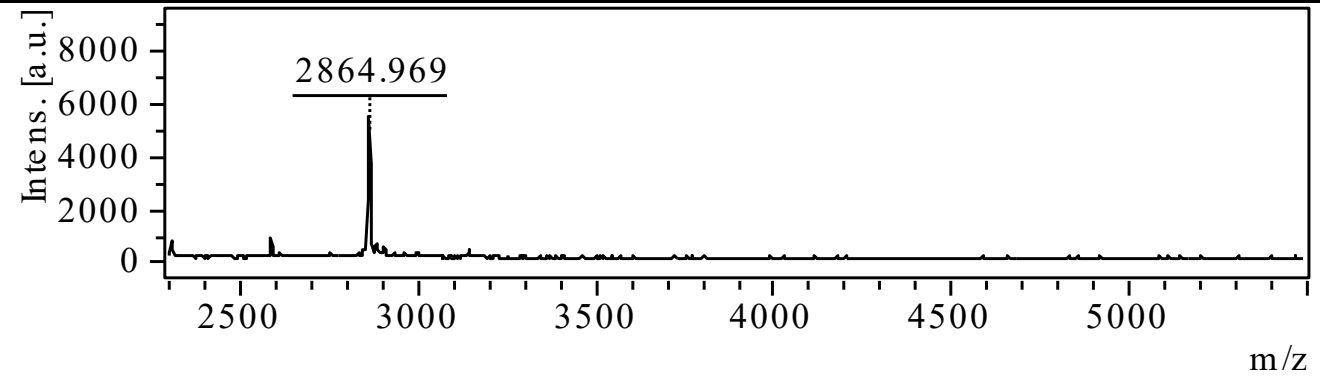
**Ex1-33**	$C_{119}H_{165}N_{49}O_{37}$	2873.935	2873.257	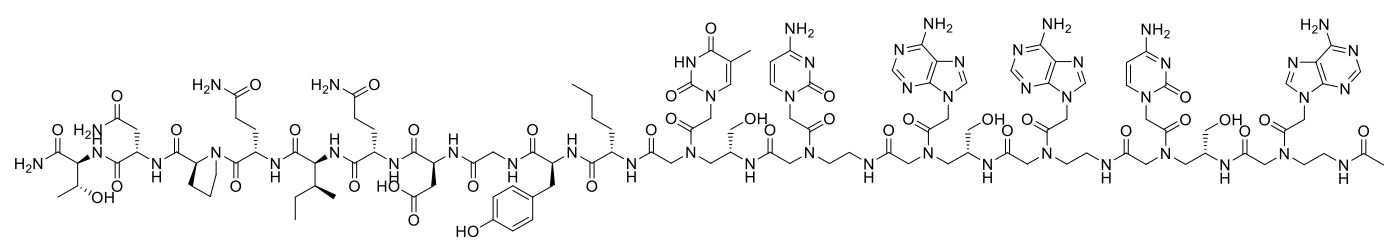	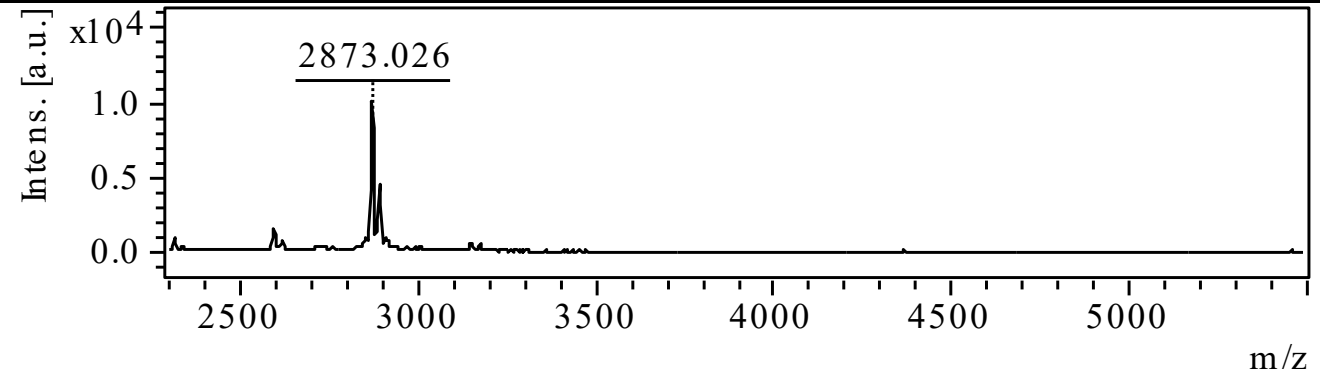
**Ex1-34**	$C_{113}H_{167}N_{53}O_{35}$	2827.915	2827.295	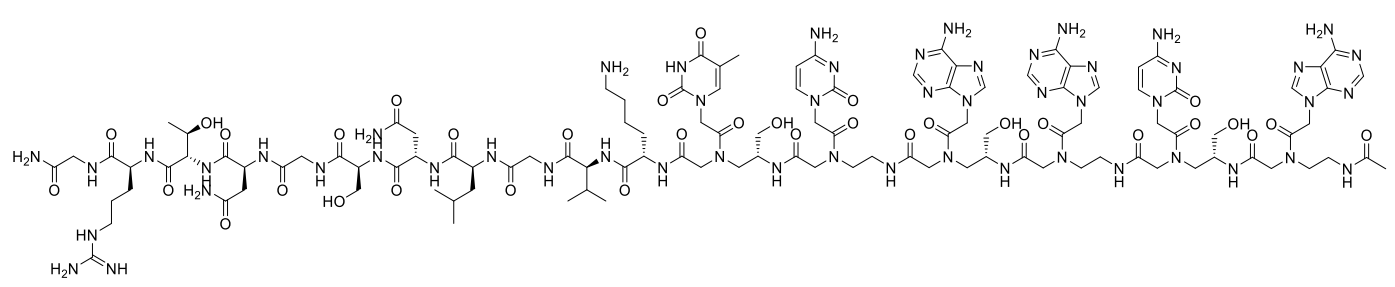	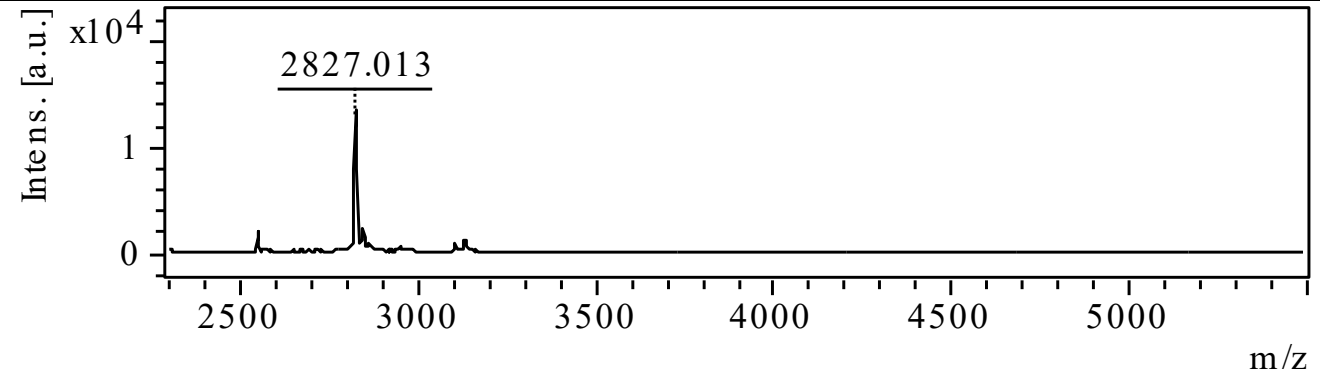
**Ex1-35**	$C_{117}H_{171}N_{51}O_{38}$	2899.974	2899.305	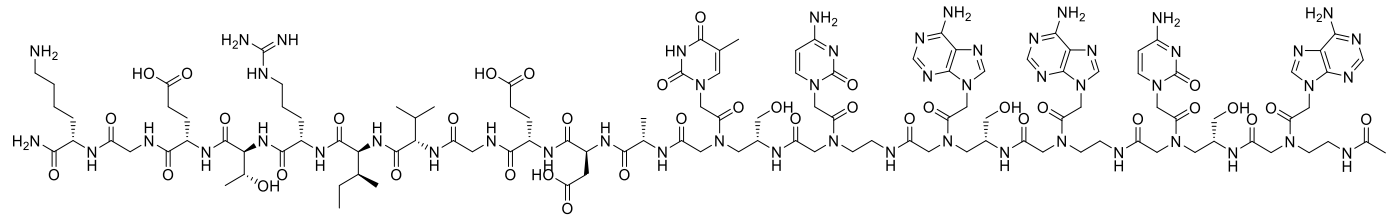	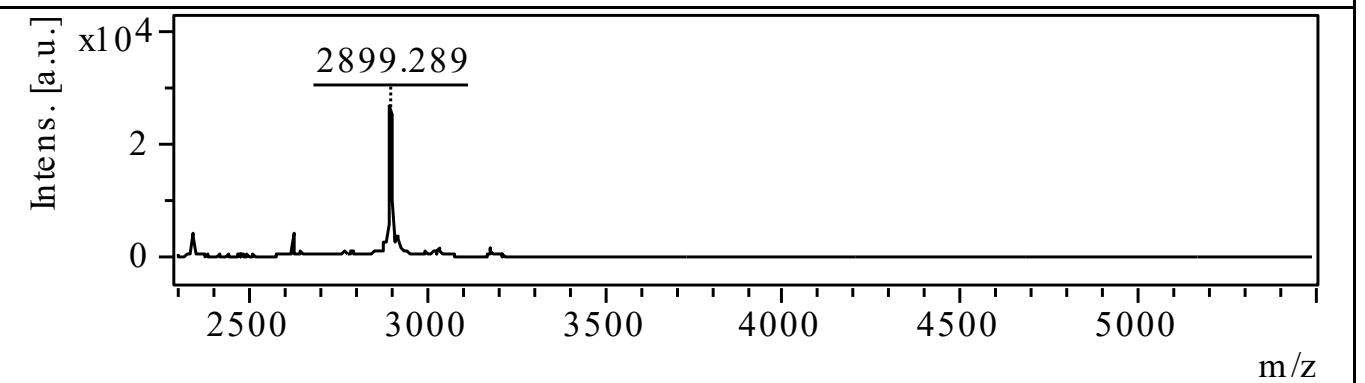

**Ex1-36**	$C_{134}H_{195}N_{53}O_{40}$	3188.365	3187.489	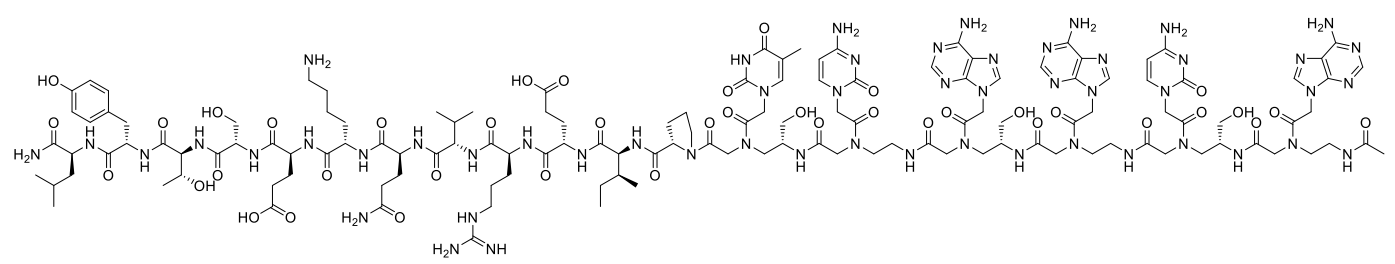	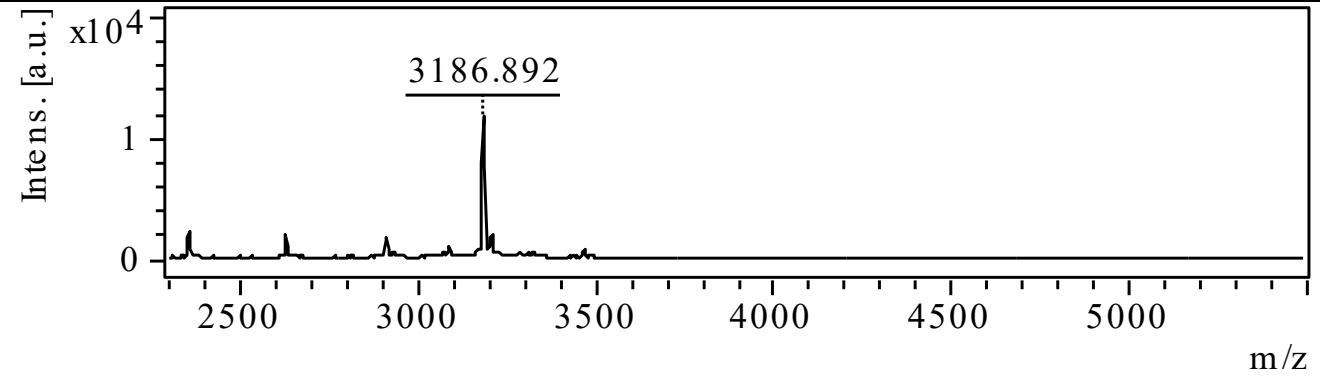
**Ex1-37**	$C_{135}H_{193}N_{53}O_{39}$	3182.361	3181.478	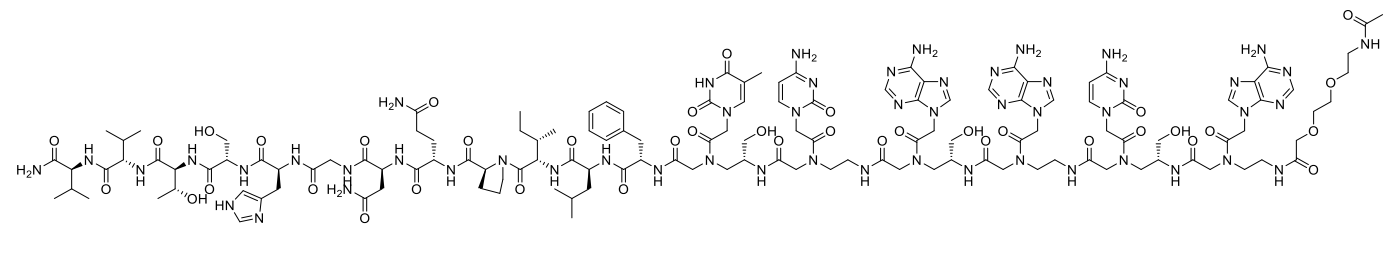	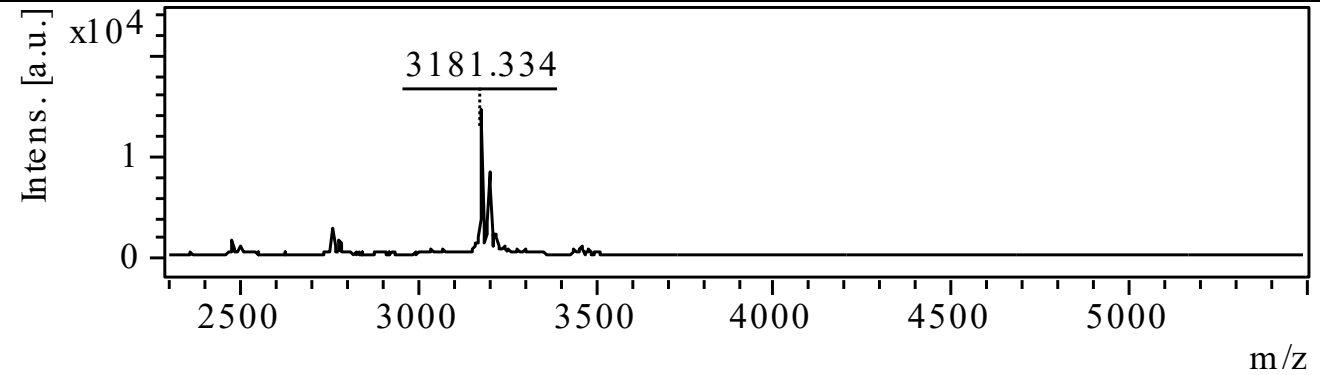
**Ex1-38**	$C_{118}H_{162}N_{50}O_{36}S$	2888.968	2888.214	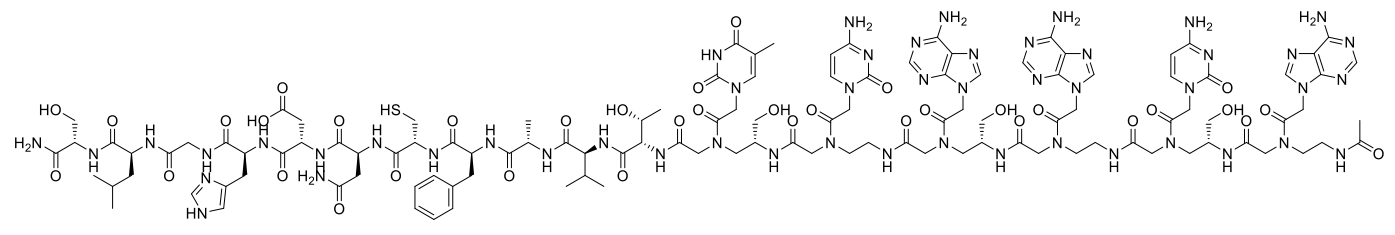	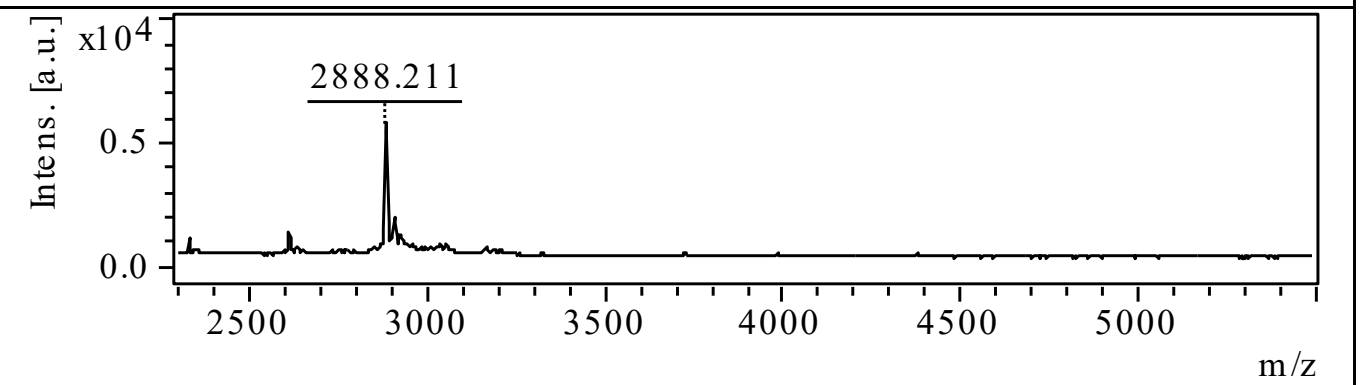
**Ex1-39**	$C_{132}H_{192}N_{54}O_{38}$	3143.328	3142.479	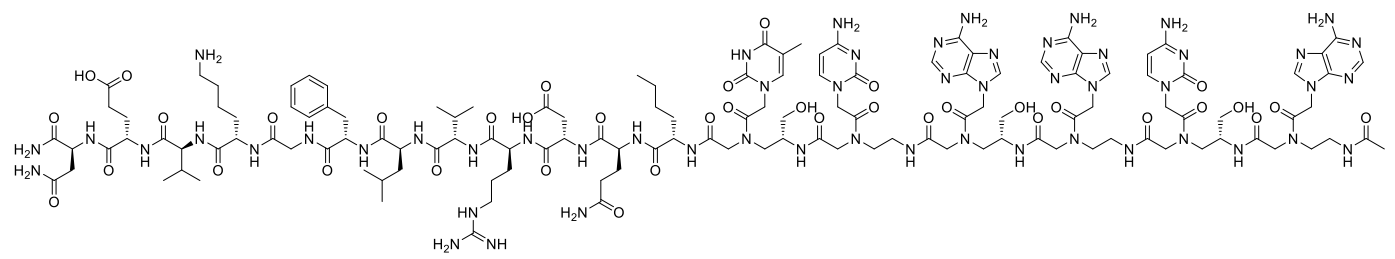	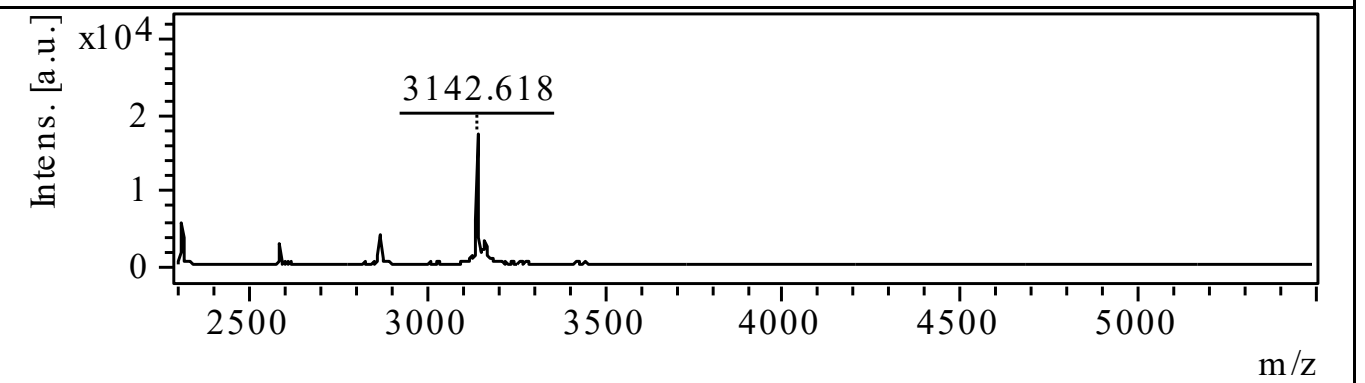
**Ex1-40**	$C_{128}H_{182}N_{56}O_{38}$	3113.218	3112.406	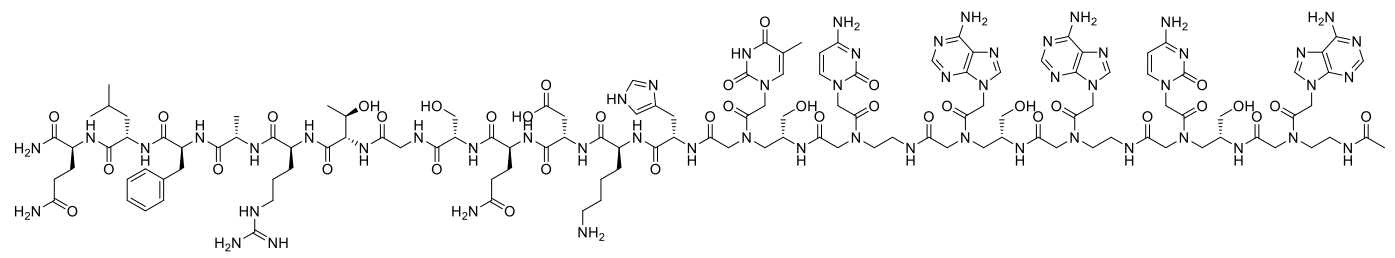	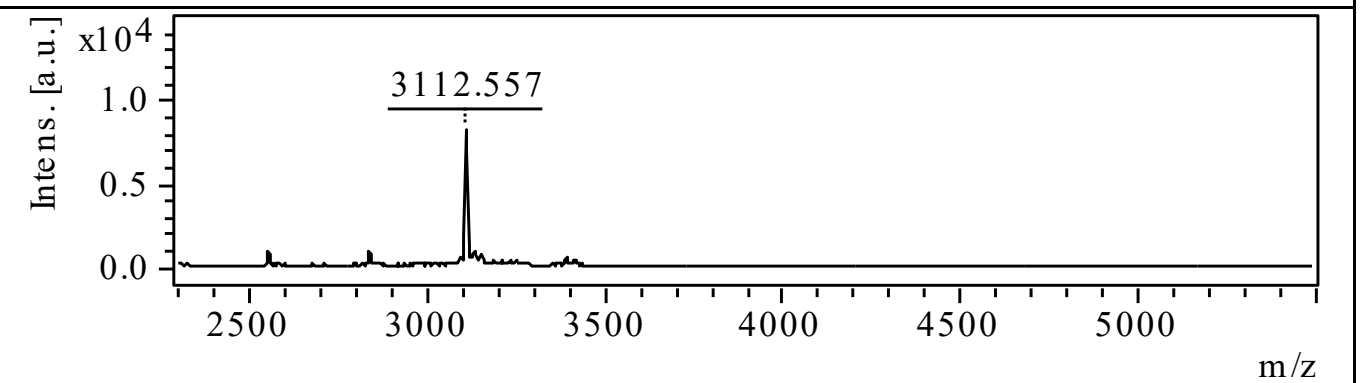
**Ex1-41**	$C_{123}H_{181}N_{51}O_{38}$	2982.12	2981.383	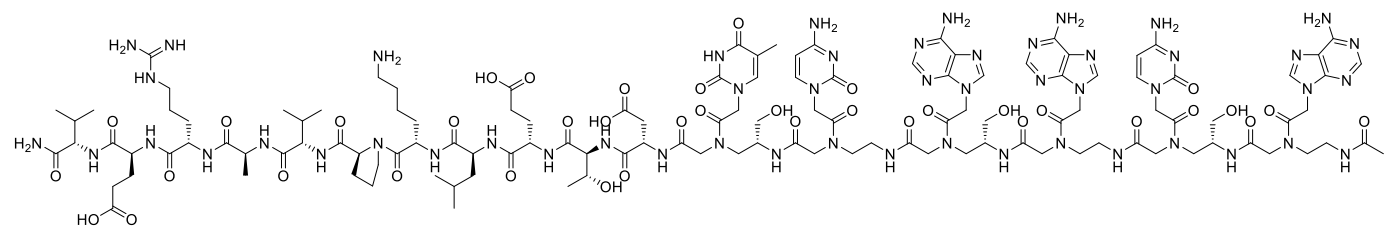	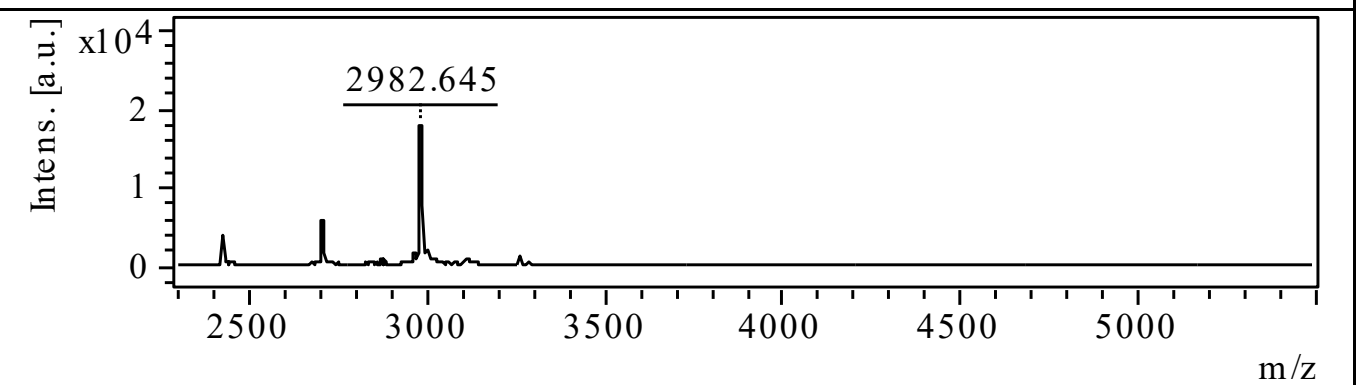

**Ex1-42**	$C_{129}H_{182}N_{54}O_{44}$	3193.209	3192.370	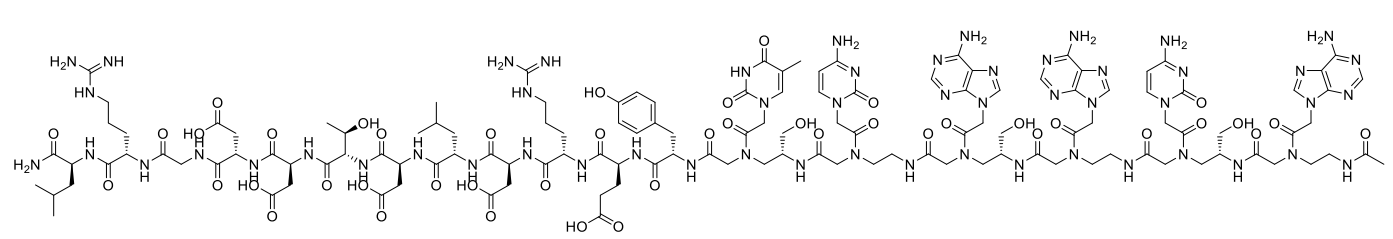	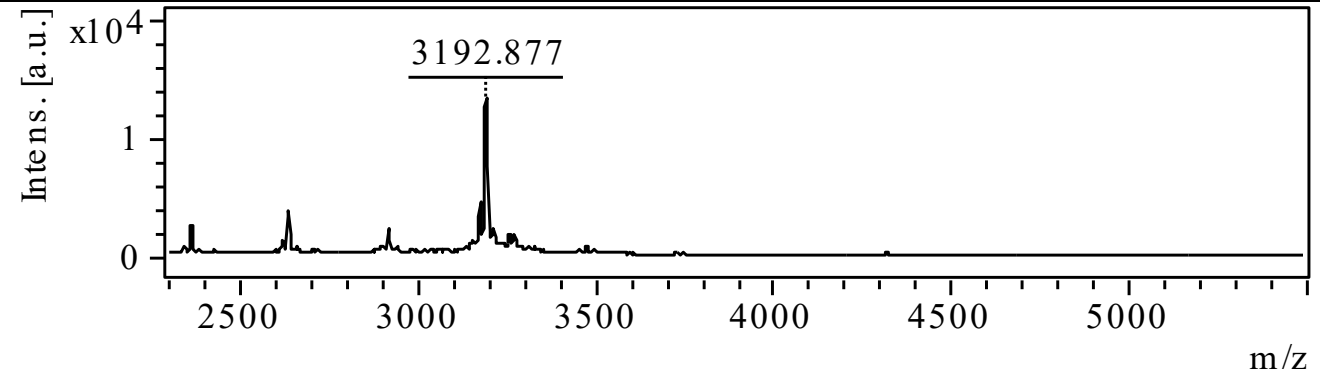
**Ex1-43**	$C_{126}H_{177}N_{55}O_{40}$	3102.147	3101.354	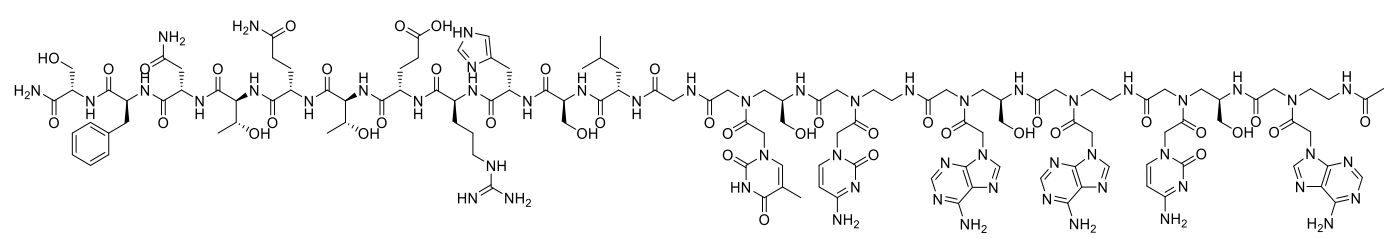	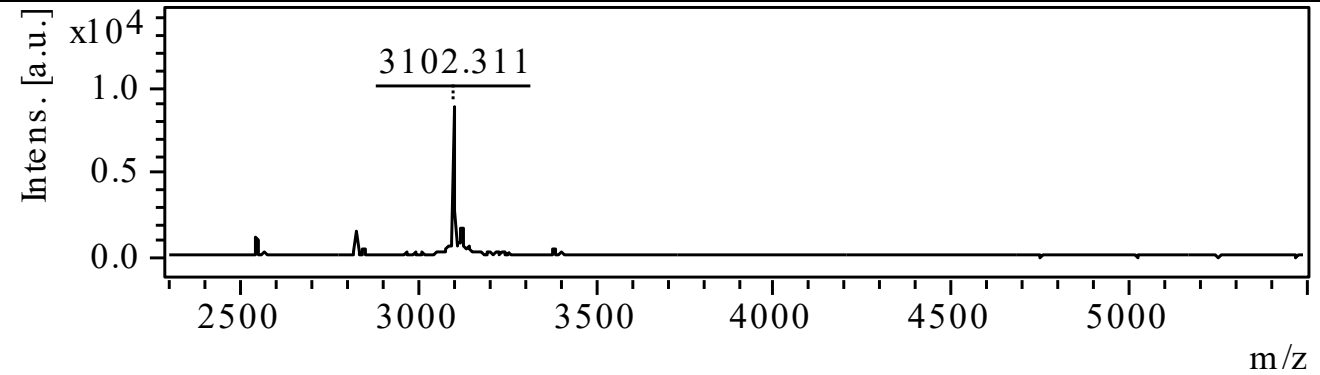
**Ex1-44**	$C_{117}H_{170}N_{52}O_{36}$	2880.975	2880.310	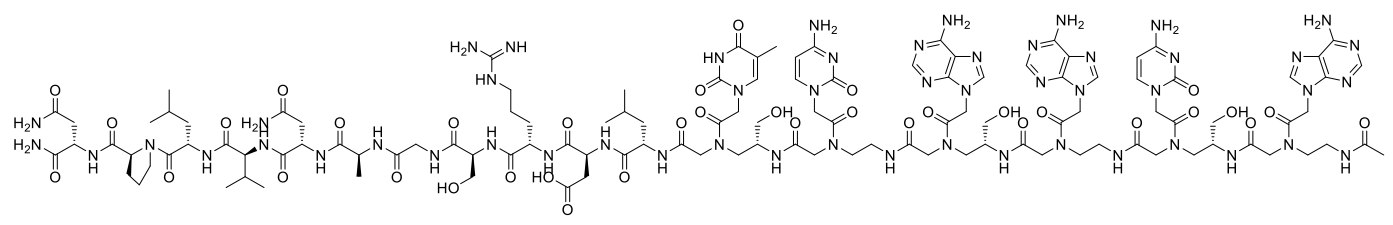	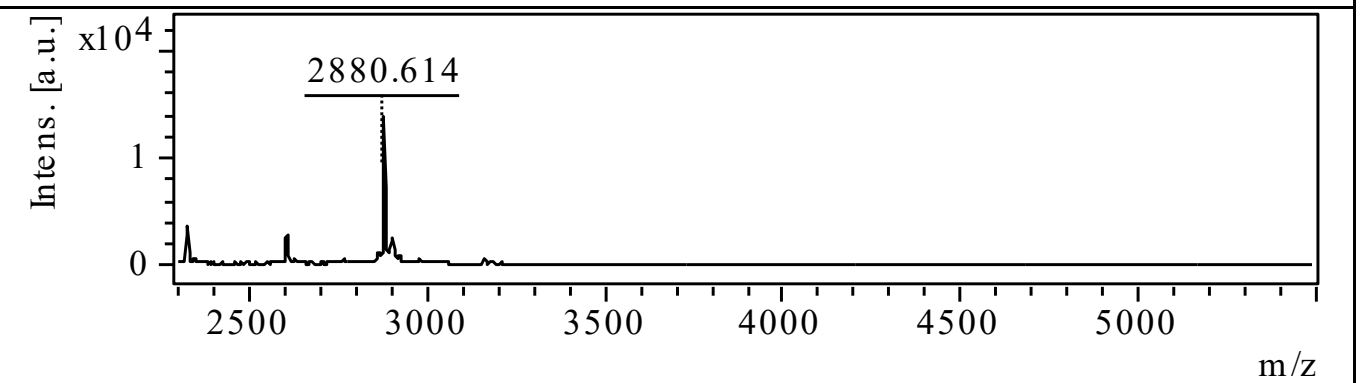
**Ex1-45**	$C_{132}H_{197}N_{57}O_{40}$	3222.387	3221.517	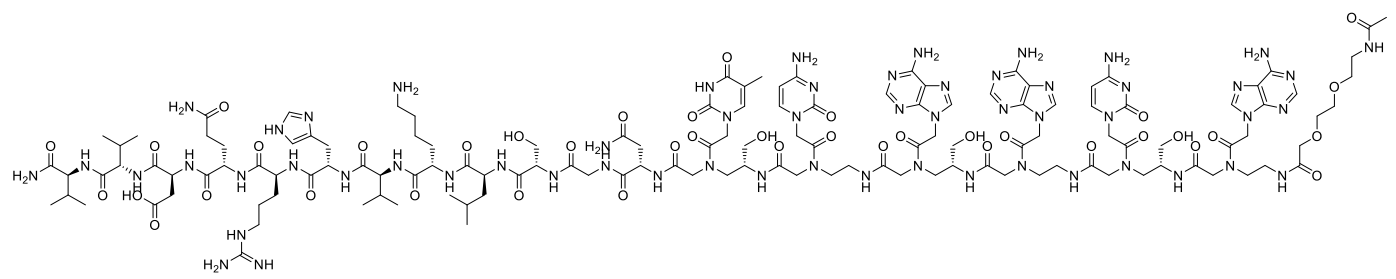	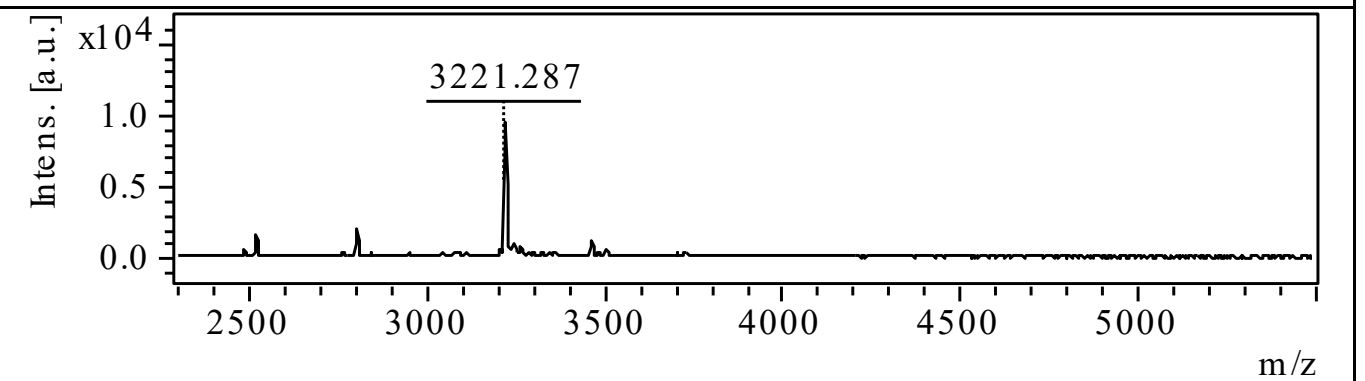
**Ex1-46**	$C_{122}H_{175}N_{53}O_{37}$	2976.076	2975.347	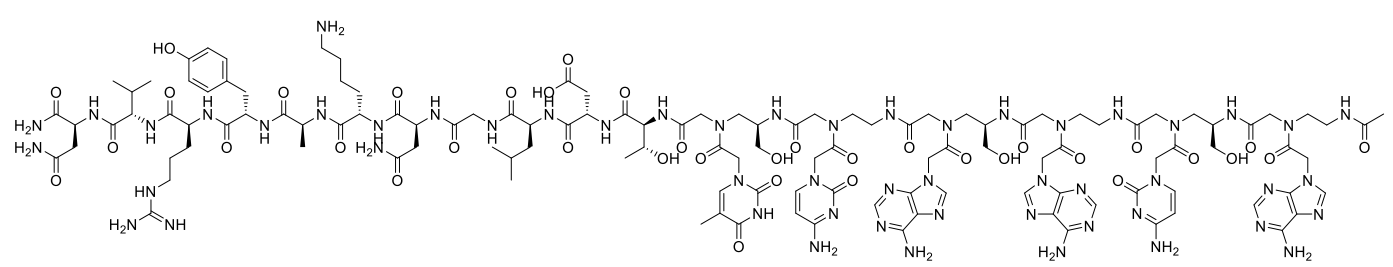	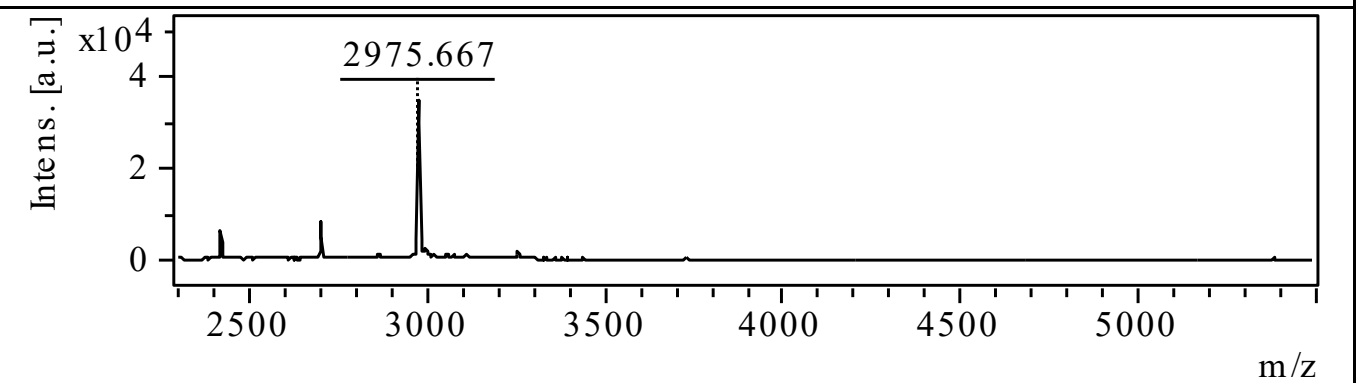
**Ex1-47**	$C_{124}H_{184}N_{54}O_{38}$	3039.176	3038.416	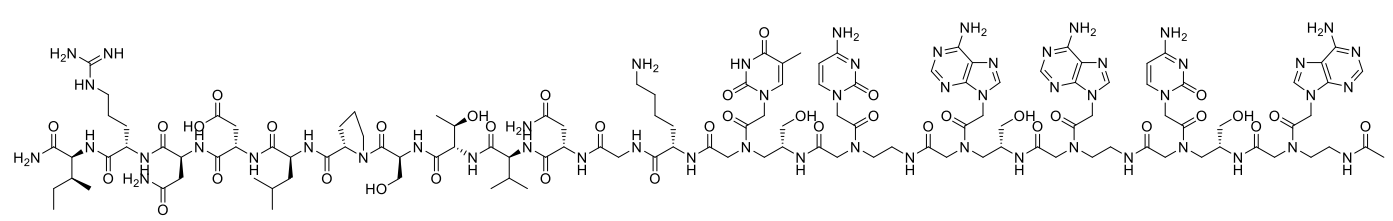	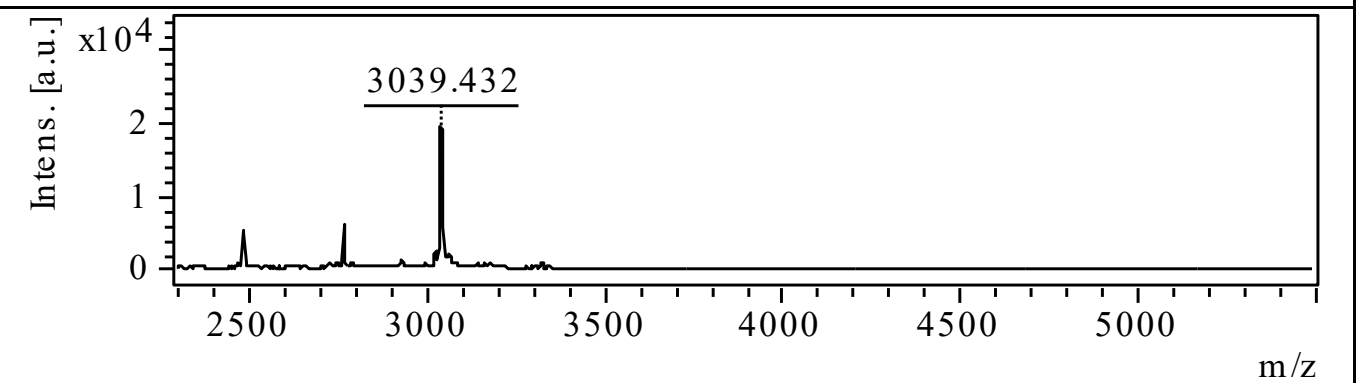

**Ex1-48**	$C_{127}H_{180}N_{52}O_{40}$	3075.161	3074.368	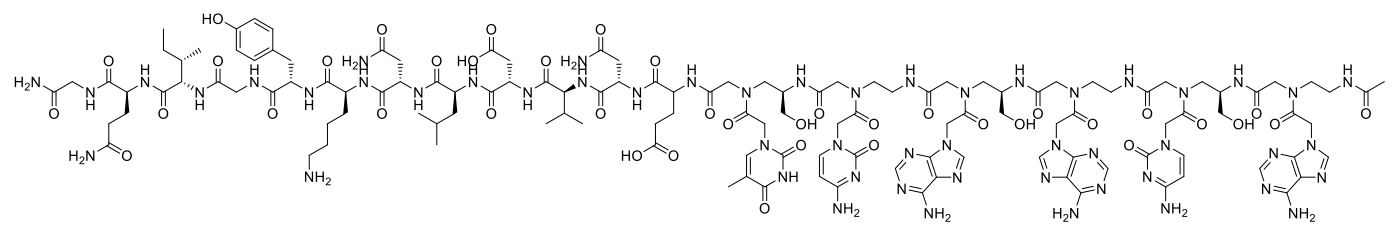	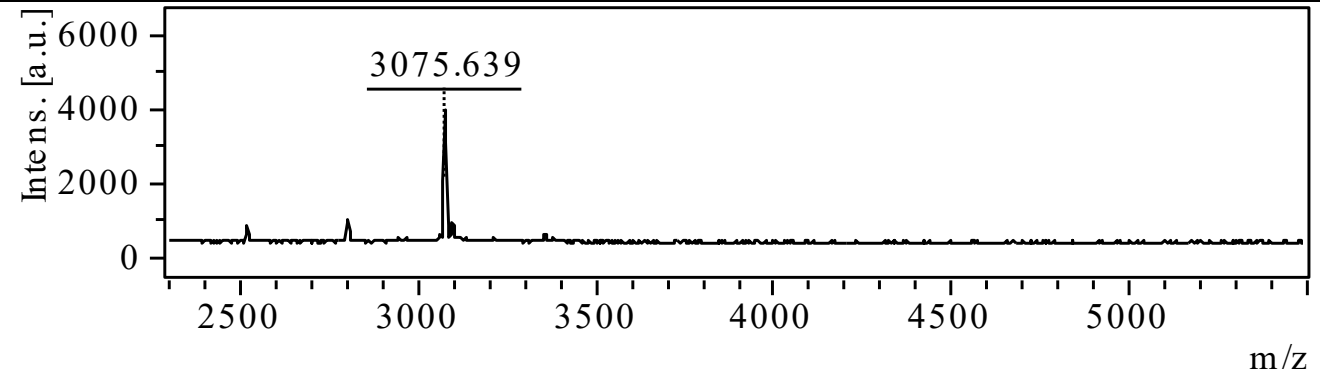
**Ex1-49**	$C_{124}H_{177}N_{53}O_{36}$	2986.115	2985.368	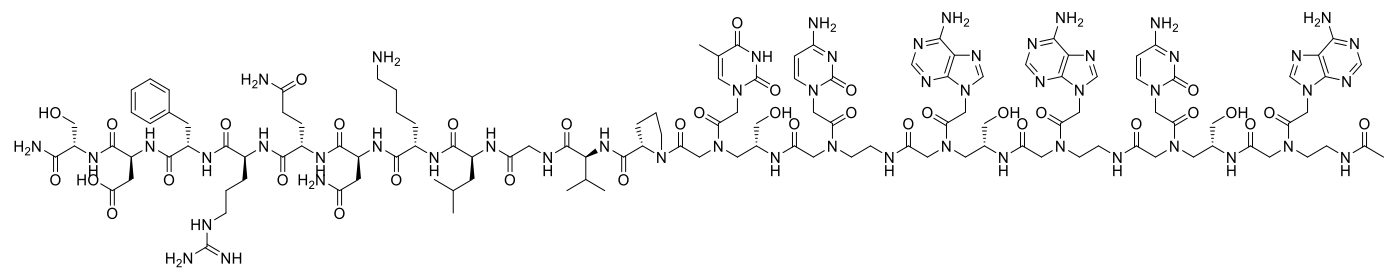	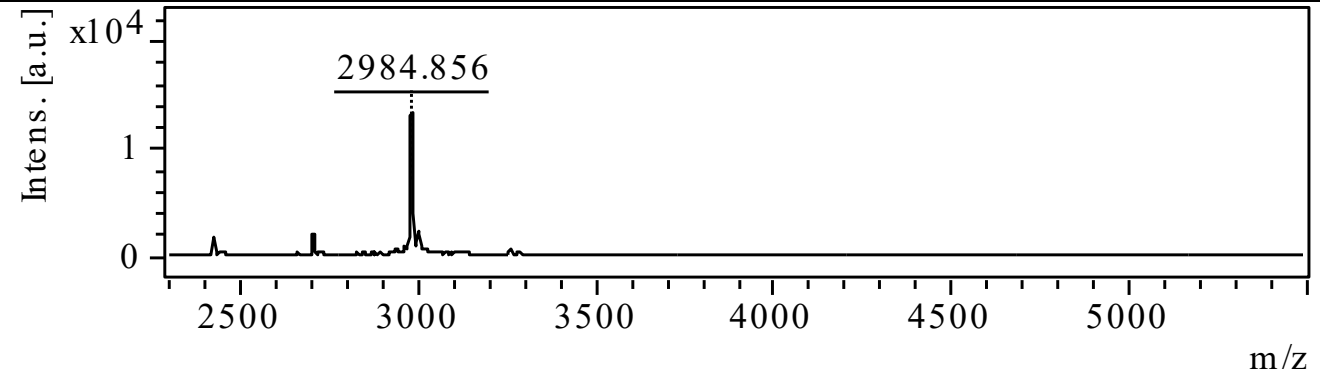
**Ex1-50**	C128H181 N55O37	3082.204	3081.401	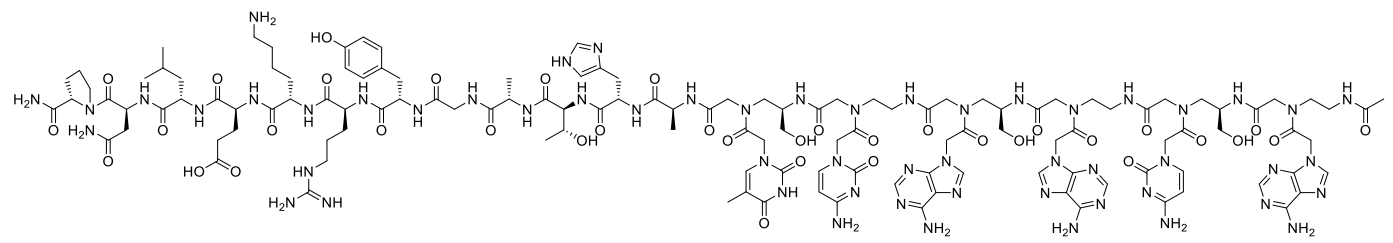	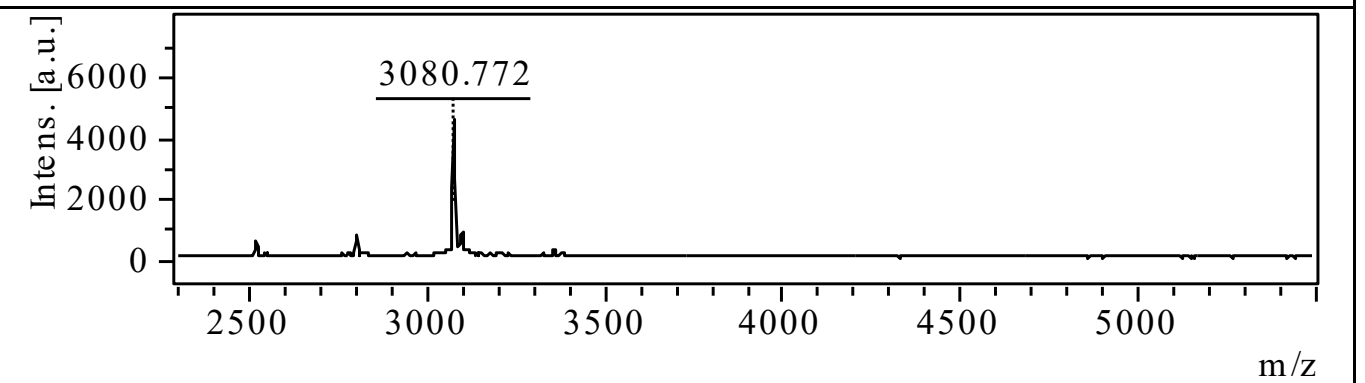

***Supplementary Table S5**: Exosite II Library Members*

	Chemical Formula	MW	m/z	Structure	MALDI
**Ex2-1**	$C_{142}H_{187}F_4N_{53}O_{53}S_2$	3624.49	3623.298	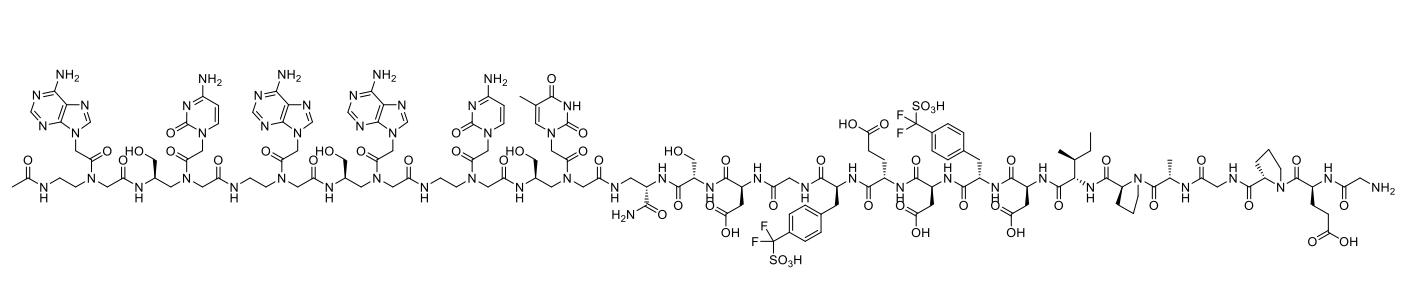	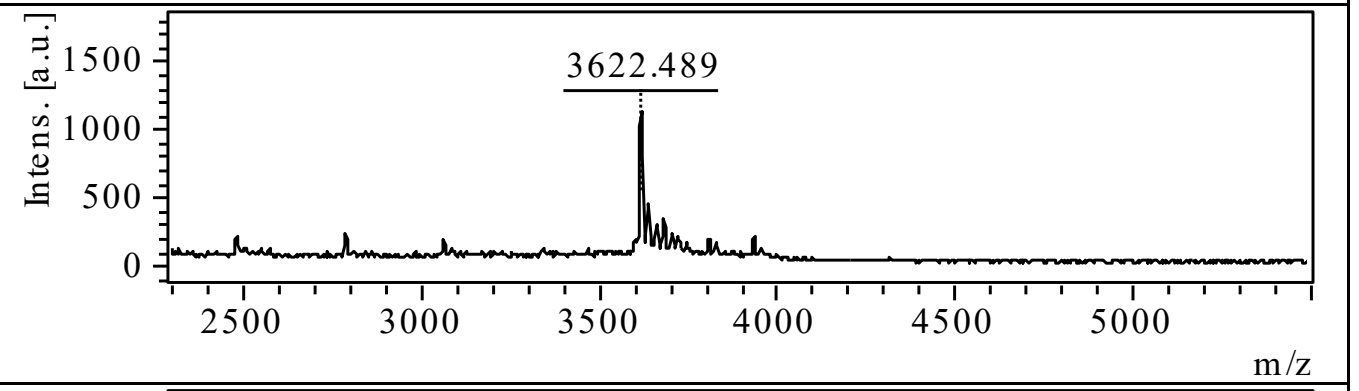
**Ex2-2**	$C_{121}H_{152}F_4N_{46}O_{48}S_2$	3158.935	3158.028	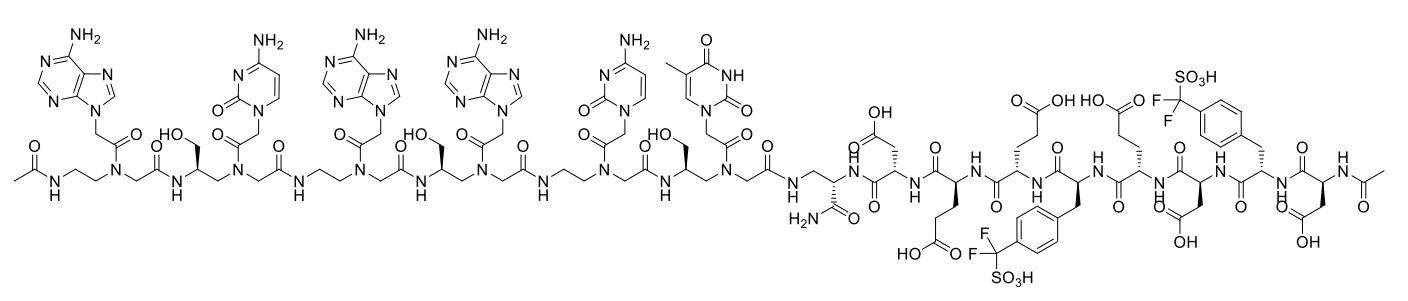	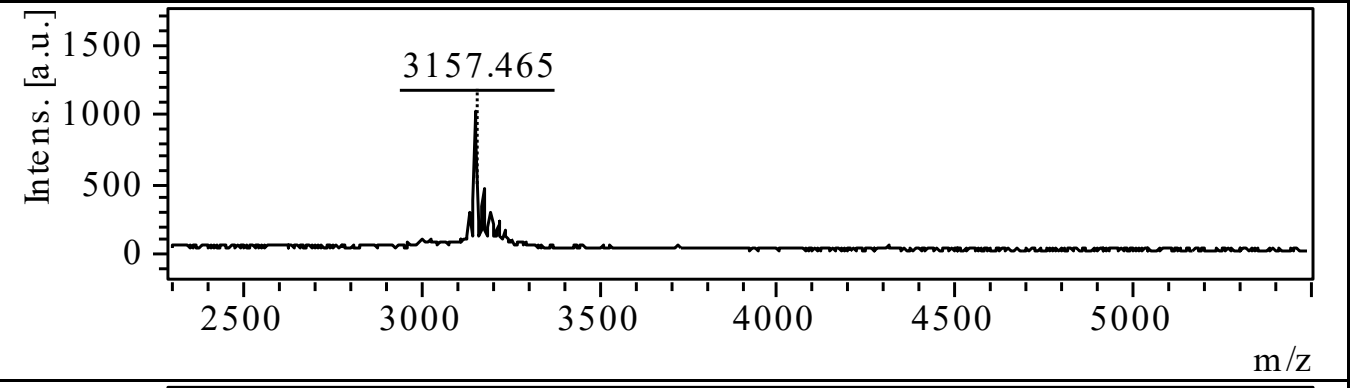
**Ex2-3**	$C_{142}H_{203}N_{57}O_{43}$	3396.542	3395.548	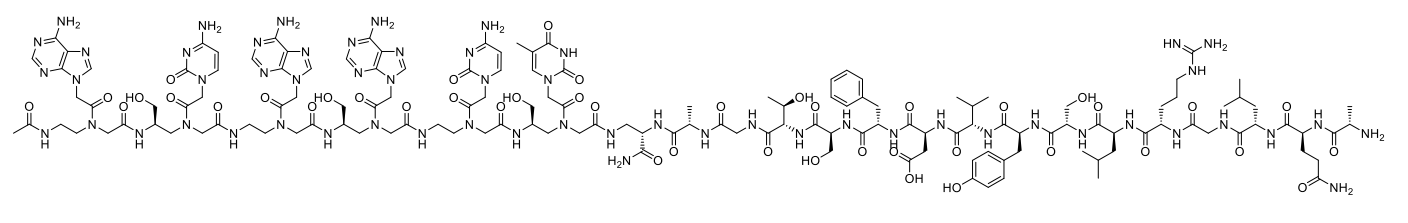	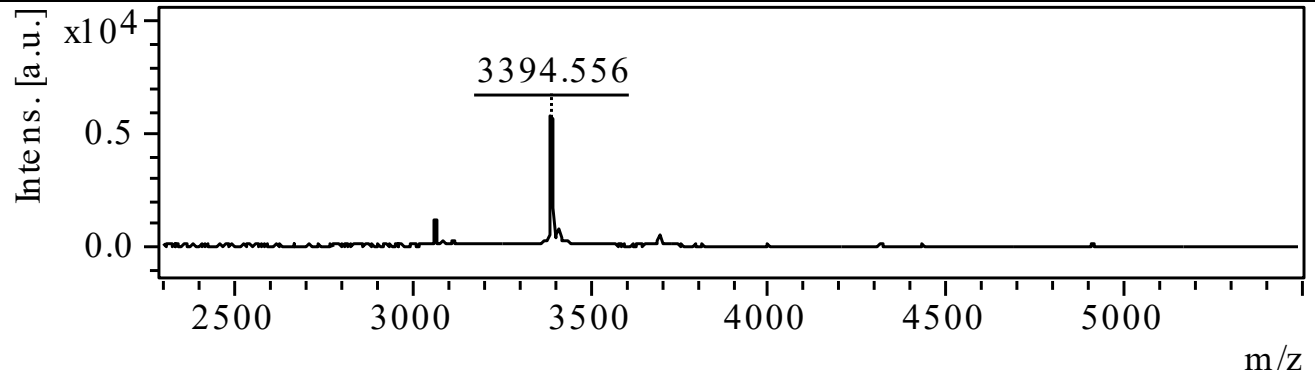
**Ex2-4**	$C_{132}H_{178}N_{50}O_{33}$	2993.193	2992.382	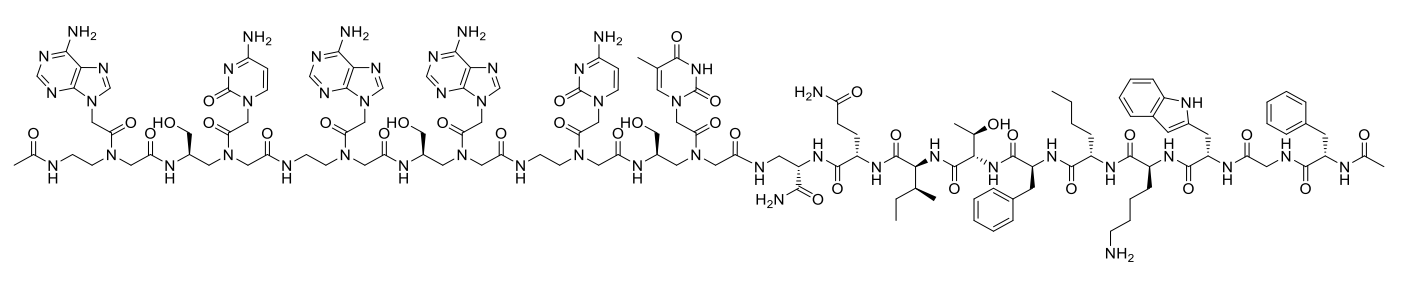	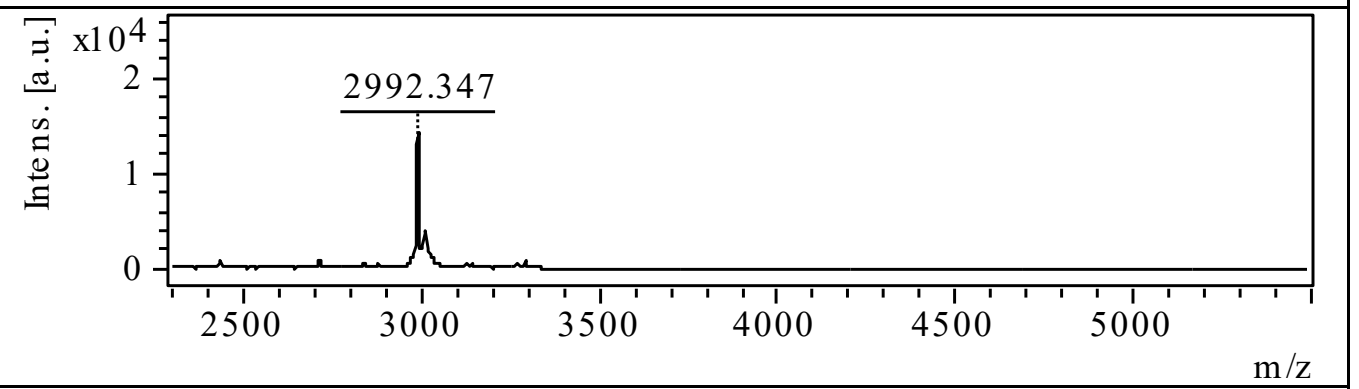
**Ex2-5**	$C_{141}H_{199}N_{55}O_{44}$	3368.484	3367.506	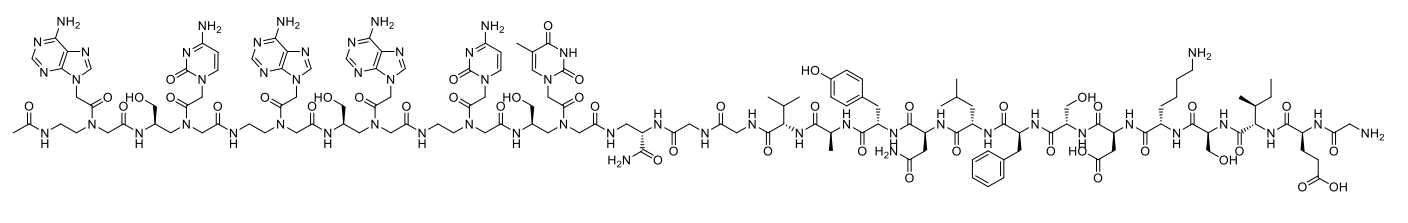	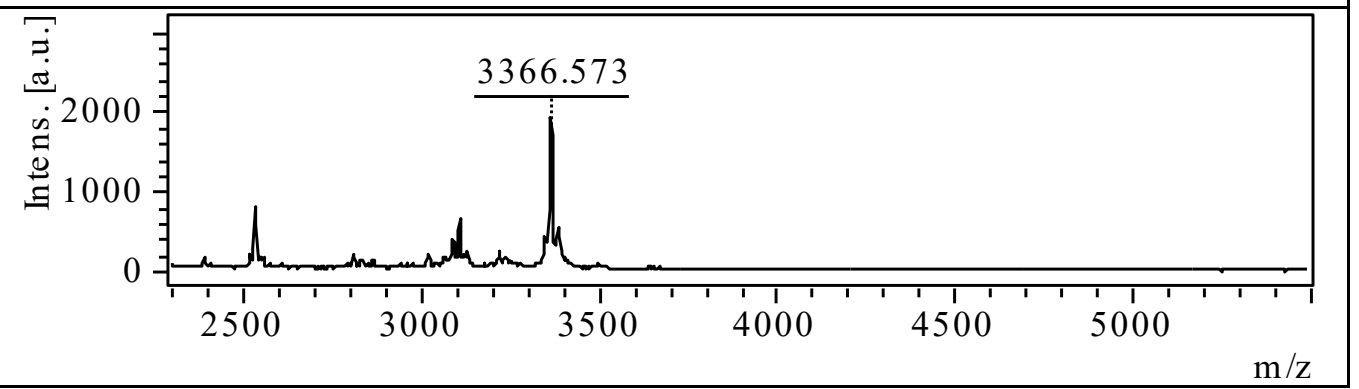

**Ex2-6**	$C_{145}H_{203}N_{59}O_{45}$	3492.587	3491.544	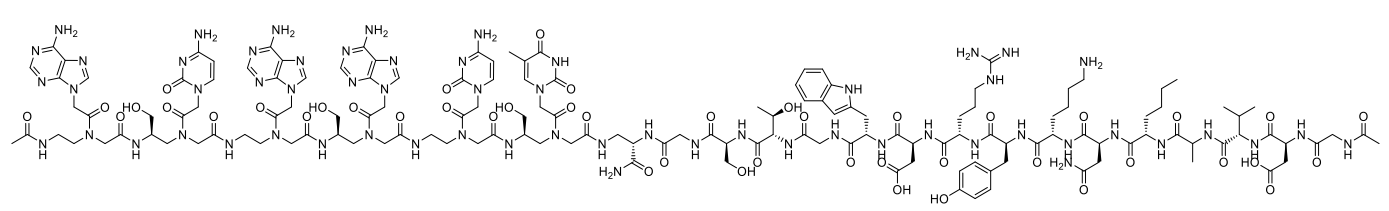	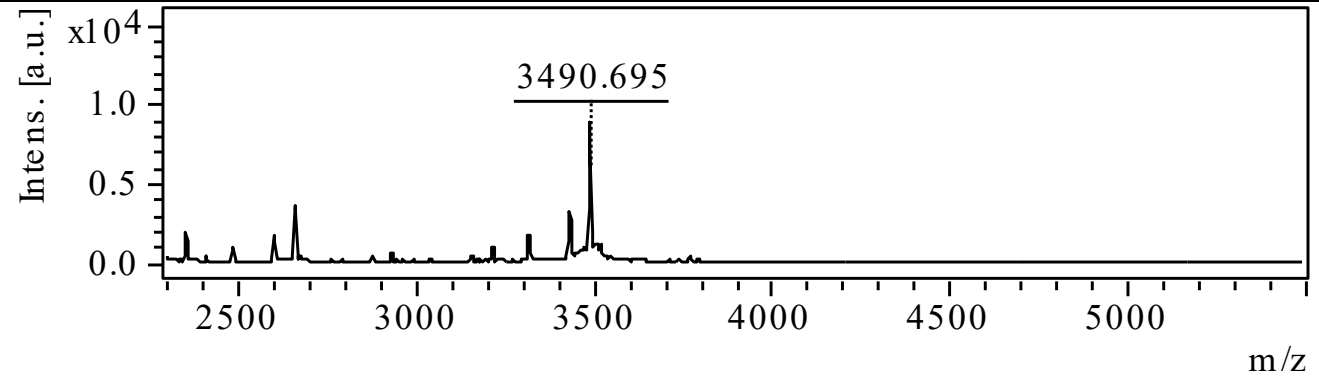
**Ex2-7**	$C_{137}H_{200}N_{58}O_{39}$	3283.474	3282.548	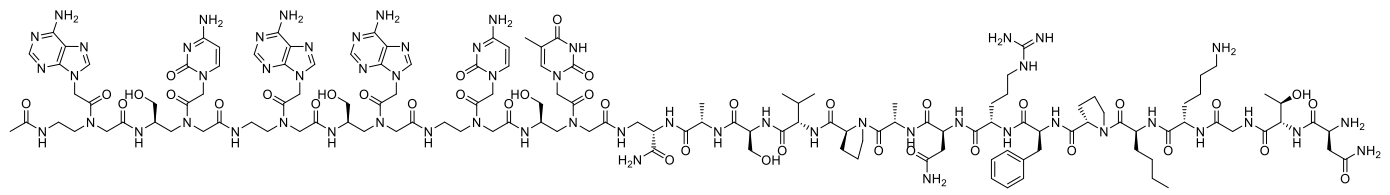	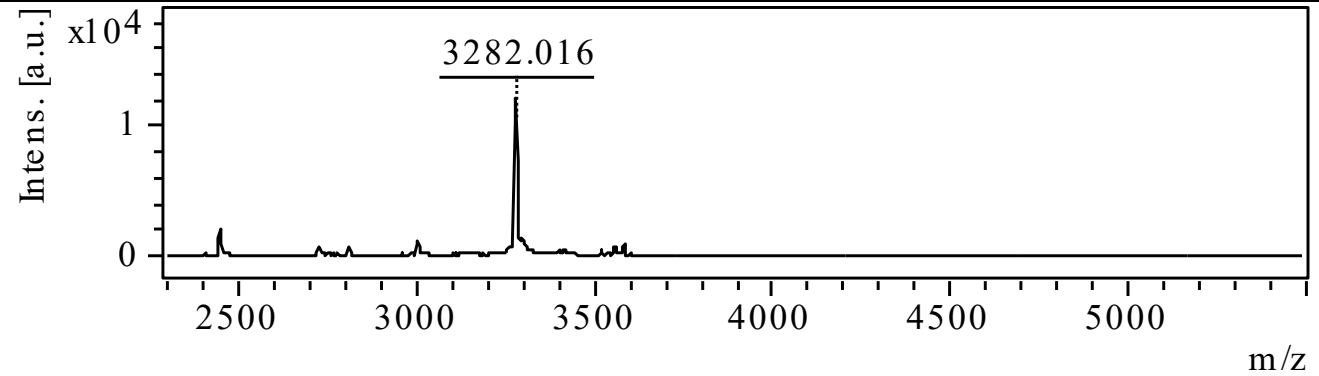
**Ex2-8**	$C_{130}H_{170}N_{52}O_{37}$	3053.17	3052.305	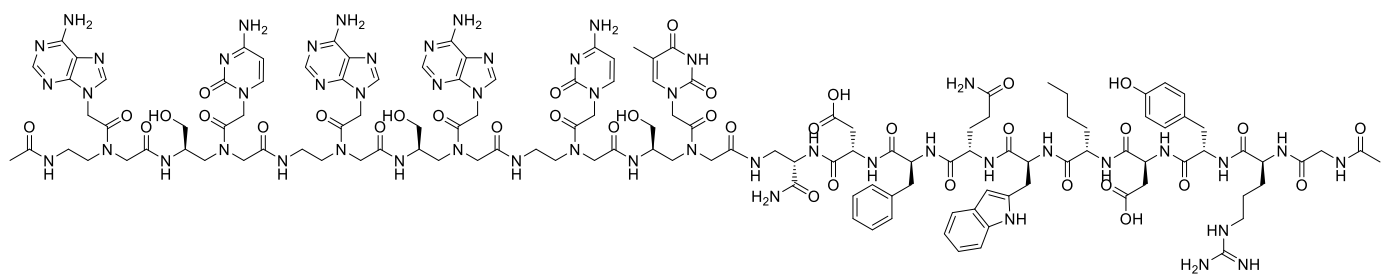	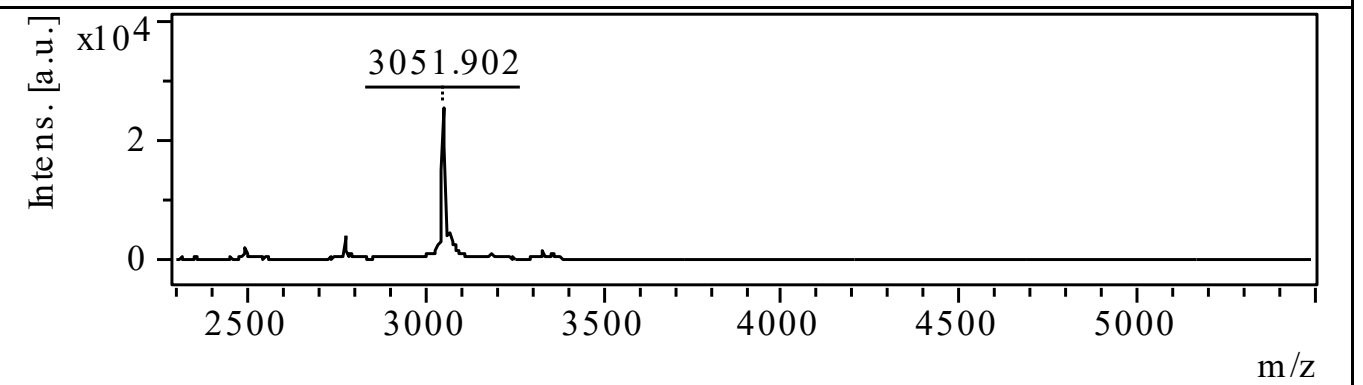
**Ex2-9**	$C_{131}H_{184}N_{54}O_{38}$	3123.263	3122.416	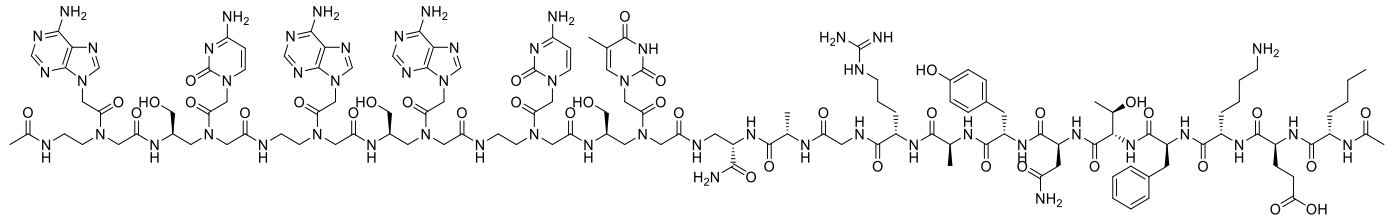	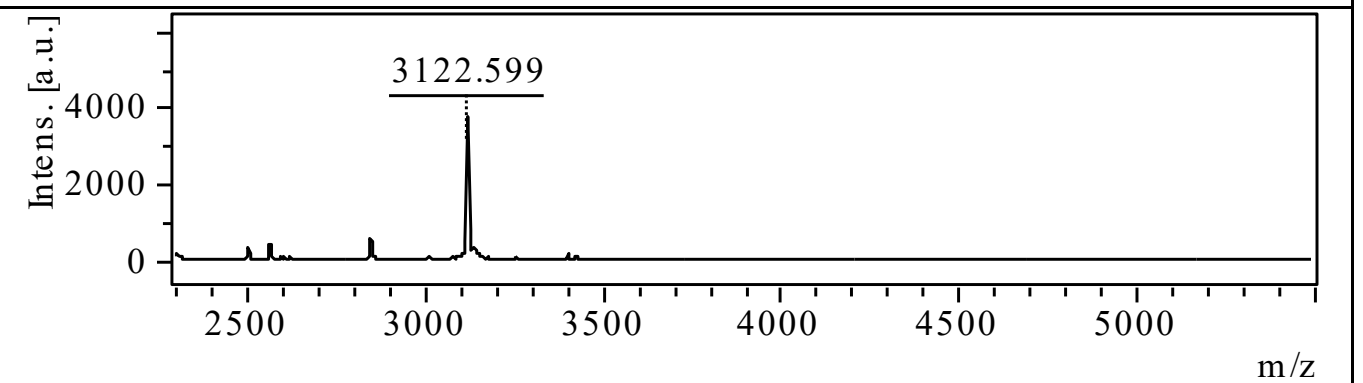
**Ex2-10**	$C_{149}H_{218}N_{58}O_{41}$	3477.748	3476.679	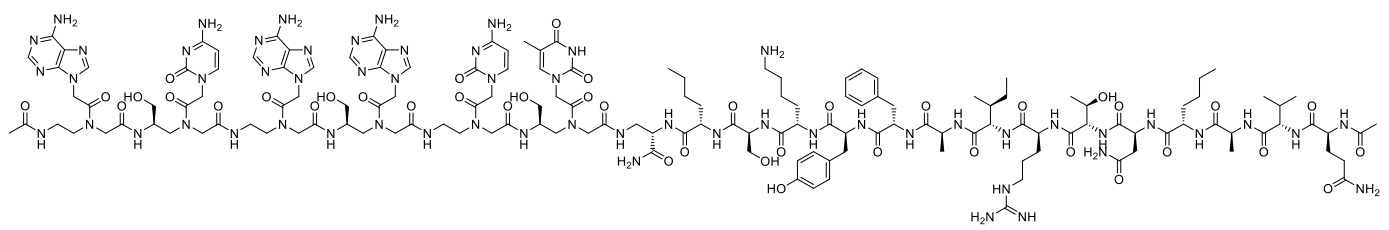	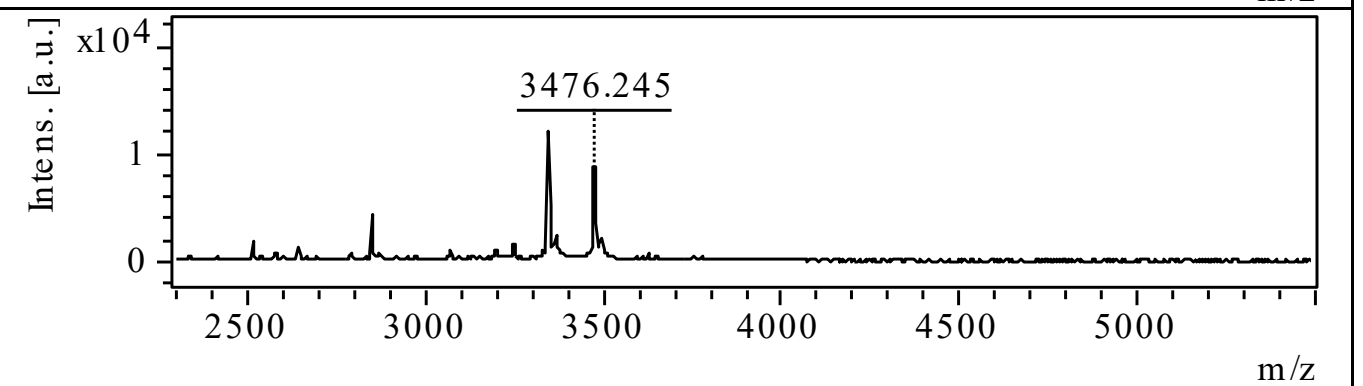
**Ex2-11**	$C_{147}H_{195}N_{55}O_{53}$	3580.509	3579.429	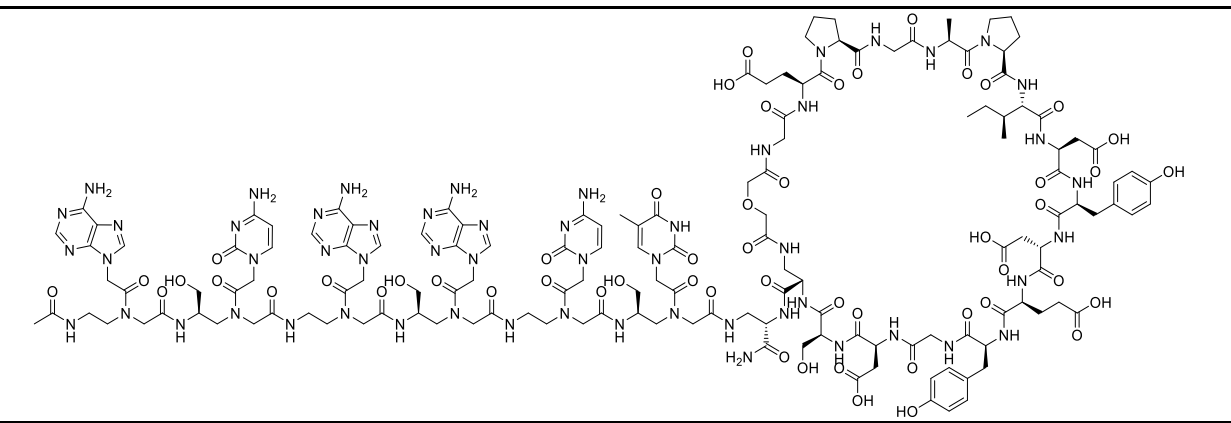	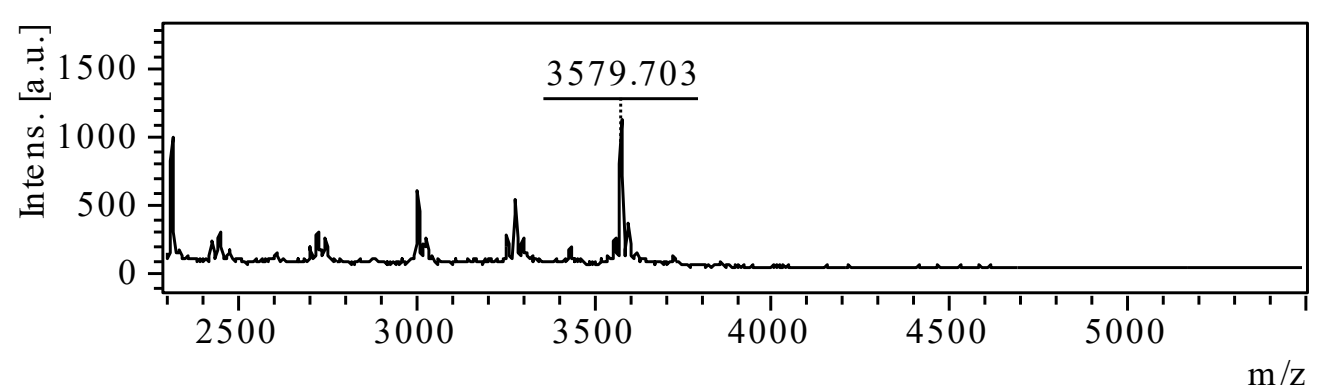

**Ex2-12**	$C_{153}H_{206}N_{56}O_{56}$	3725.667	3724.503	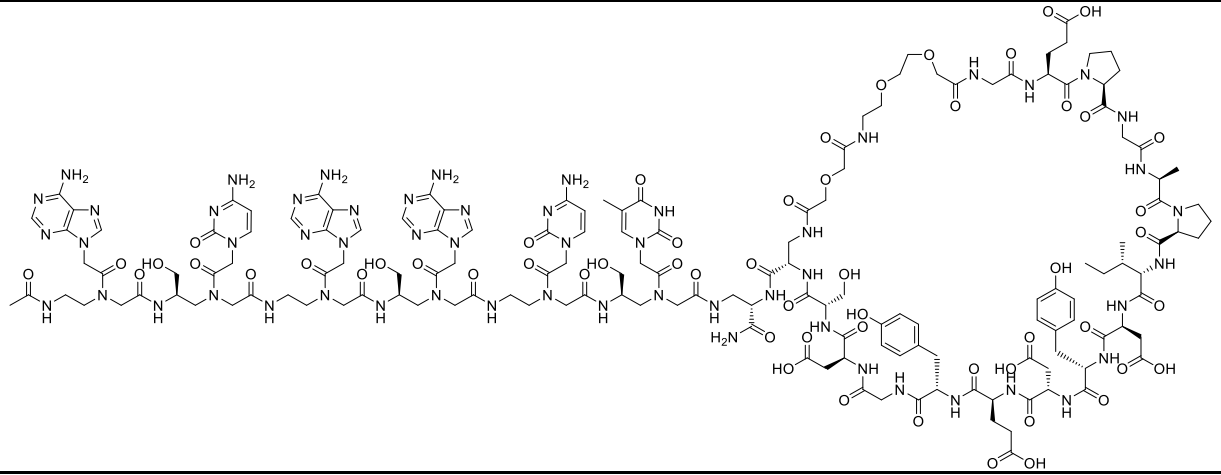	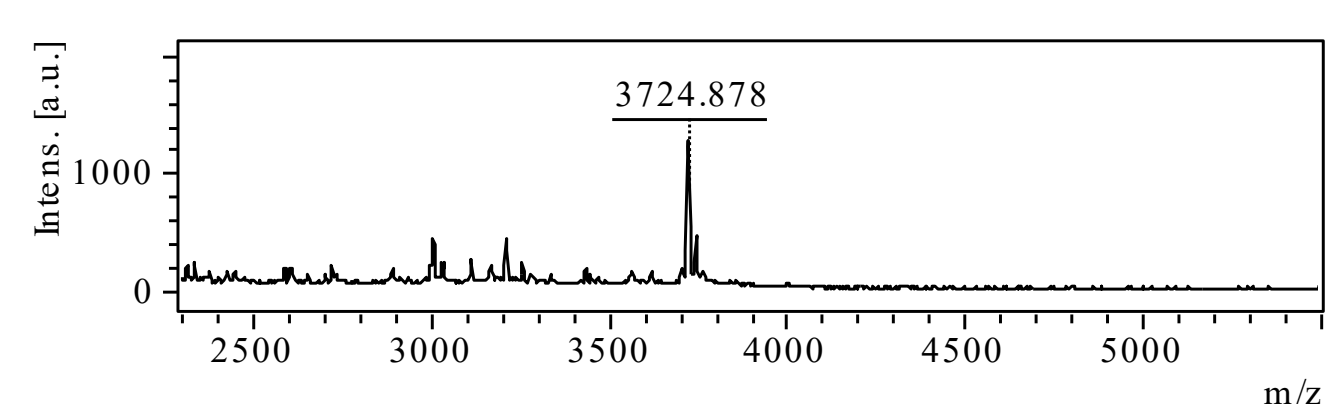
**Ex2-13**	$C_{144}H_{187}F_4N_{53}O_{60}S_2$	3760.505	3759.262	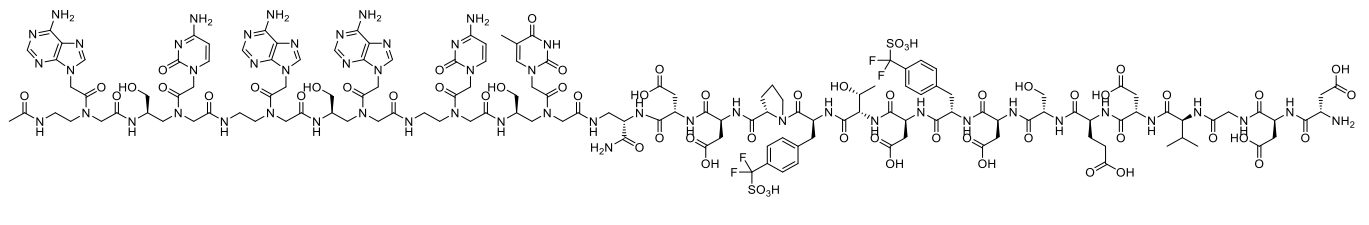	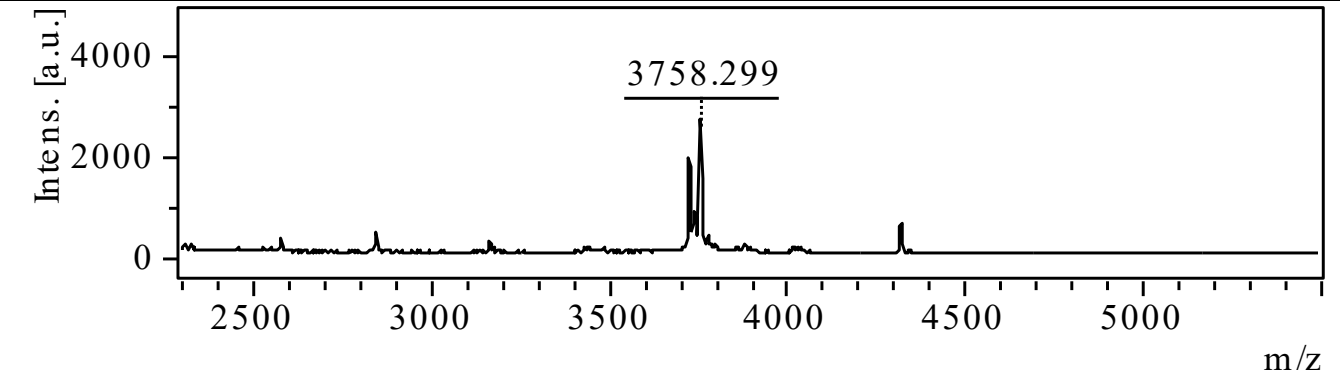
**Ex2-14**	$C_{148}H_{193}F_4N_{53}O_{59}S_2$	3798.598	3797.314	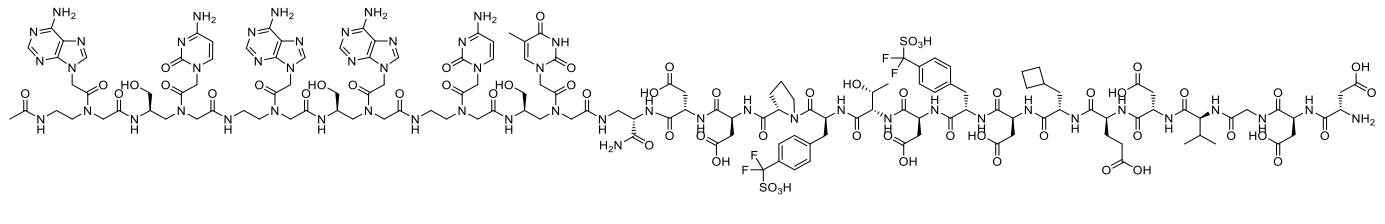	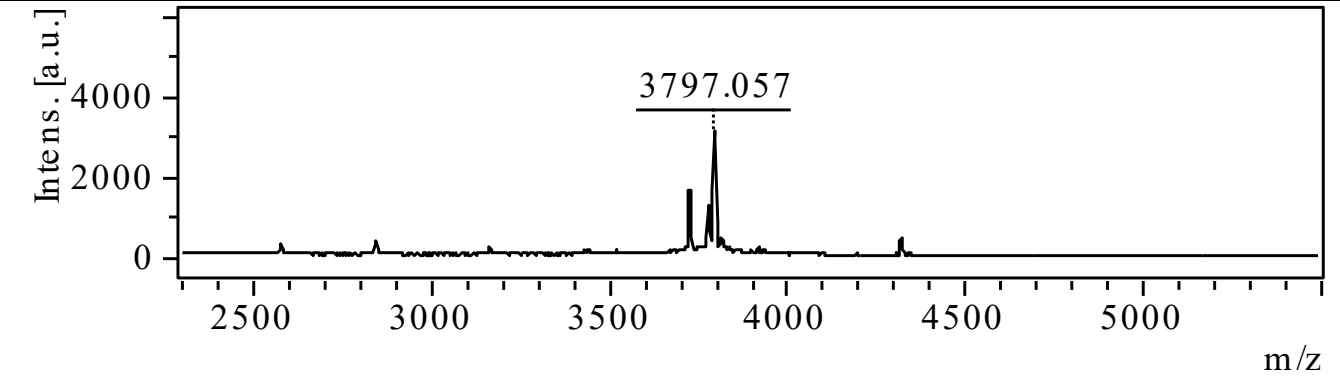
**Ex2-15**	$C_{122}H_{162}N_{52}O_{40}S$	3029.022	3028.200	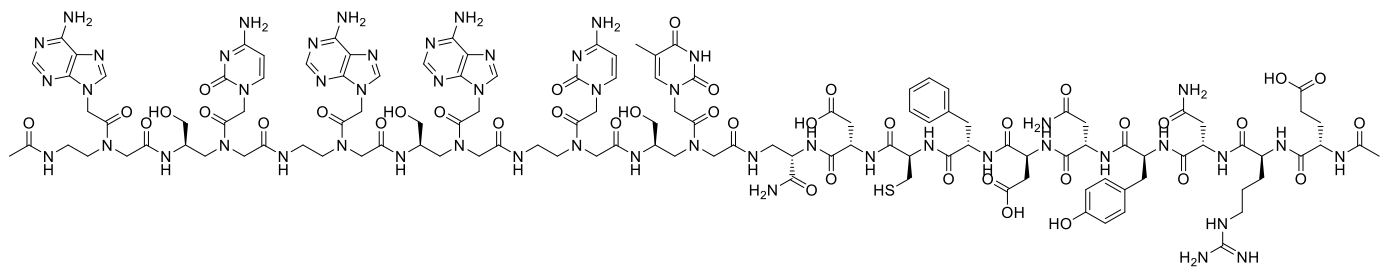	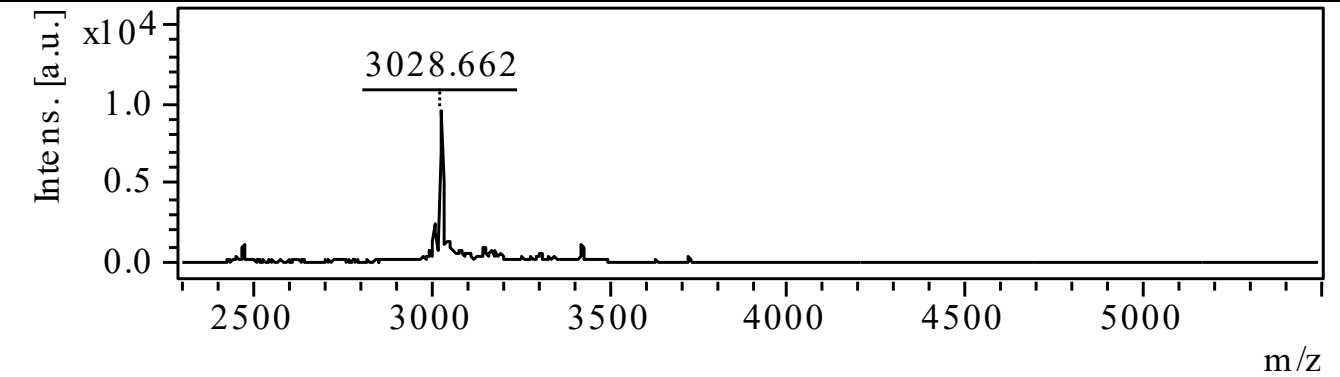
**Ex2-16**	C135H184 N58O40	3259.323	3258.418	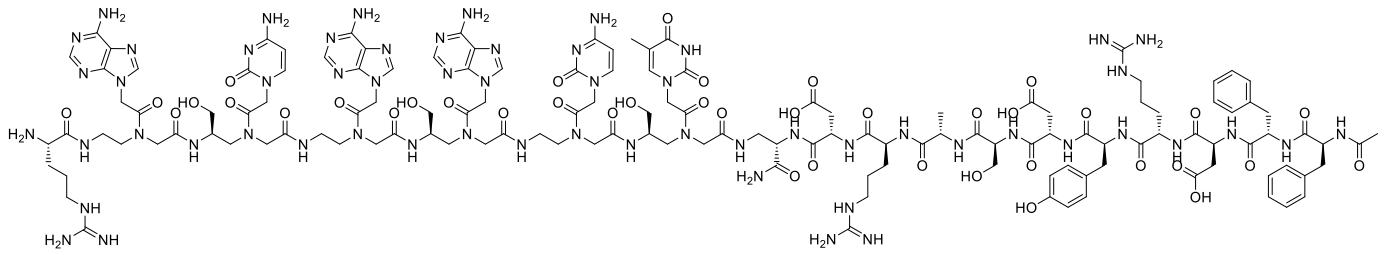	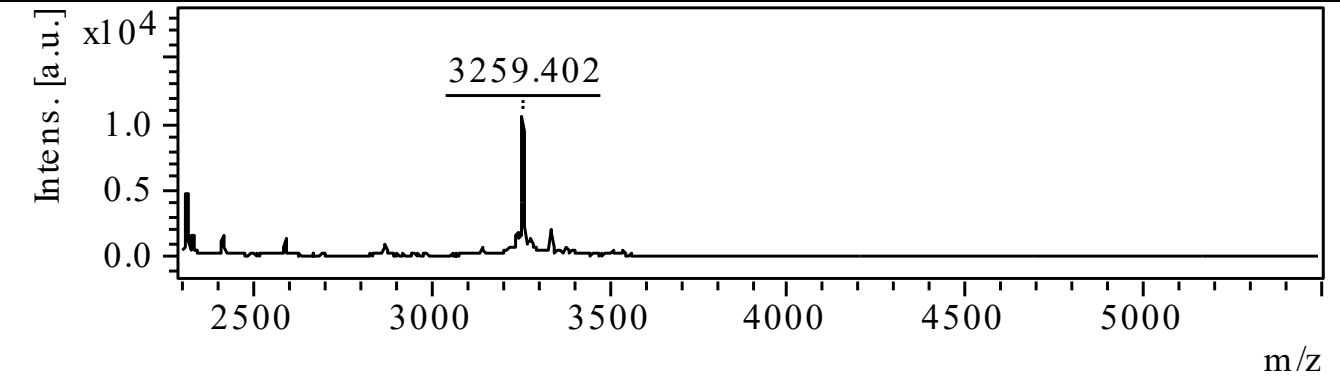
**Ex2-17**	$C_{172}H_{218}N_{62}O_{46}$	3889.036	3888.666	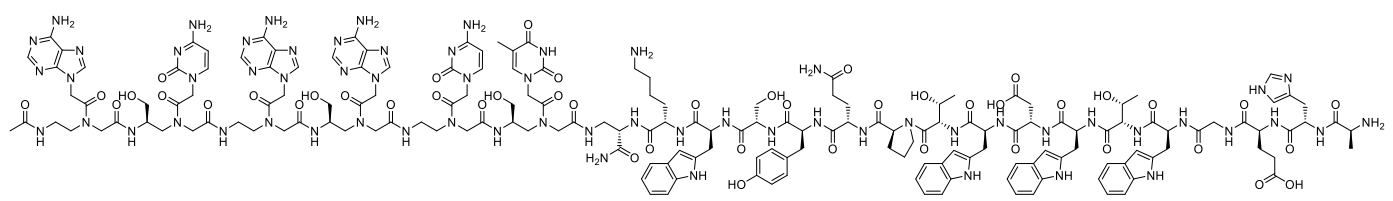	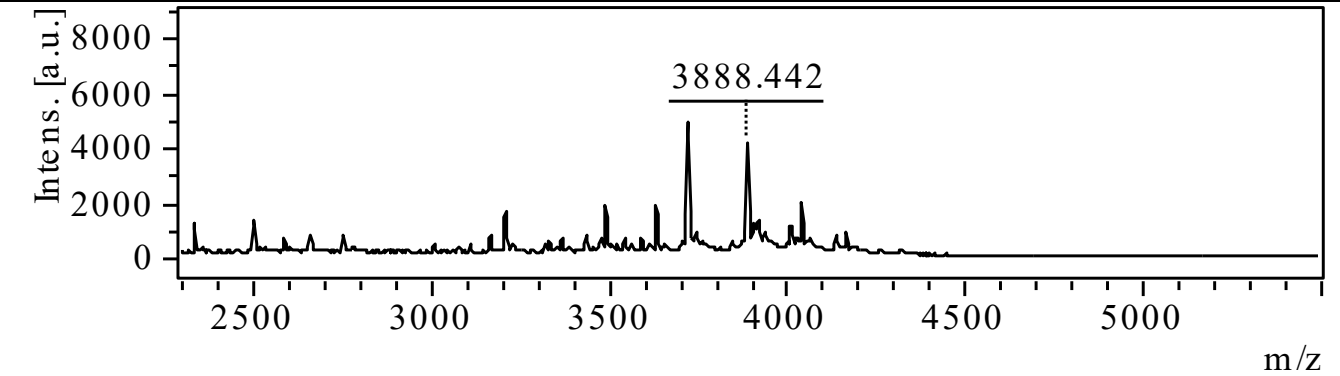

**Ex2-18**	$C_{156}H_{211}N_{61}O_{45}$	3660.786	3659.613	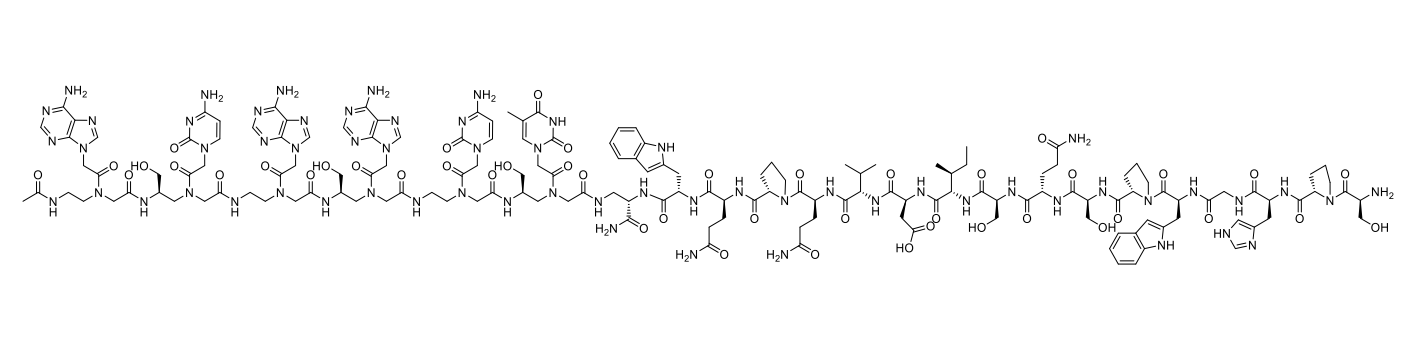	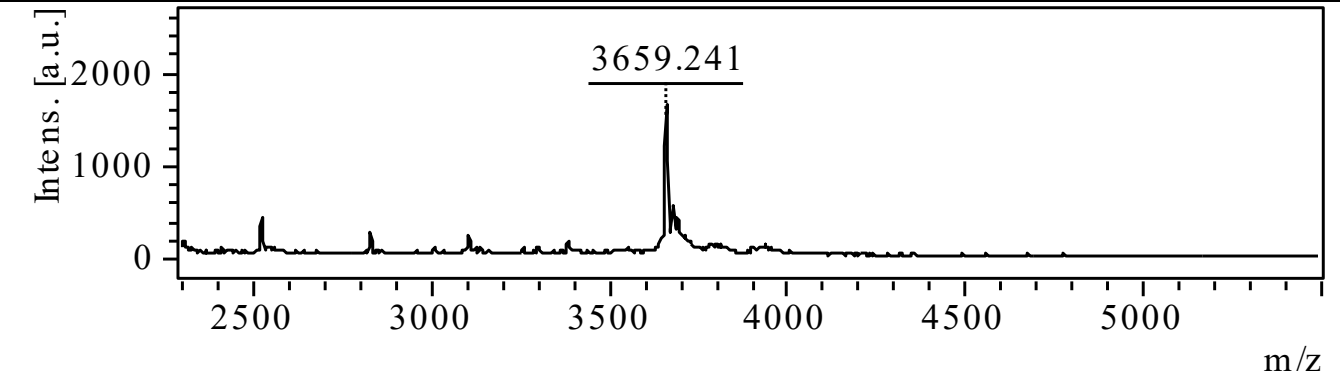
**Ex2-19**	$C_{159}H_{216}N_{64}O_{45}$	3743.88	3742.661	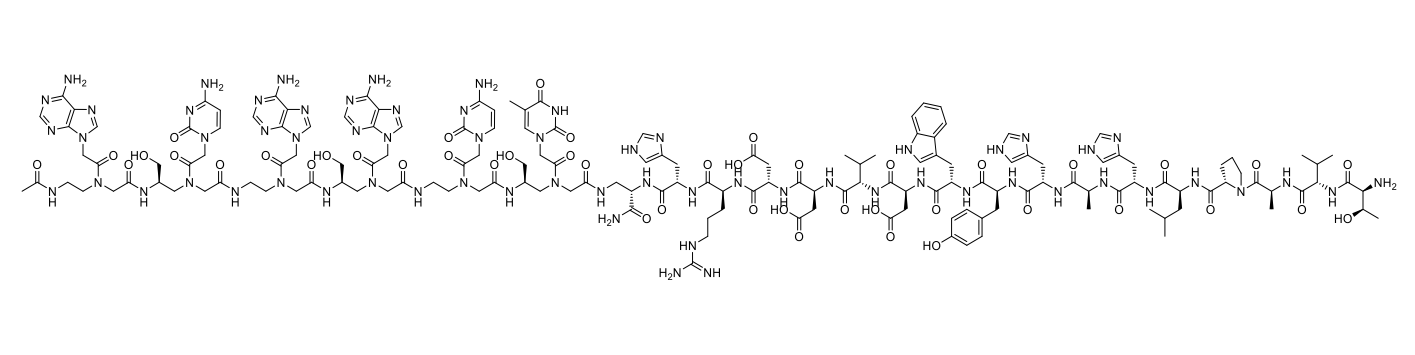	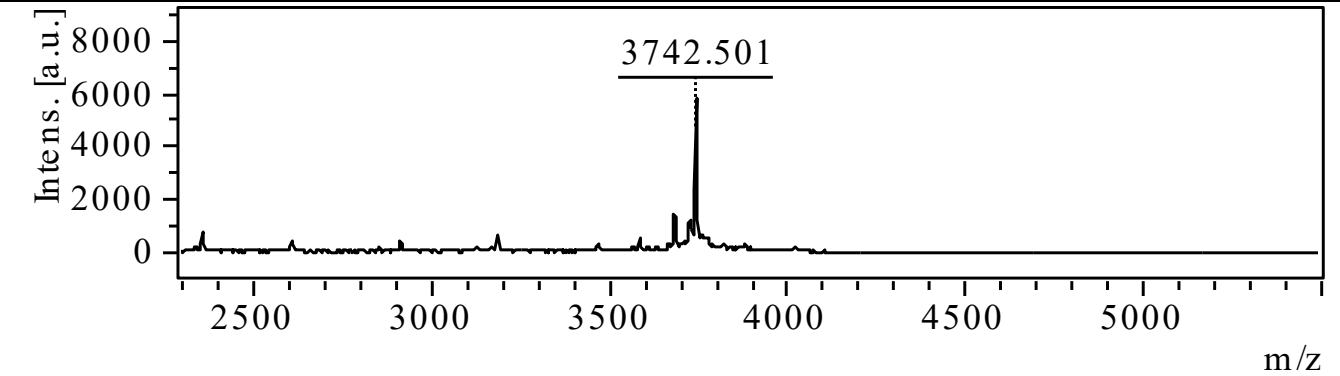
**Ex2-20**	$C_{140}H_{188}N_{52}O_{49}$	3383.359	3382.385	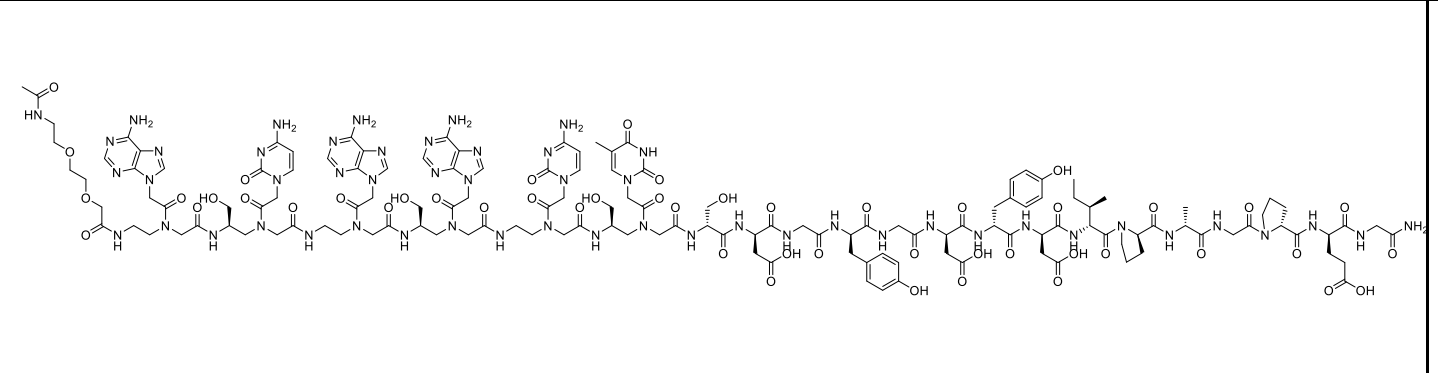	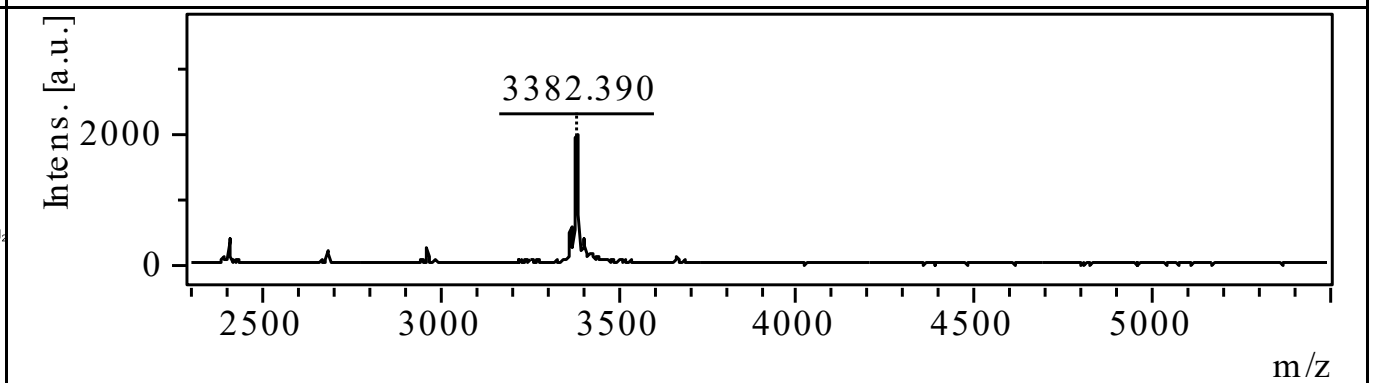
**Ex2-21**	$C_{114}H_{144}N_{44}O_{42}$	2802.672	2802.052	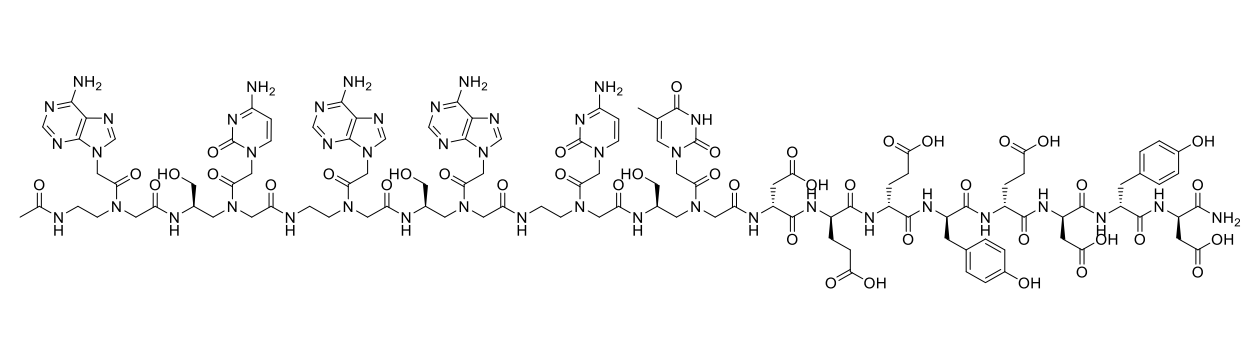	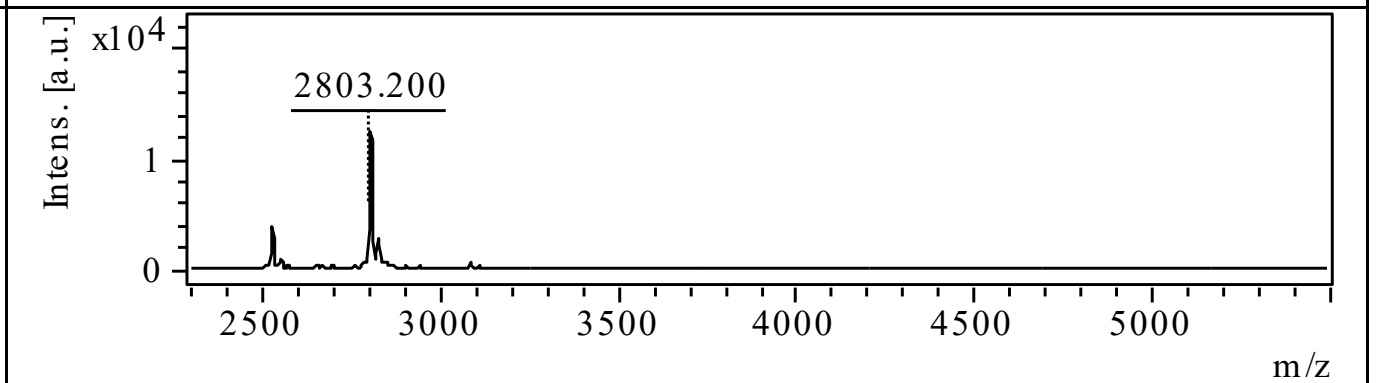
**Ex2-22**	$C_{154}H_{243}N_{67}O_{46}$	3769.061	3767.877	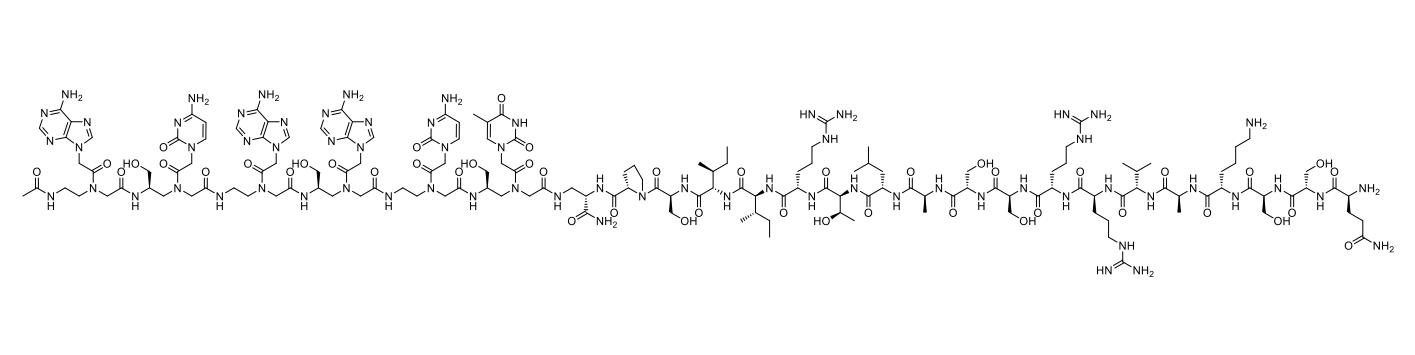	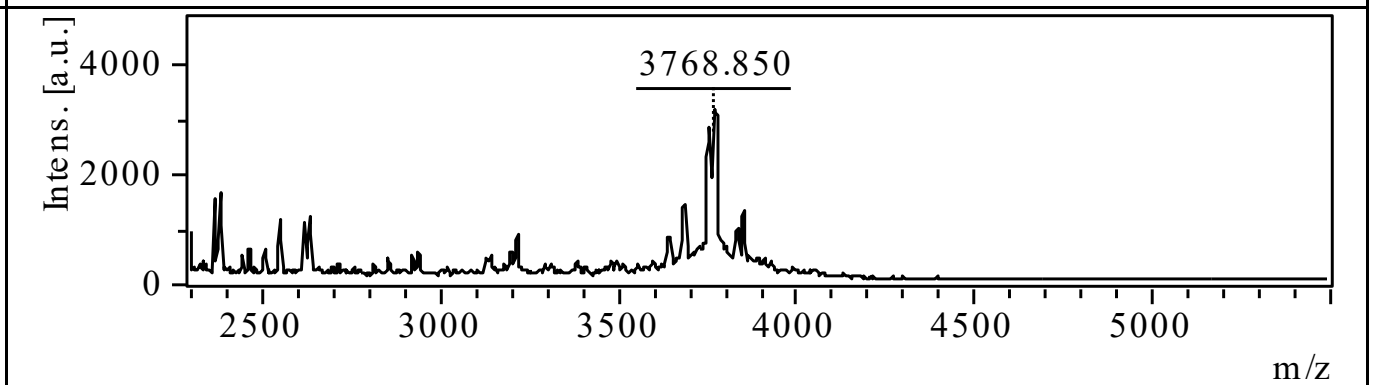
**Ex2-23**	$C_{111}H_{165}N_{49}O_{31}$	2681.853	2681.287	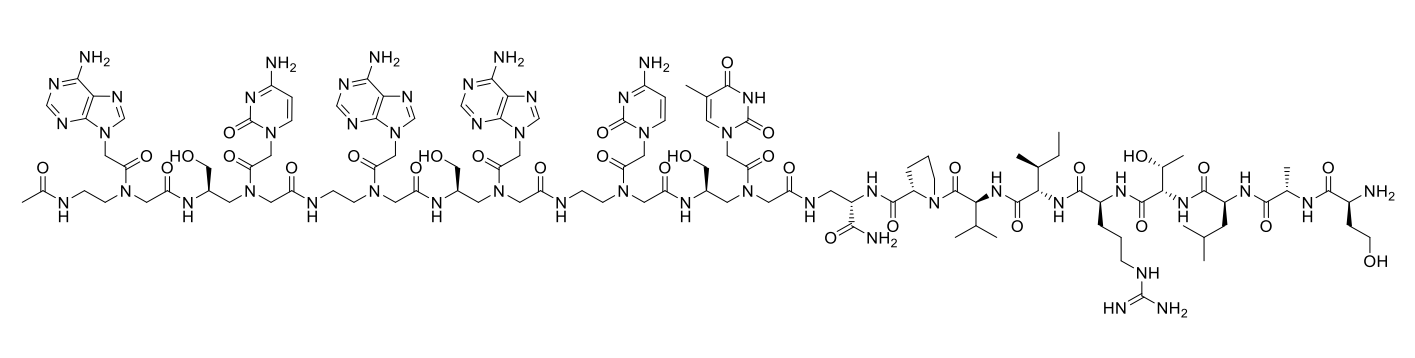	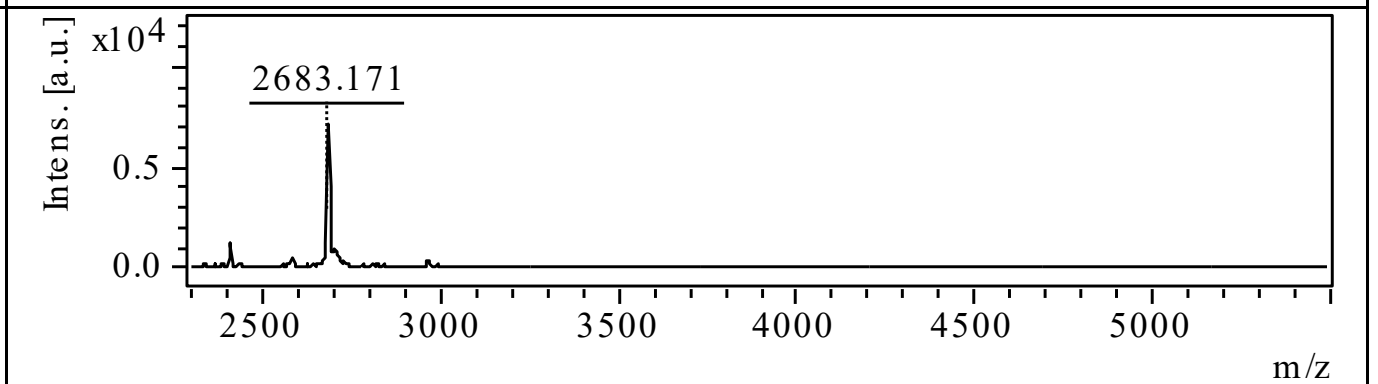

**Ex2-24**	$C_{114}H_{171}N_{51}O_{32}$	2767.947	2767.335	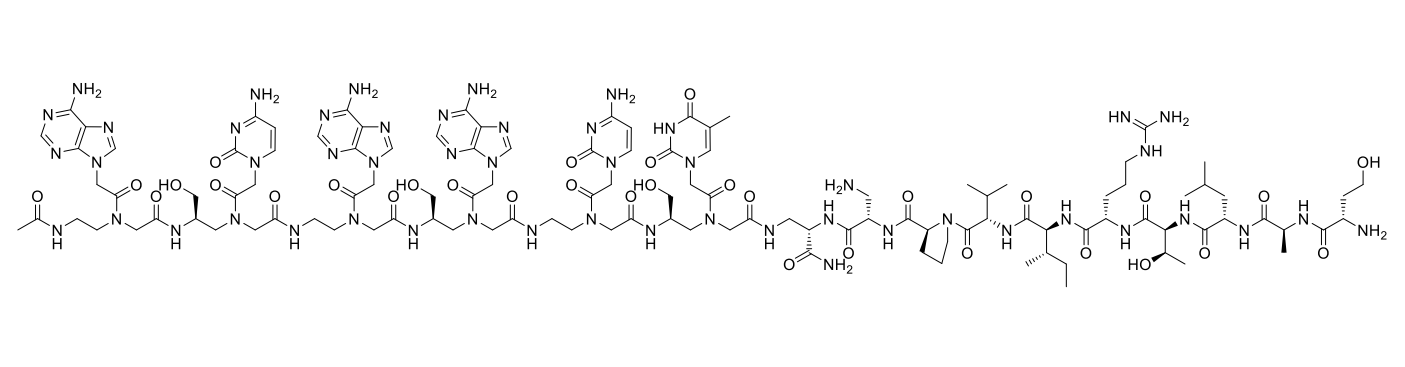	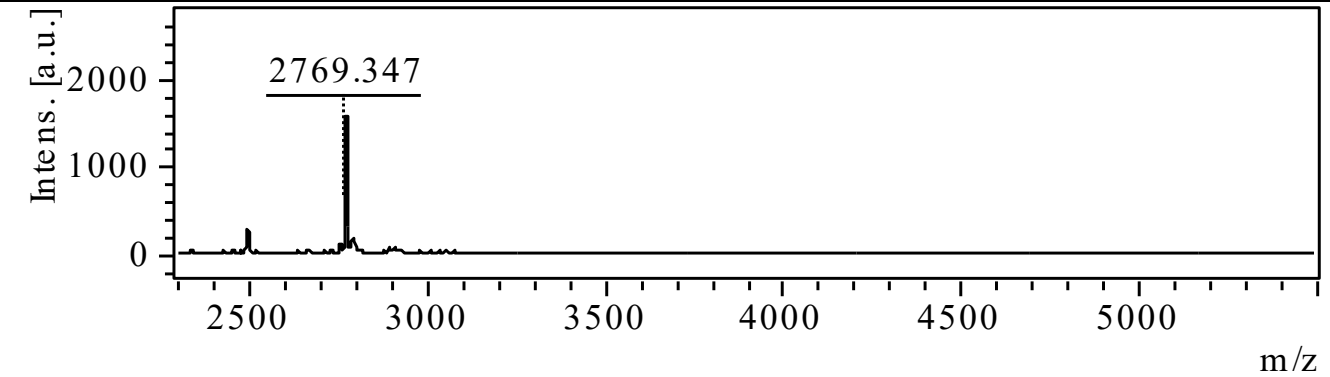
**Ex2-25**	$C_{124}H_{186}N_{56}O_{36}$	3037.208	3036.448	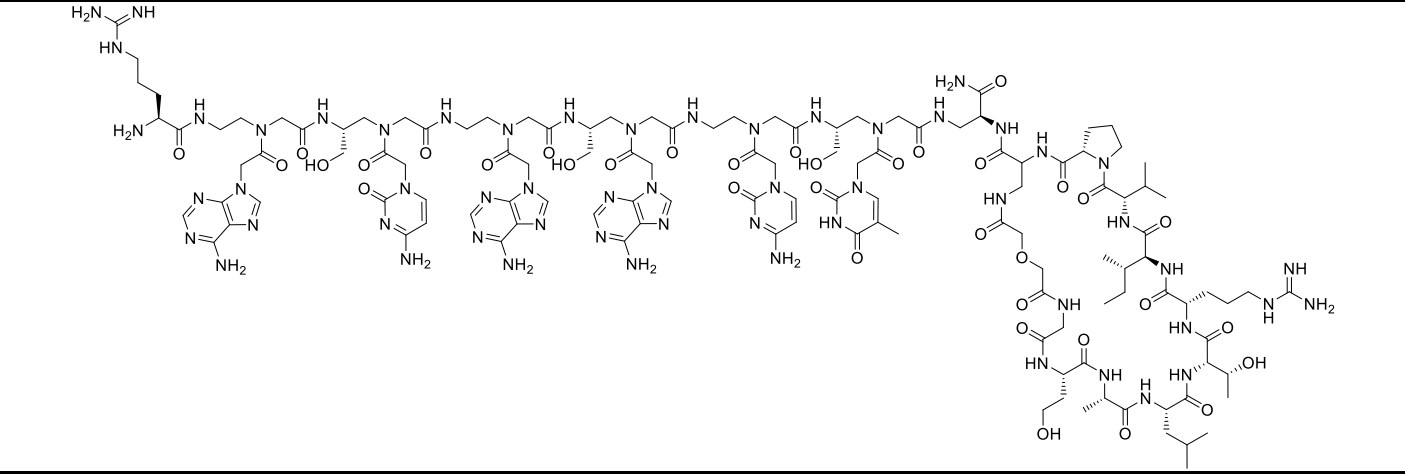	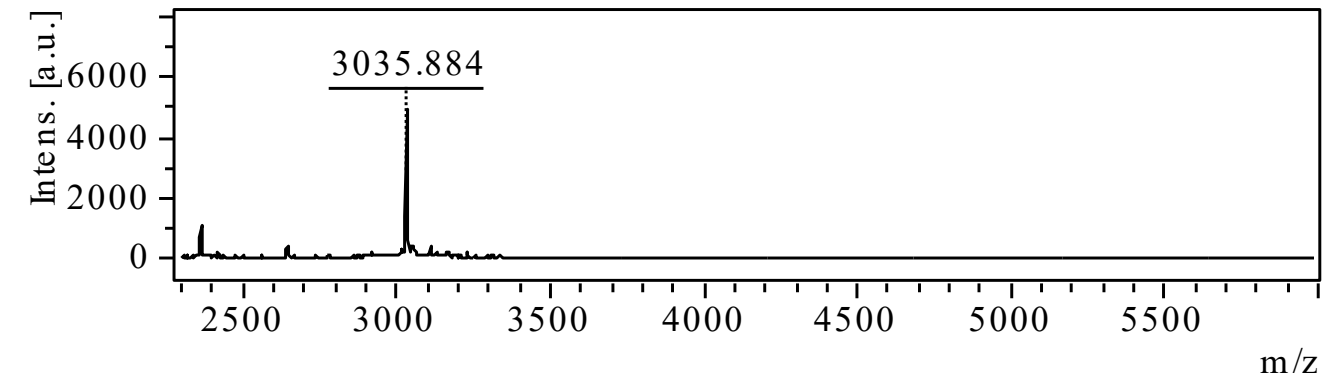
**Ex2-26**	$C_{168}H_{243}N_{65}O_{42}$	3845.205	3843.891	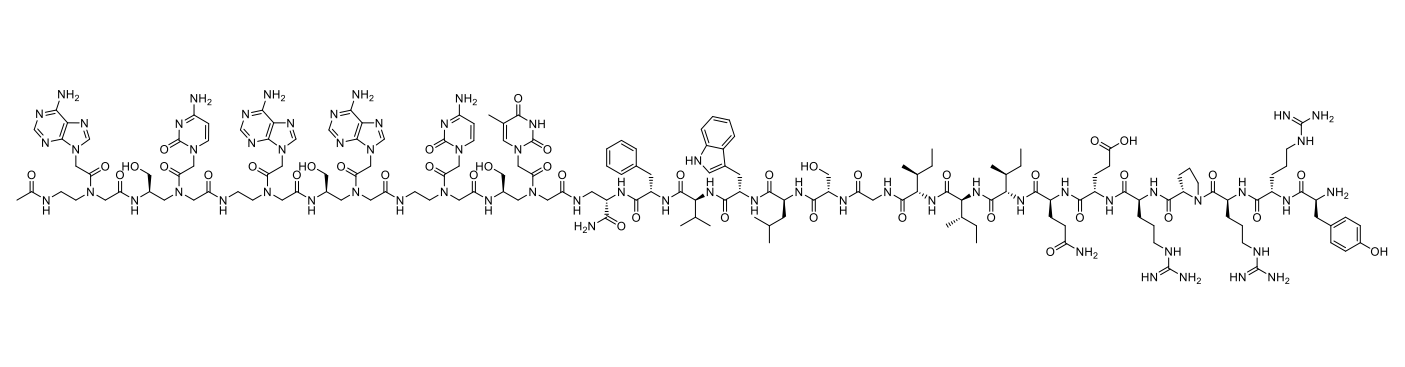	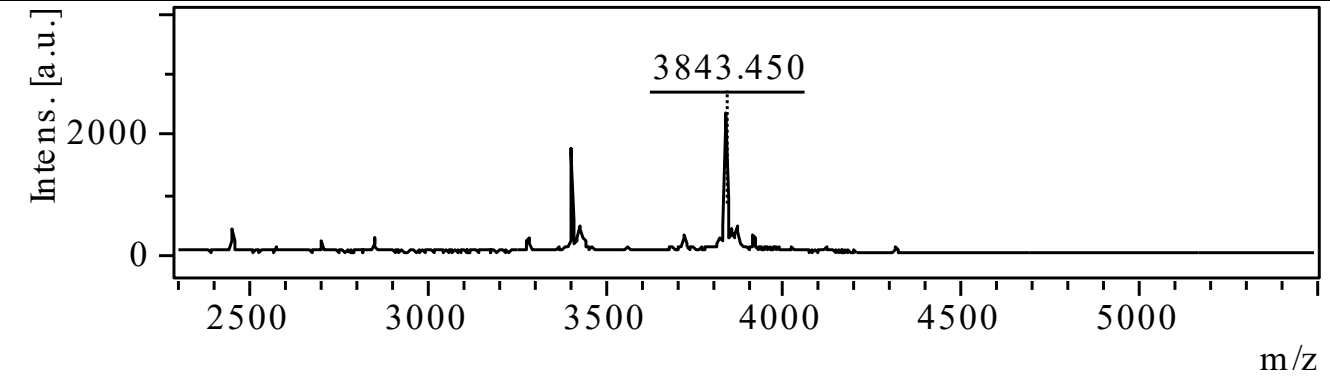
**Ex2-27**	$C_{132}H_{183}N_{53}O_{51}S_2$	3392.356	3391.283	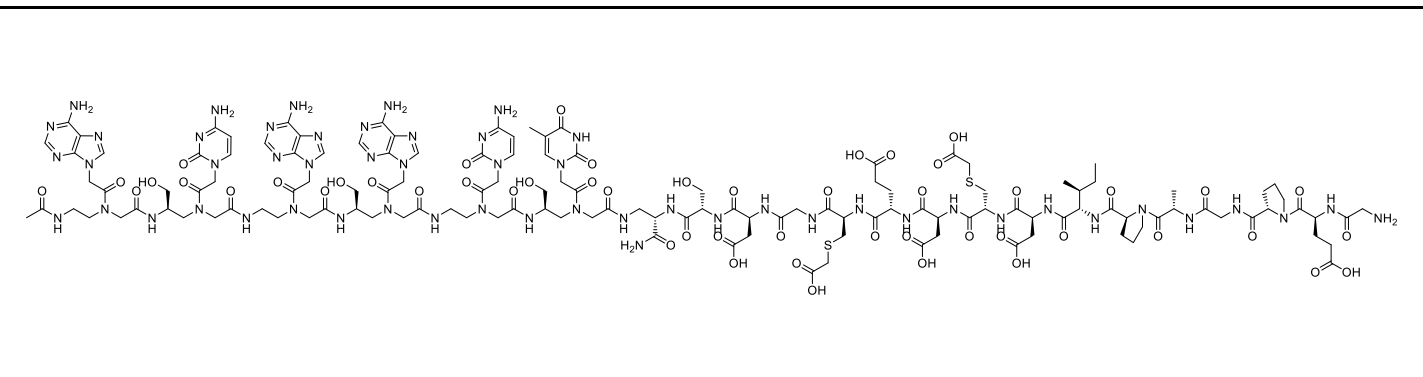	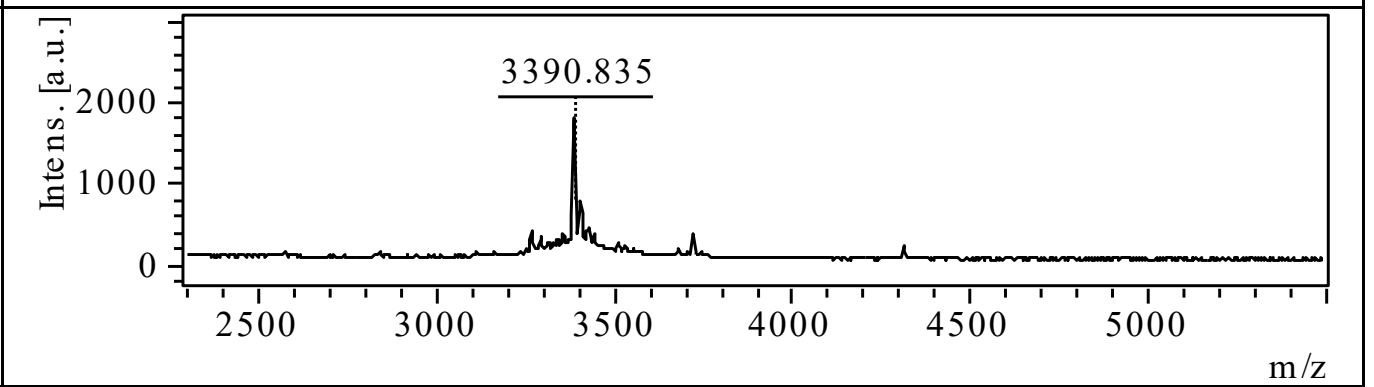
**Ex2-28**	$C_{150}H_{220}N_{64}O_{39}$	3543.819	3542.723	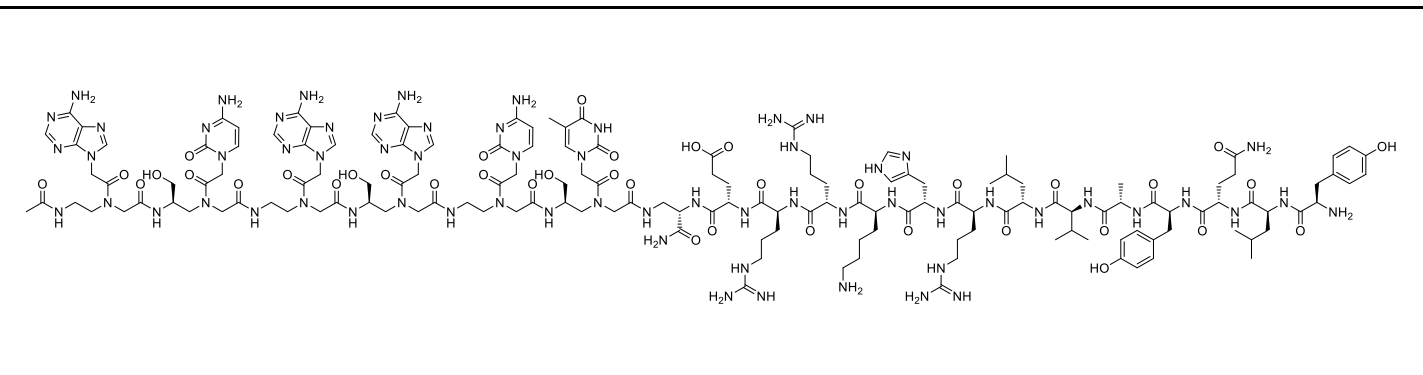	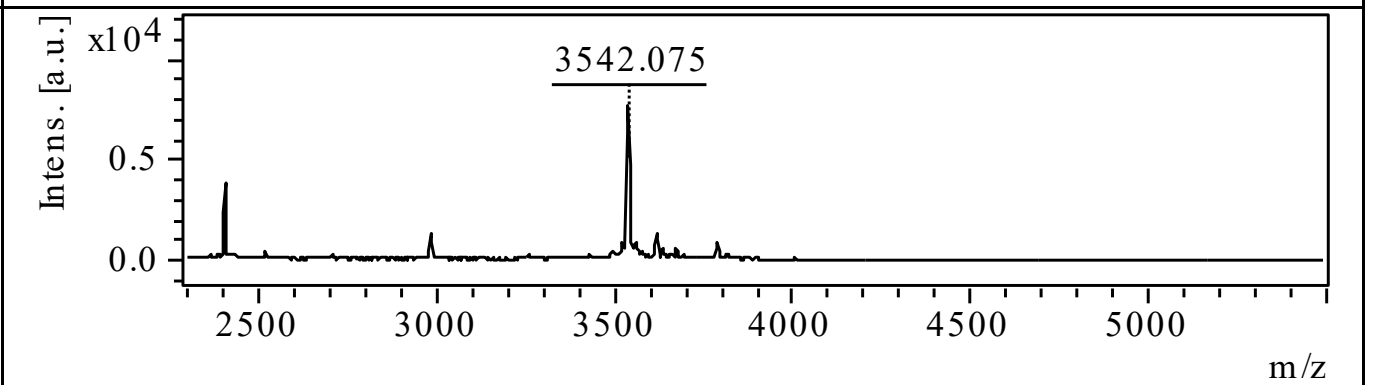

**Ex2-29**	$C_{157}H_{228}N_{66}O_{43}$	3727.97	3726.772	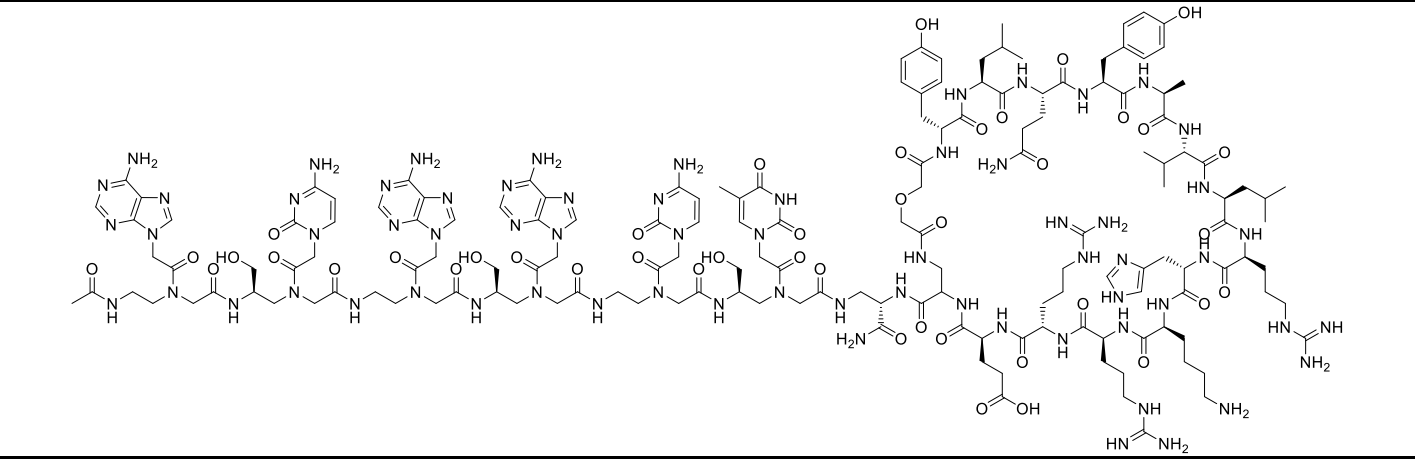	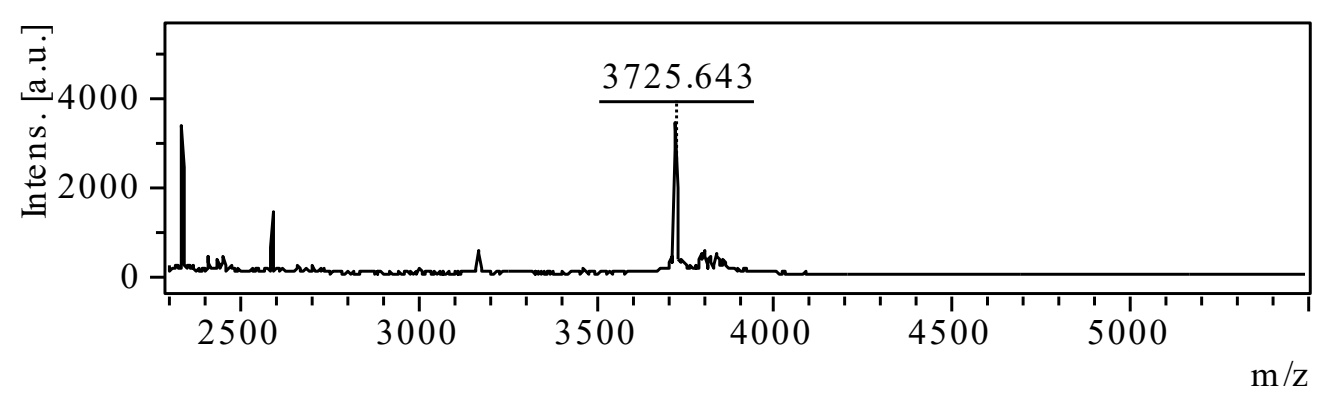
**Ex2-30**	$C_{176}H_{262}N_{62}O_{48}$	4014.418	4013.000	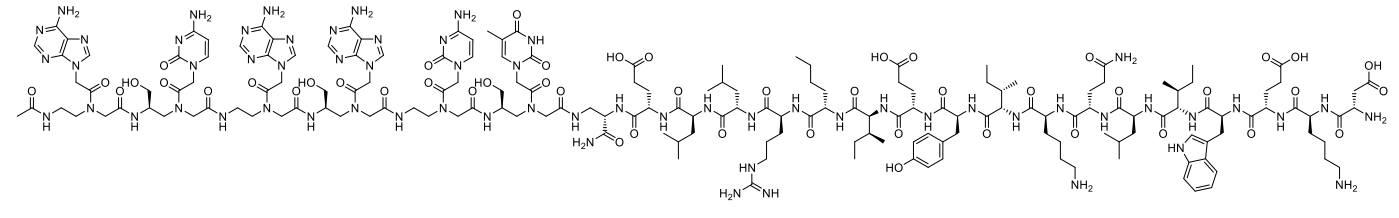	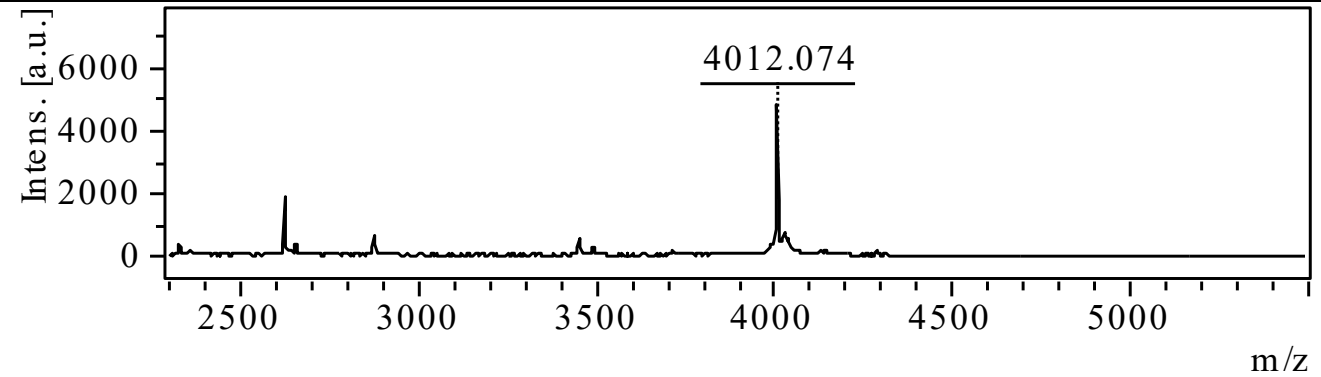
**Ex2-31**	$C_{160}H_{237}N_{59}O_{46}$	3723.023	3721.805	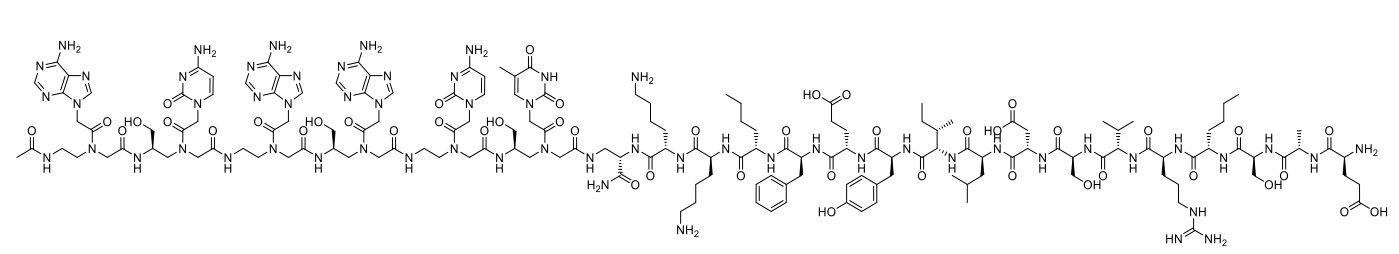	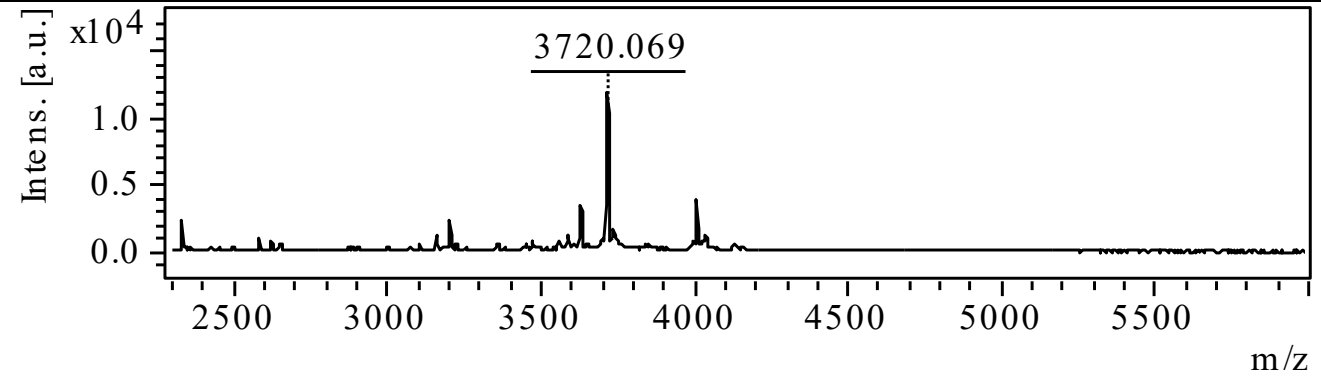
**Ex2-32**	C133H181 N53O43	3210.239	3209.364	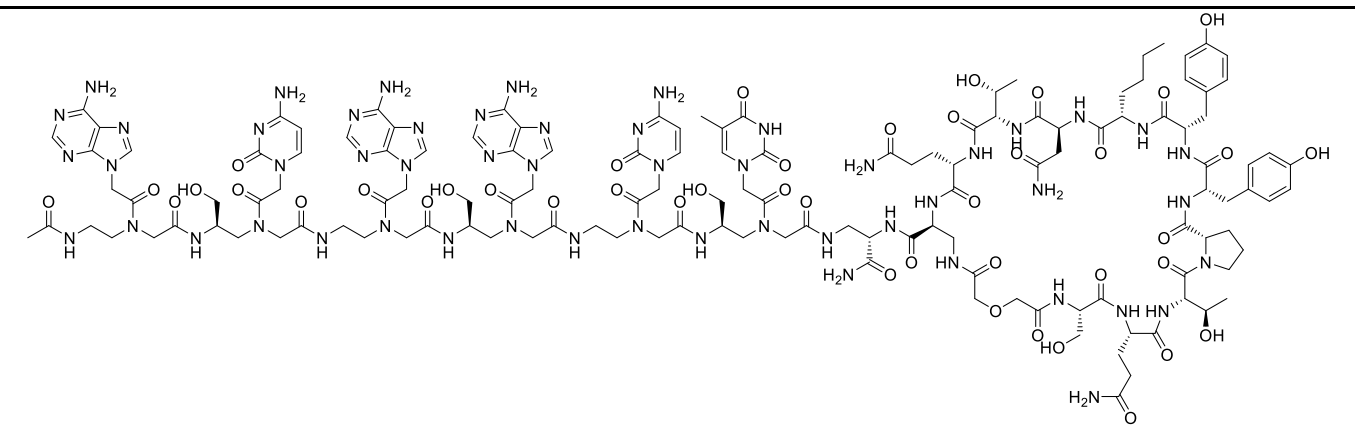	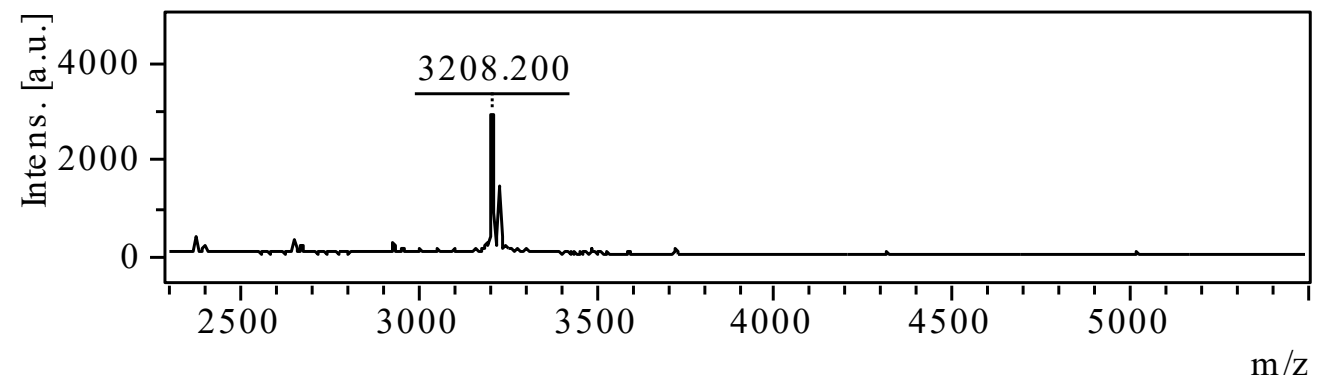
**Ex2-33**	$C_{150}H_{201}F_4N_{57}O_{60}S_2$	3902.711	3901.384	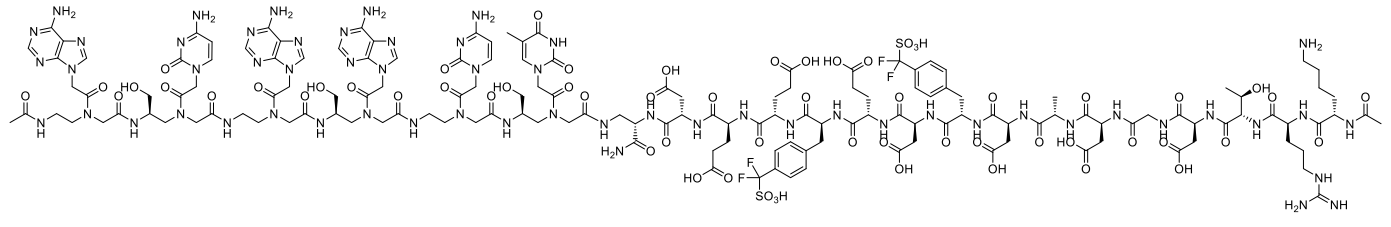	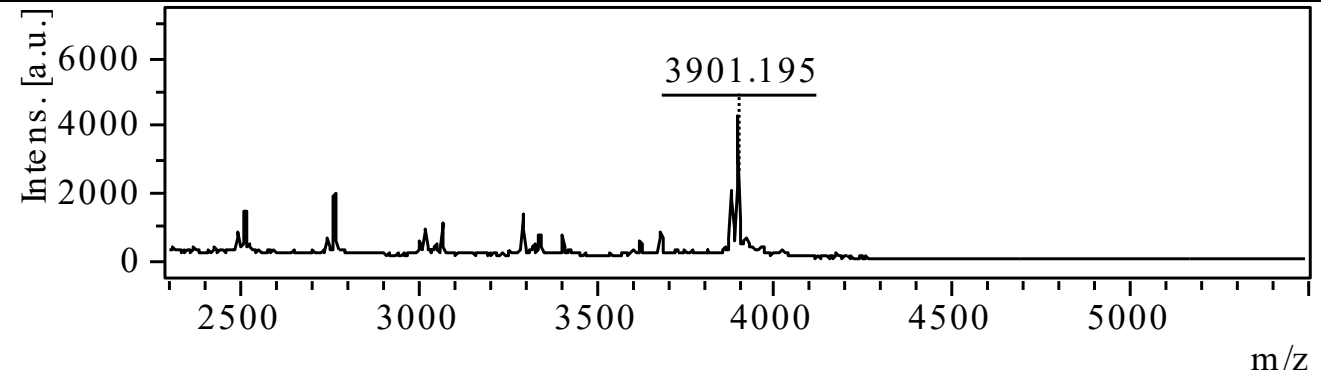
**Ex2-34**	$C_{174}H_{247}N_{65}O_{63}$	4257.282	4255.816	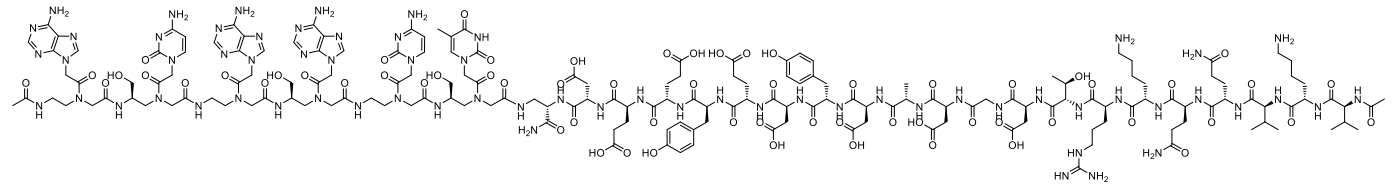	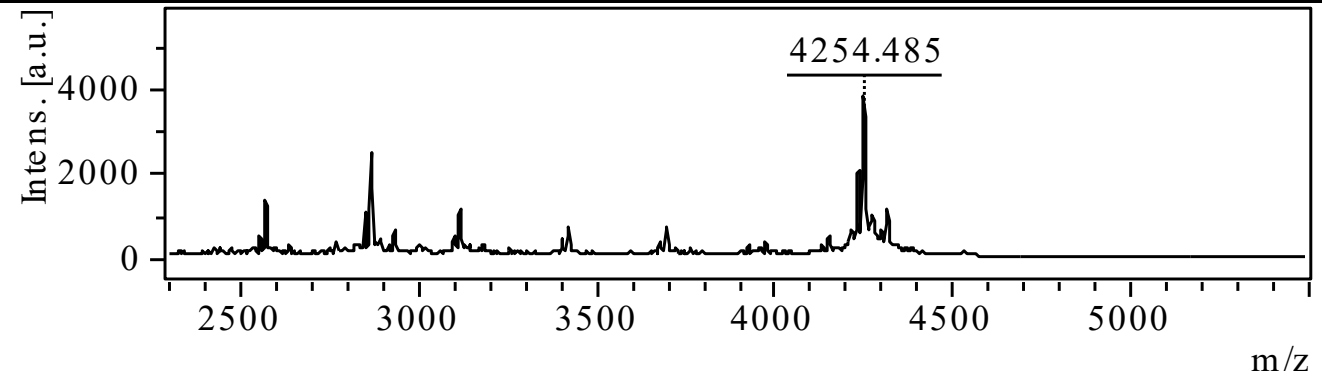

**Ex2-35**	$C_{167}H_{230}N_{62}O_{65}$	4146.046	4144.663	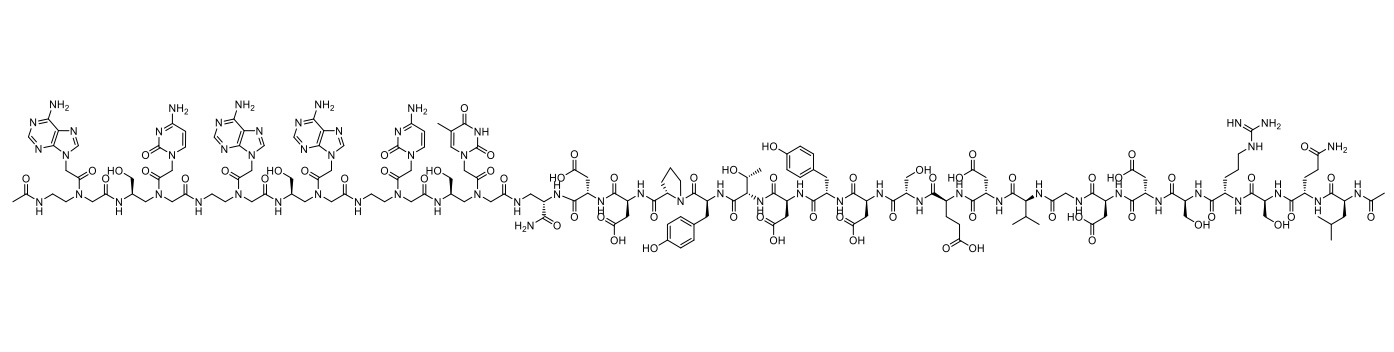	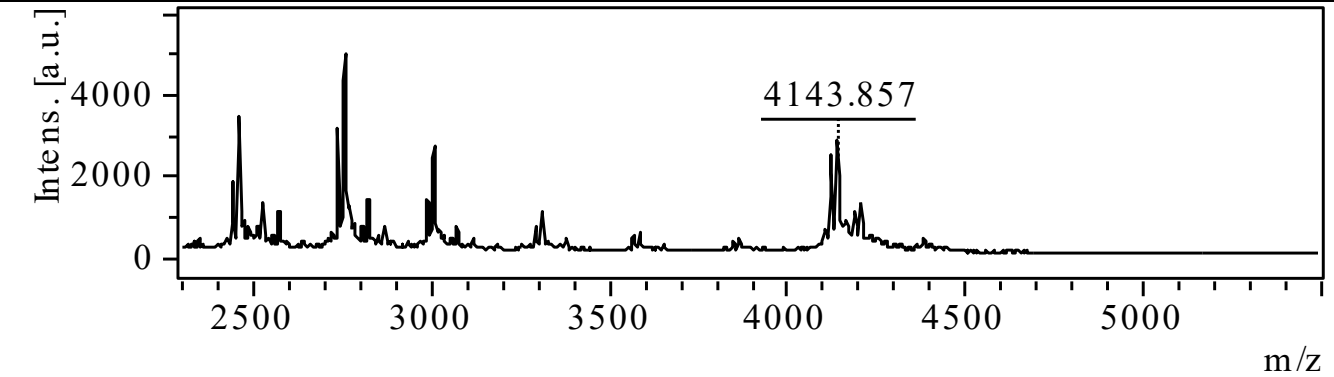
**Ex2-36**	$C_{143}H_{186}F_4N_{56}O_{51}S_2$	3645.516	3644.309	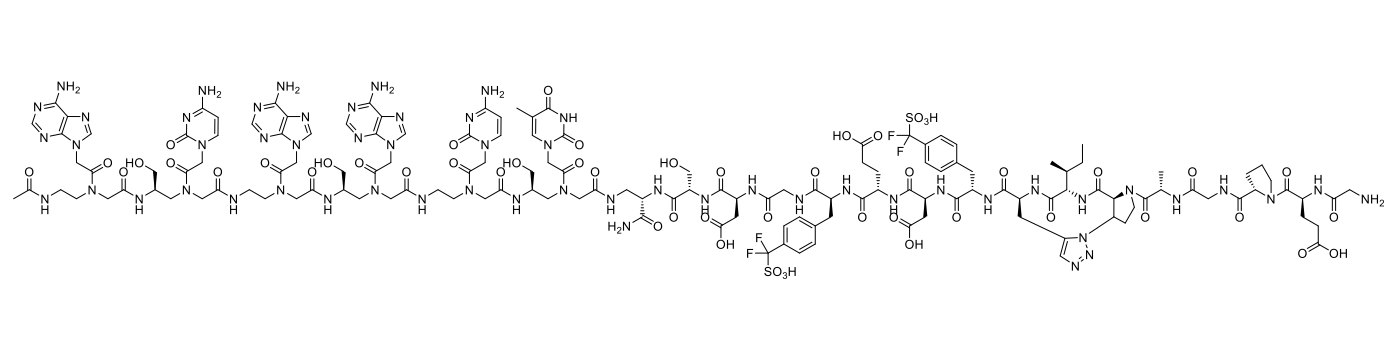	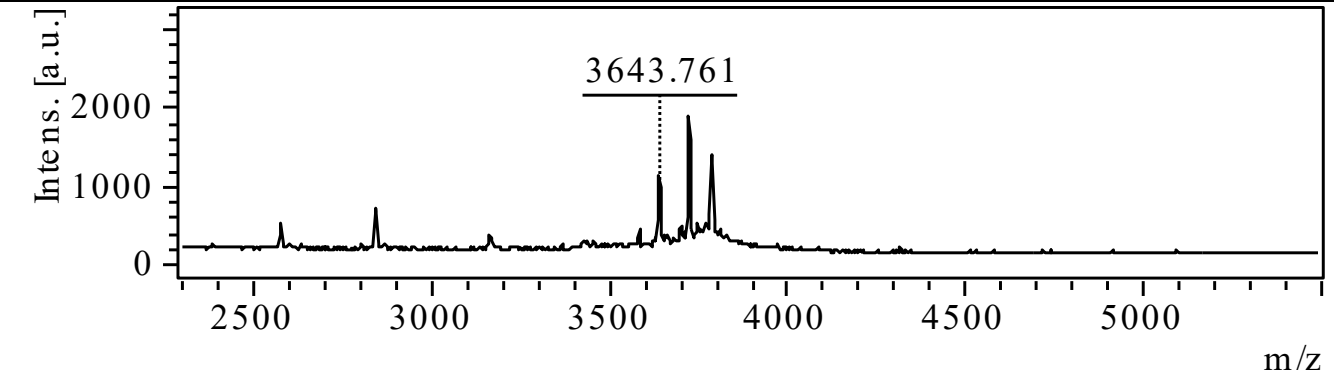
**Ex2-37**	$C_{148}H_{198}N_{52}O_{52}$	3537.524	3536.448	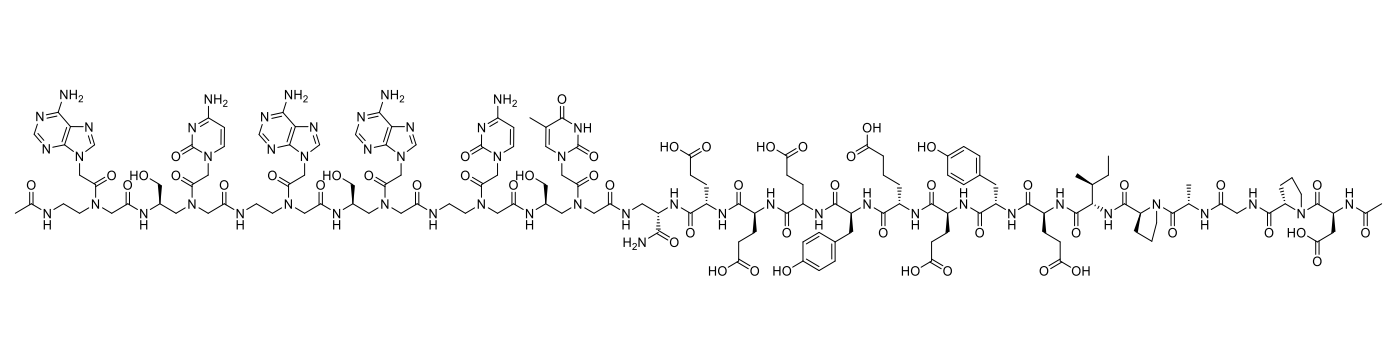	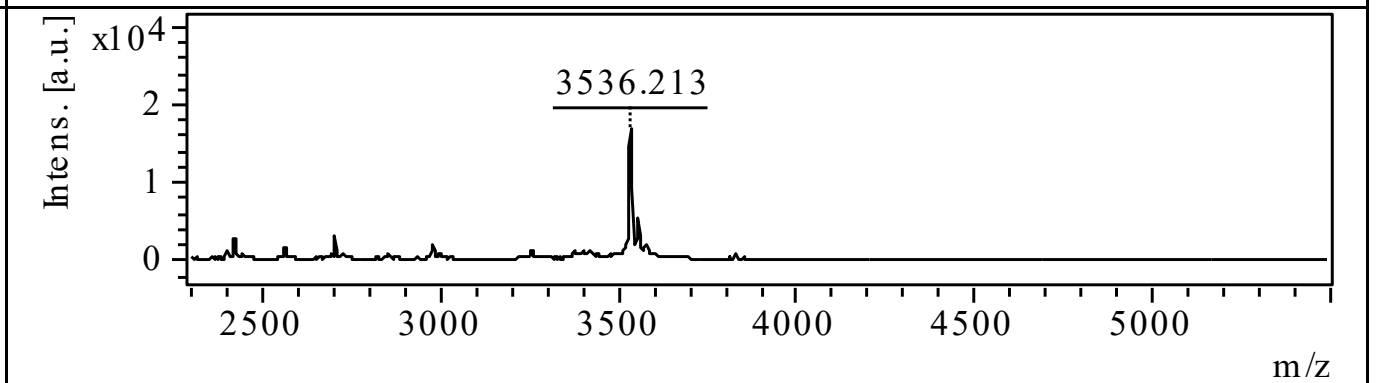
**Ex2-38**	$C_{145}H_{191}F_4N_{53}O_{53}S_2$	3664.555	3663.329	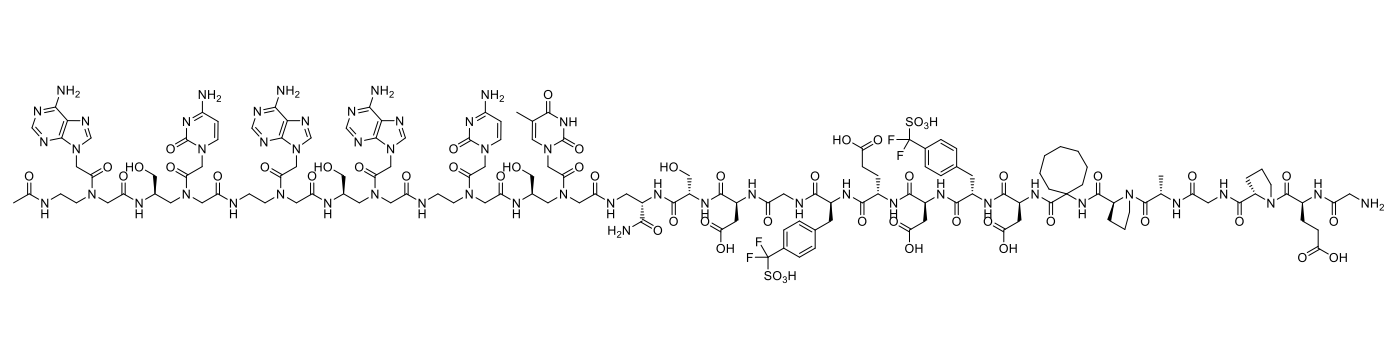	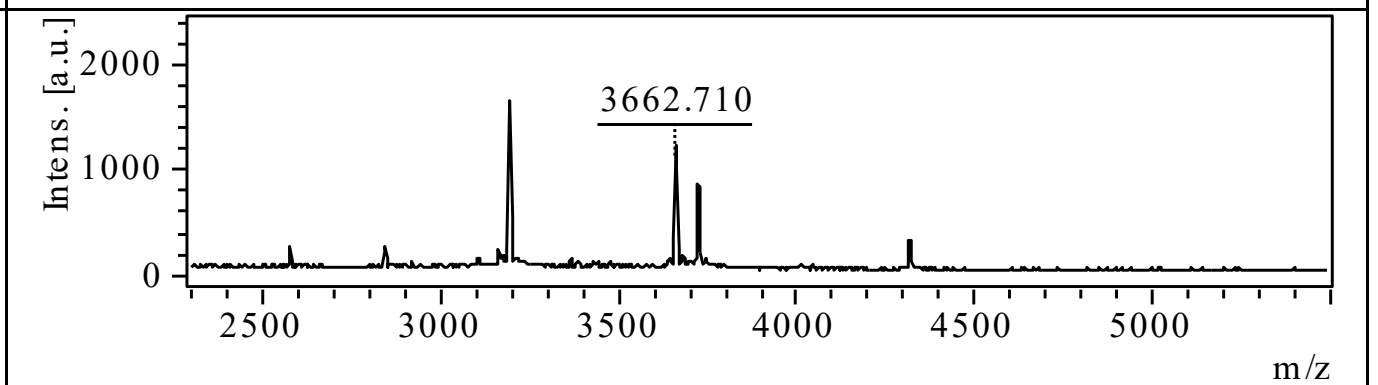
**Ex2-39**	$C_{140}H_{185}N_{55}O_{51}$	3454.354	3453.361 *3,421.381(Deg. of NO_2 on MALDI)	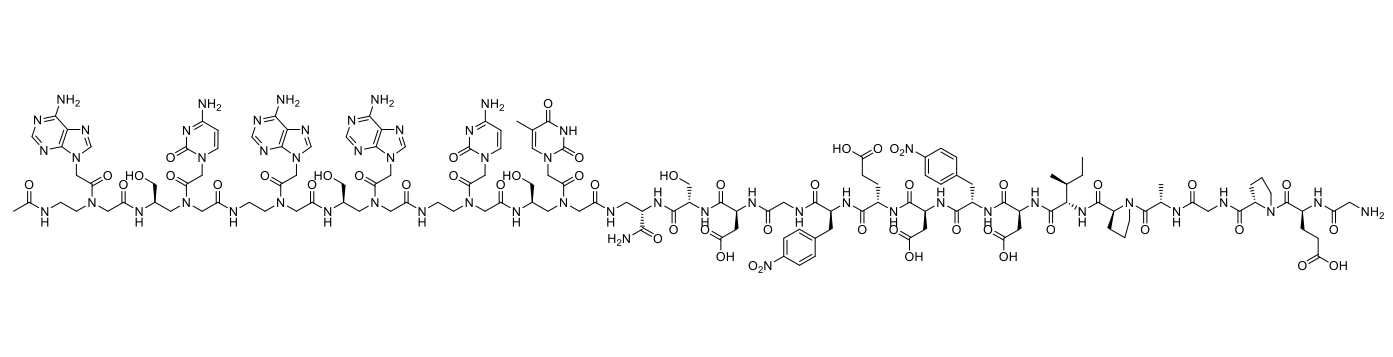	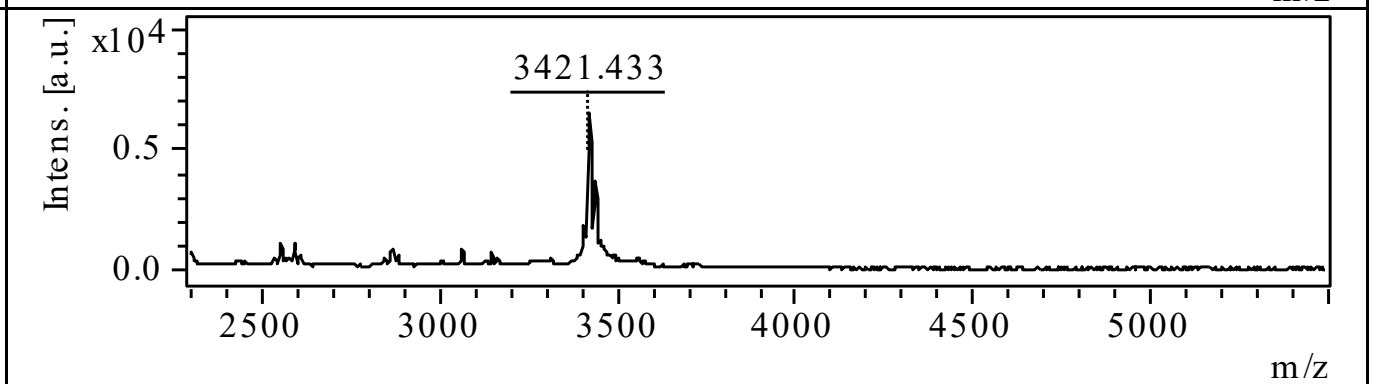
**Ex2-40**	$C_{142}H_{189}N_{55}O_{55}S_2$	3610.524	3609.316	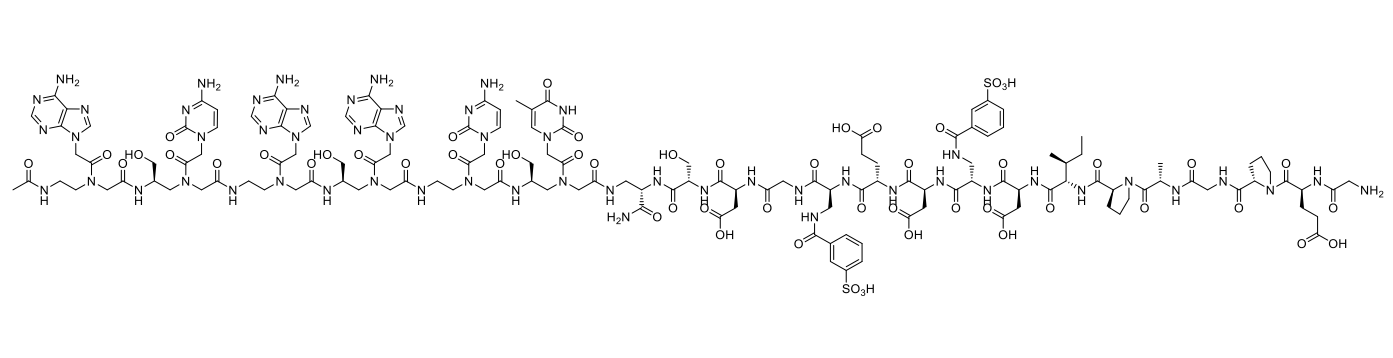	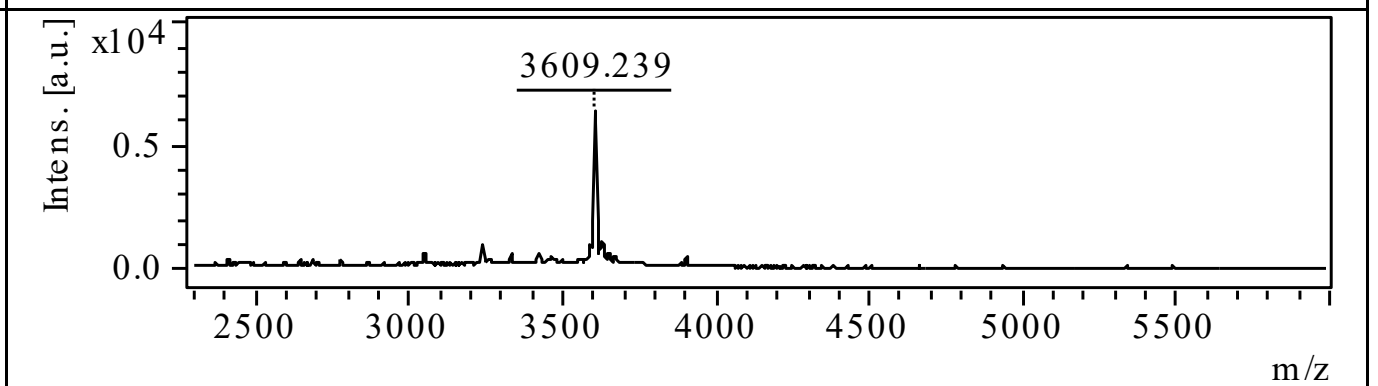

**Ex2-41**	$C_{145}H_{194}N_{56}O_{57}S_2$	3697.602	3696.348	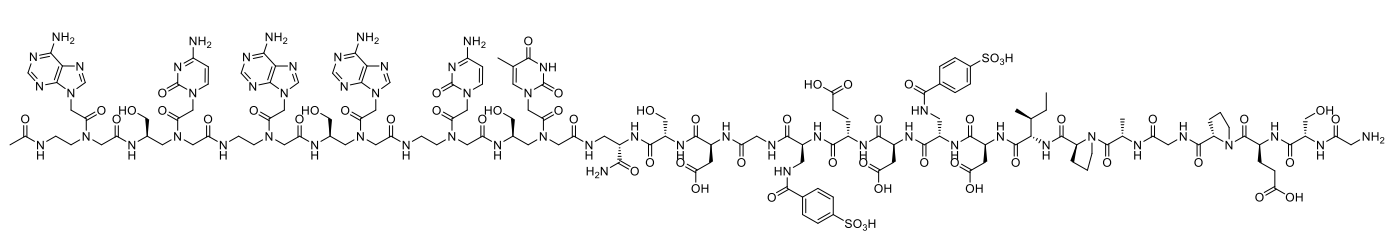	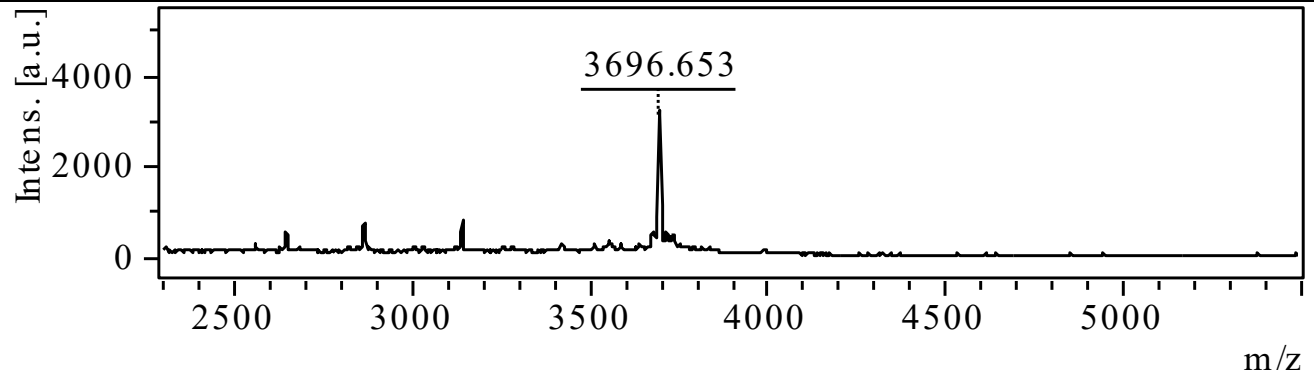
**Ex2-42**	$C_{135}H_{191}N_{59}O_{51}P_2$	3518.323	3517.367	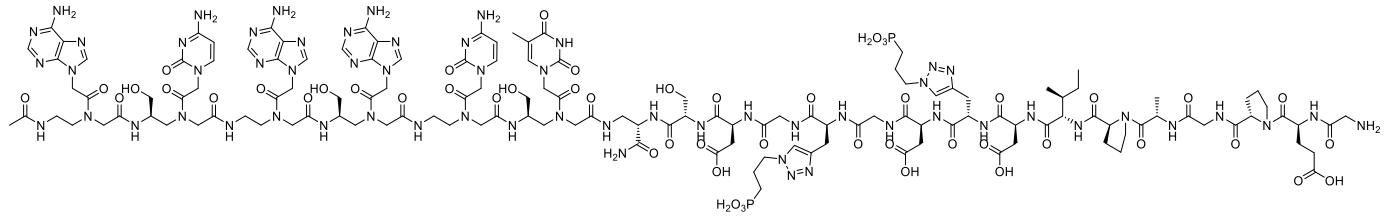	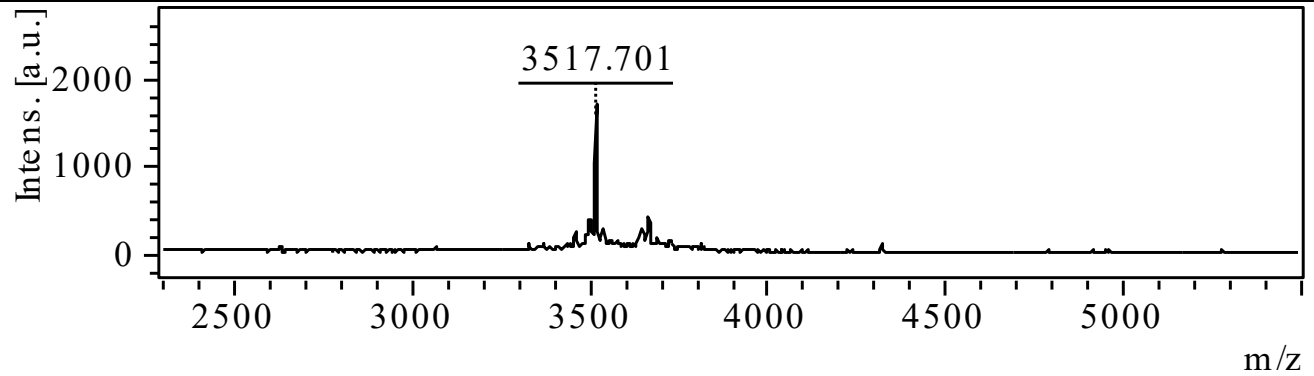
**Ex2-43**	$C_{140}H_{194}N_{62}O_{51}$	3561.475	3560.45	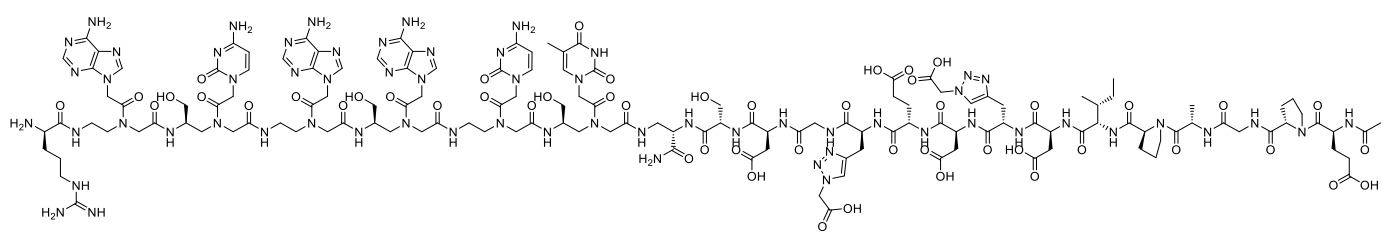	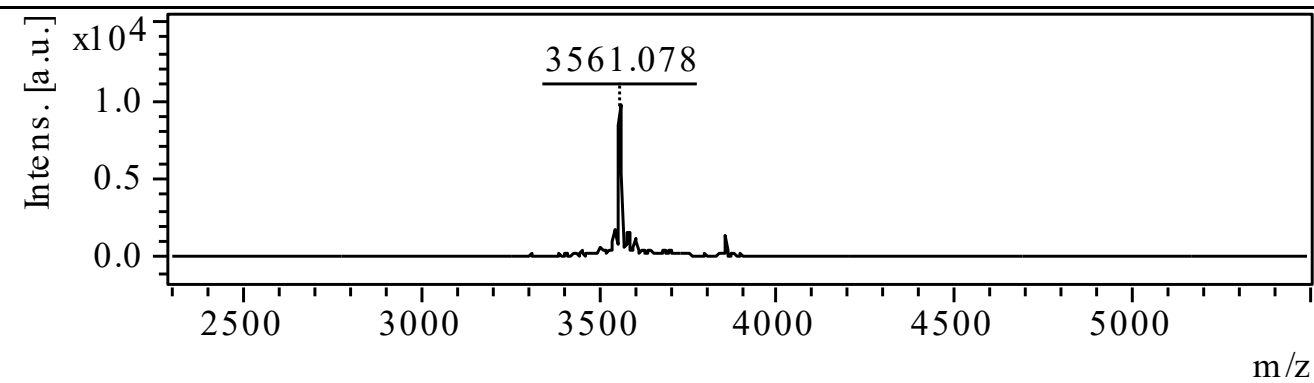
**Ex2-44**	$C_{134}H_{193}N_{55}O_{47}$	3326.356	3325.444	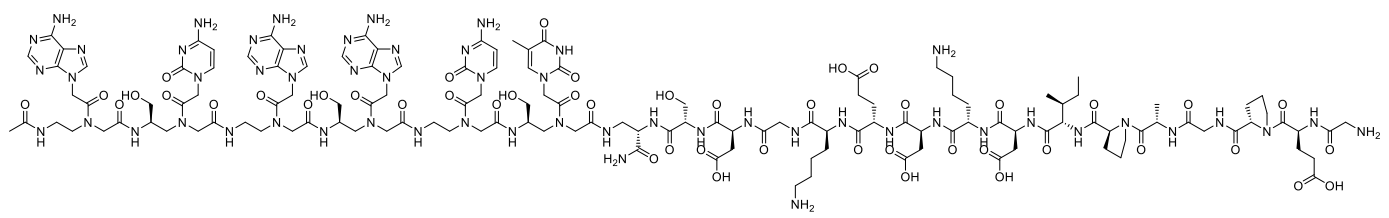	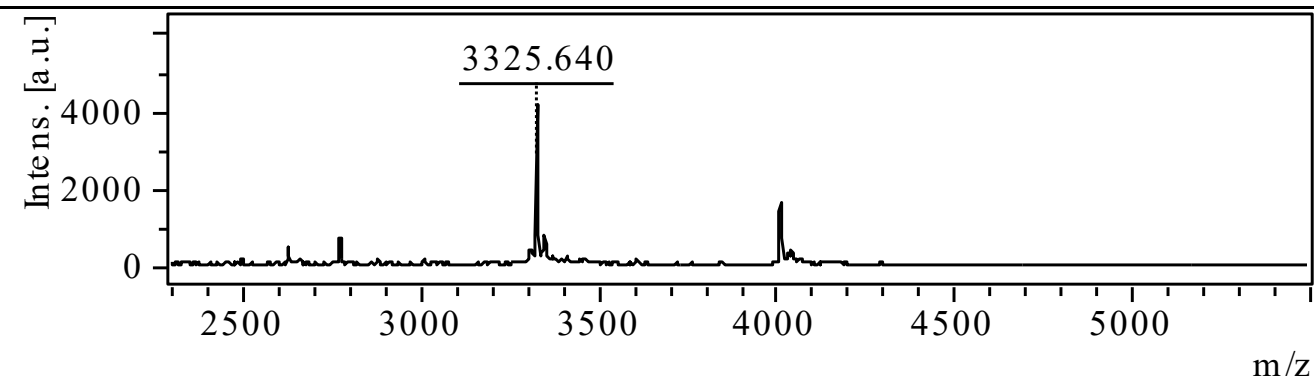
**Ex2-45**	$C_{150}H_{189}F_2N_{53}O_{49}$	3556.481	3555.393	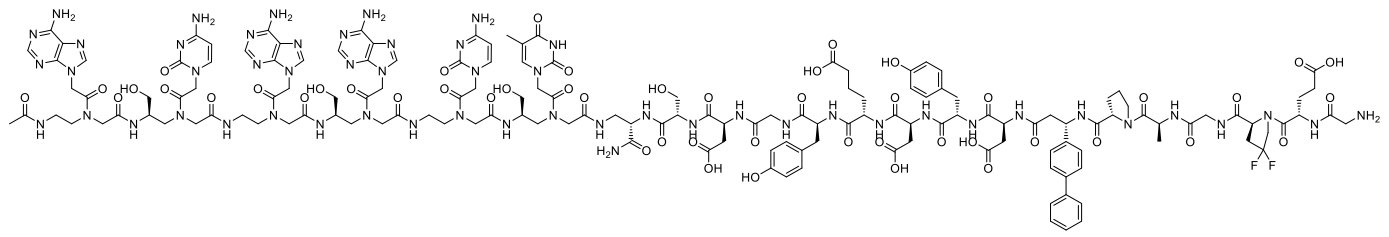	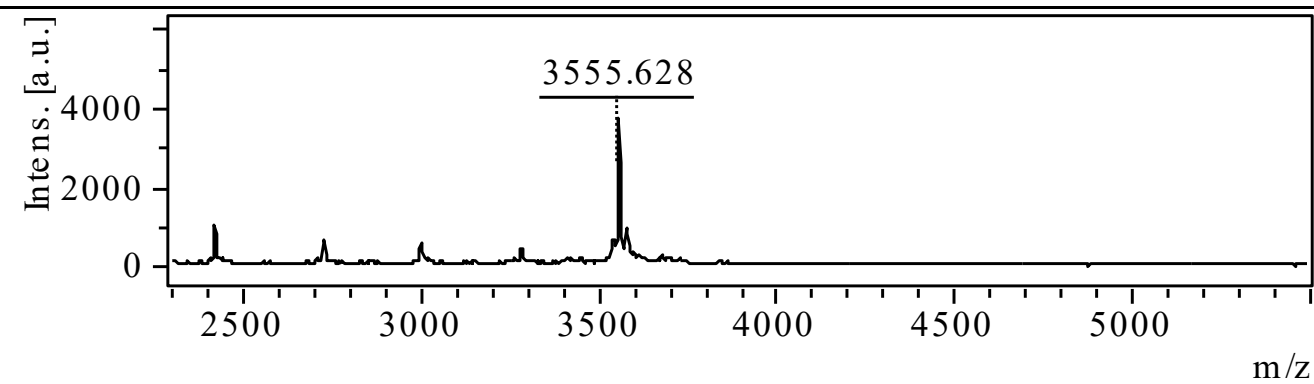
**Ex2-46**	$C_{142}H_{183}ClN_{54}O_{48}$	3449.806	3448.326	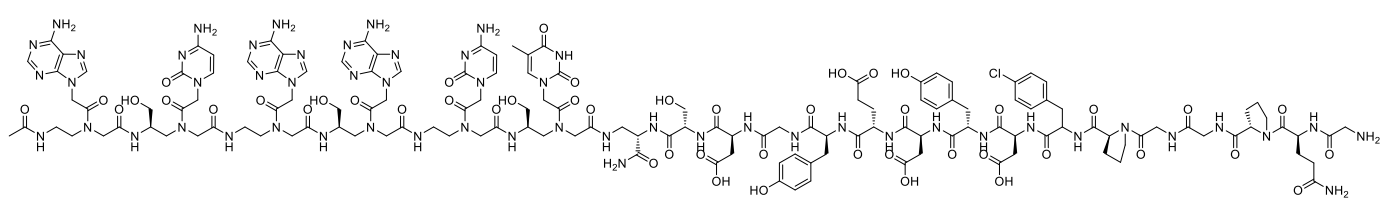	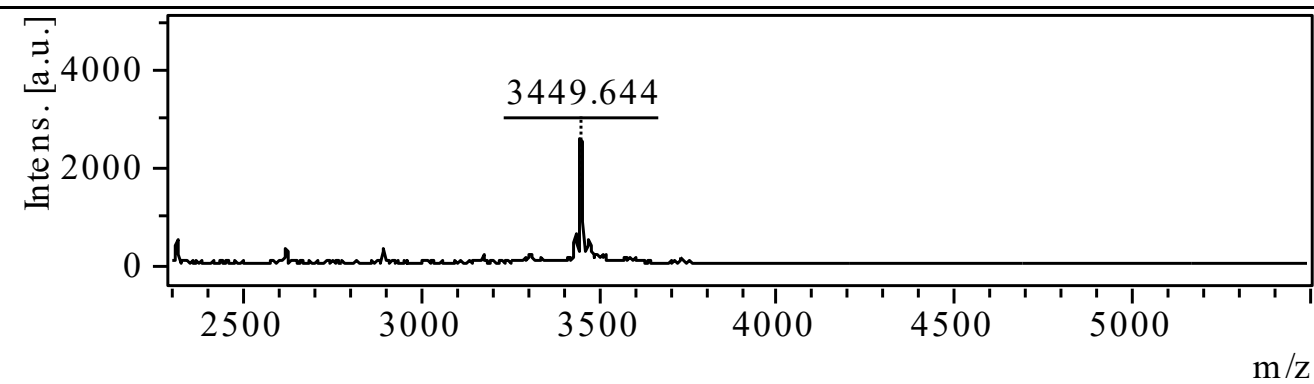

**Ex2-47**	$C_{142}H_{189}F_2N_{53}O_{56}P_2$	3634.333	3633.305	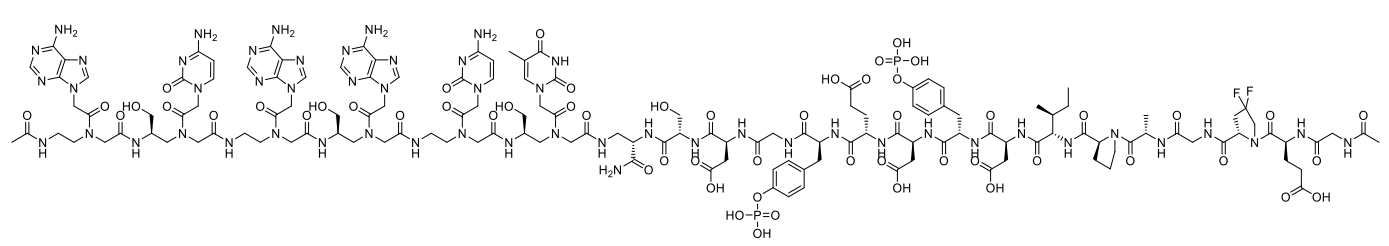	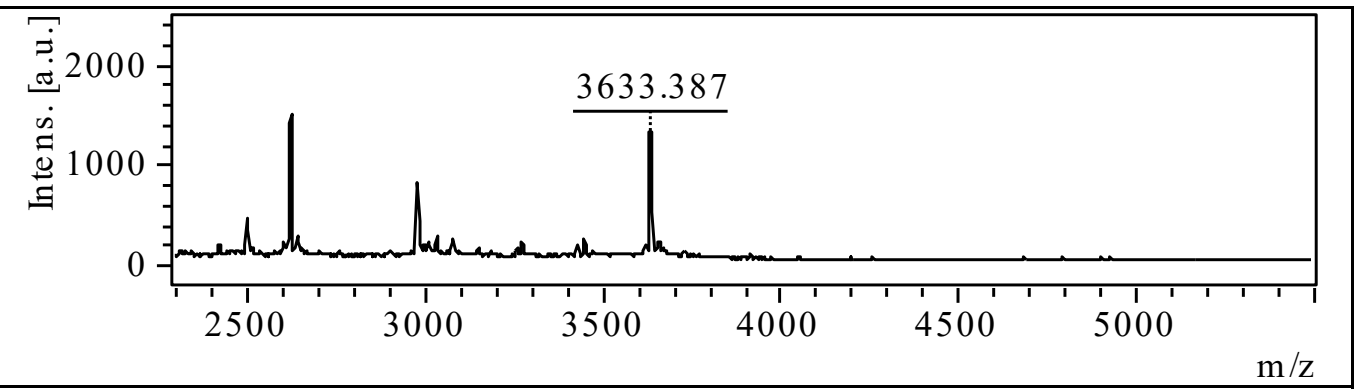
**Ex2-48**	$C_{142}H_{185}F_6N_{53}O_{47}$	3500.356	3499.365	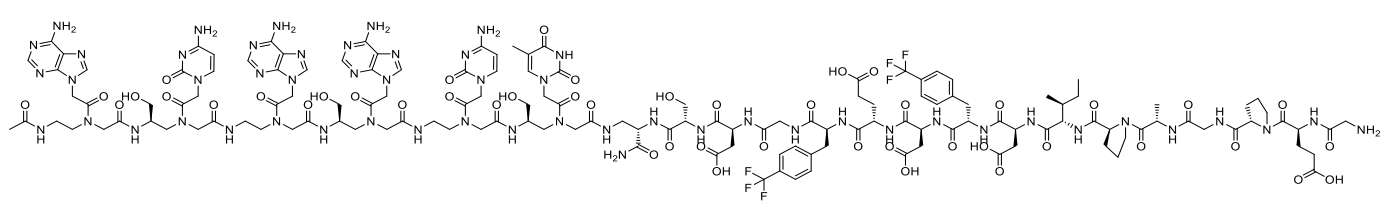	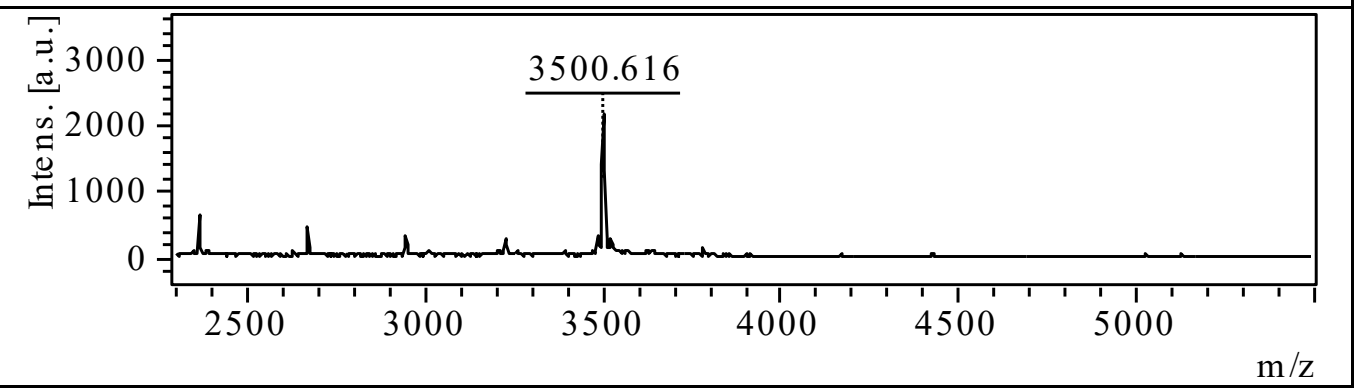
**Ex2-49**	$C_{128}H_{179}N_{53}O_{47}S_2$	3276.284	3275.272	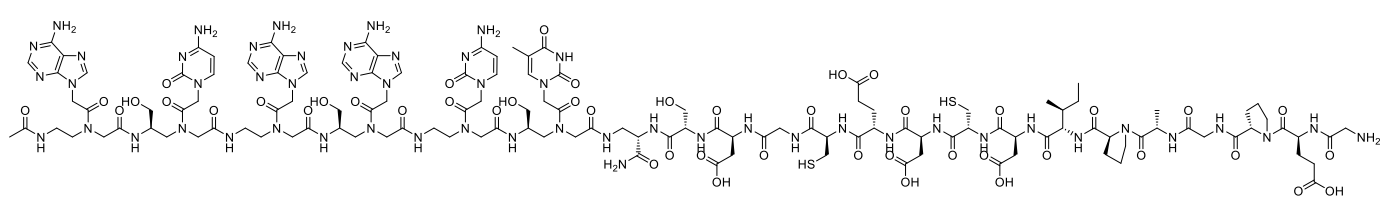	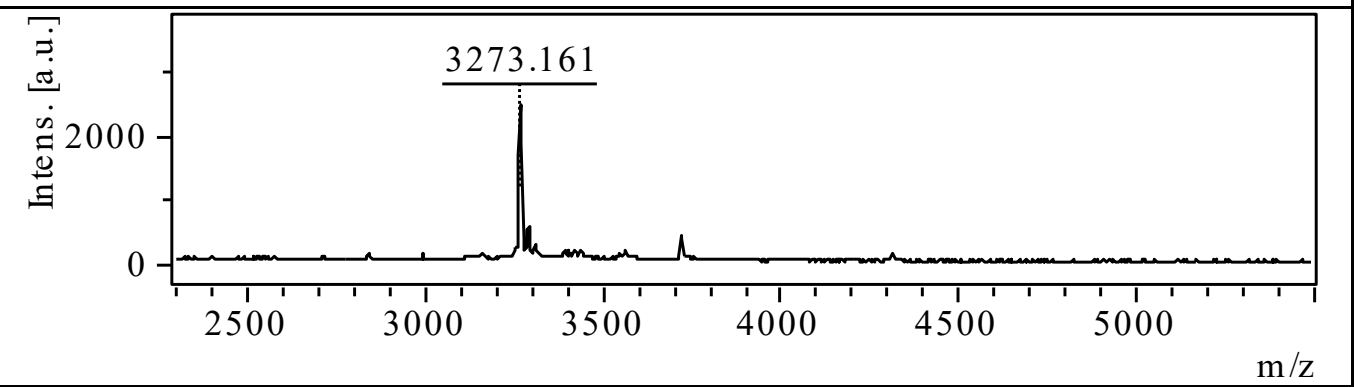
**Ex2-50**	$C_{96}H_{137}N_{47}O_{31}$	2445.450	2445.062	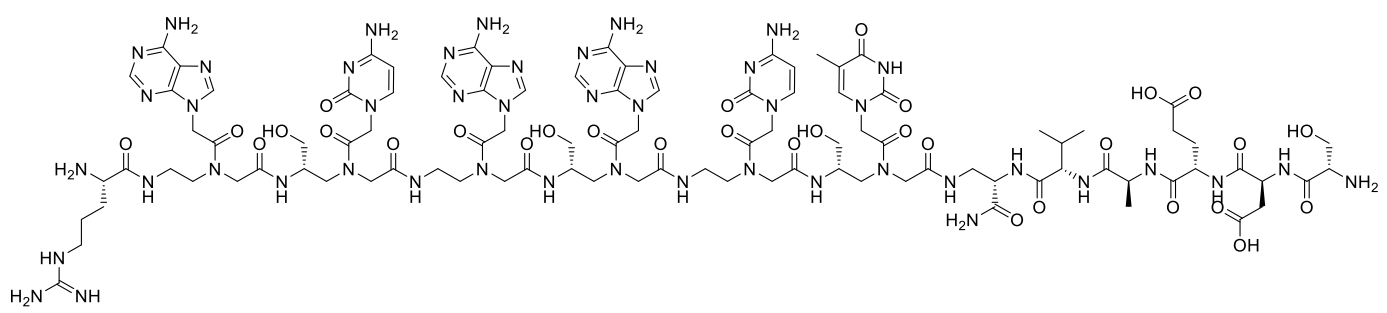	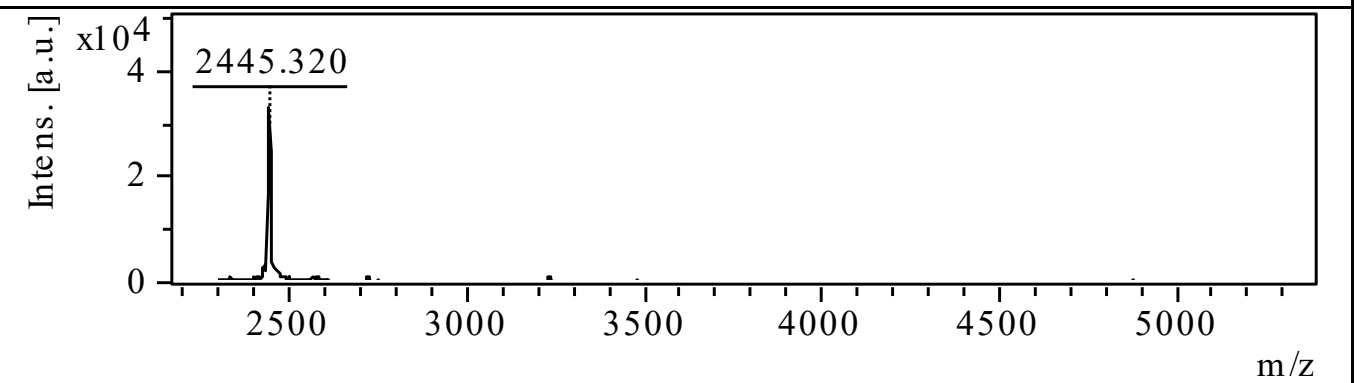

Supplementary Table S6*: Active Site Library Members*

	Chemical Formula	MW	m/z	Structure	MALDI
**AS-1**	$C_{211}H_{284}N_{92}O_{65}S$	5181.232	5180.153	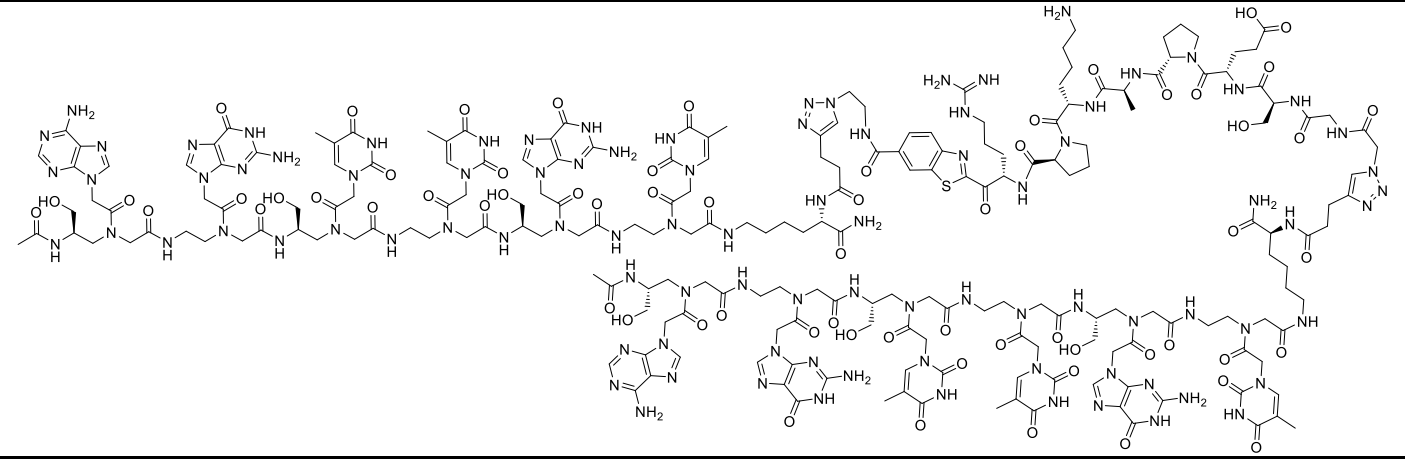	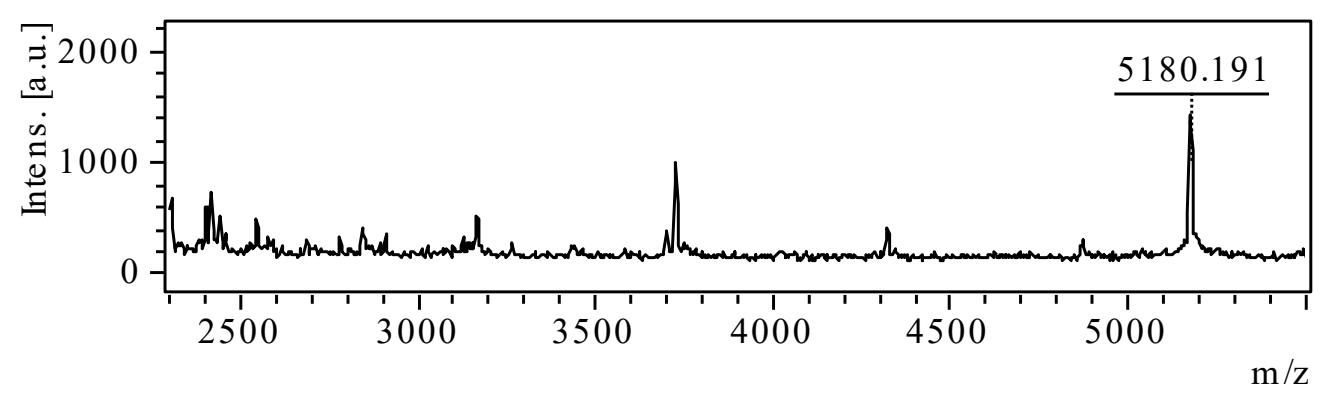
**AS-2**	$C_{211}H_{277}N_{91}O_{63}S$	5128.171	5127.106	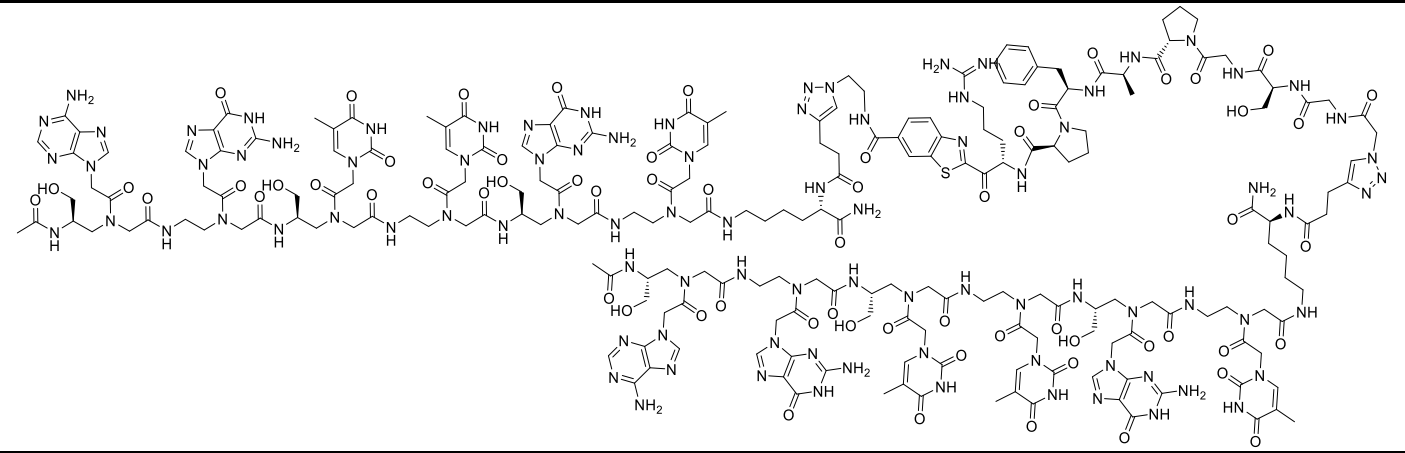	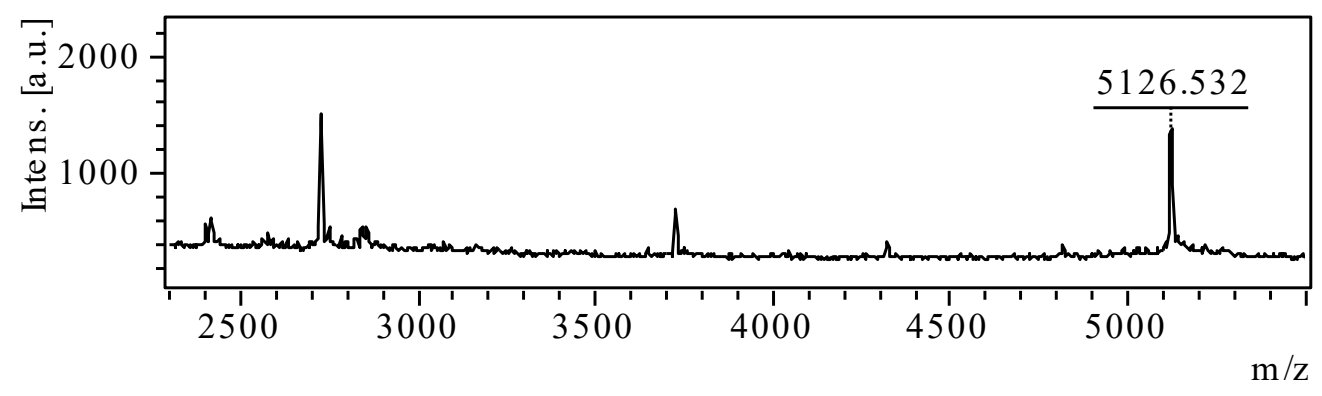
**AS-3**	$C_{214}H_{284}N_{92}O_{66}S$	5233.264	5232.148	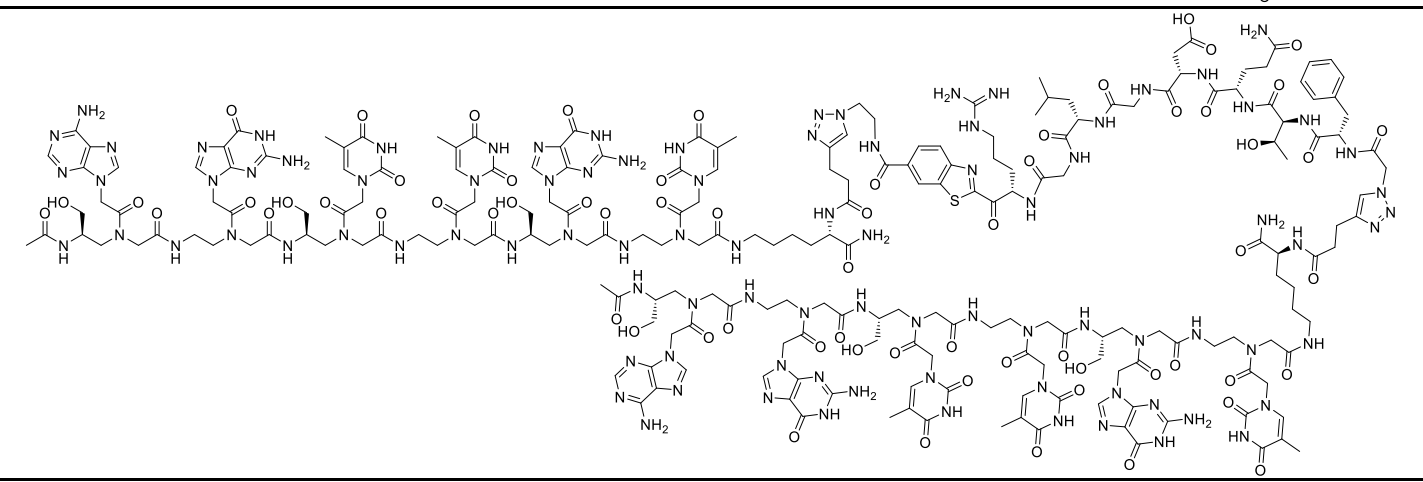	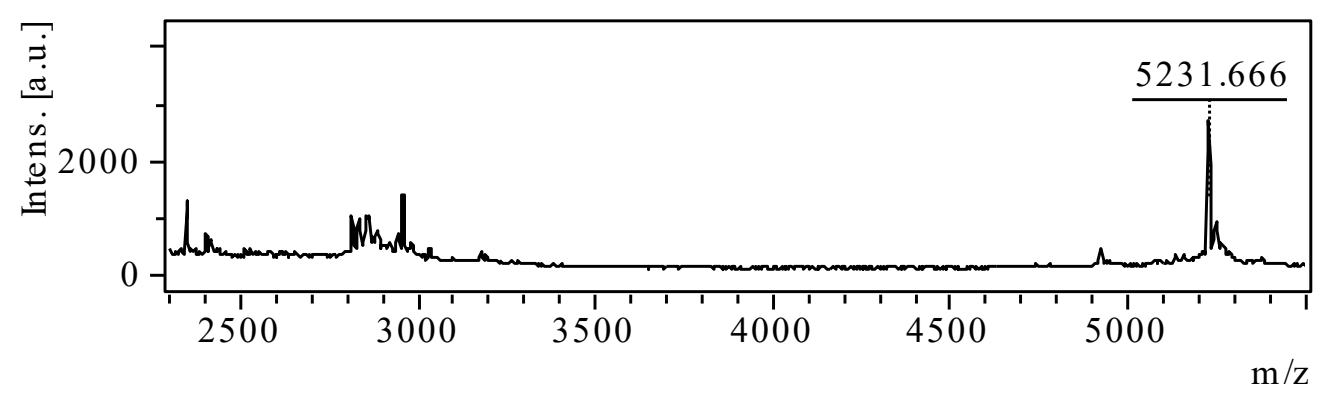
**AS-4**	$C_{214}H_{285}N_{93}O_{67}S$	5264.278	5263.154	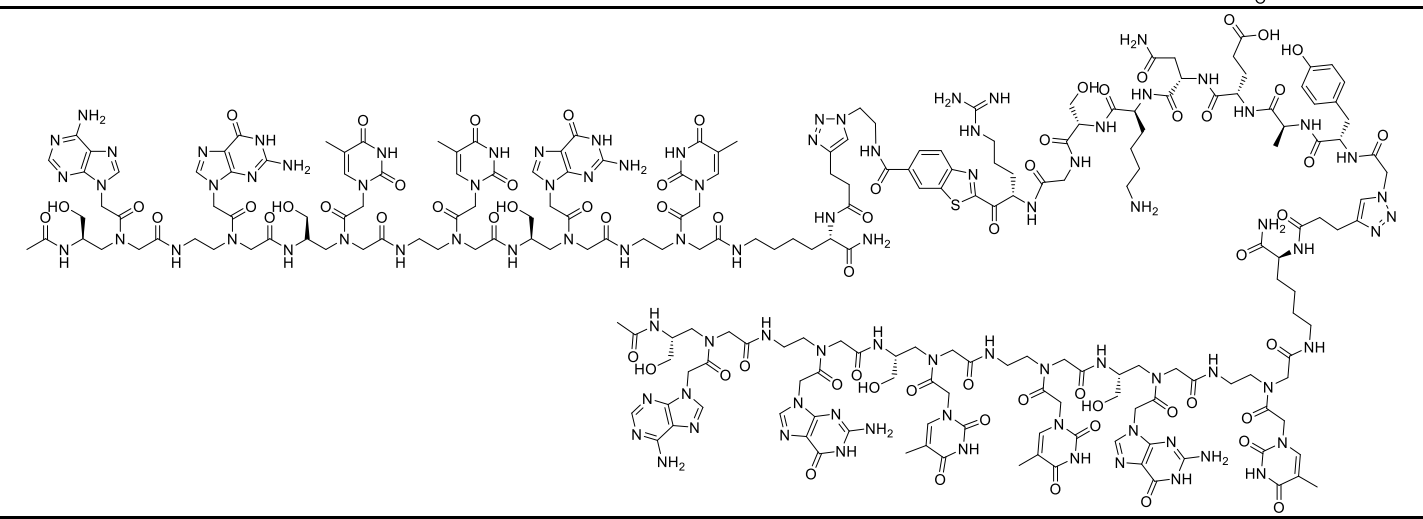	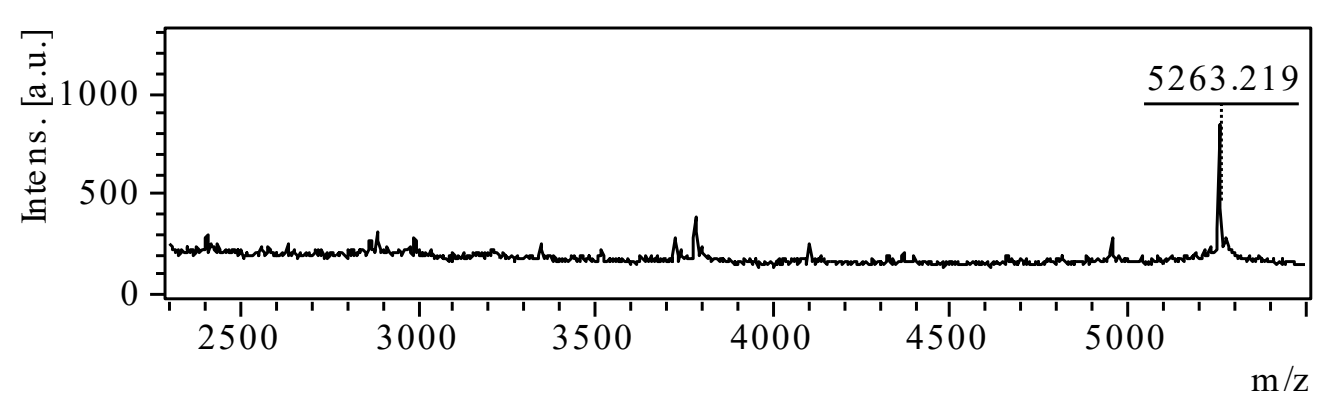

**AS-5**	$C_{211}H_{287}N_{91}O_{65}S$	5170.249	5169.174	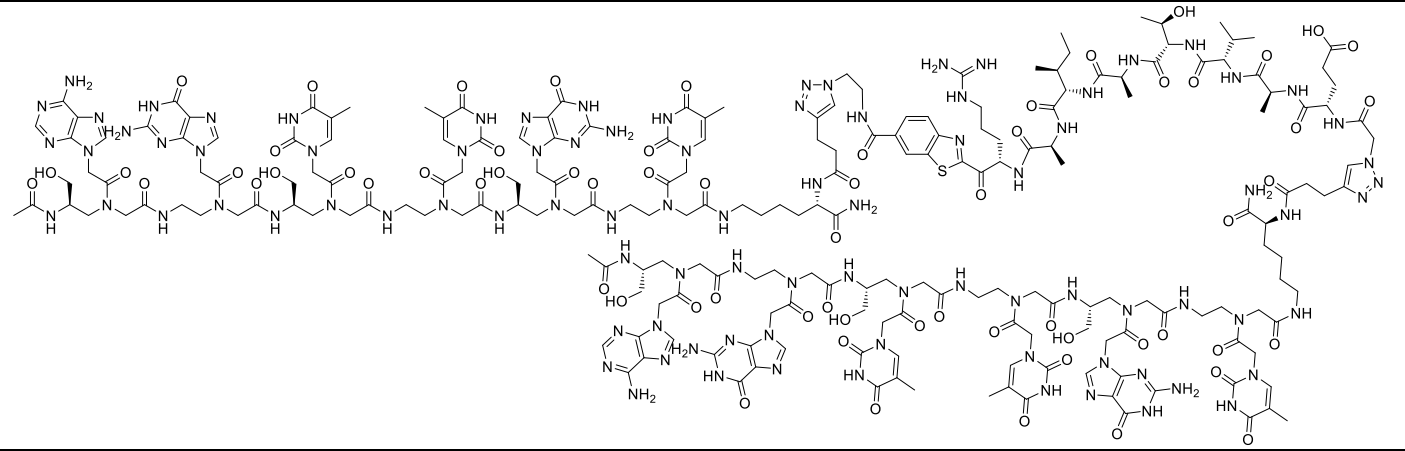	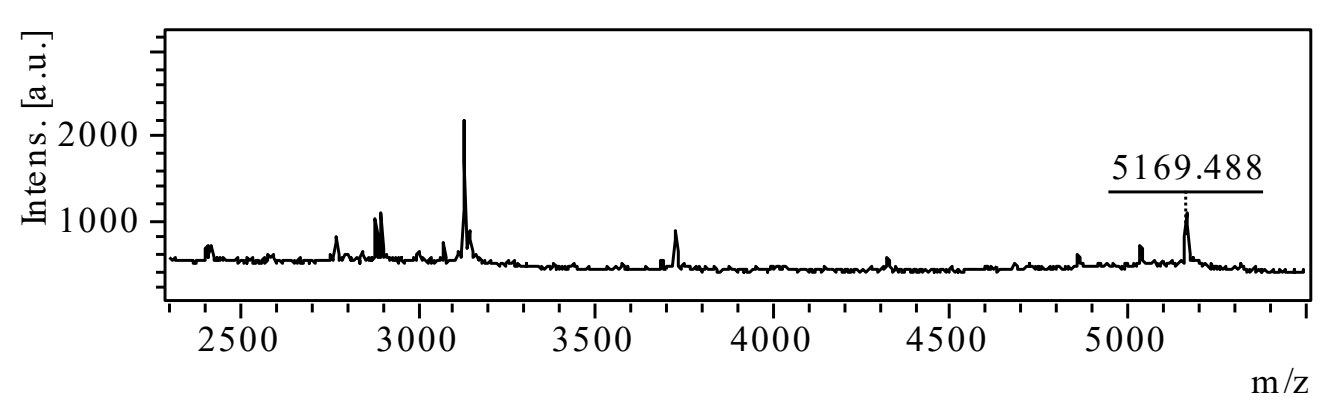
**AS-6**	$C_{204}H_{288}N_{86}O_{71}$	5081.079	5080.164	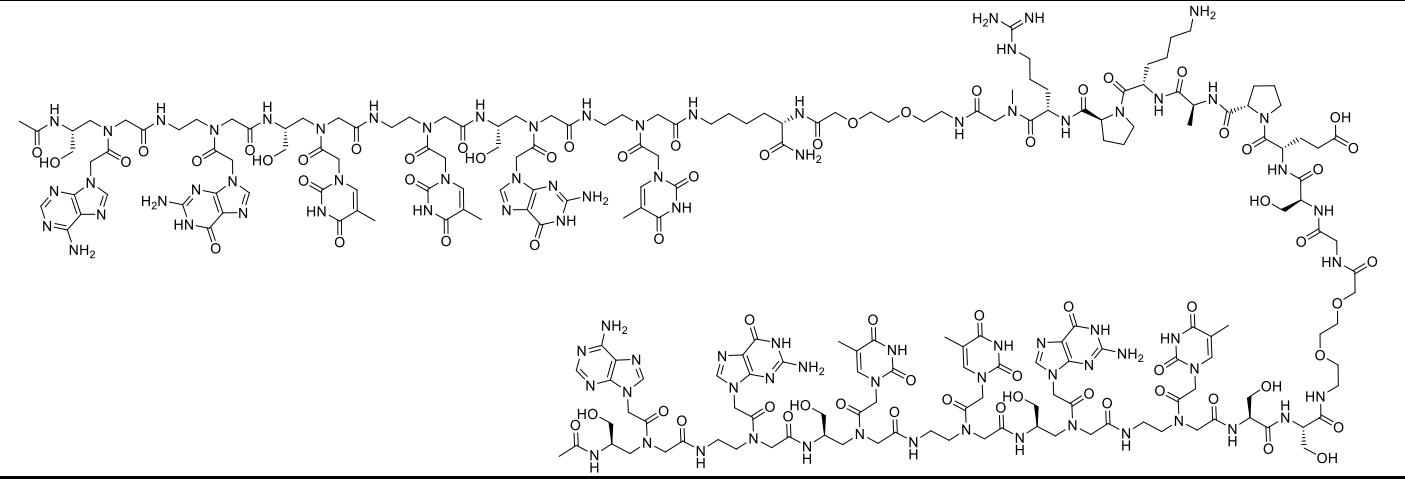	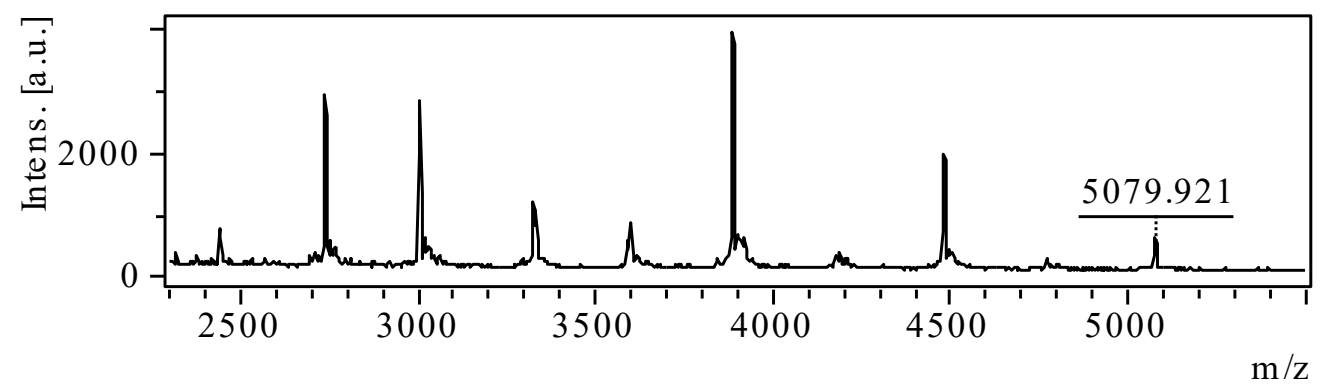
**AS-7**	$C_{207}H_{285}N_{85}O_{71}$	5100.081	5099.137	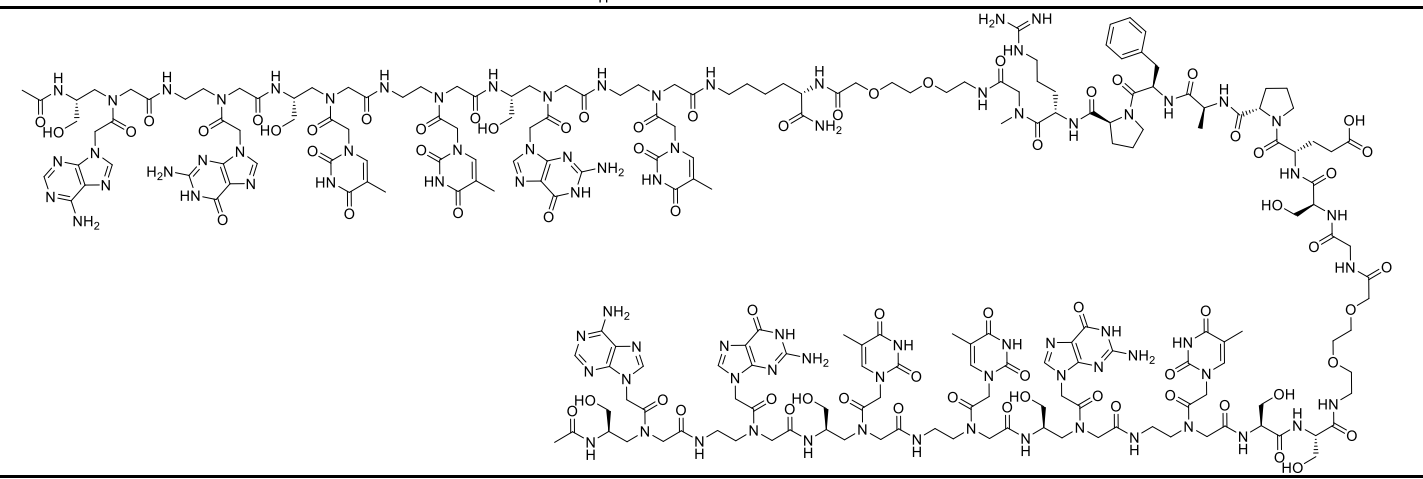	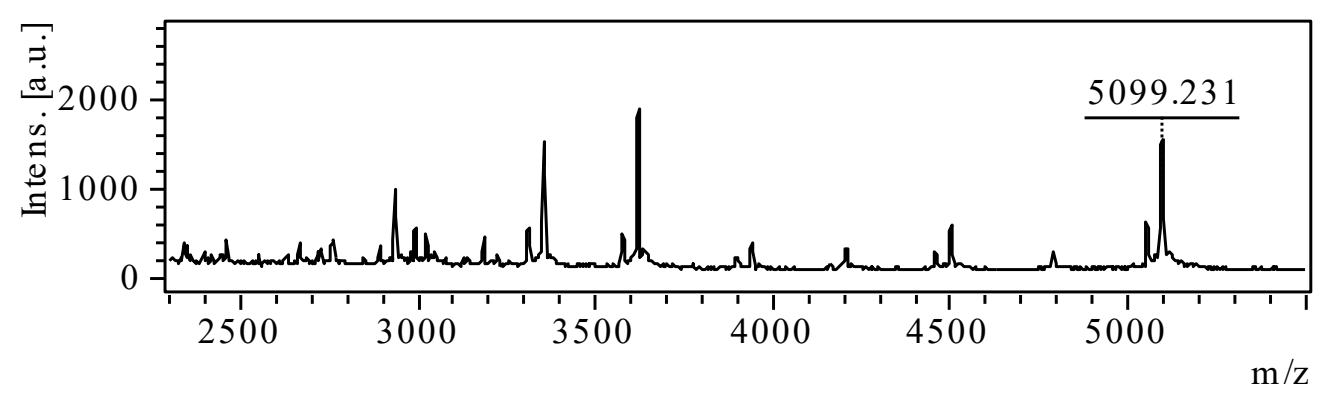
**AS-8**	$C_{206}H_{283}N_{83}O_{74}$	5106.037	5105.100	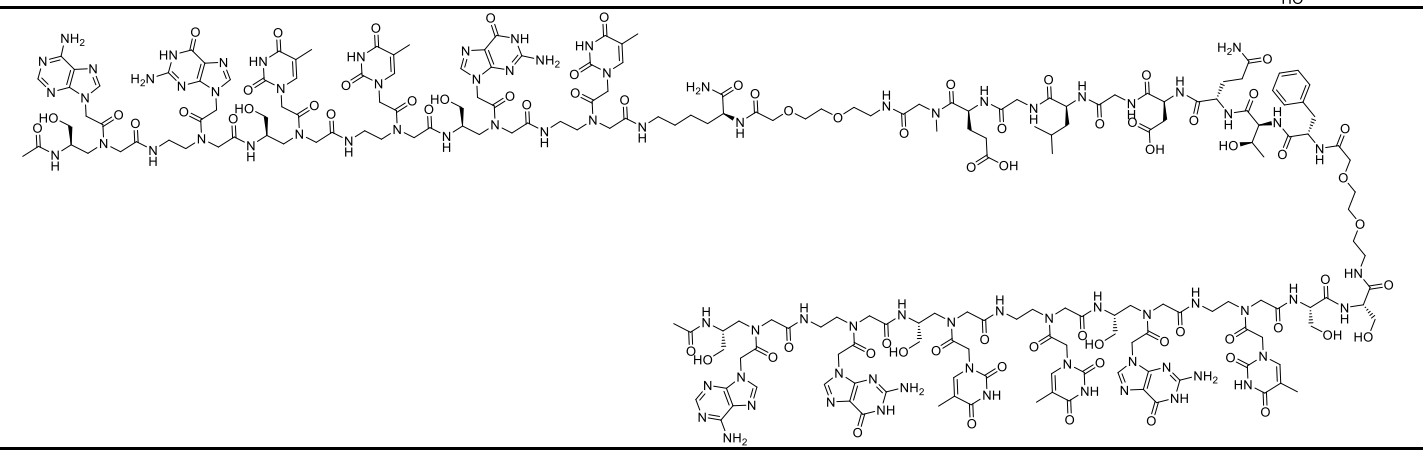	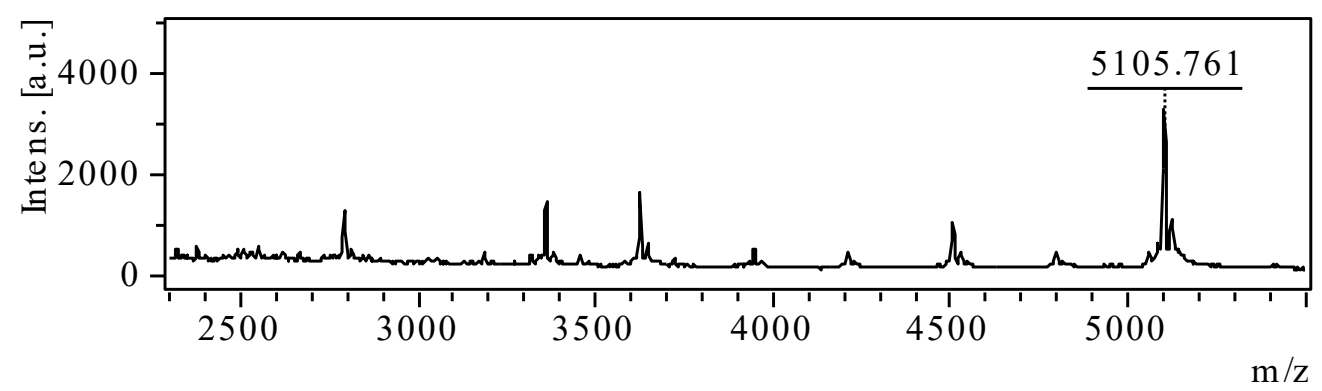
**AS-9**	$C_{207}H_{287}N_{85}O_{75}$	5166.093	5165.132	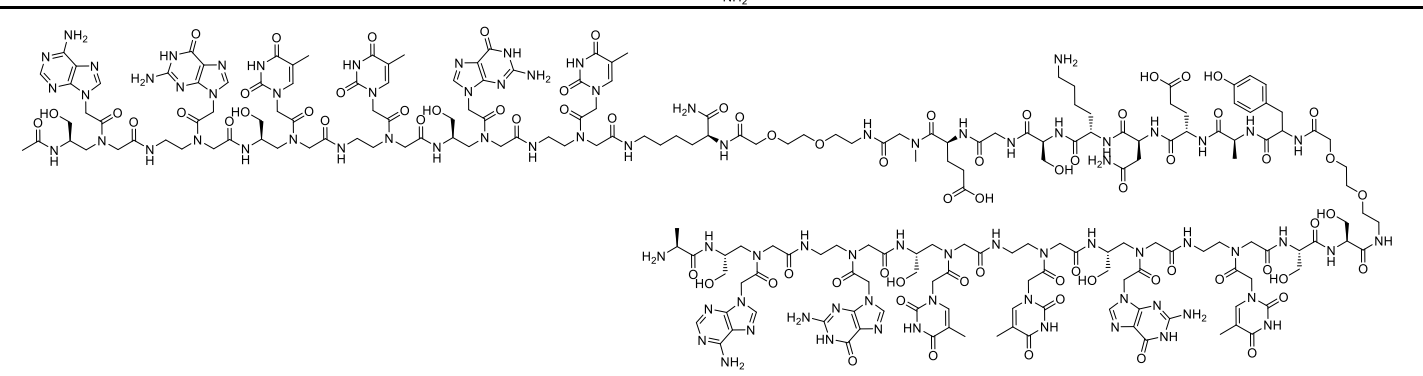	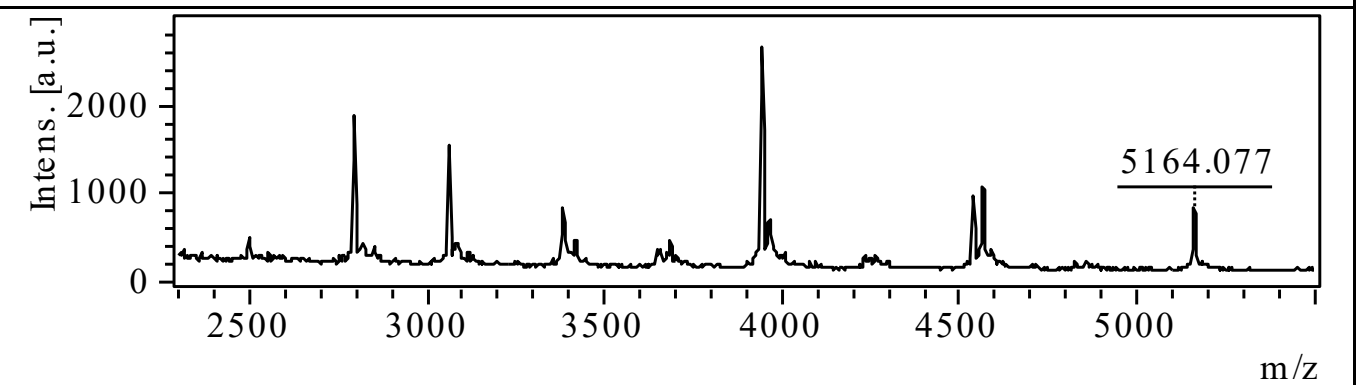

**AS-10**	$C_{203}H_{286}N_{82}O_{73}$	5043.022	5042.126	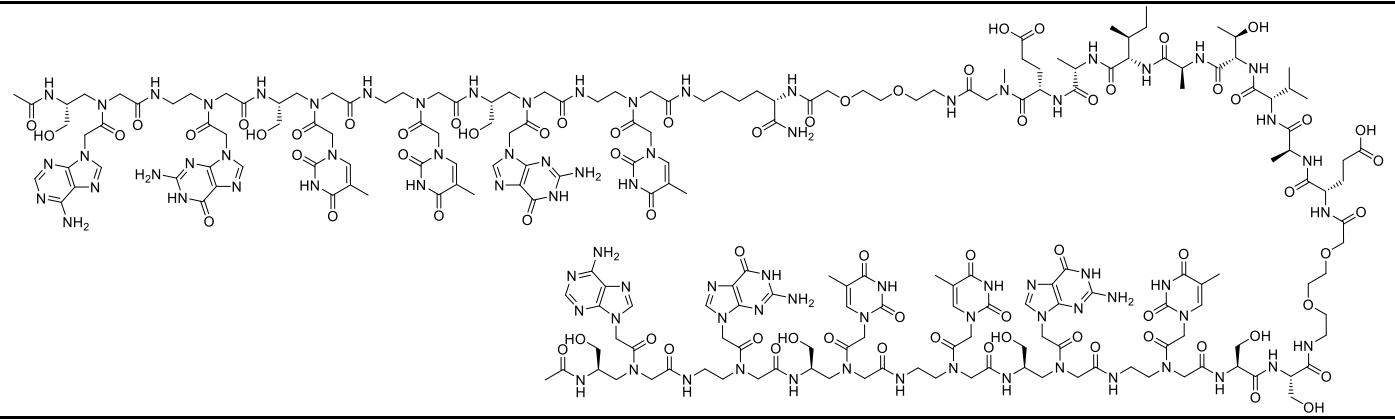	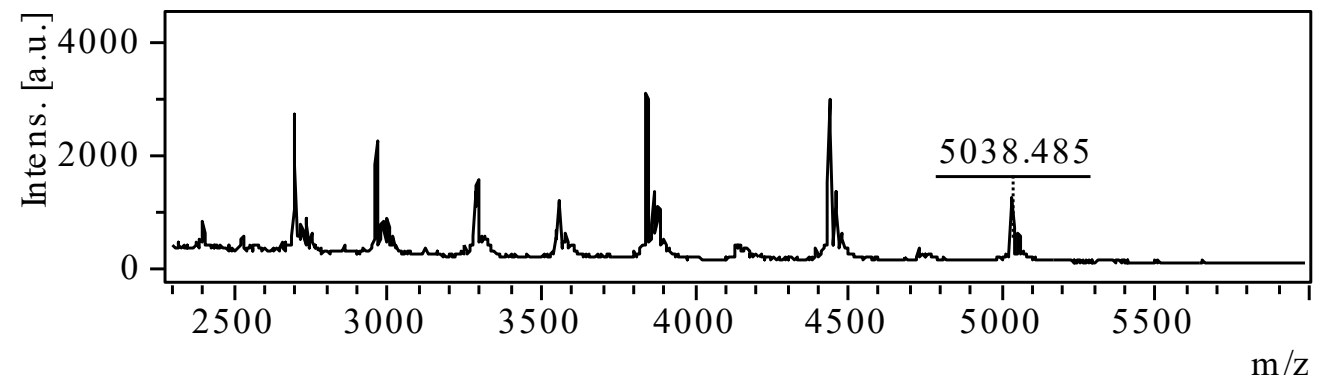
**AS-11**	$C_{213}H_{279}Cl_2N_{91}O_{64}S$	5241.108	5239.054	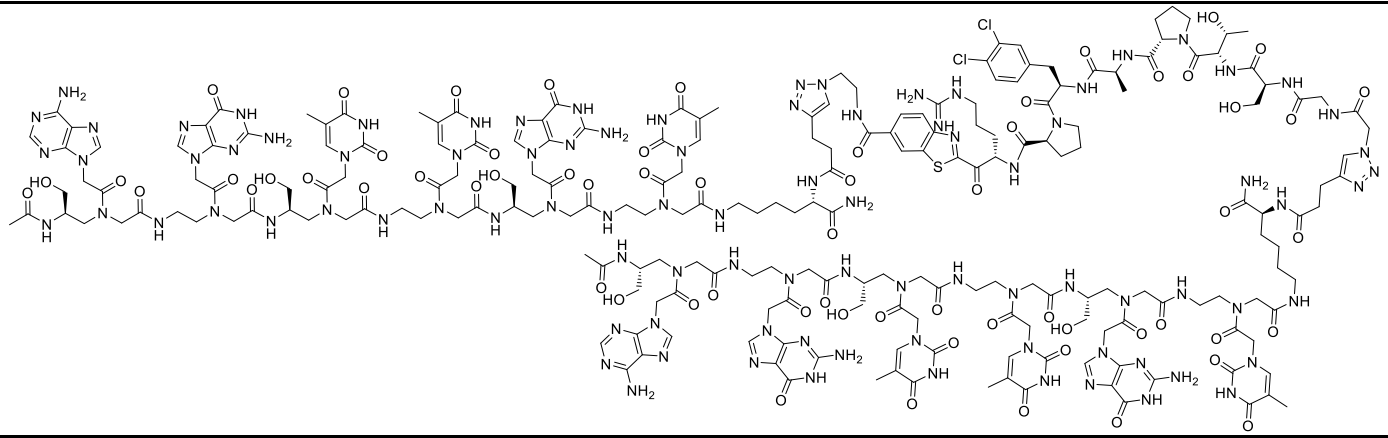	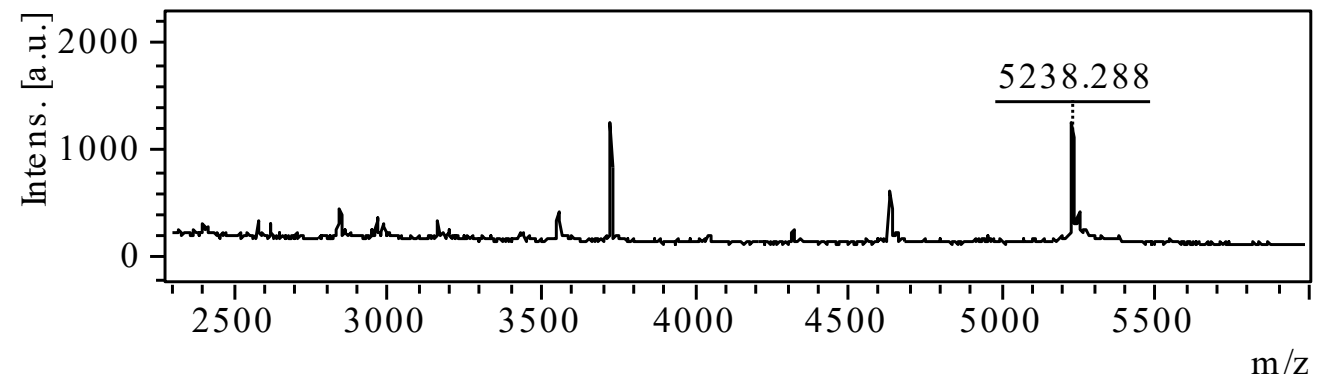
**AS-12**	$C_{189}H_{261}N_{79}O_{60}S$	4631.72	4630.959	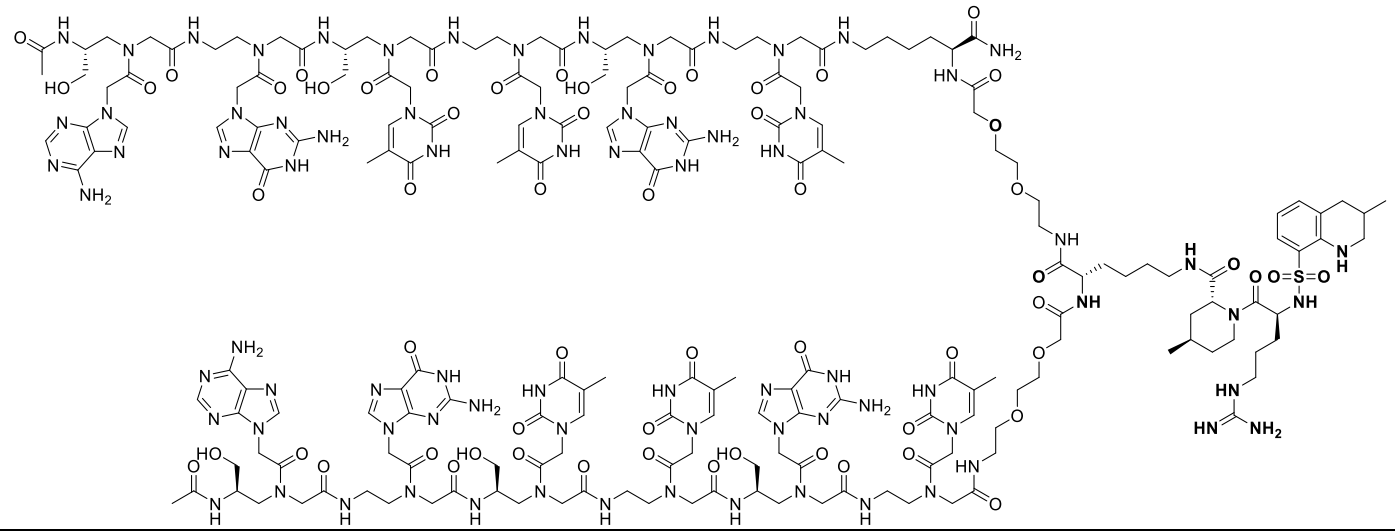	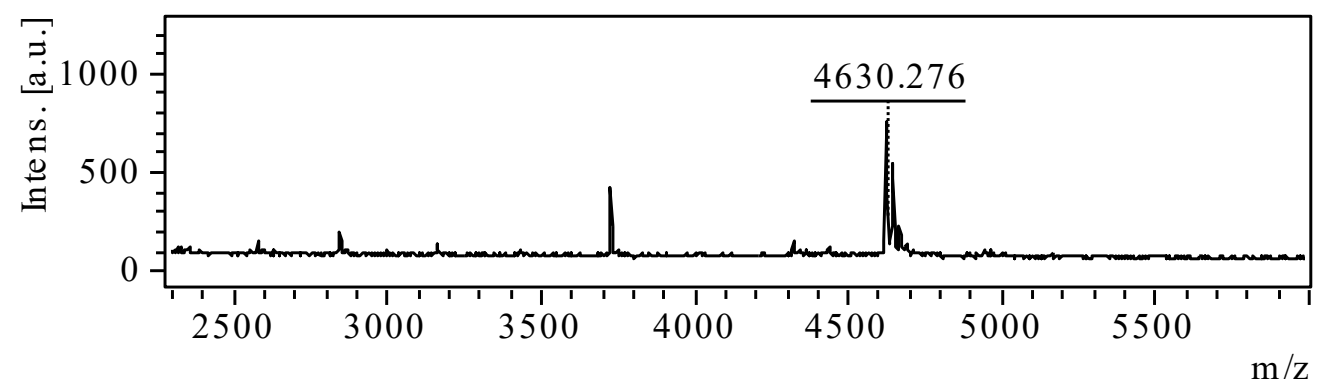
**AS-13**	$C_{195}H_{272}N_{80}O_{63}S$	4776.878	4776.033	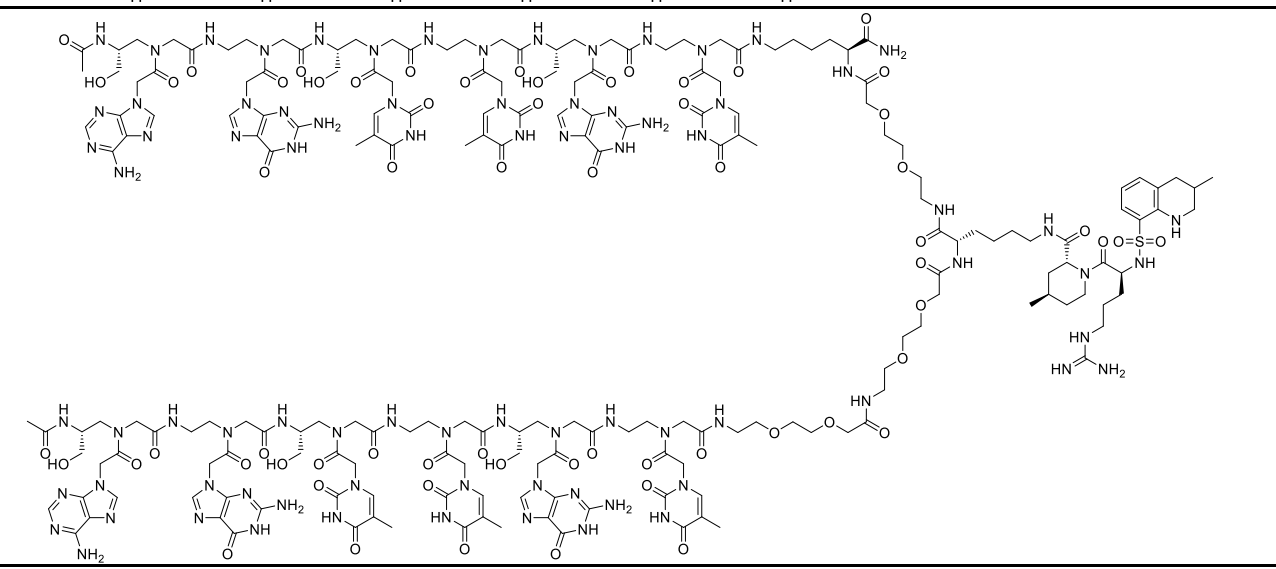	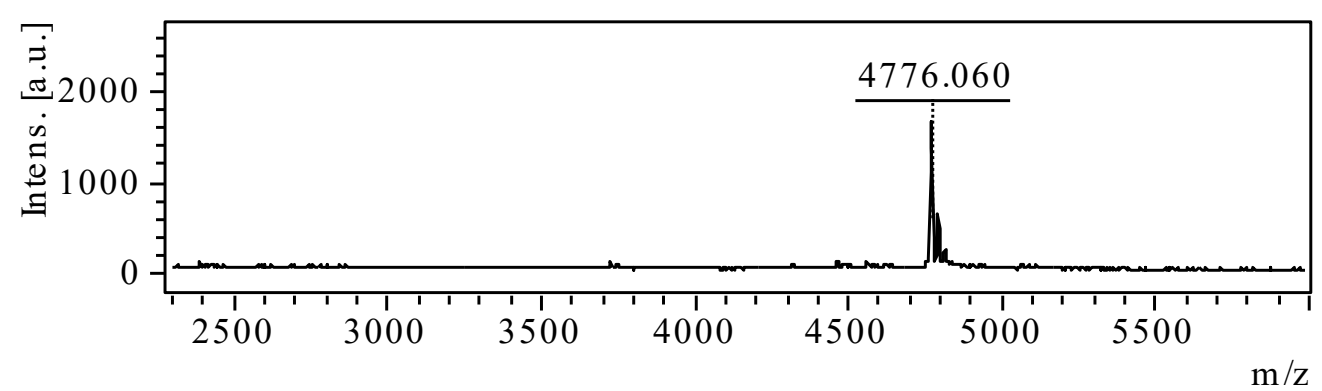
**AS-14**	$C_{216}H_{282}ClN_{91}O_{67}S$	5292.712	5291.093	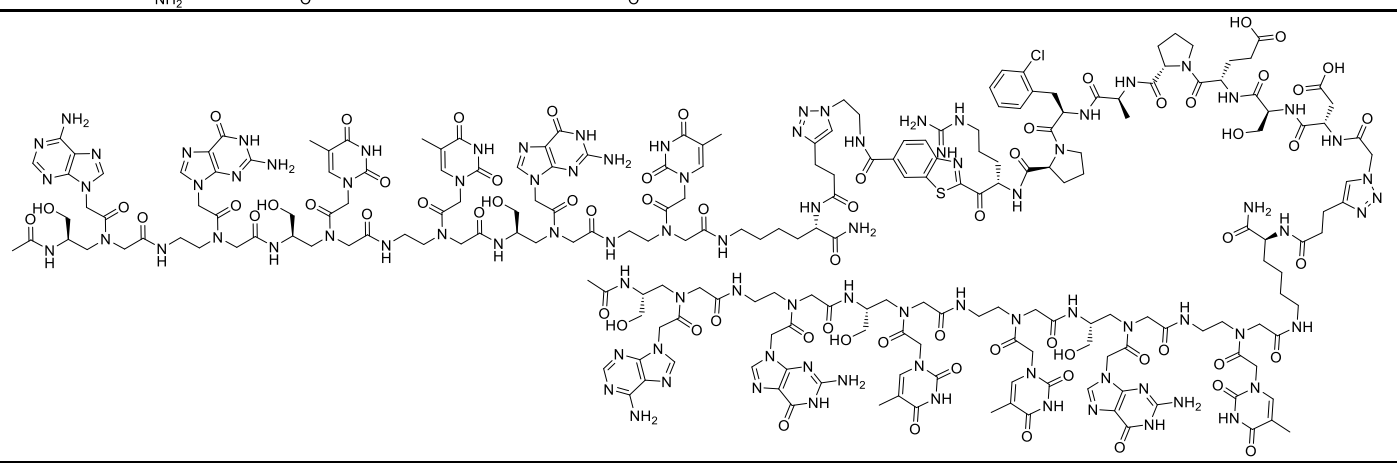	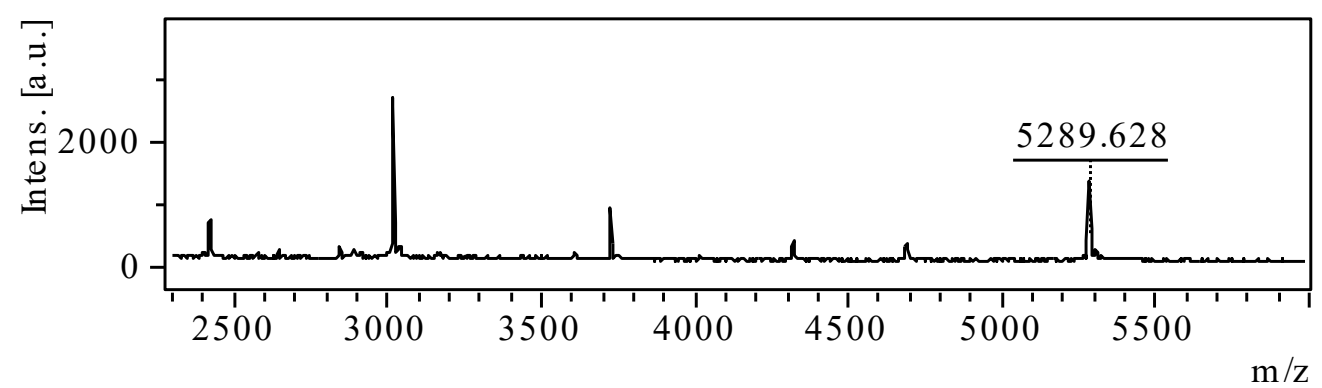

**AS-15**	$C_{212}H_{279}N_{91}O_{63}S$	5142.198	5141.121	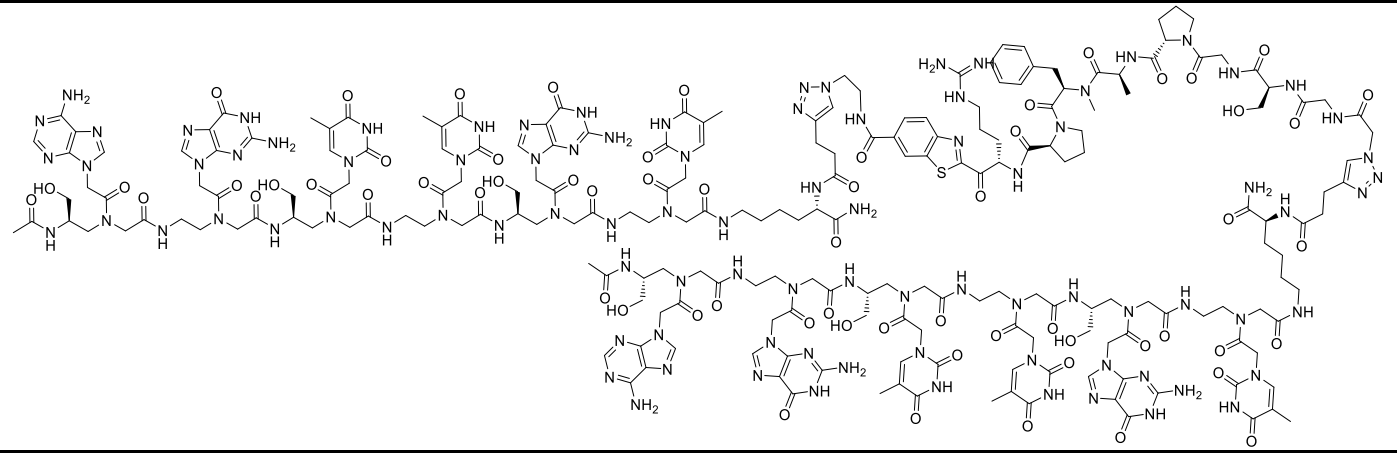	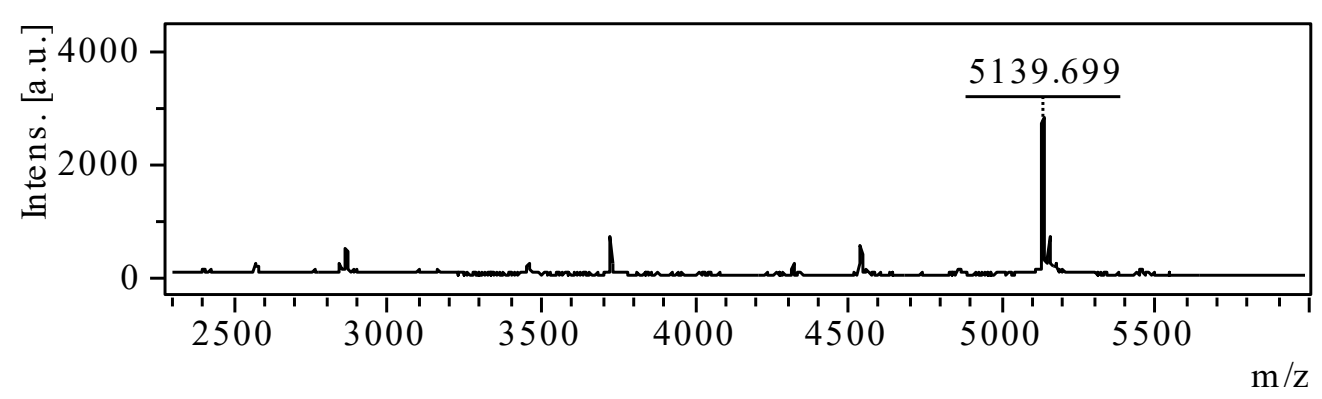
**AS-16**	$C_{211}H_{274}F_2N_{92}O_{65}S$	5209.149	5208.072 *5,192.082(Deg. of NO_2 on MALDI)	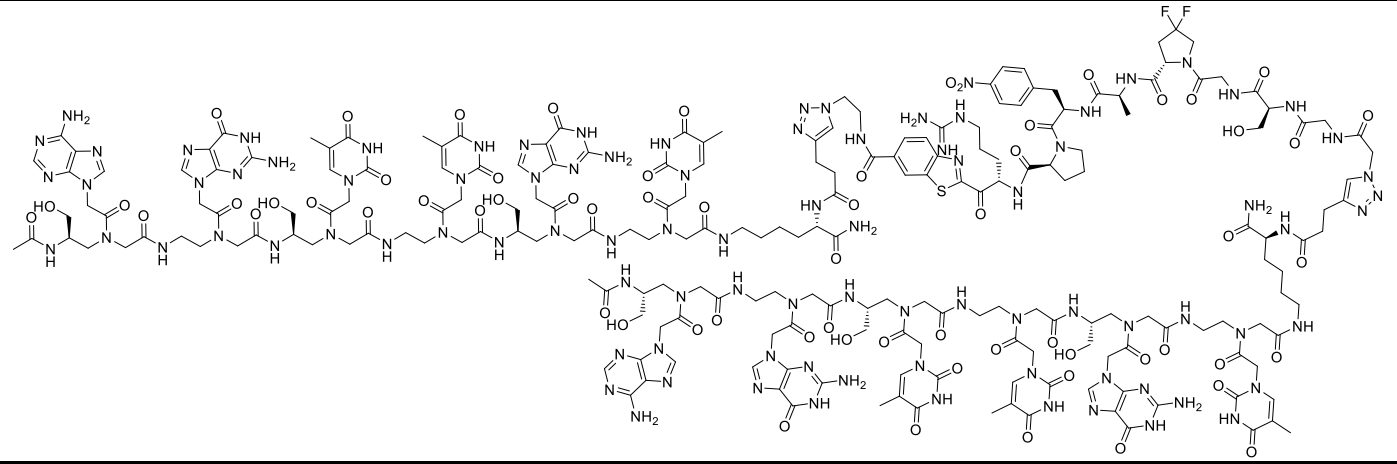	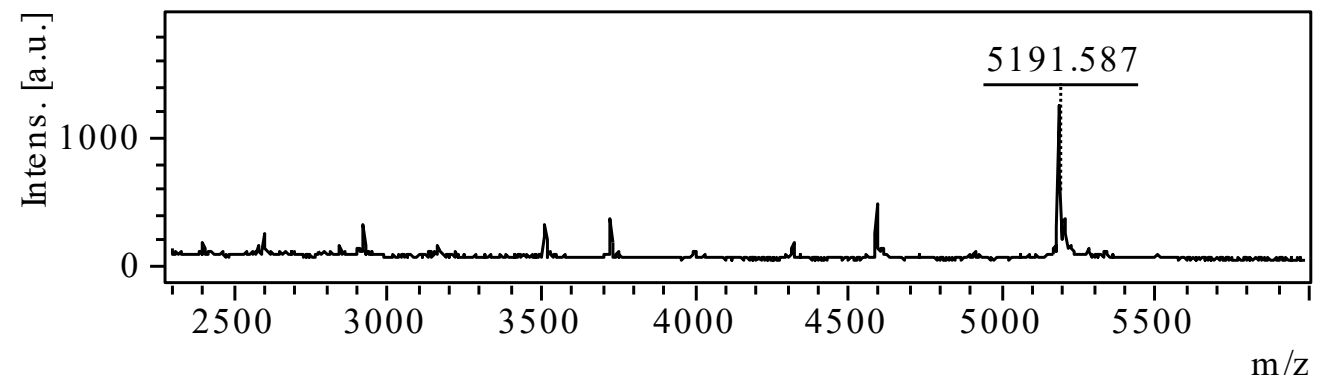
**AS-17**	$C_{205}H_{290}N_{86}O_{71}$	5095.106	5094.179	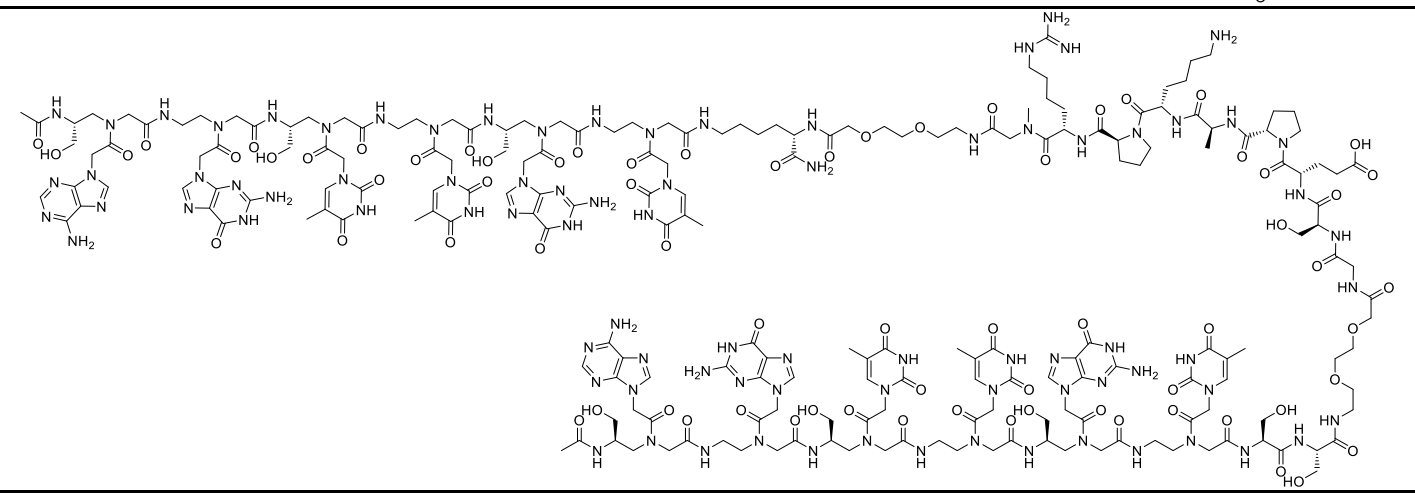	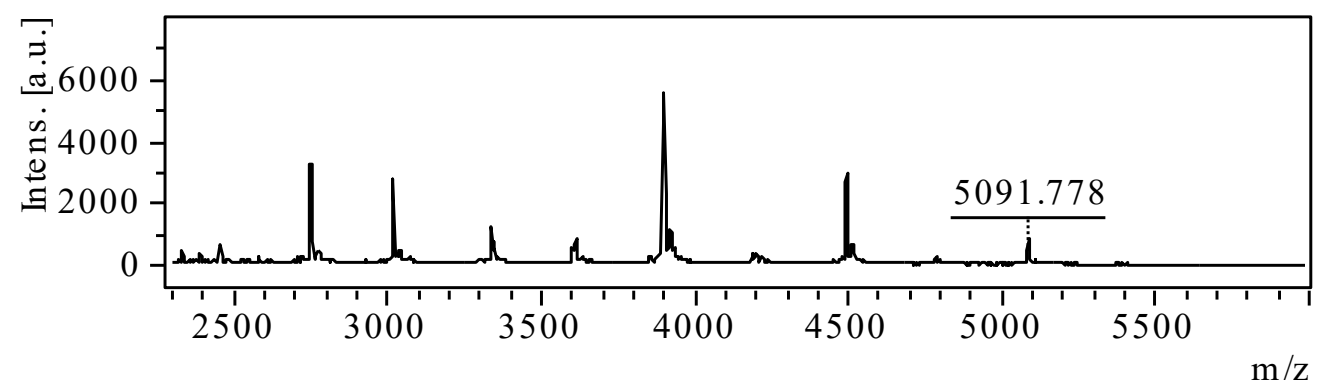
**AS-18**	$C_{219}H_{285}N_{91}O_{66}S$	5280.32	5279.153	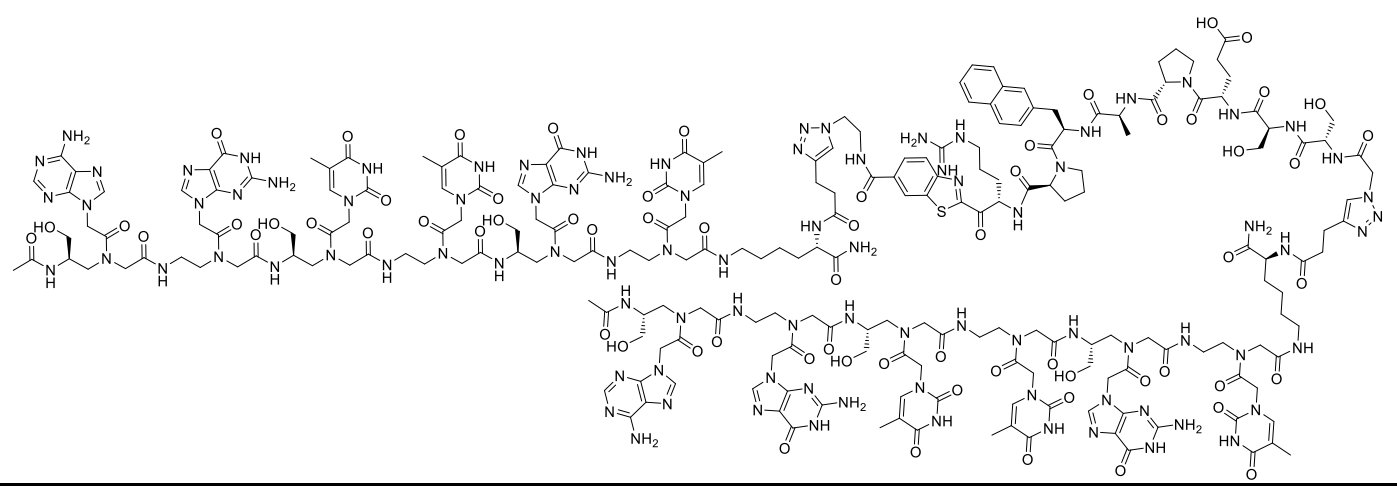	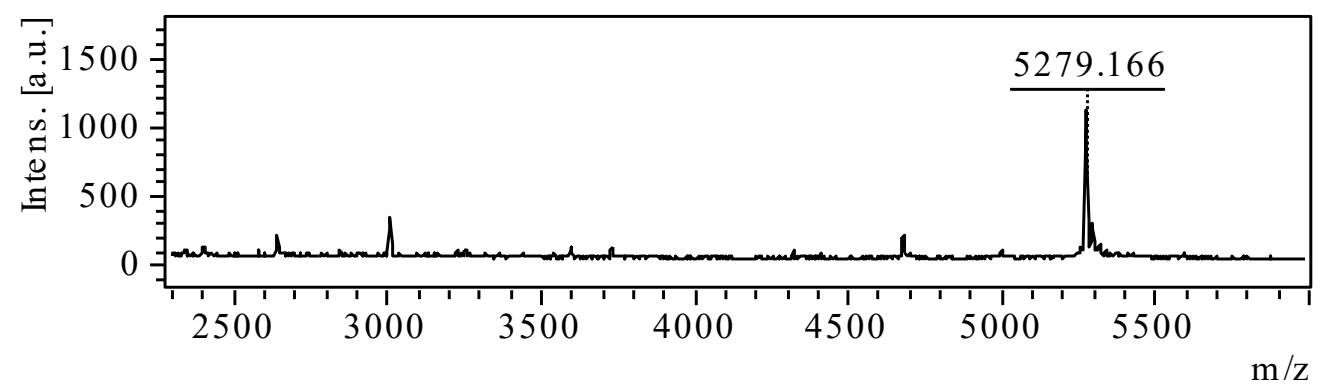

**AS-19**	$C_{197}H_{275}N_{81}O_{64}S$	4833.930	4833.054	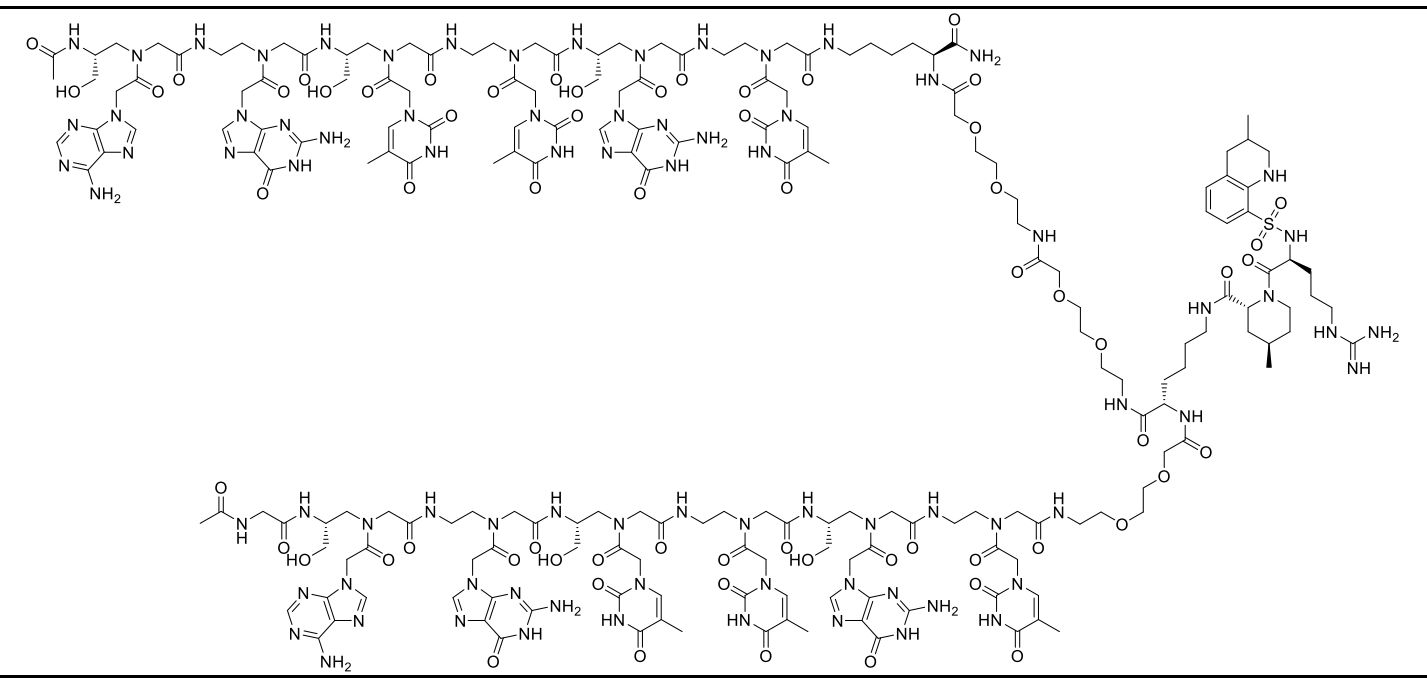	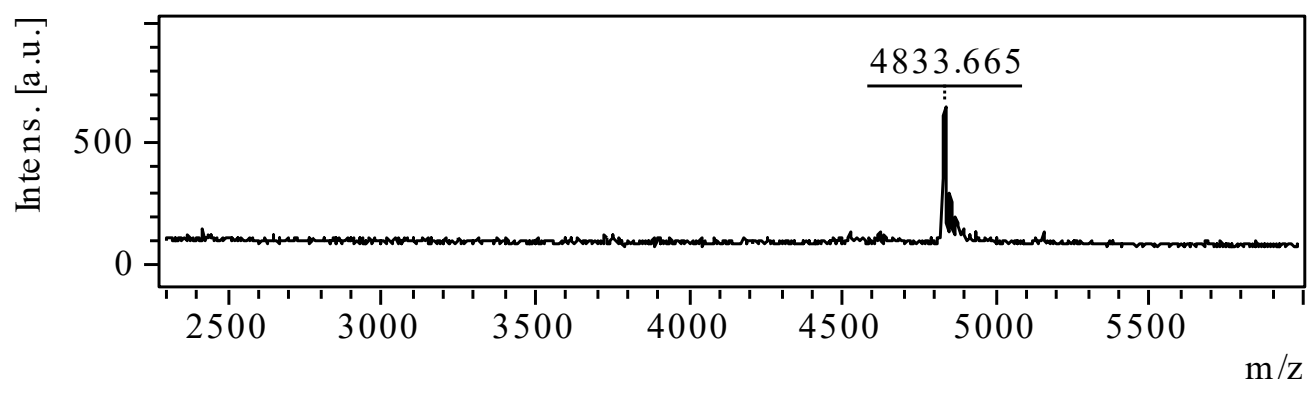
**AS-20**	$C_{208}H_{294}N_{86}O_{71}$	5135.171	5134.211	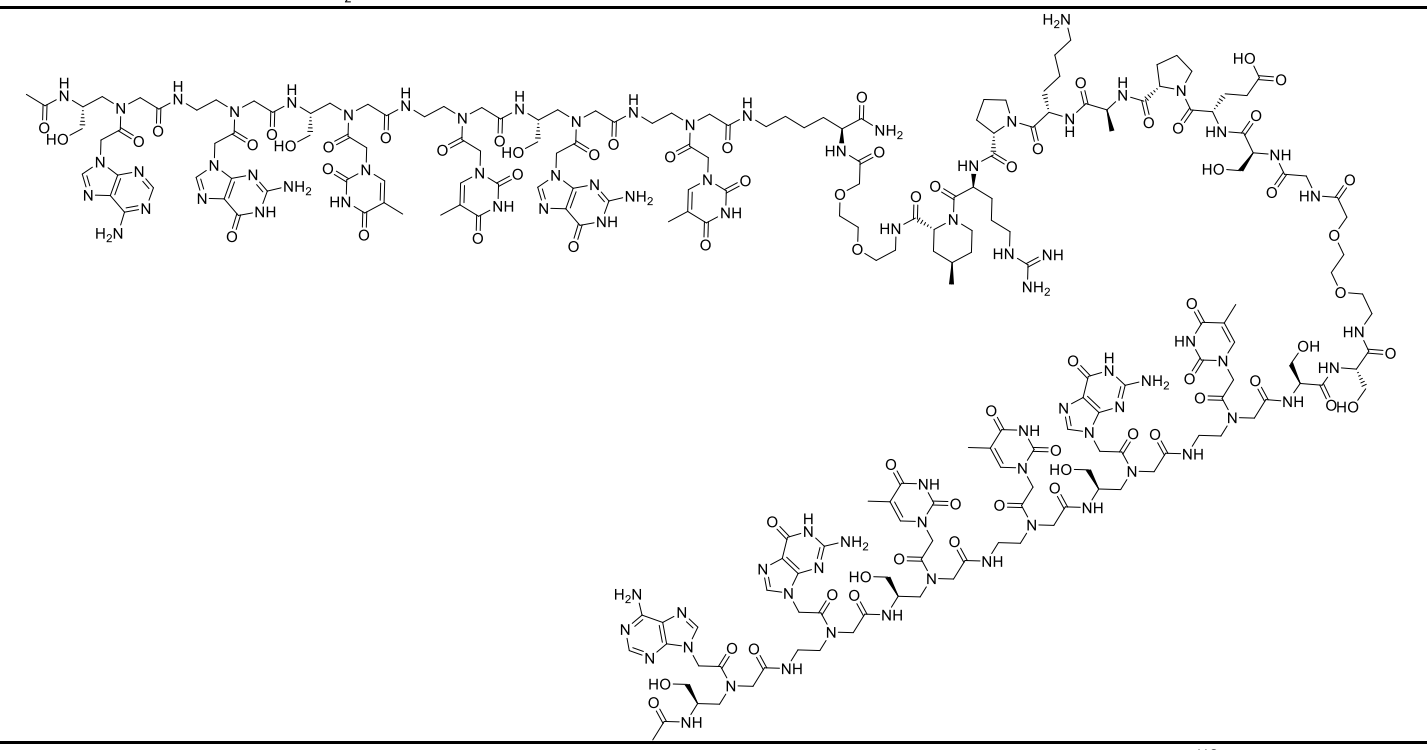	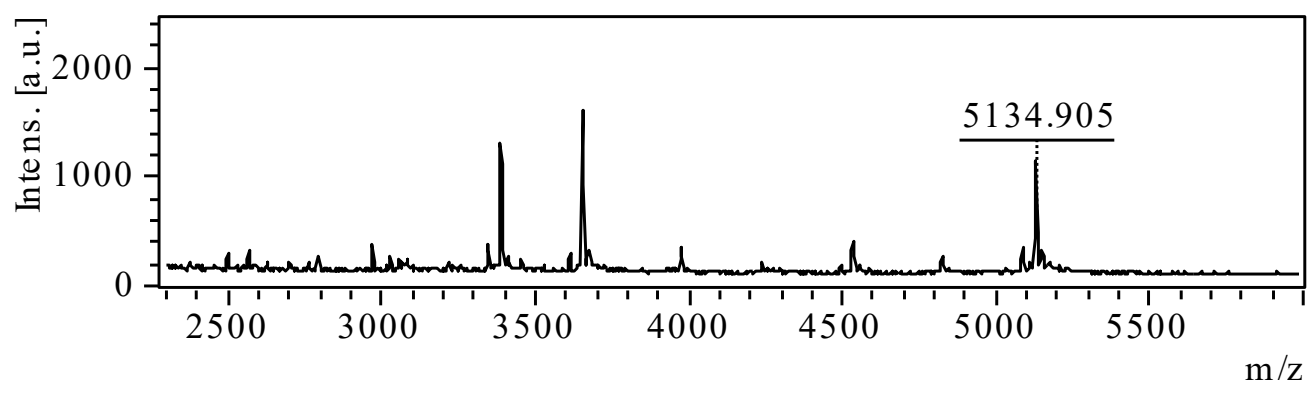
**AS-21**	$C_{211}H_{291}N_{85}O_{71}$	5154.173	5153.184	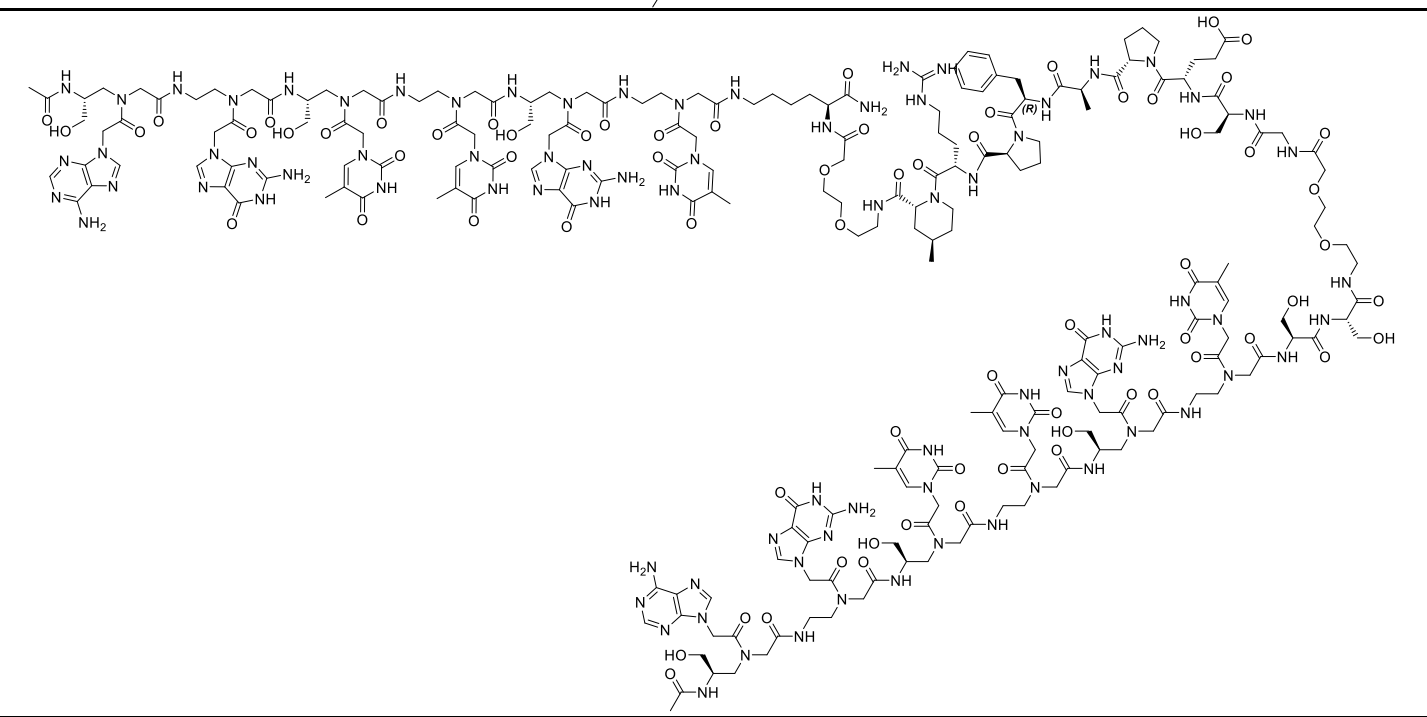	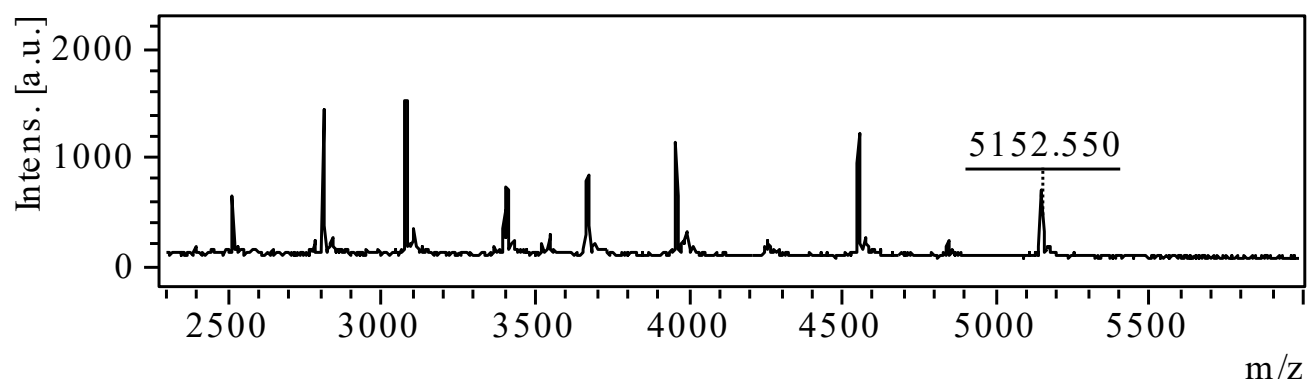

**AS-22**	$C_{212}H_{293}N_{83}O_{75}$	5204.182	5203.173	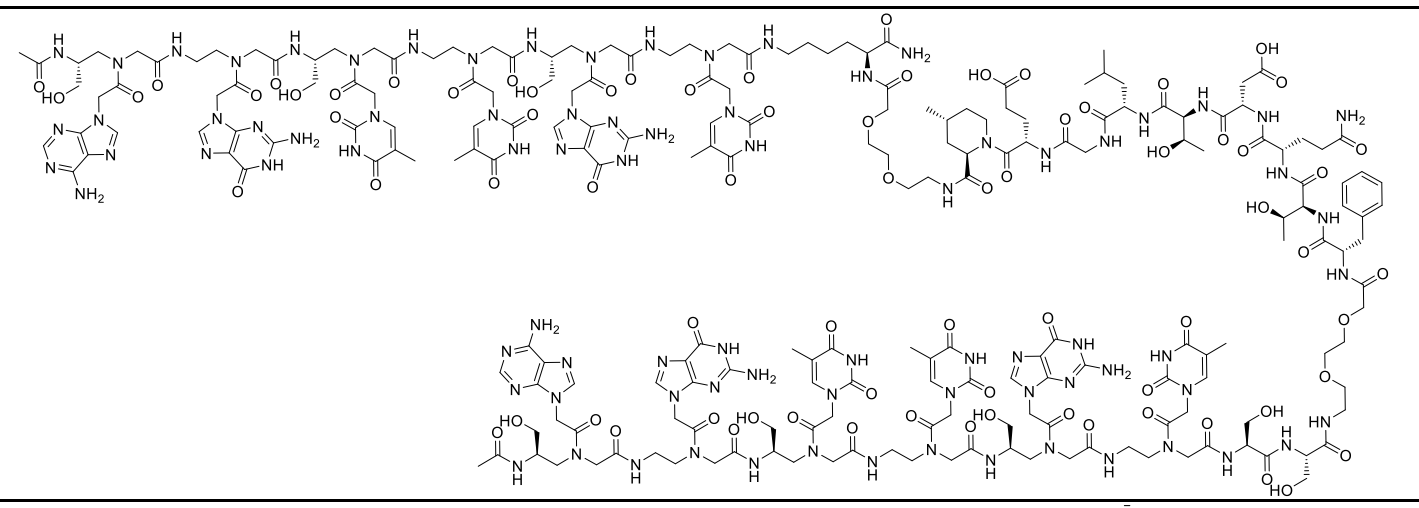	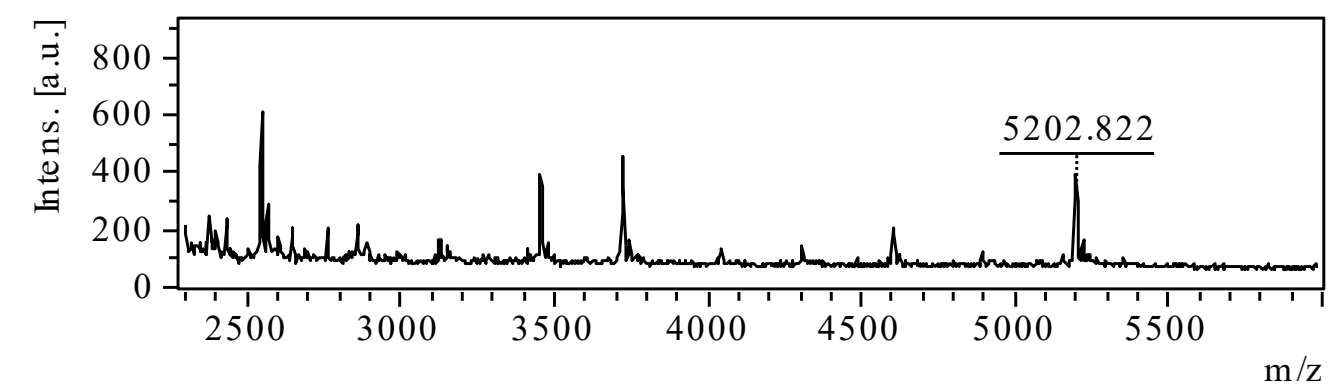
**AS-23**	$C_{210}H_{290}N_{84}O_{75}$	5191.143	5190.153	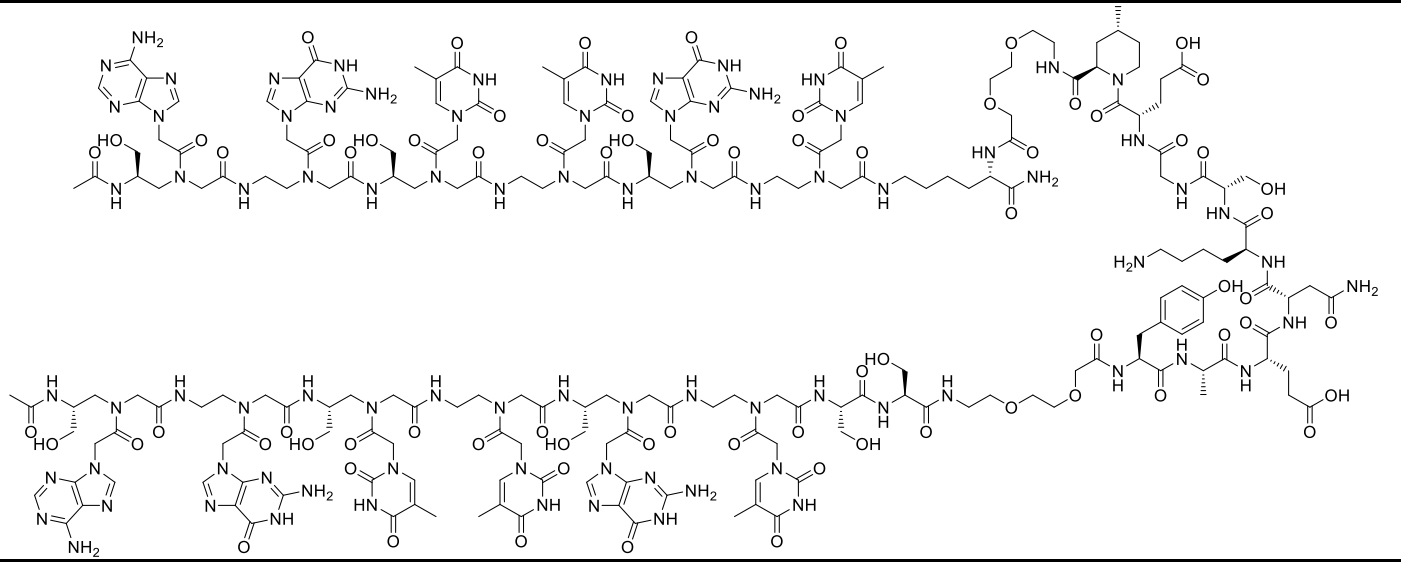	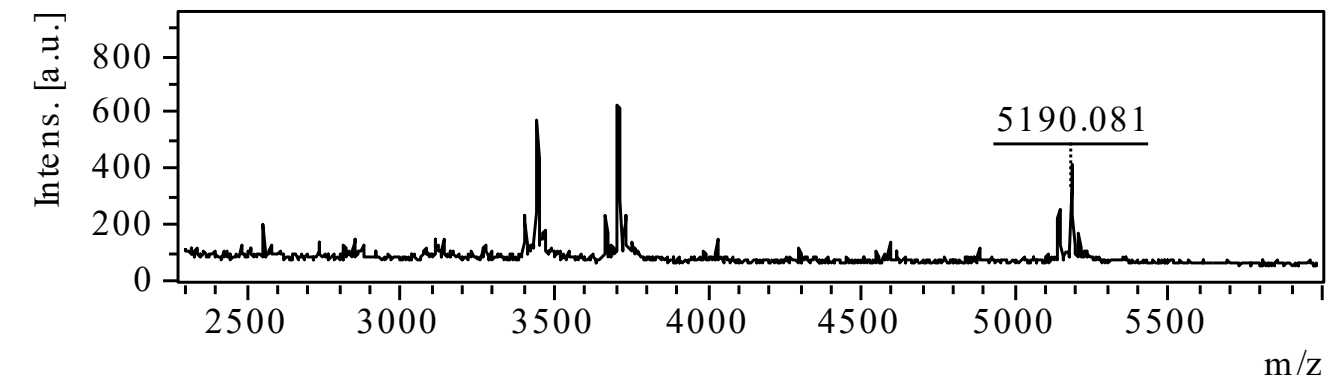
**AS-24**	$C_{212}H_{295}ClN_{88}O_{69}$	5215.689	5214.204	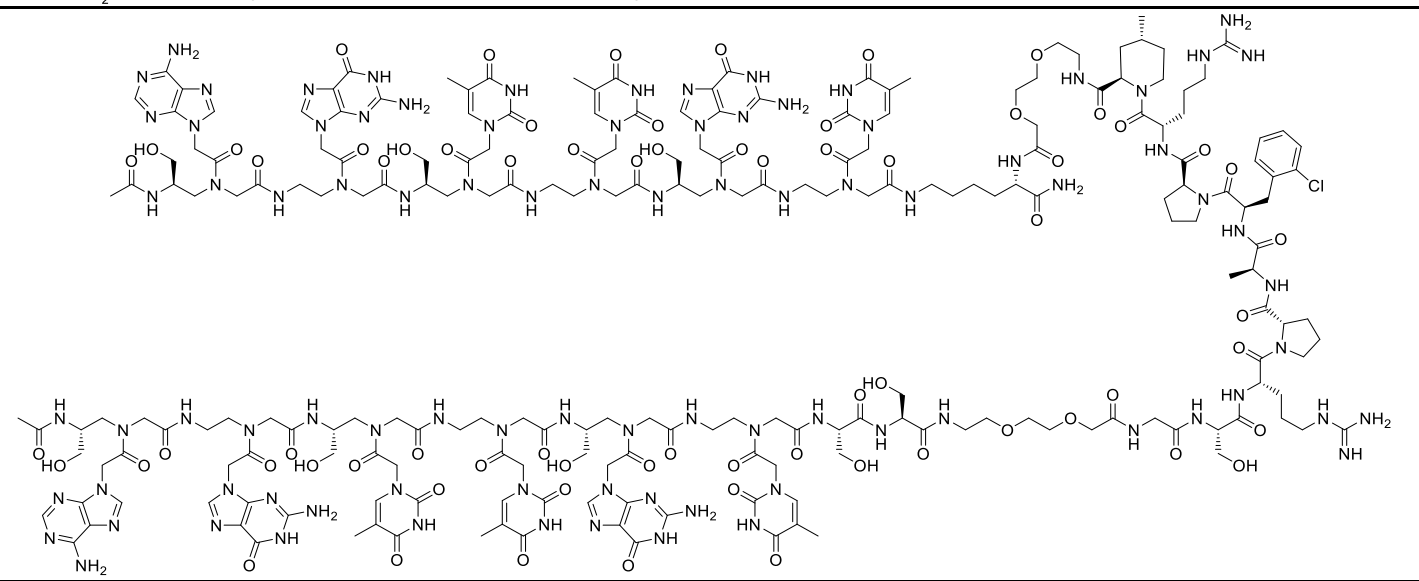	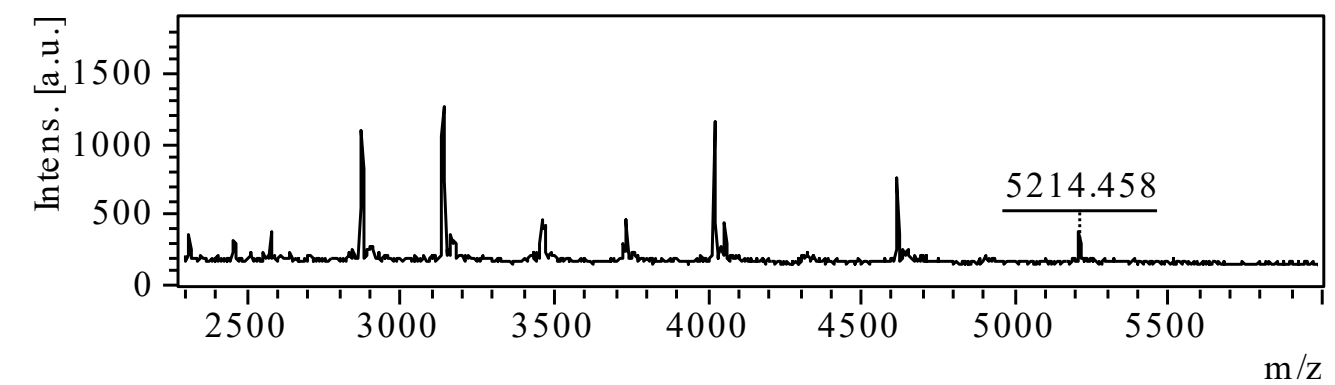
**AS-25**	$C_{216}H_{302}N_{88}O_{70}$	5251.338	5250.284	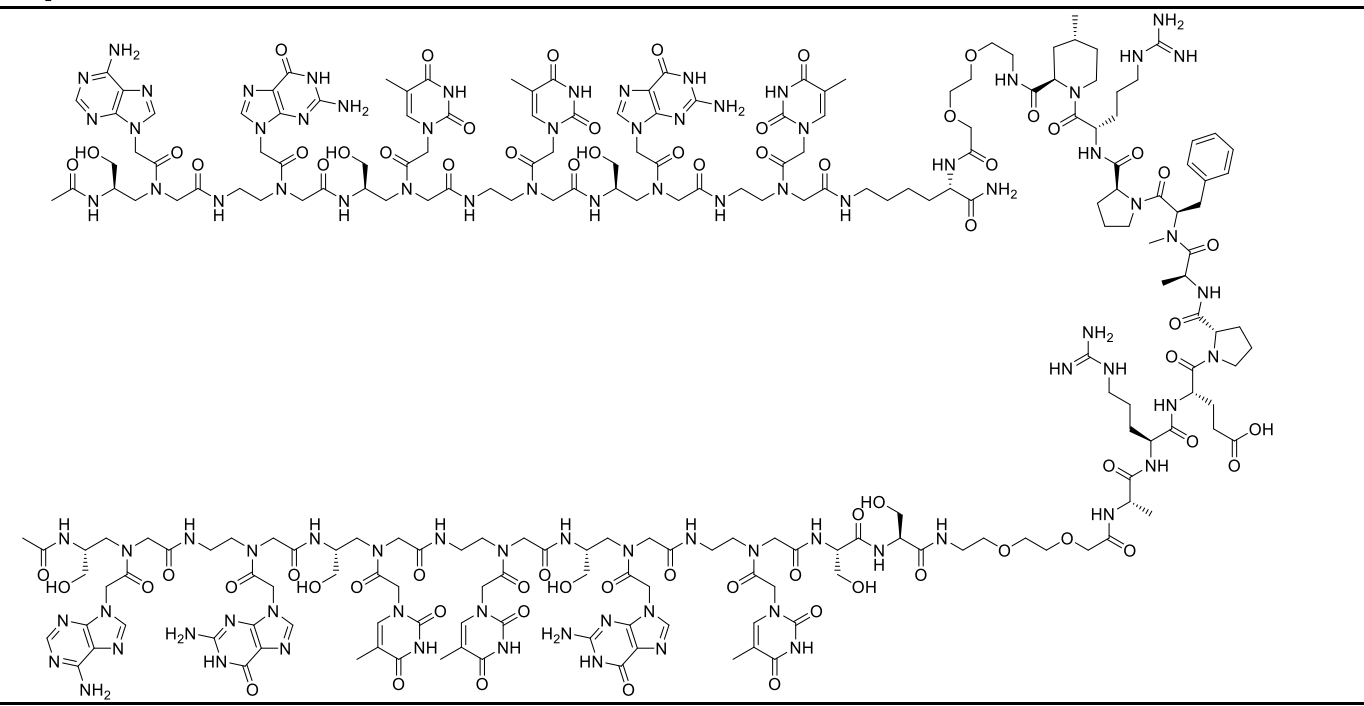	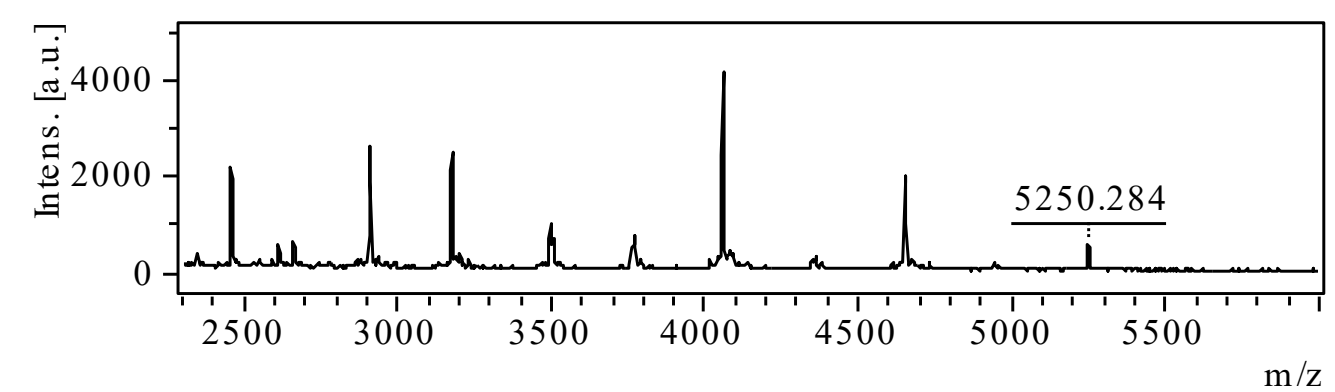

**AS-26**	$C_{211}H_{290}N_{86}O_{73}$	5199.17	5198.169 *5,182.179 (Deg. of NO_2 on MALDI)	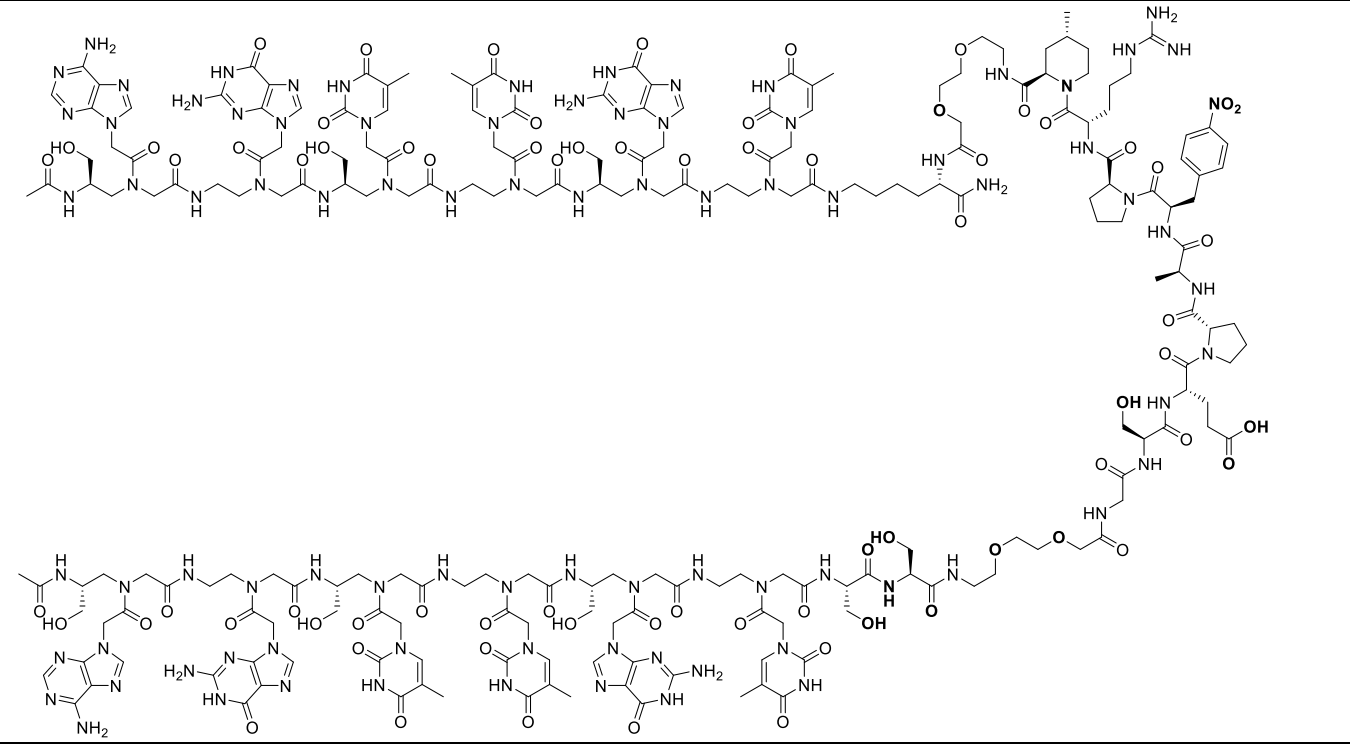	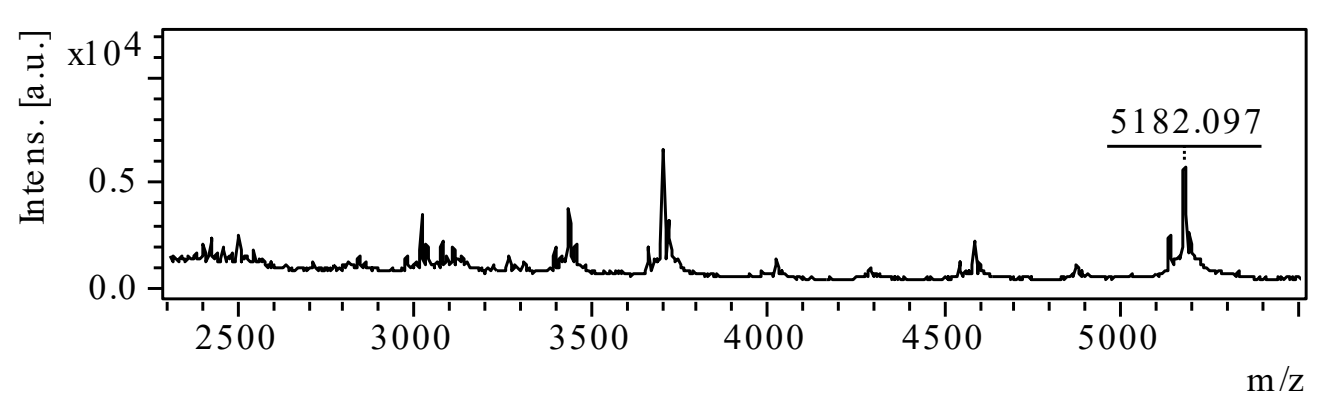
**AS-27**	$C_{211}H_{288}ClF_2N_{85}O_{71}$	5224.596	5223.126	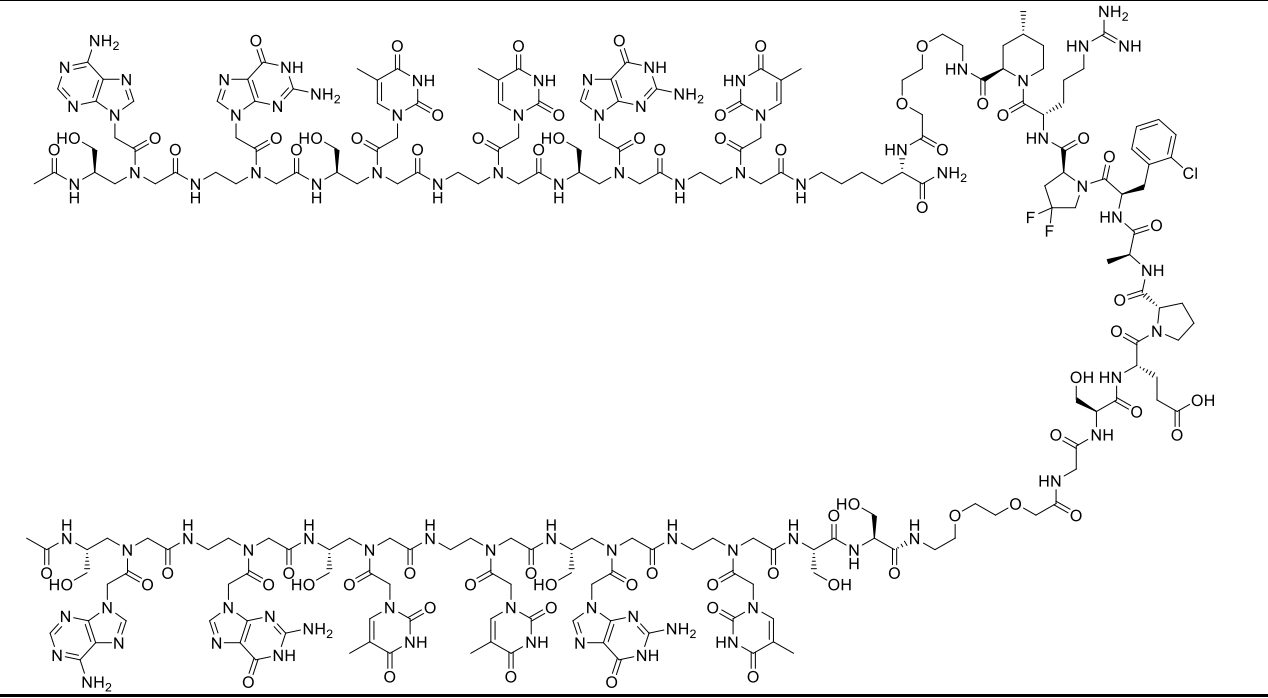	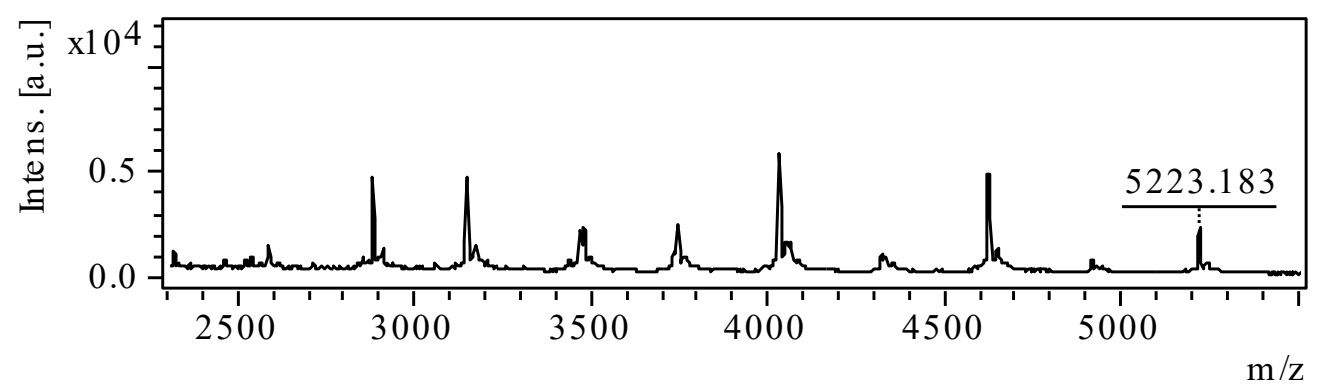
**AS-28**	$C_{166}H_{226}N_{72}O_{58}$	4158.08	4156.698	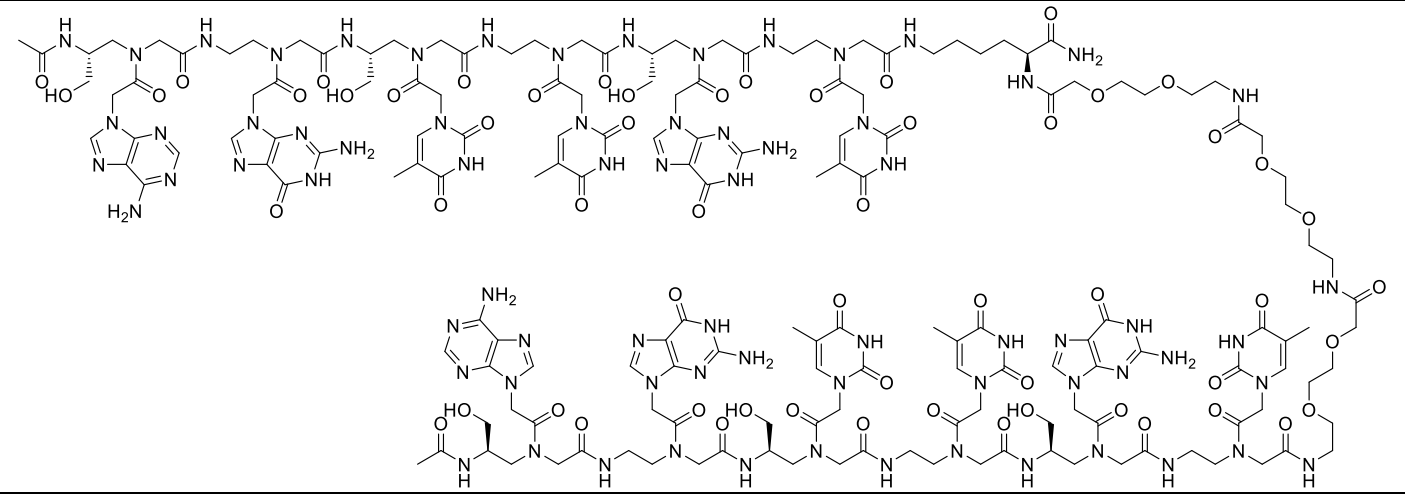	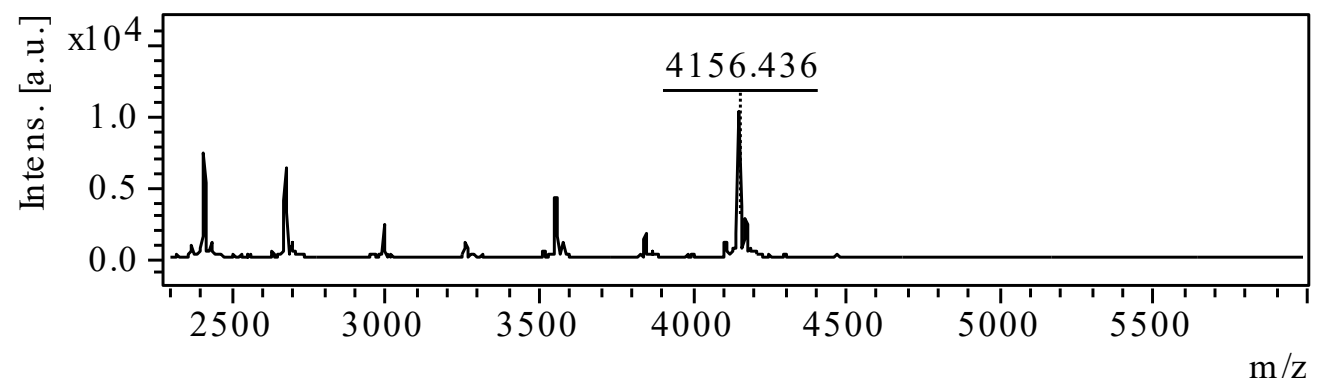

**AS-29**	$C_{160}H_{215}N_{71}O_{55}$	4012.922	4011.624	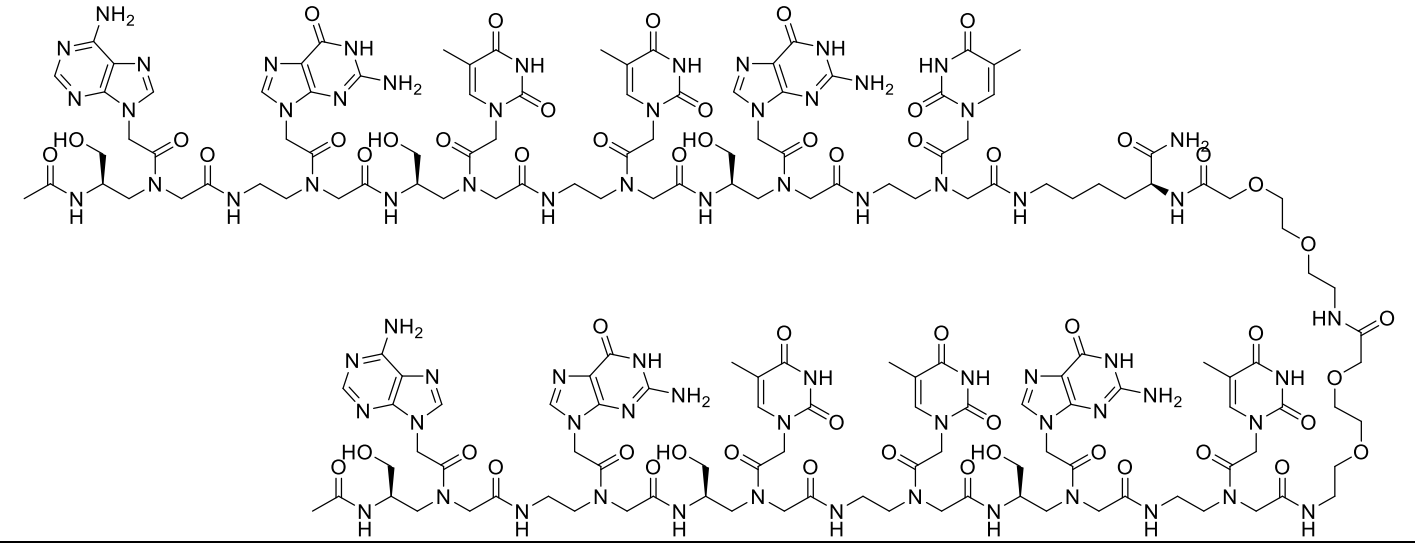	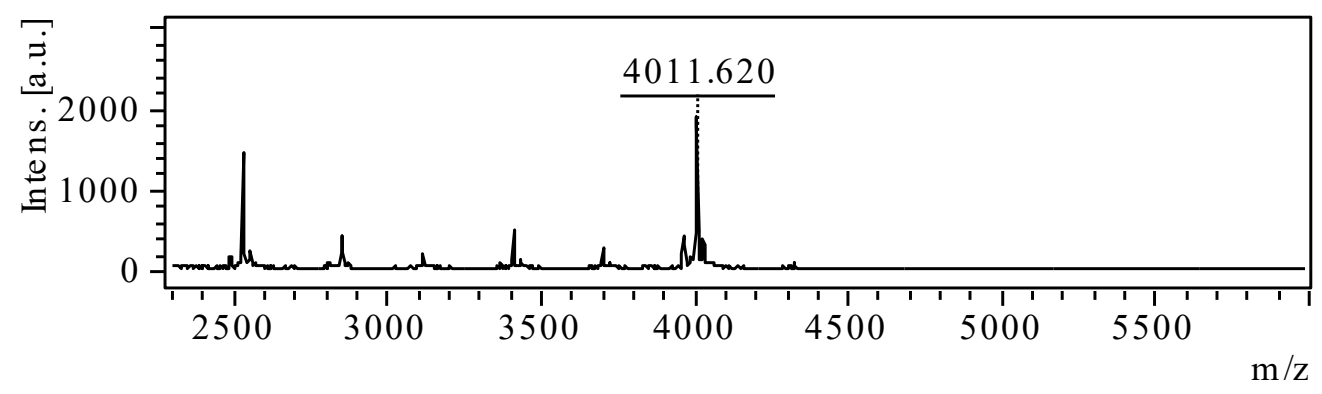
**AS-30**	$C_{172}H_{237}N_{73}O_{61}$	4303.238	4301.772	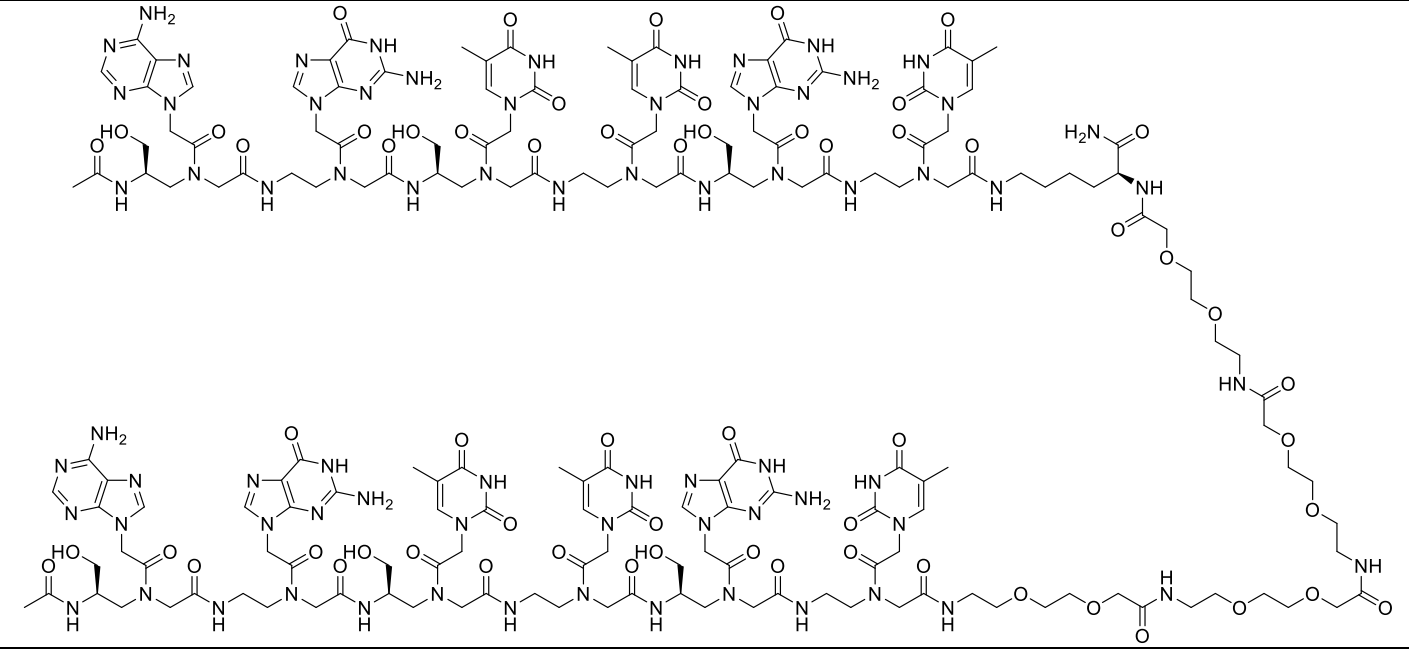	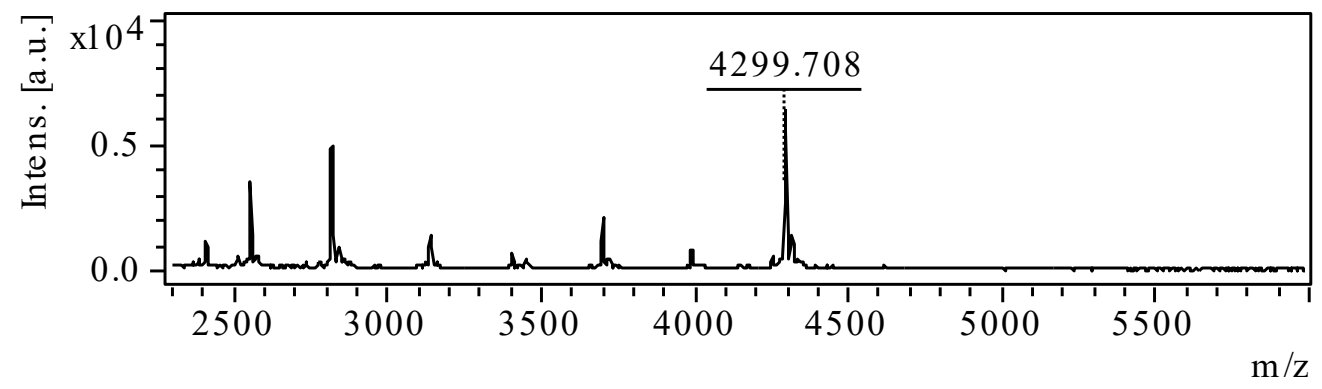
**AS-31**	$C_{191}H_{250}N_{80}O_{58}$	4594.6	4593.914	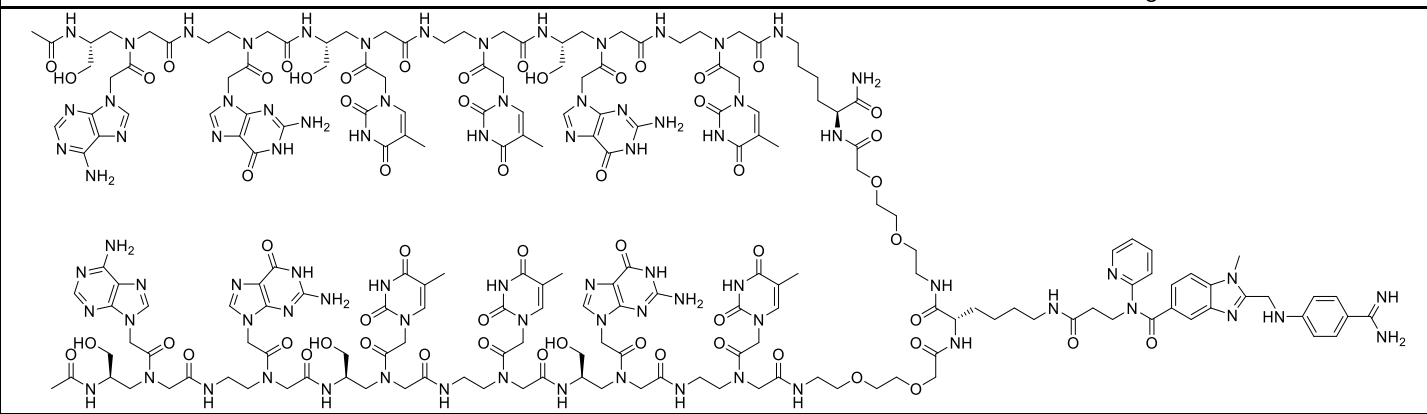	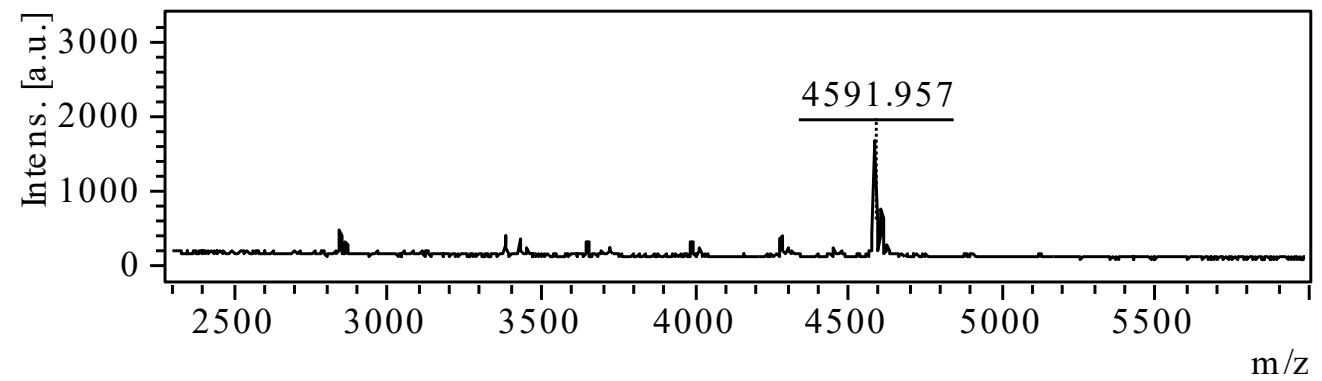

**AS-32**	$C_{222}H_{314}N_{90}O_{74}$	5427.51	5426.364	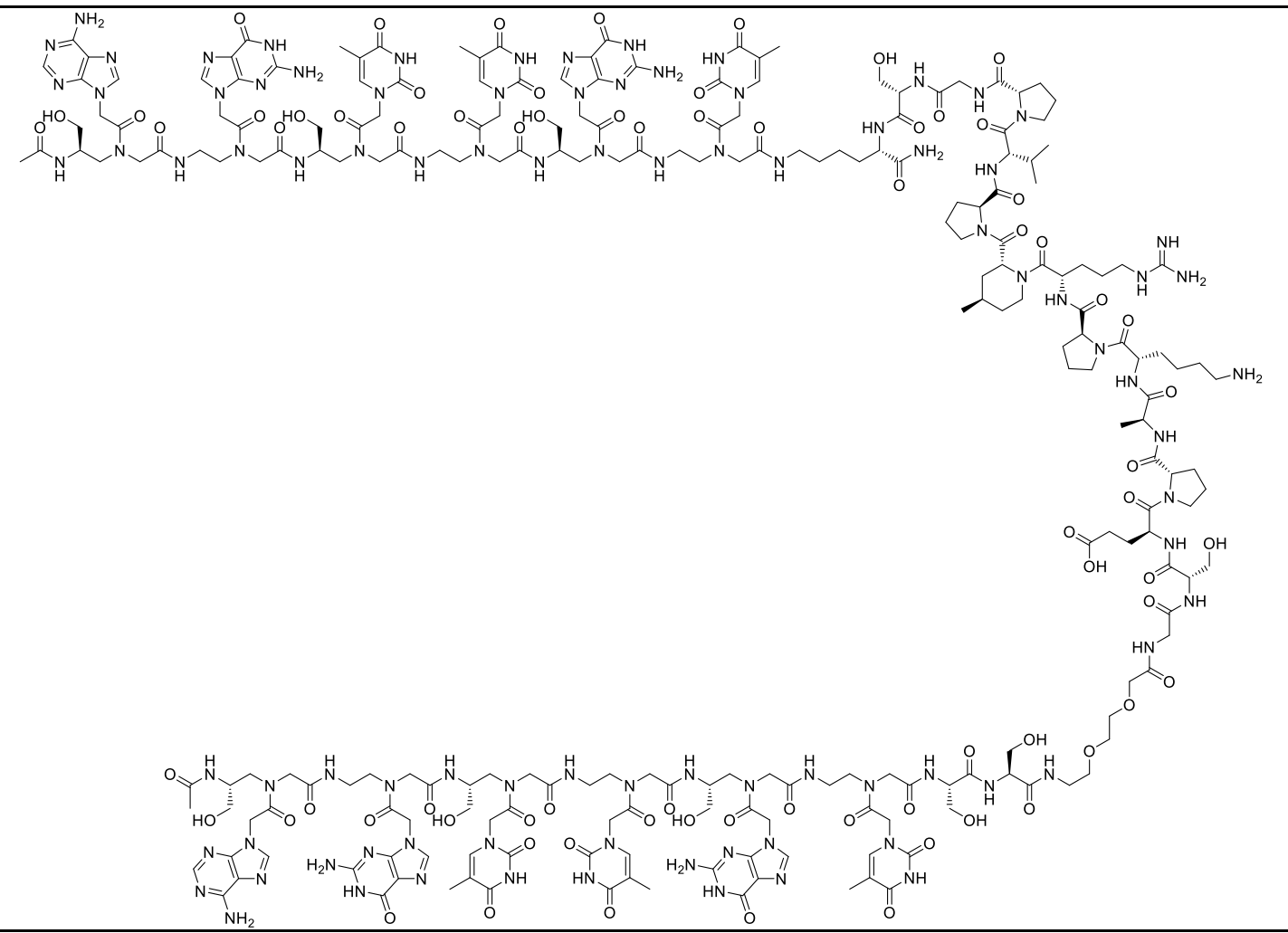	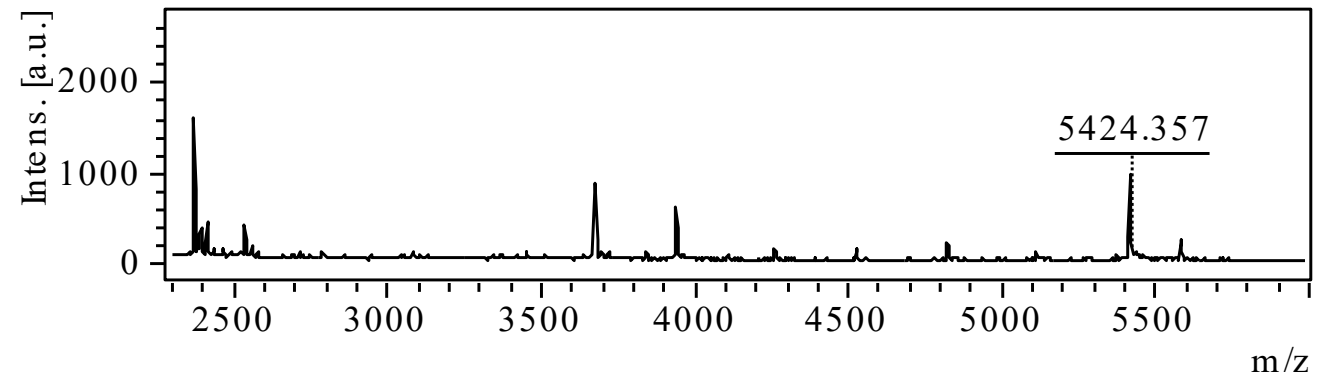
**AS-33**	$C_{197}H_{261}N_{81}O_{61}$	4739.761	4738.988	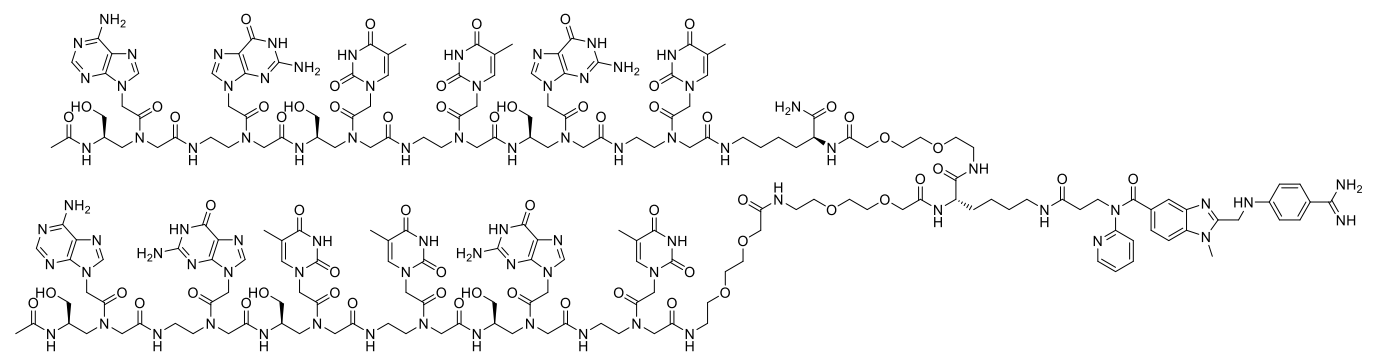	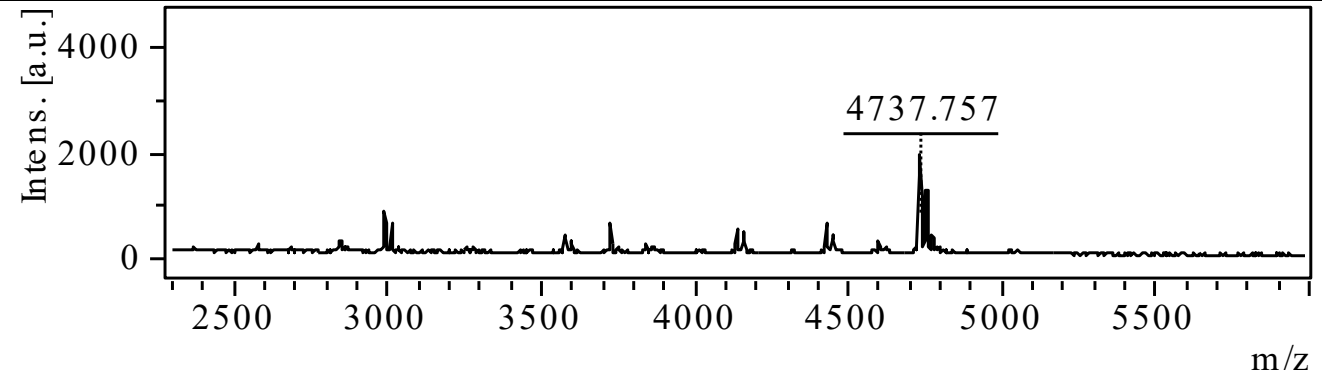
**AS-34**	$C_{215}H_{309}N_{87}O_{75}$	5312.371	5311.311	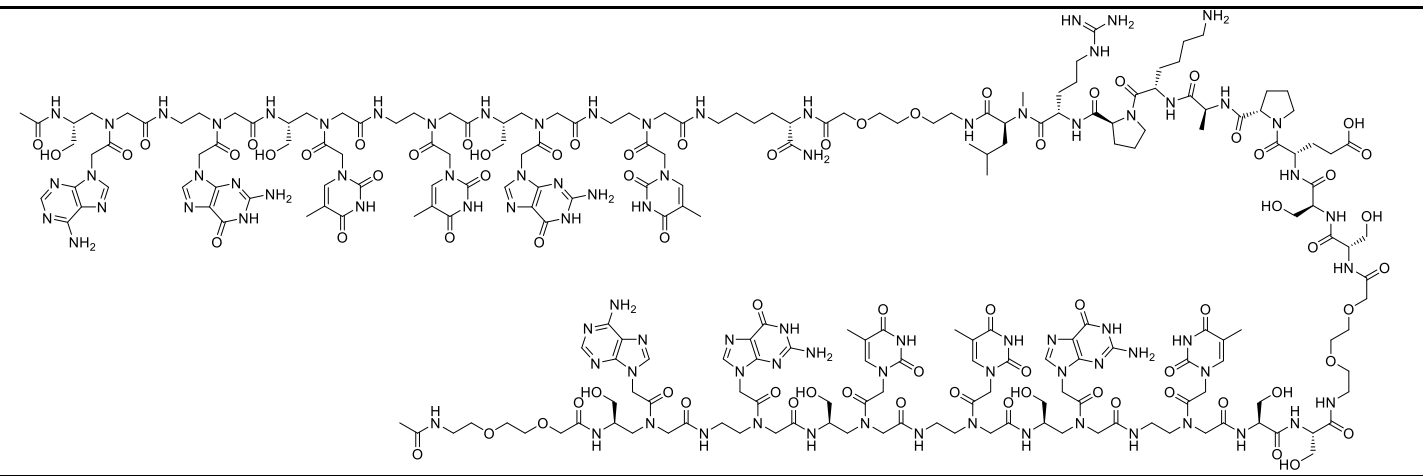	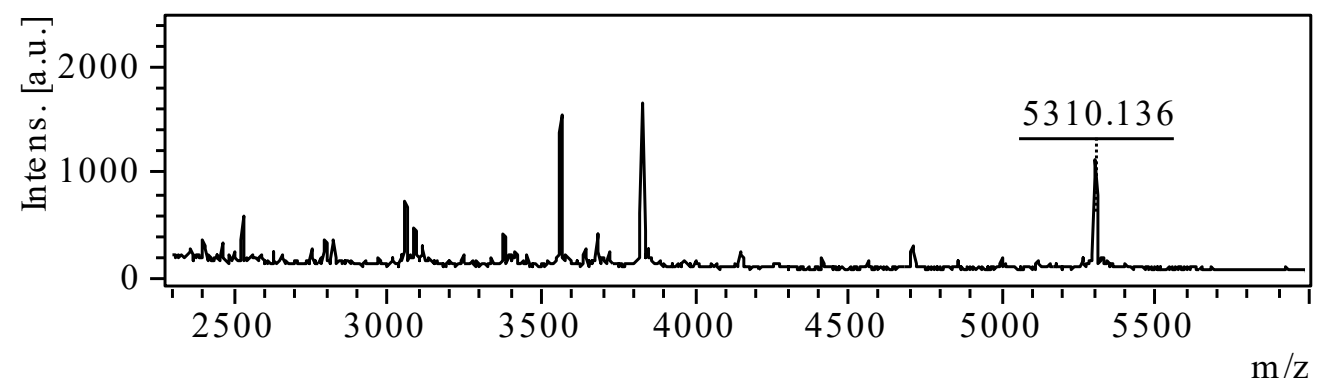

**AS-35**	$C_{208}H_{289}N_{85}O_{69}$	5084.126	5083.179	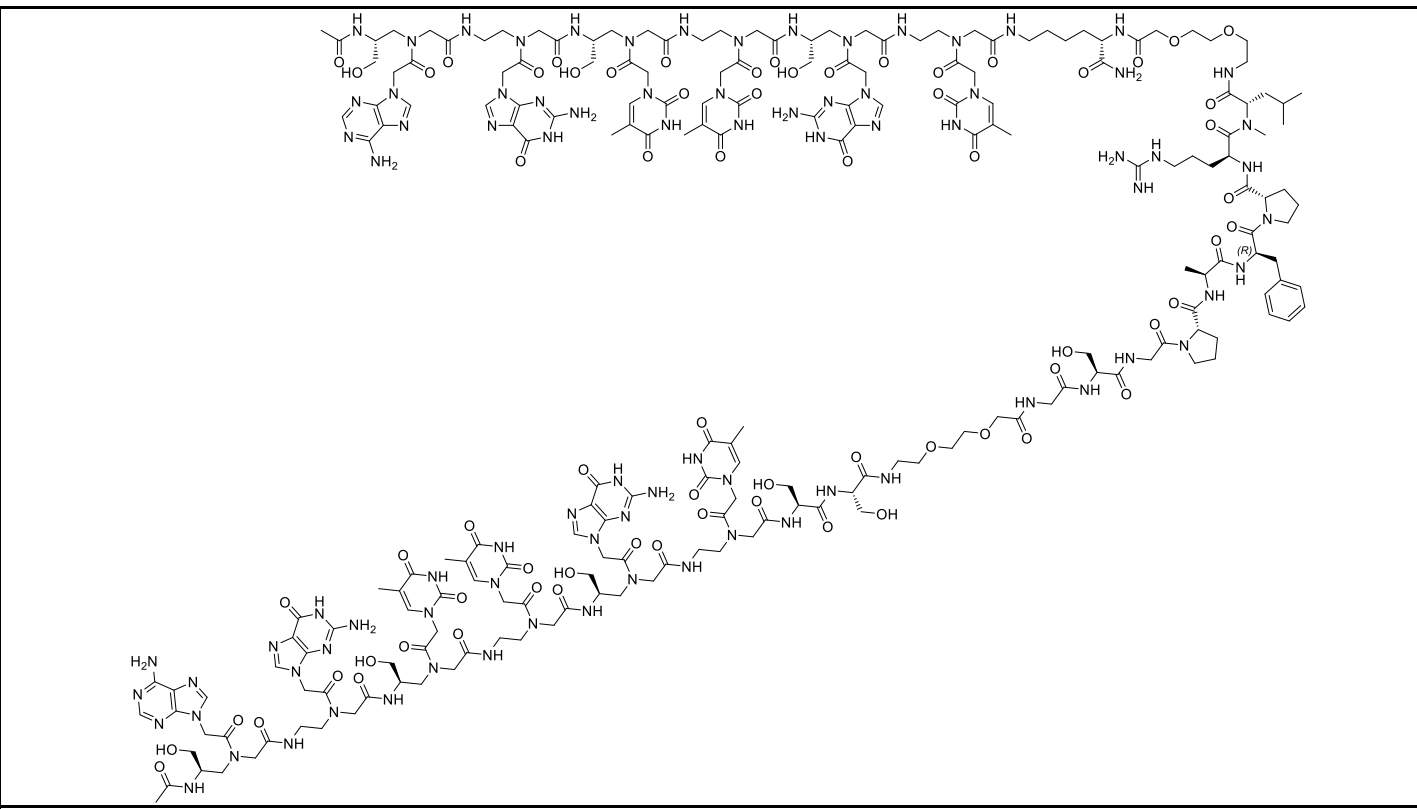	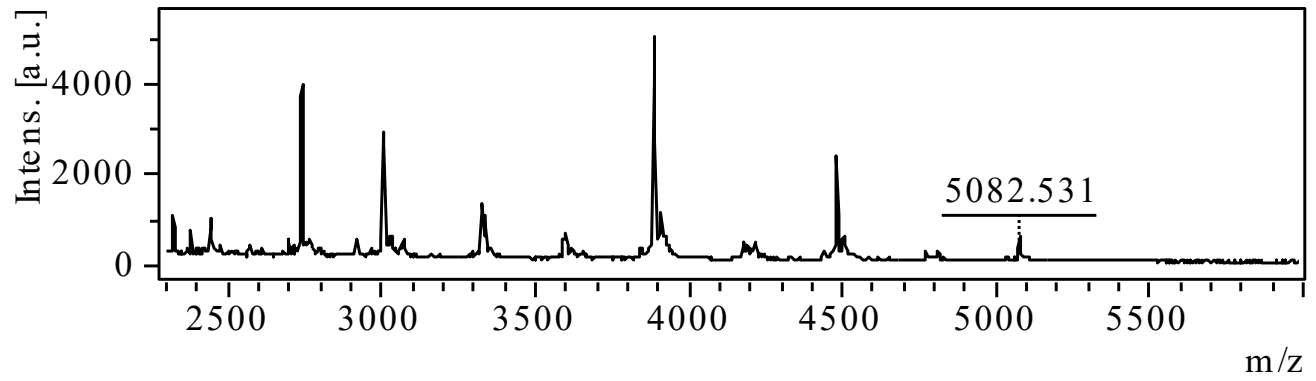
**AS-36**	$C_{199}H_{264}N_{82}O_{62}$	4796.813	4796.009	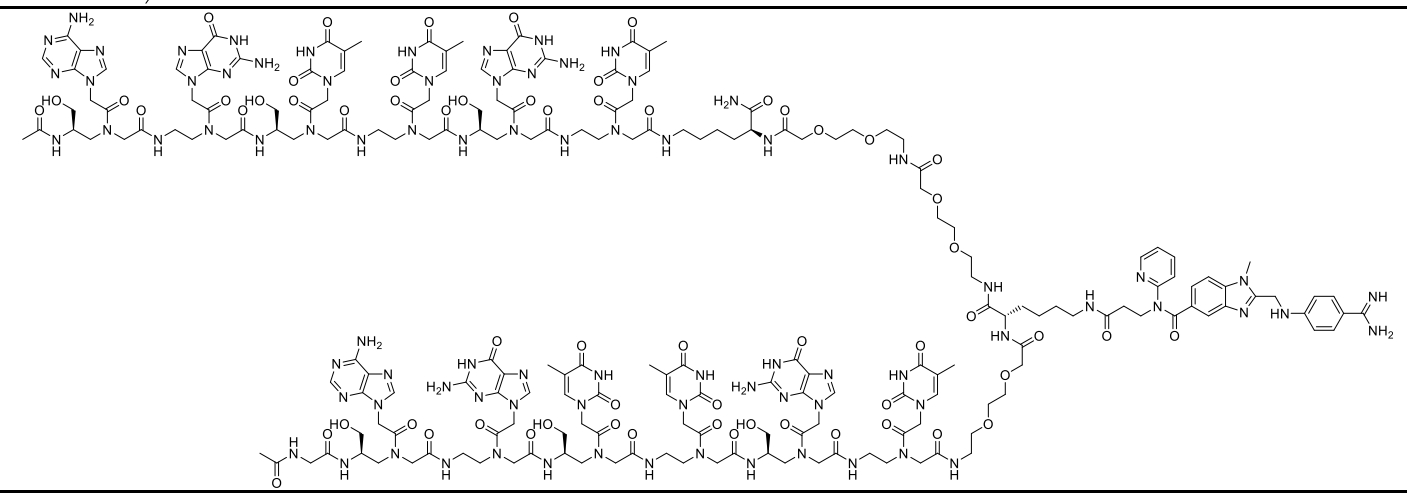	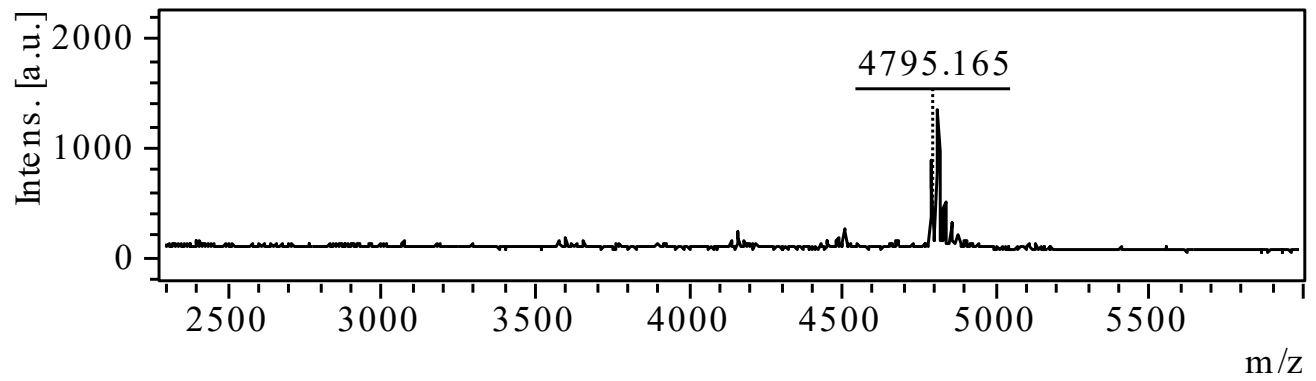
**AS-37**	$C_{216}H_{304}N_{86}O_{76}$	5321.334	5320.263	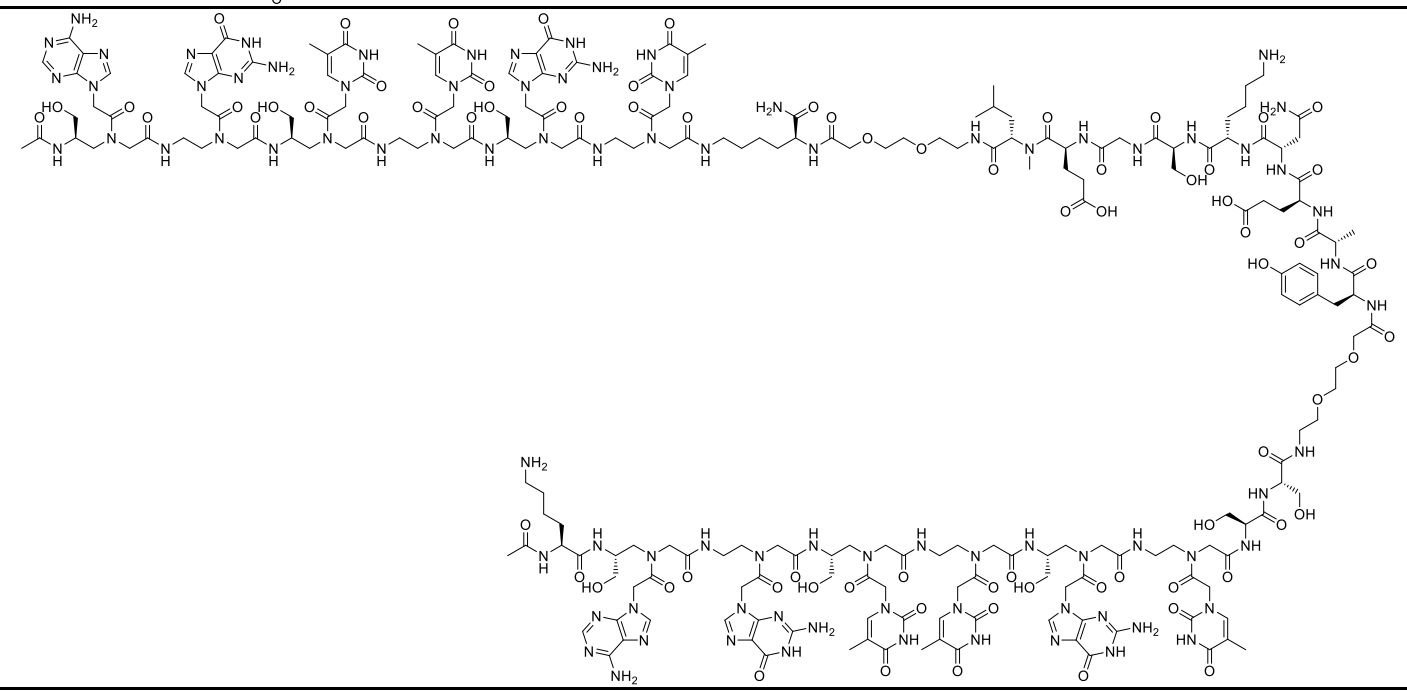	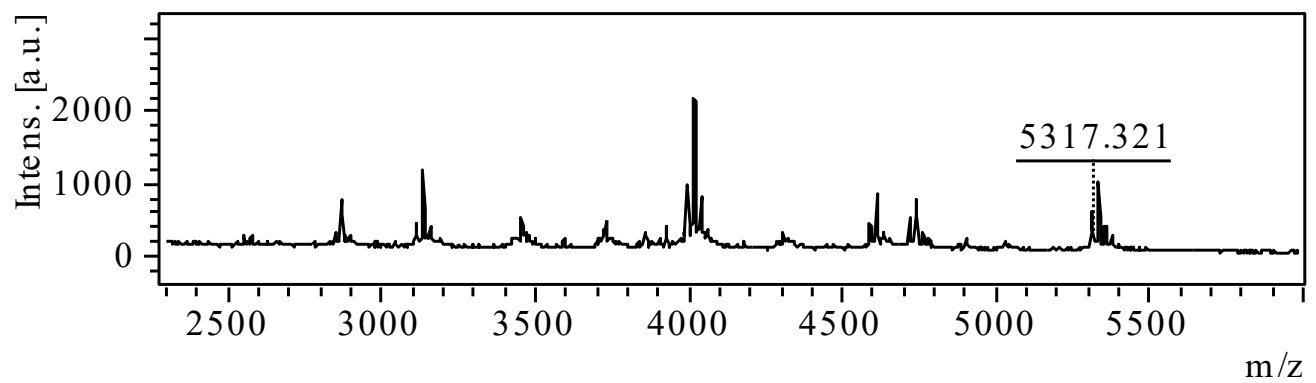
**AS-38**	$C_{203}H_{272}N_{82}O_{64}$	4884.919	4884.062	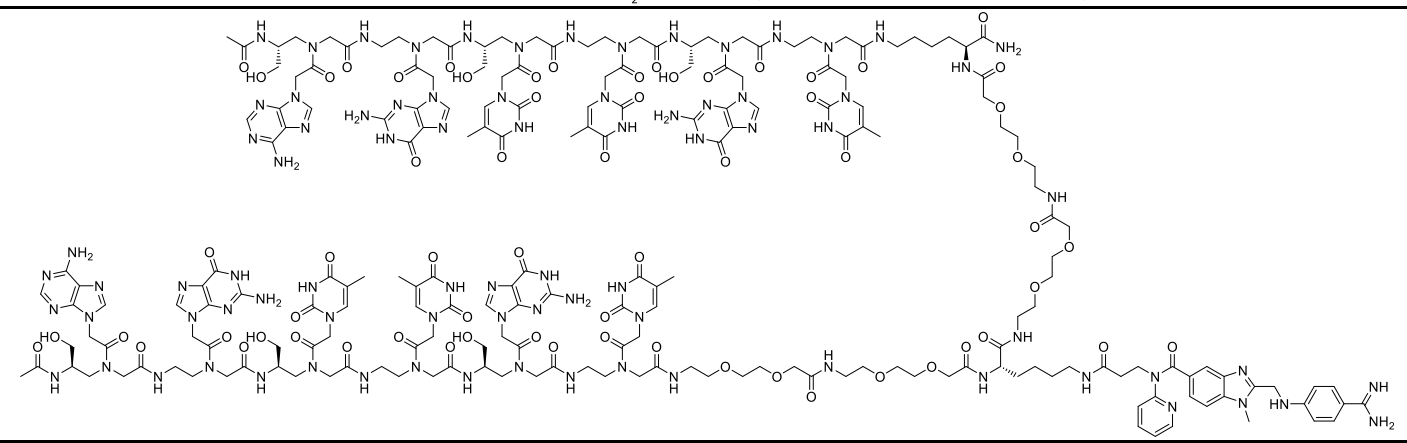	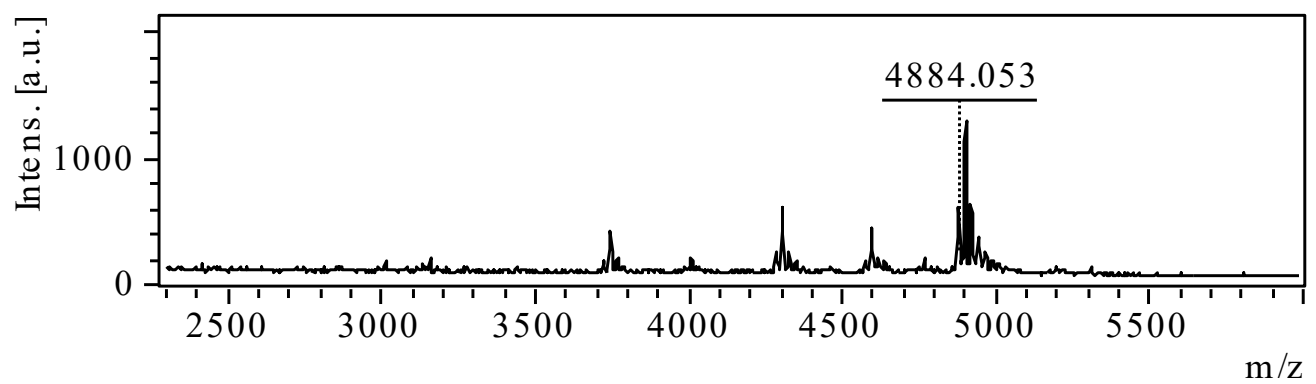

**AS-39**	$C_{223}H_{323}N_{95}O_{72}$	5486.63	5485.460	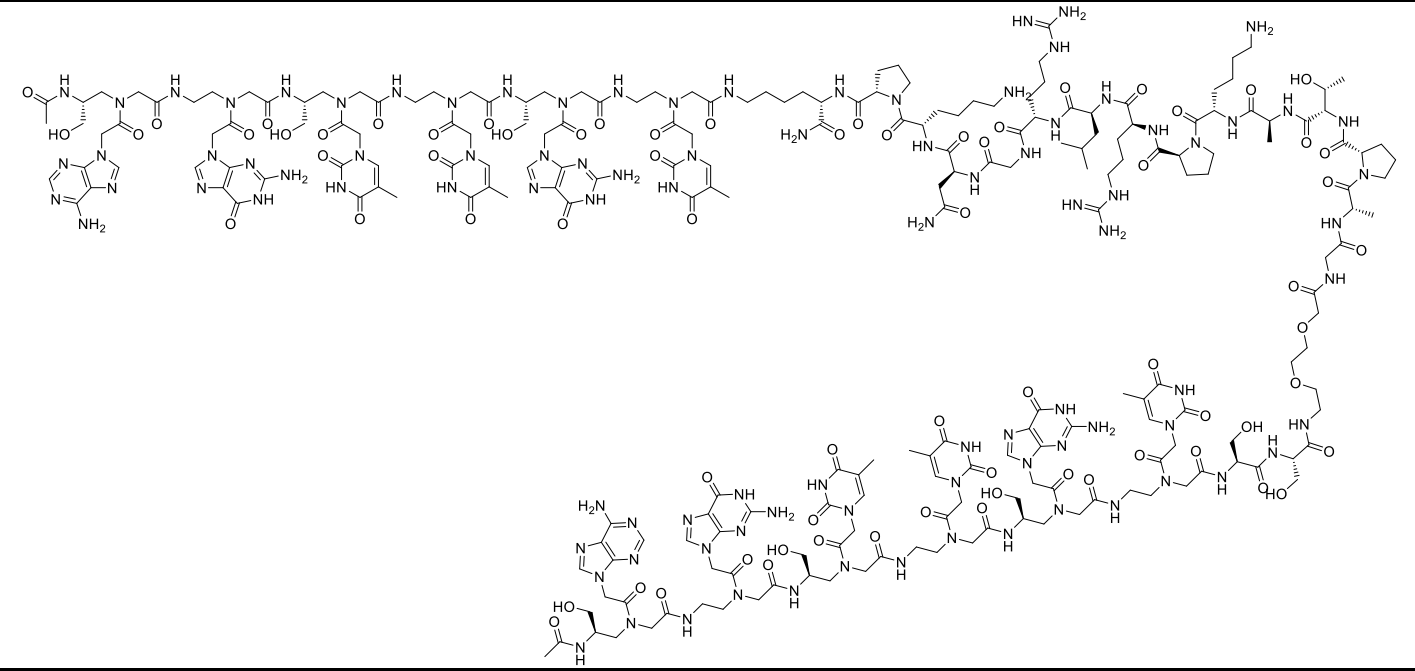	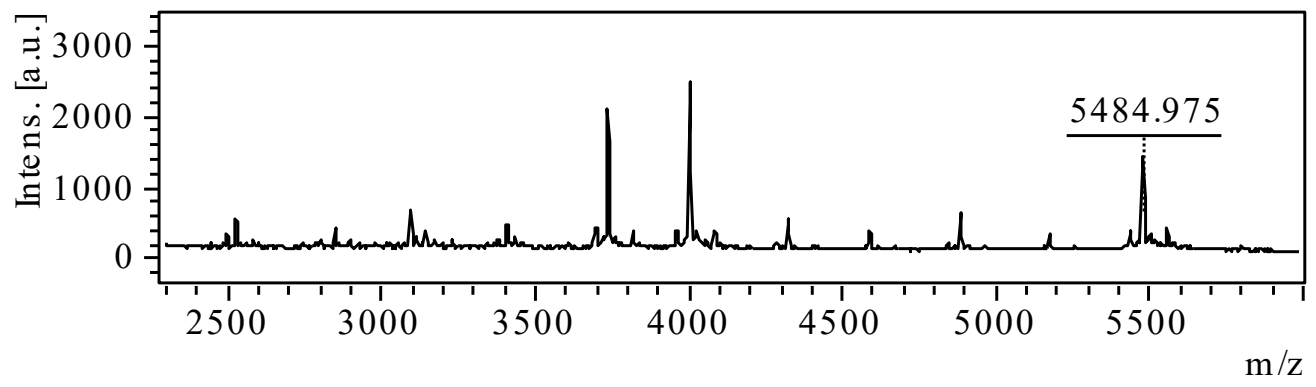
**AS-40**	$C_{210}H_{297}N_{87}O_{70}$	5160.225	5160.225	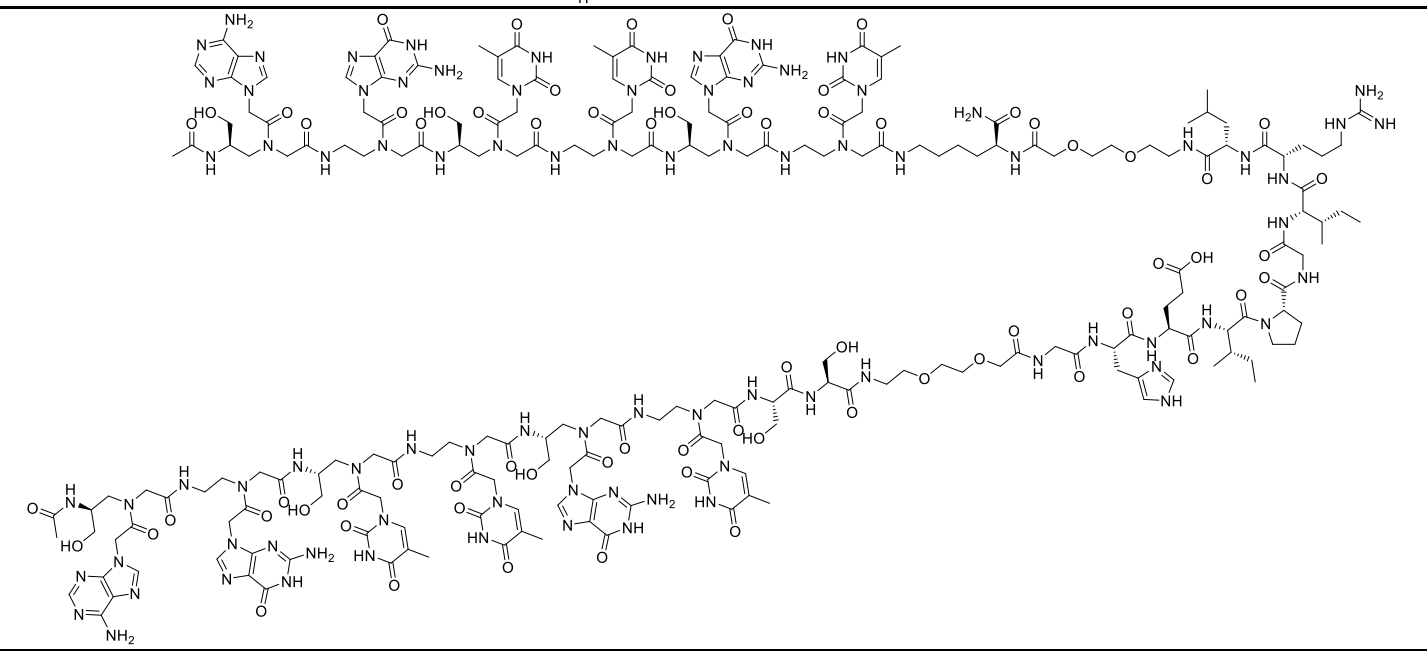	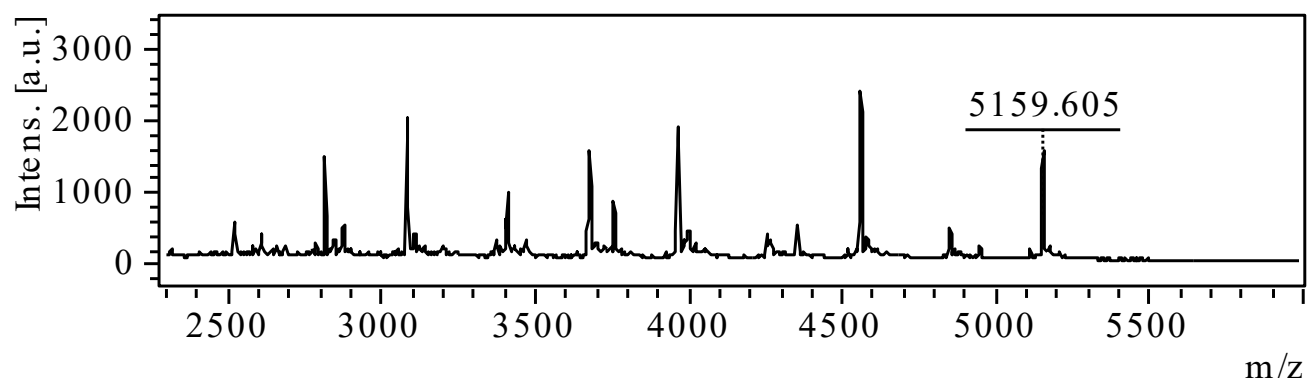
**AS-41**	$C_{214}H_{301}N_{87}O_{72}$	5244.299	5243.263	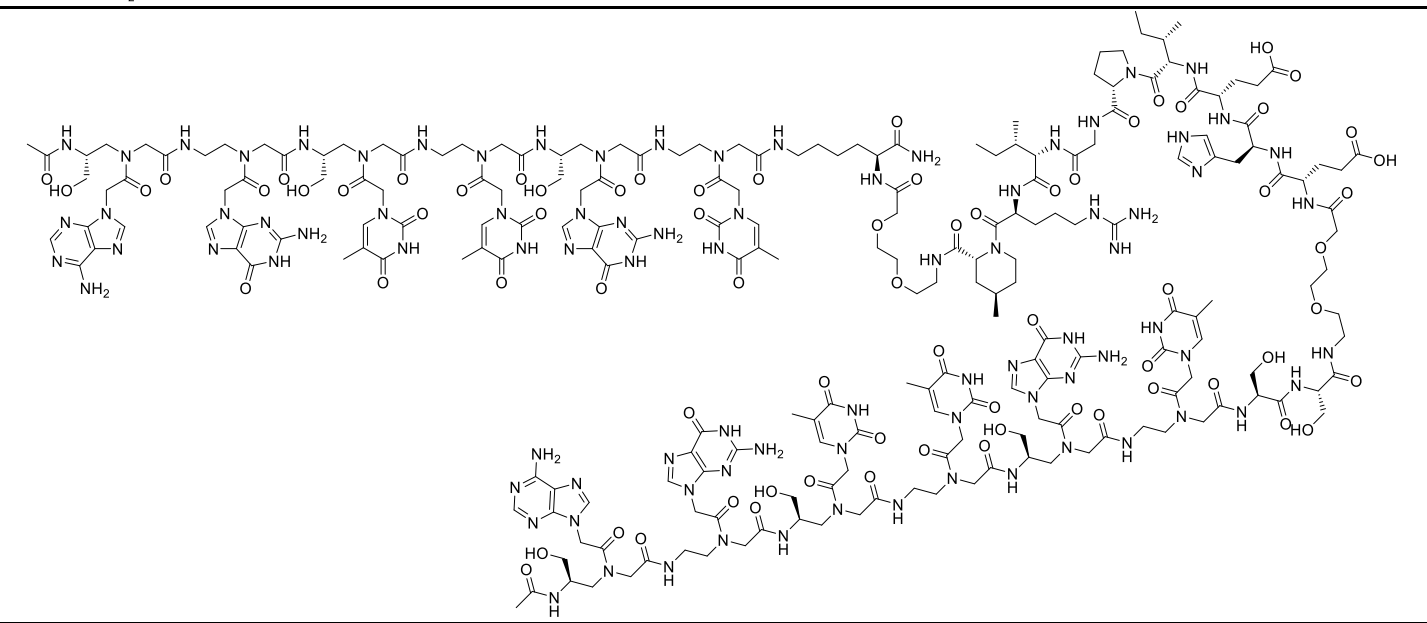	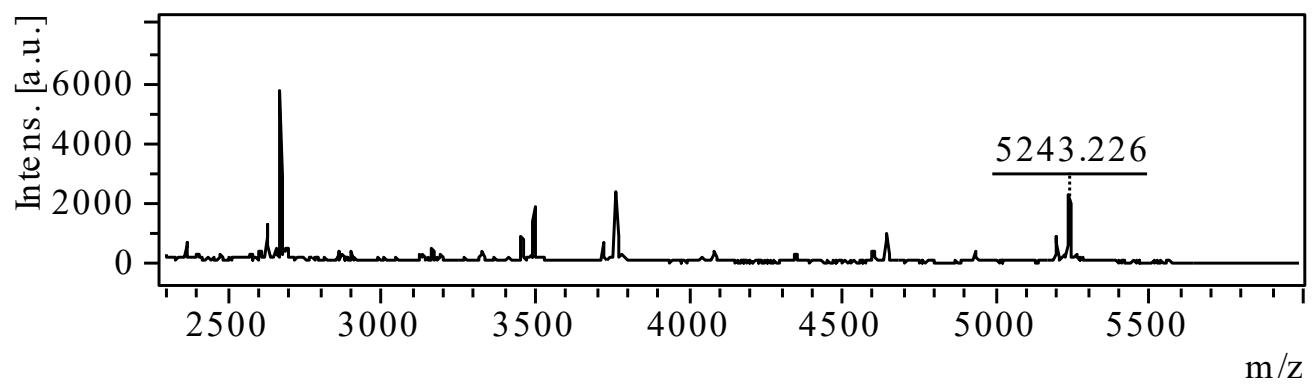

**AS-42**	$C_{211}H_{299}N_{87}O_{70}$	5174.252	5173.258	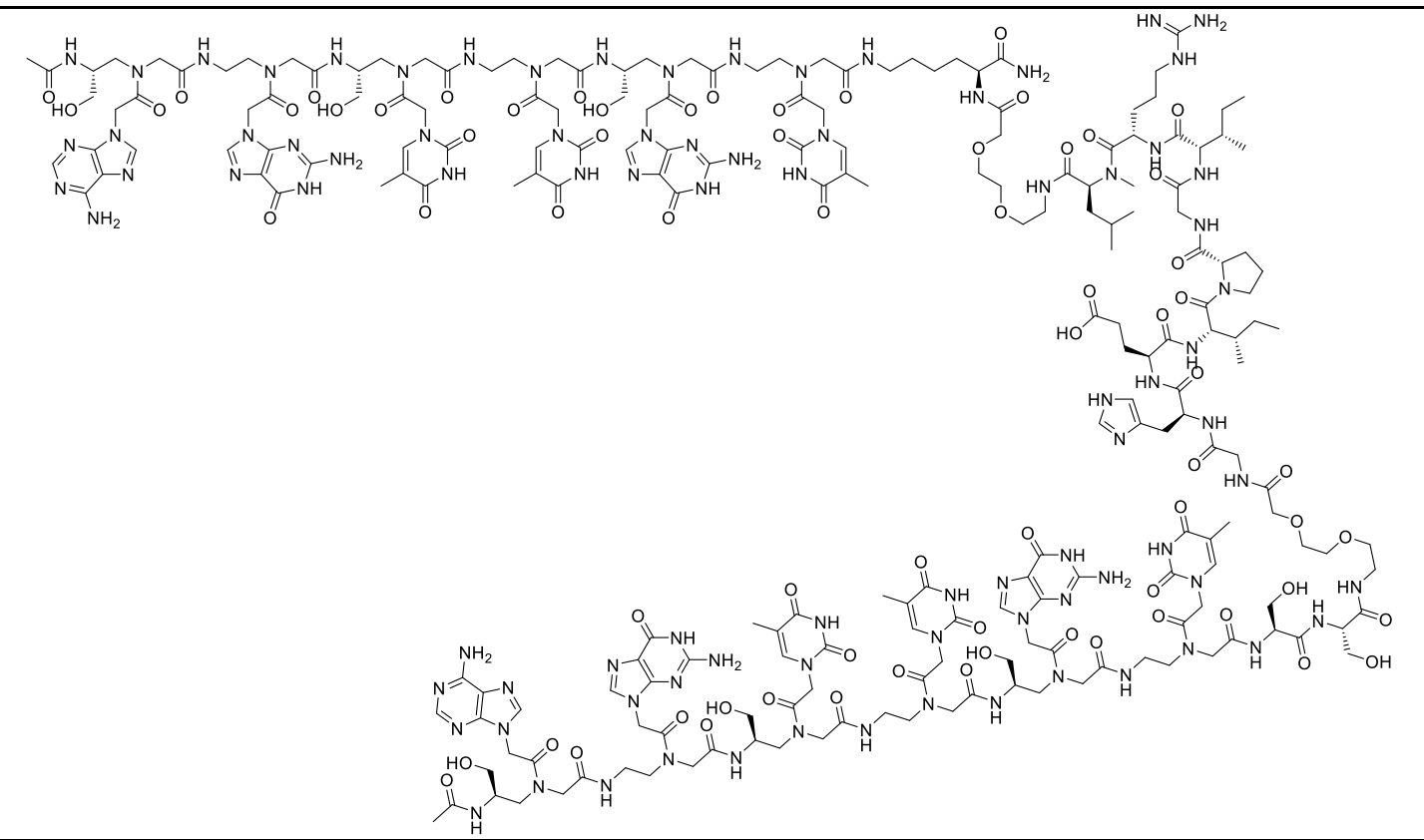	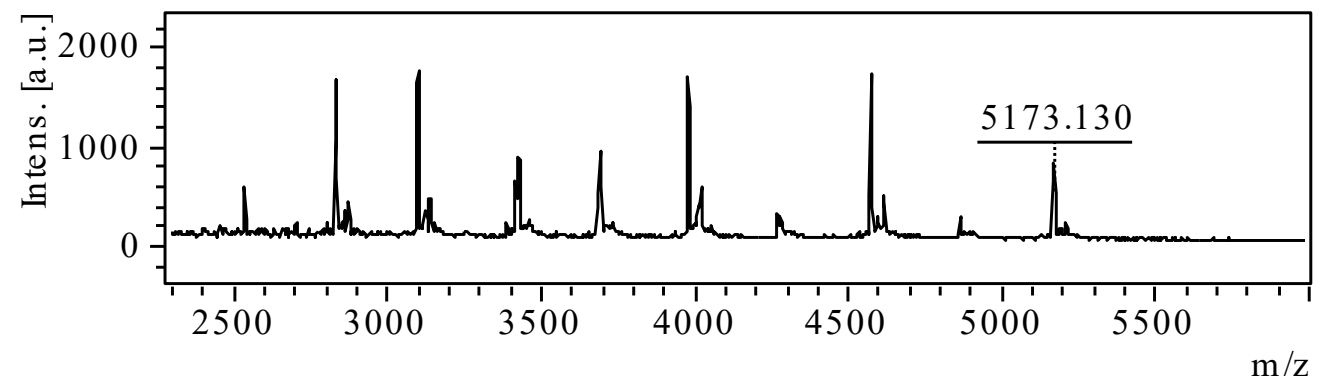
**AS-43**	$C_{220}H_{308}N_{92}O_{70}$	5361.458	5360.344	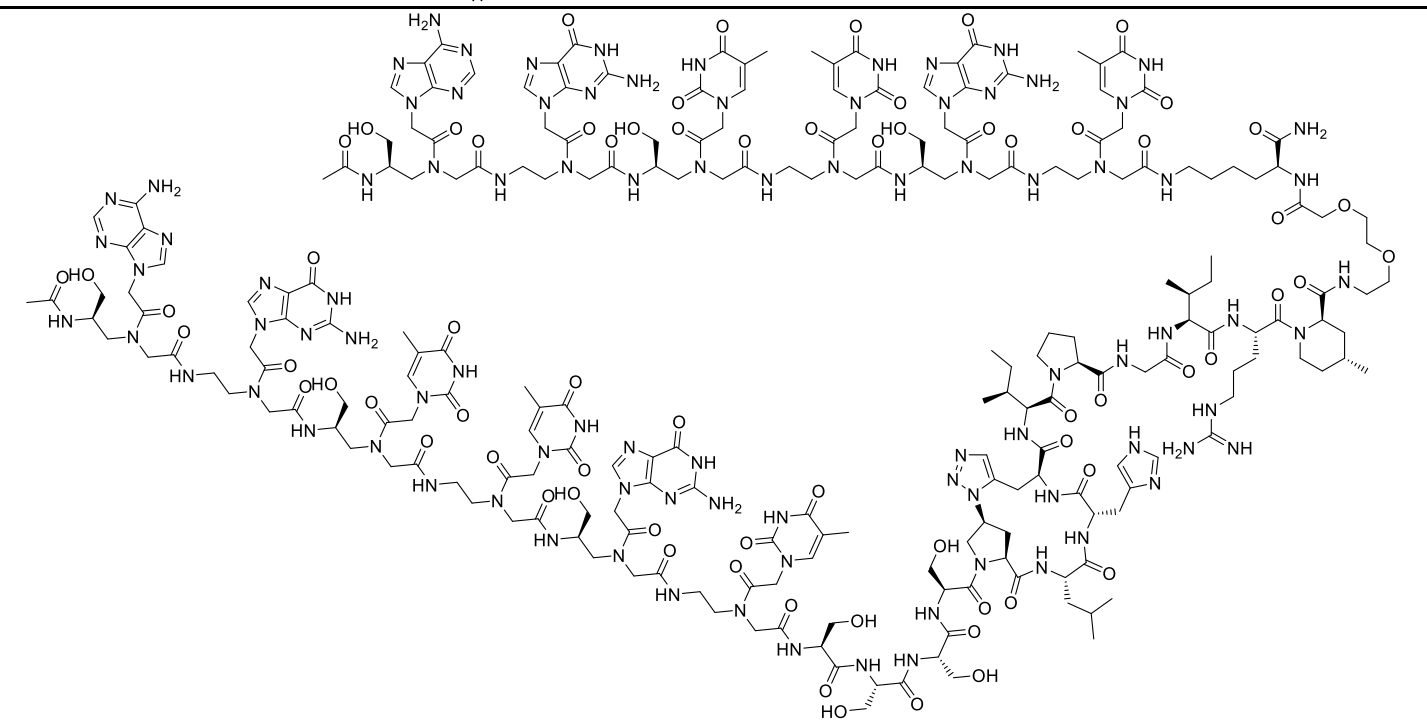	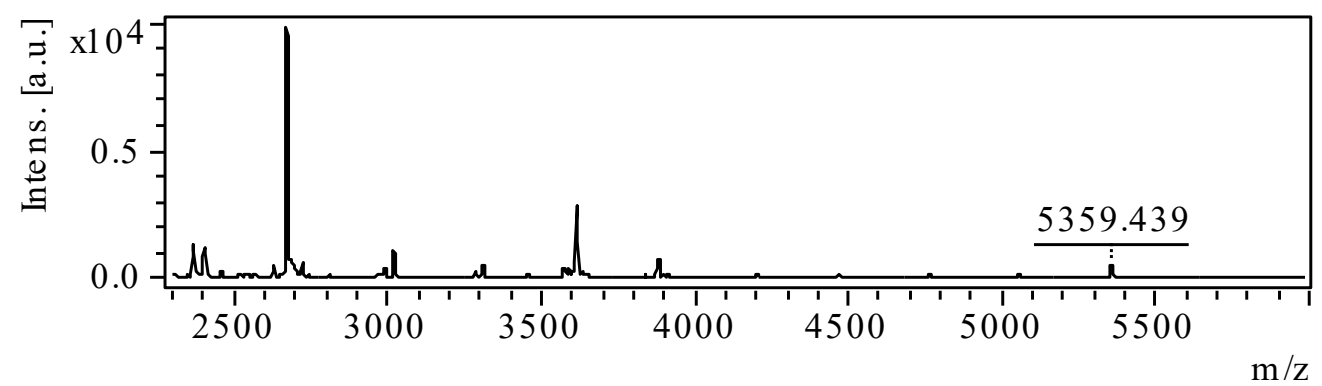
**AS-44**	$C_{203}H_{286}N_{82}O_{67}S$	4979.088	4978.128	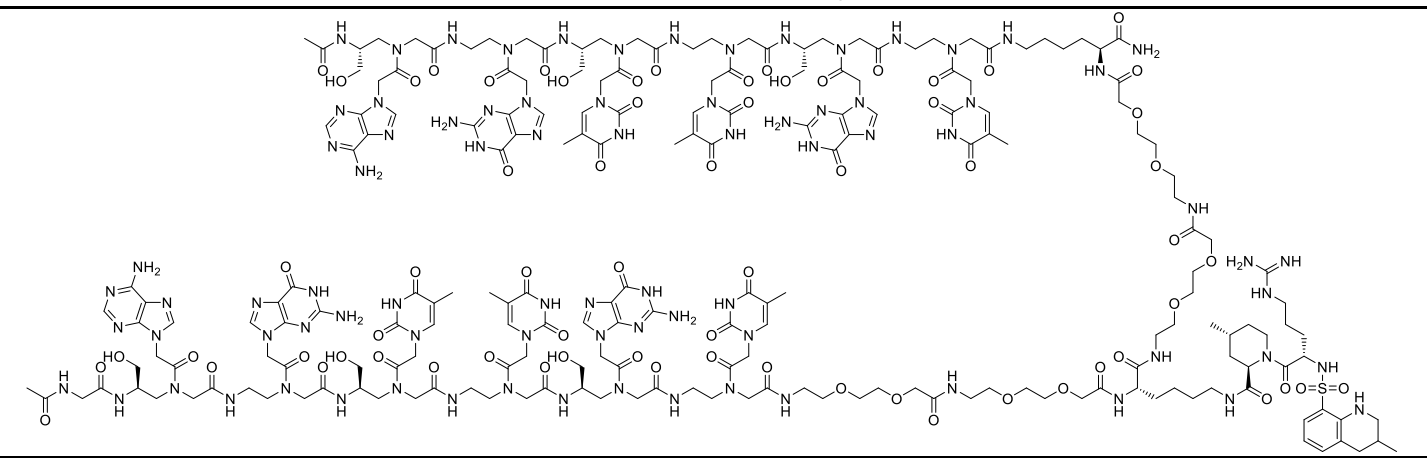	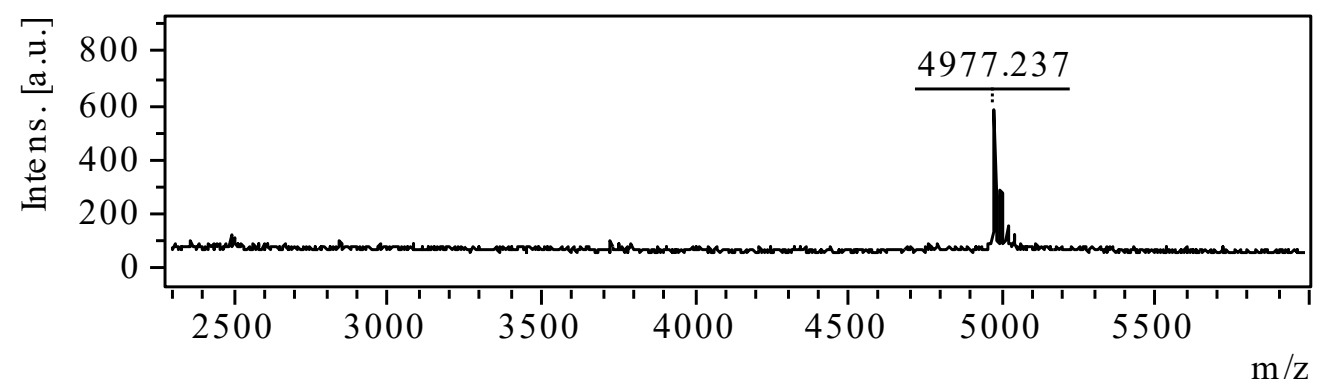

**AS-45**	$C_{198}H_{280}N_{86}O_{68}$	4952.952	4952.116	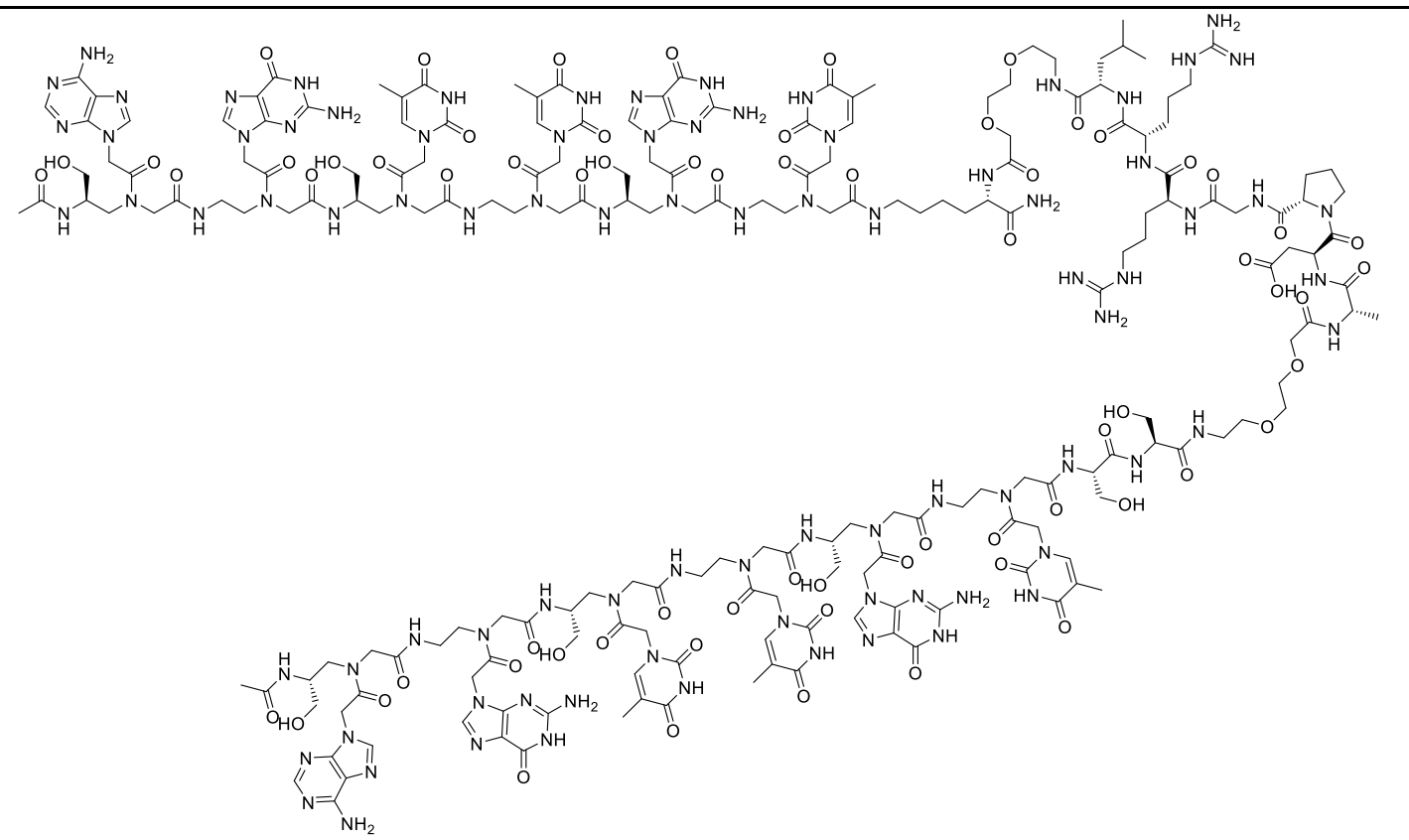	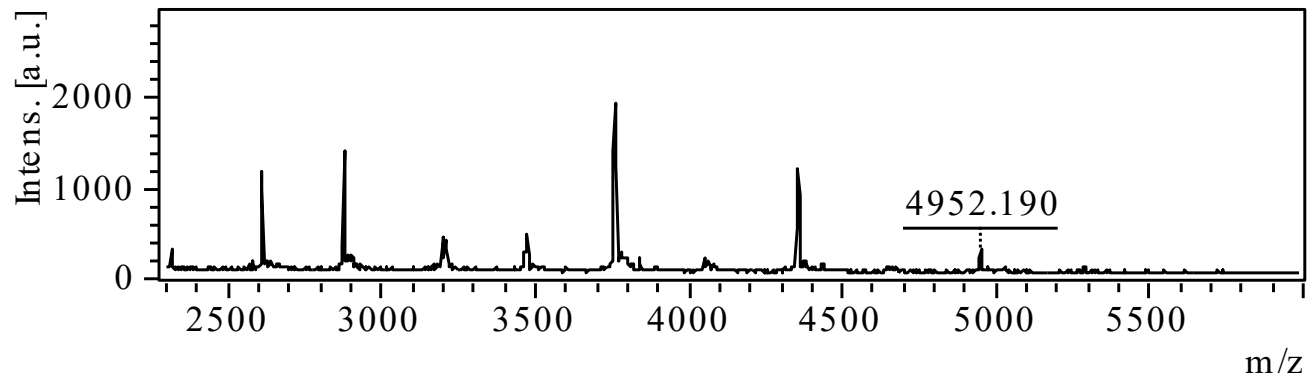
**AS-46**	$C_{199}H_{280}N_{86}O_{68}$	4964.963	4964.116	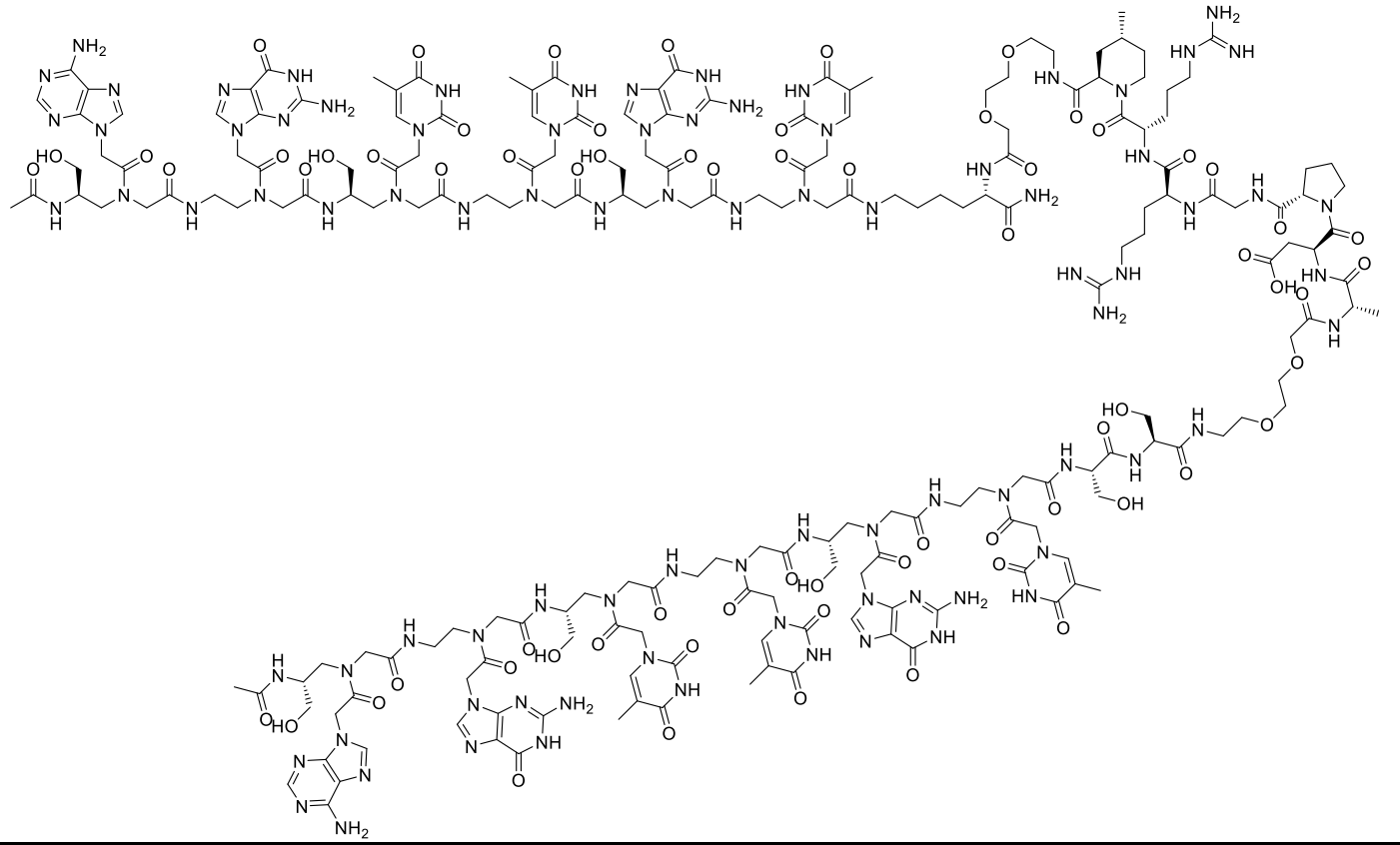	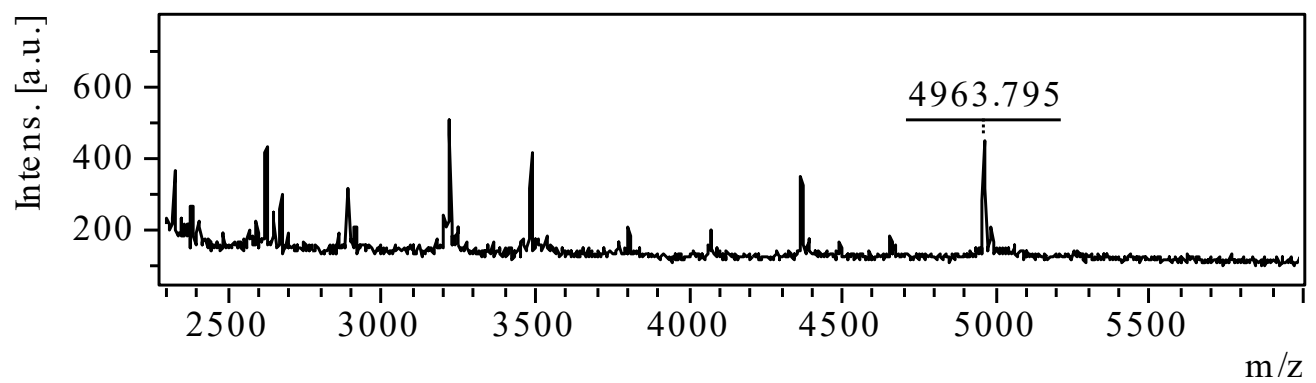

**AS-47**	$C_{203}H_{291}N_{87}O_{70}$	5070.1	5069.195	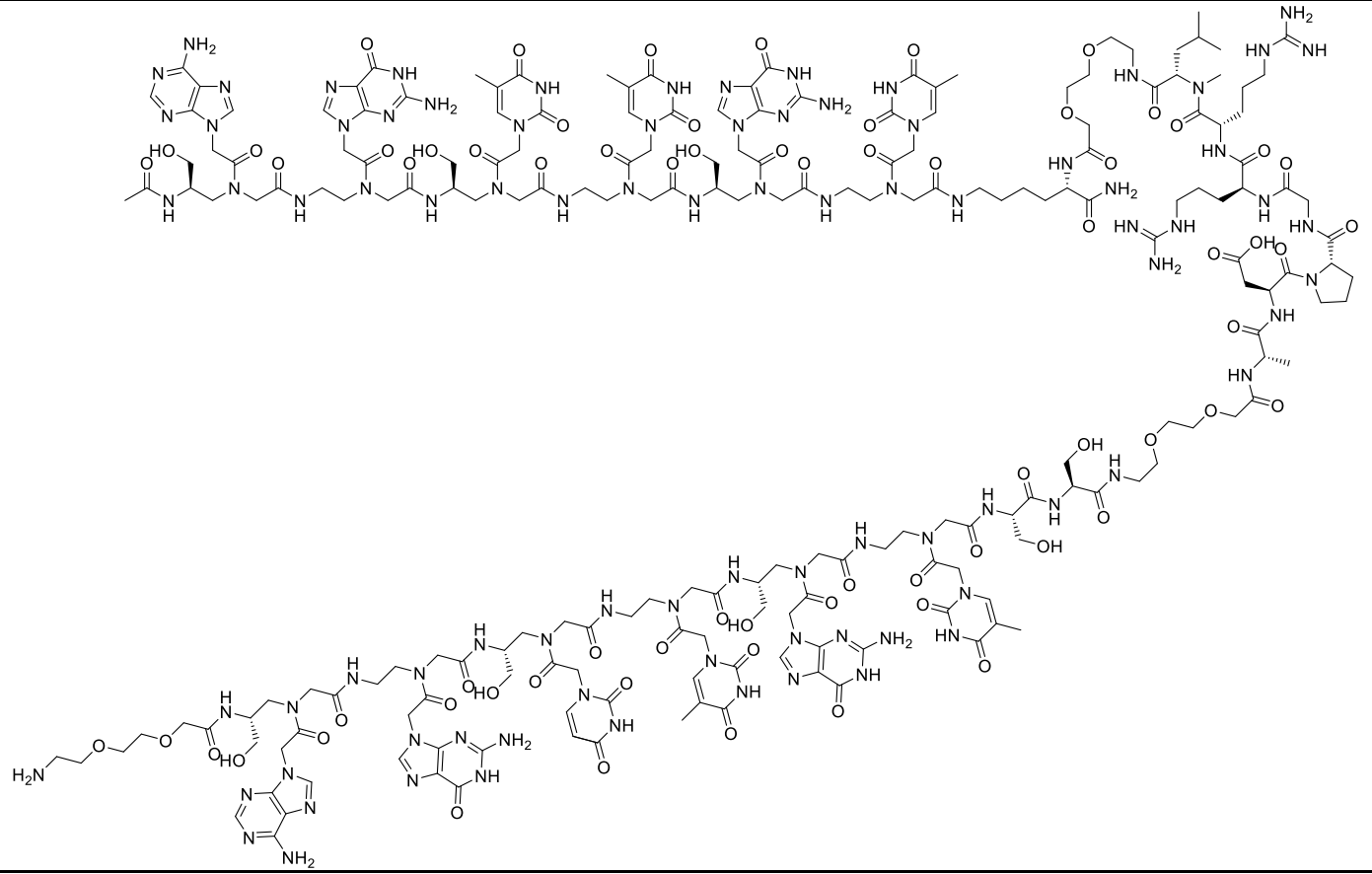	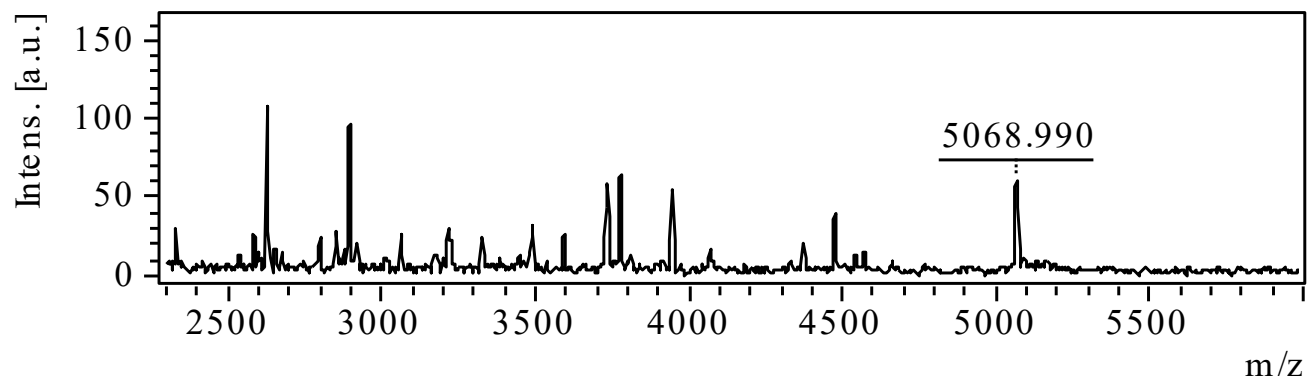
**AS-48**	$C_{202}H_{270}N_{92}O_{62}S$	5011.024	5010.059	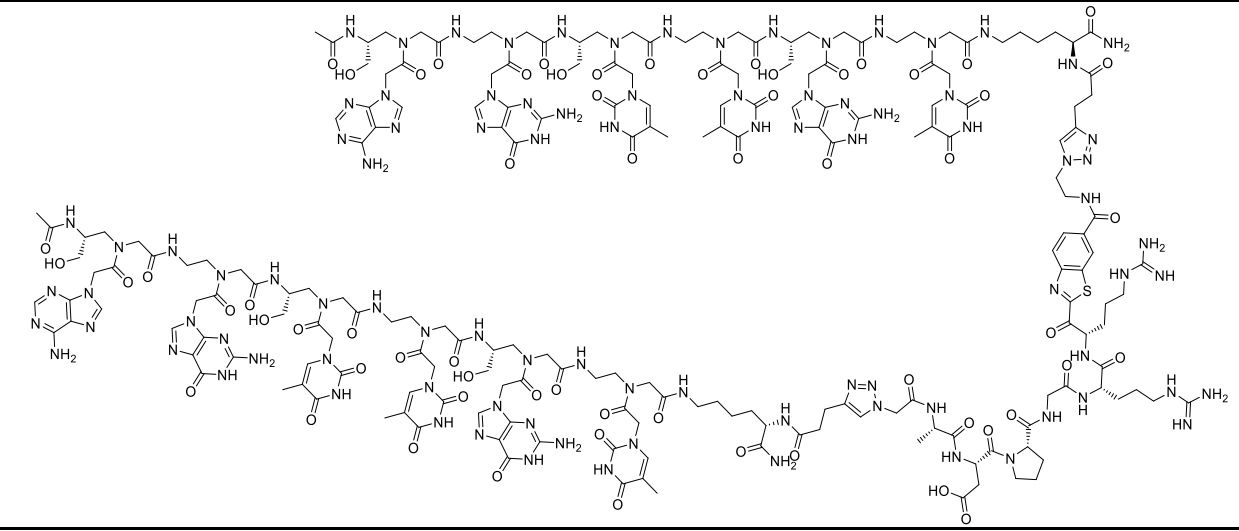	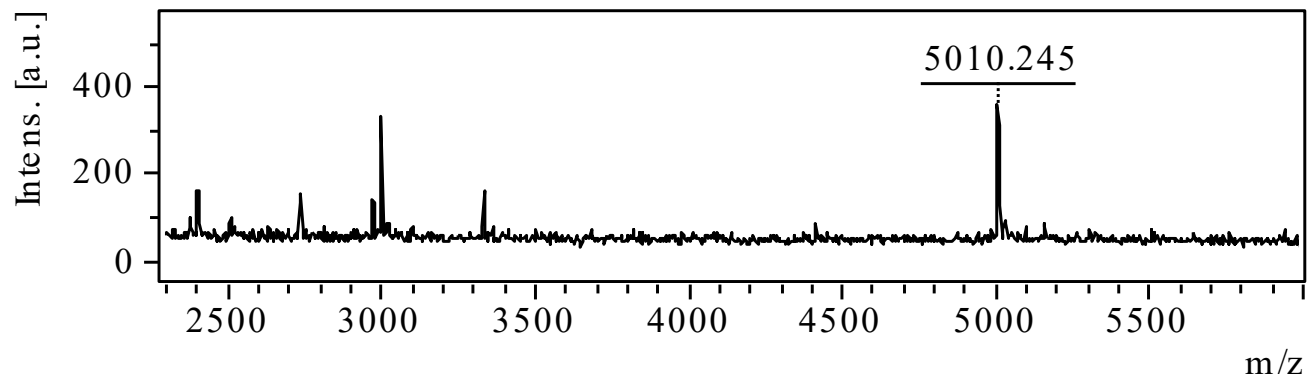
**AS-49**	$C_{202}H_{279}F_2N_{81}O_{69}$	4983.949	4983.085	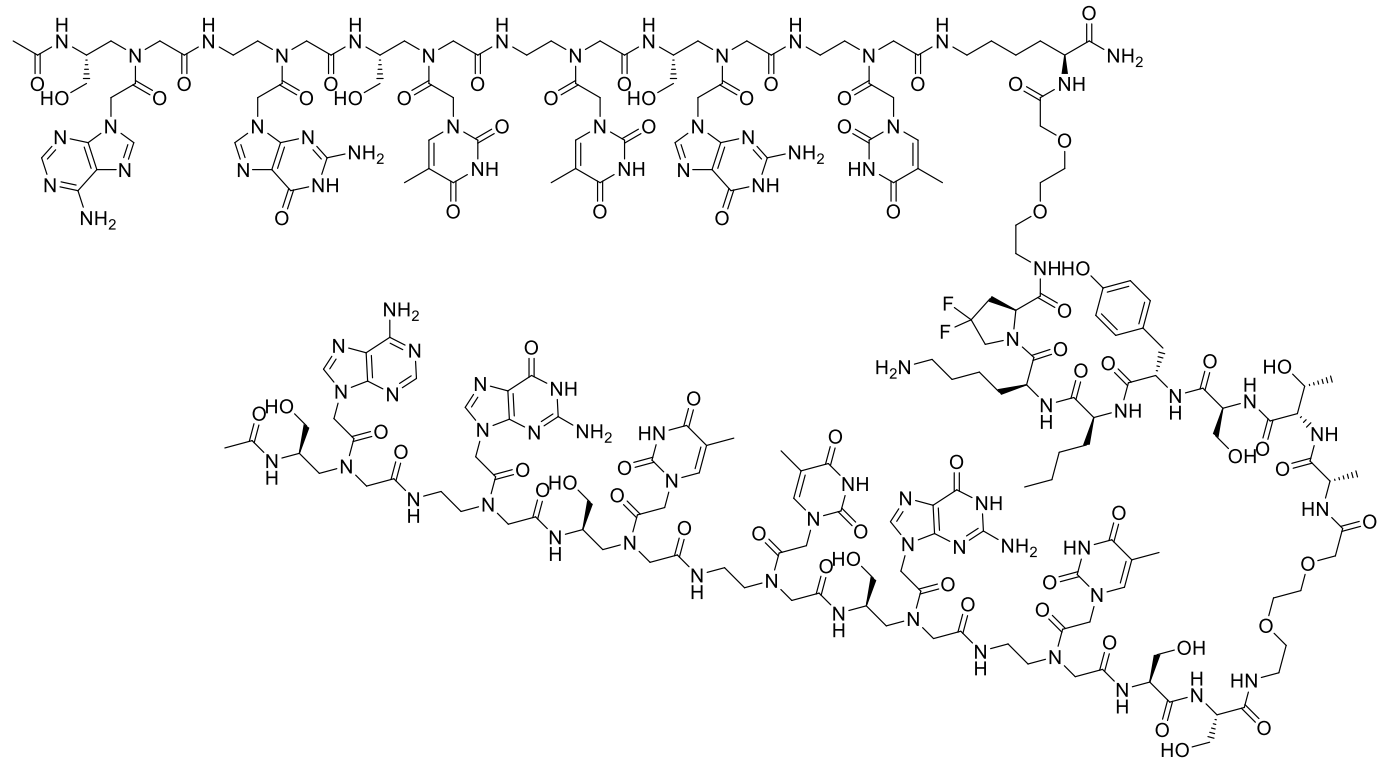	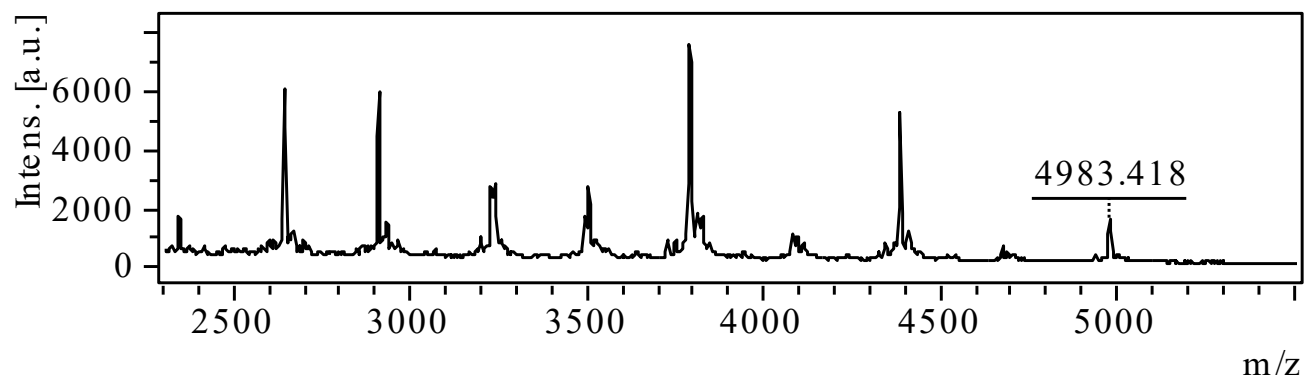

**AS-50**	$C_{200}H_{279}N_{81}O_{69}$	4921.93	4921.088	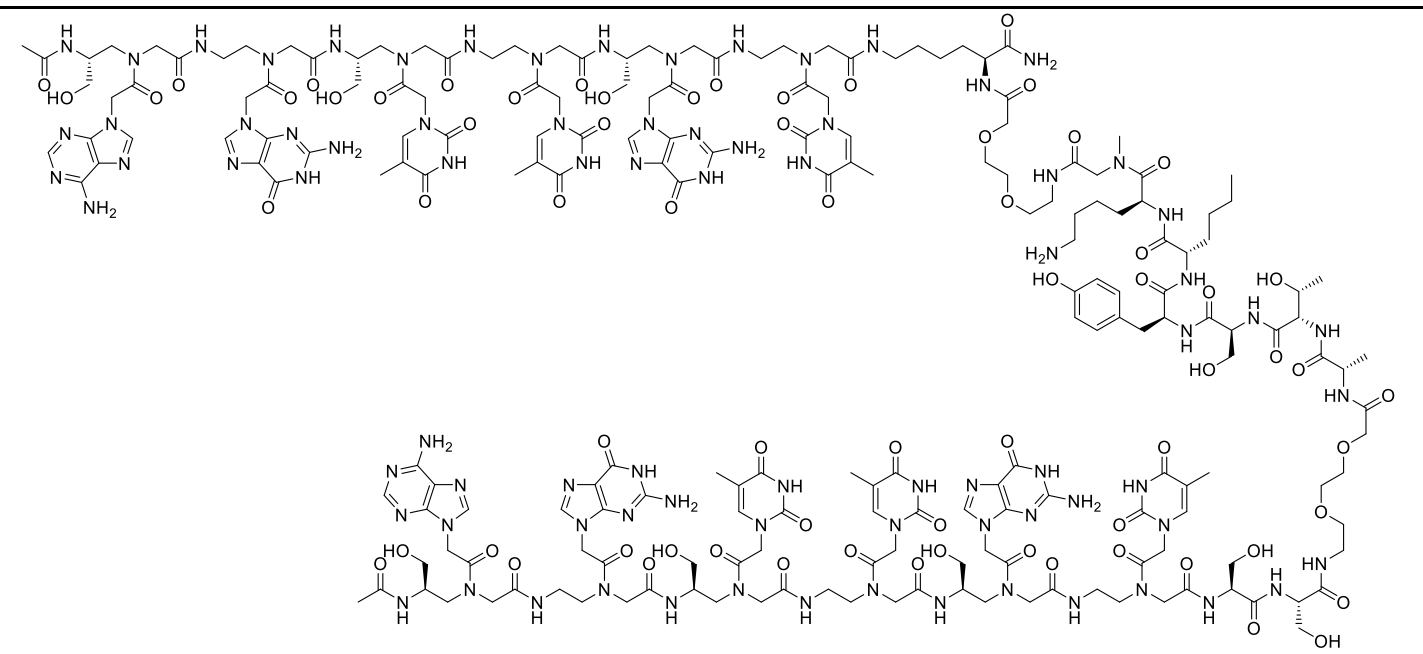	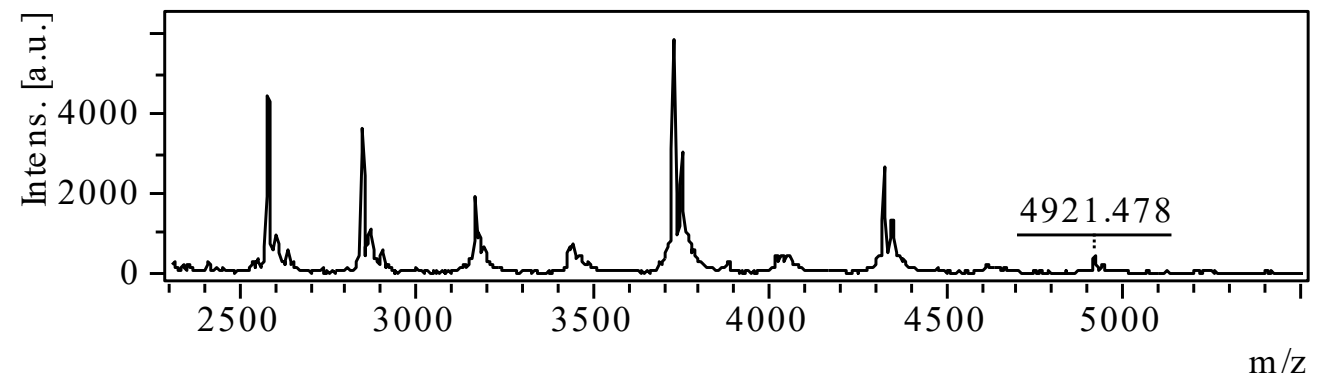

13. Final compound characterisation

Ex1-16

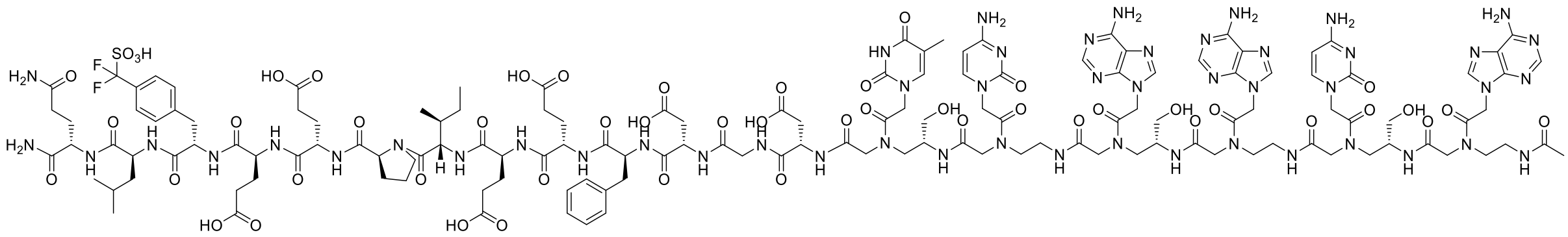

Chemical Formula: $C_{140}H_{186}F_2N_{50}O_{49}S$, **Exact Mass**: 3421.33, **Molecular Weight**: 3423.39.

MALDI-TOF; m/z found: 3424.454.

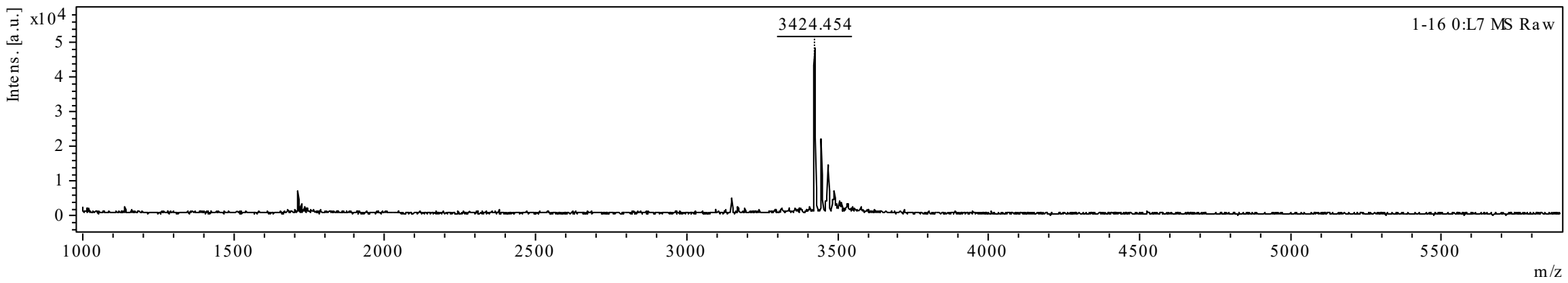

LCMS (ESI); RT= 1.60 min, $[M+2H]^{2+}$: 1712.42, $[M+3H]^{3+}$: 1142.00, $[M+4H]^{4+}$: 856.83.

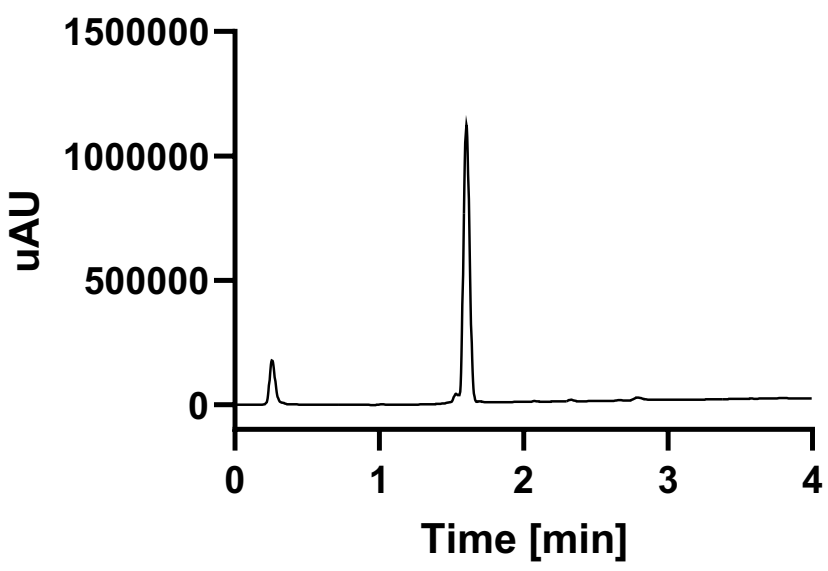

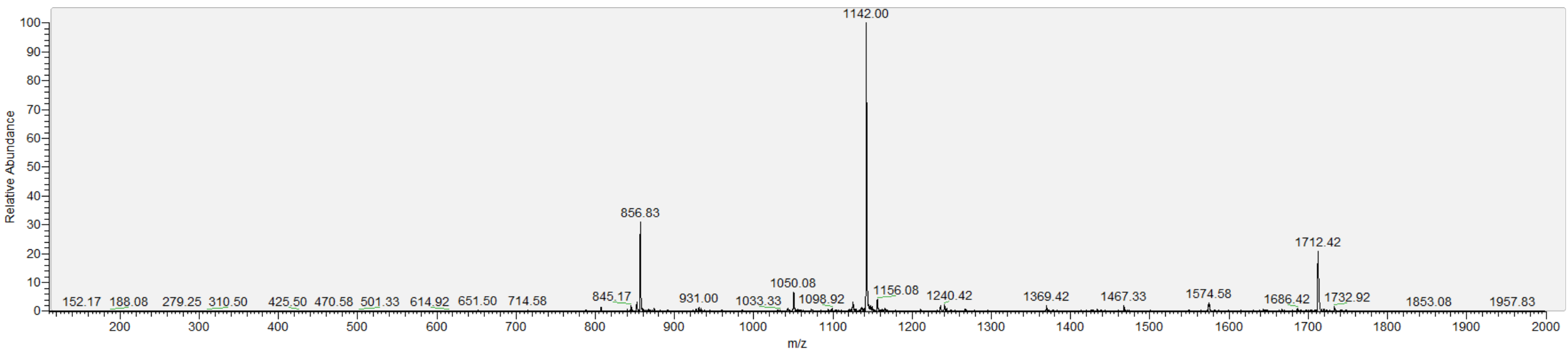

Ex1-16-TH

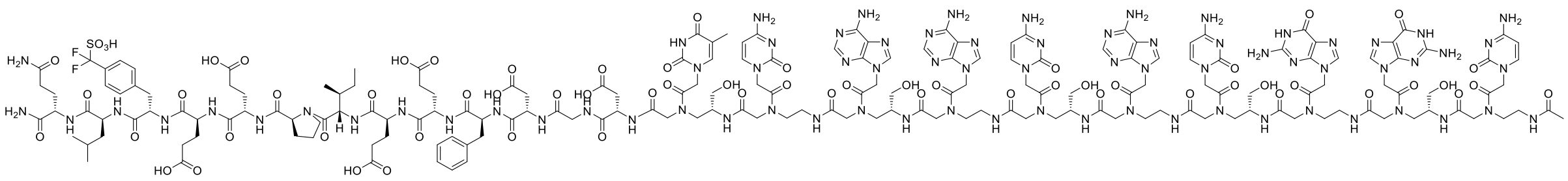

Chemical Formula: $C_{184}H_{242}F_2N_{74}O_{63}S$, **Exact Mass**: 4565.77, **Molecular Weight**: 4568.47.

MALDI-TOF; m/z found: 4569.208.

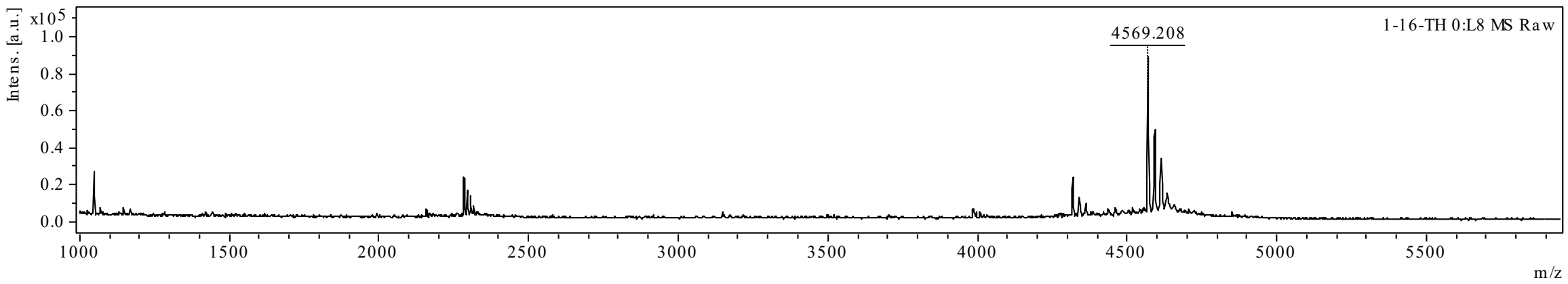

LCMS (ESI); RT= 1.58 min, $[M+3H]^{3+}$: 1523.50, $[M+4H]^{4+}$: 1143.08, $[M+5H]^{5+}$: 914.83.

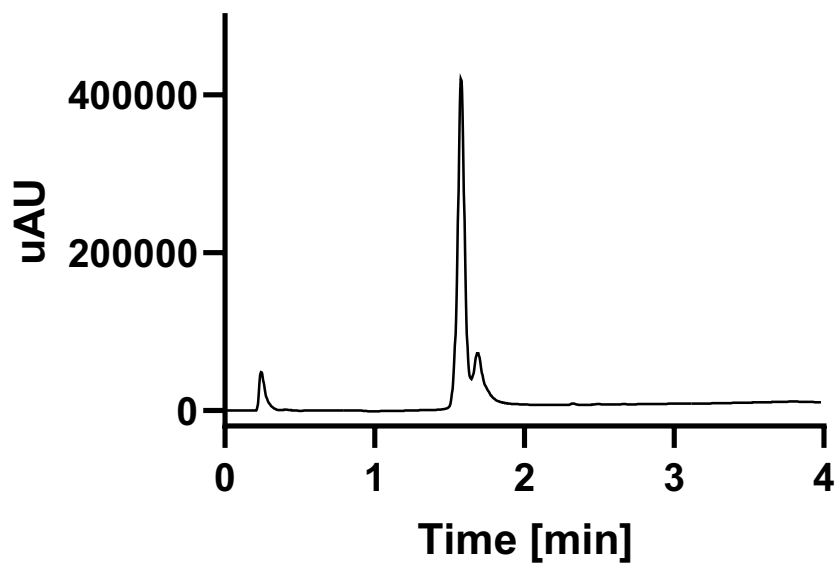

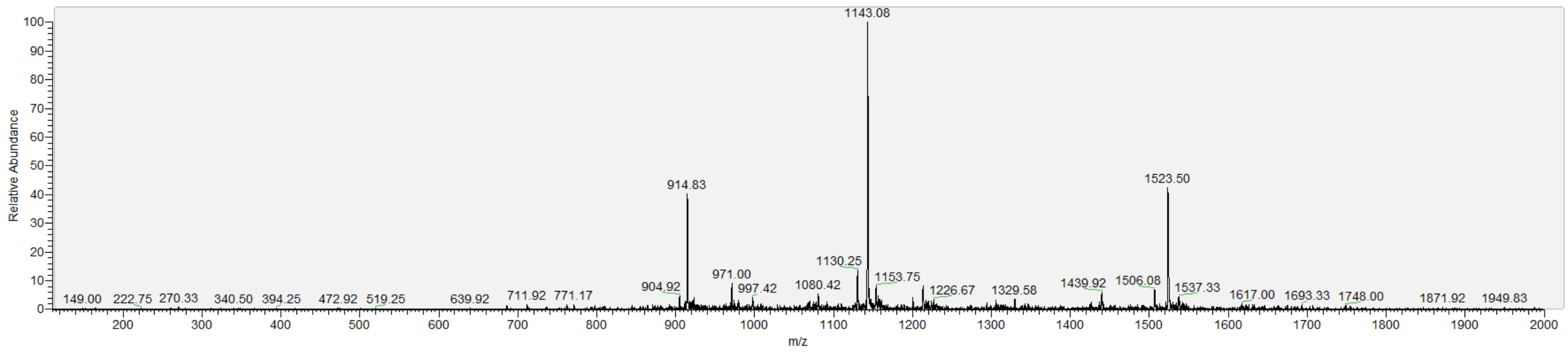

Ex2-1

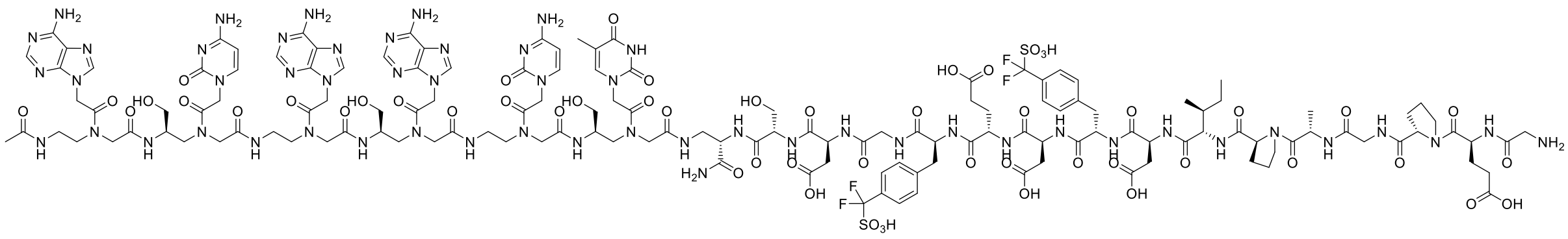

Chemical Formula: $C_{142}H_{187}F_4N_{53}O_{53}S_2$, **Exact Mass**: 3622.29, **Molecular Weight**: 3624.49.

MALDI-TOF; m/z found: 3625.478.

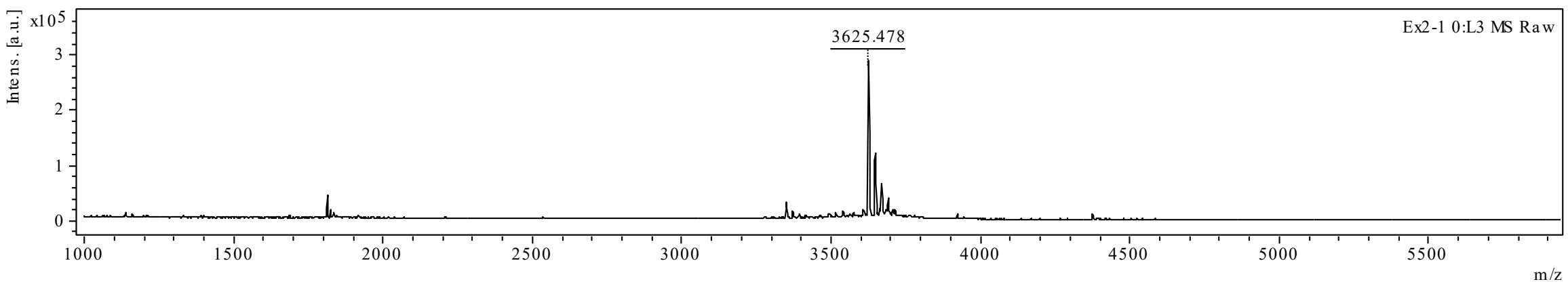

LCMS (ESI); RT= 1.40 min, $[M+3H]^{3+}$: 1208.92, $[M+4H]^{4+}$: 906.92.

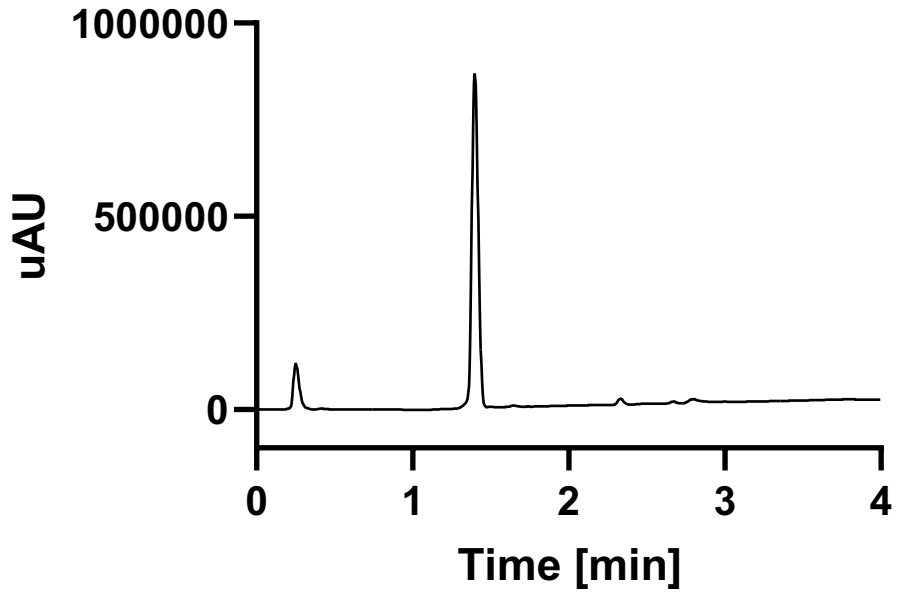

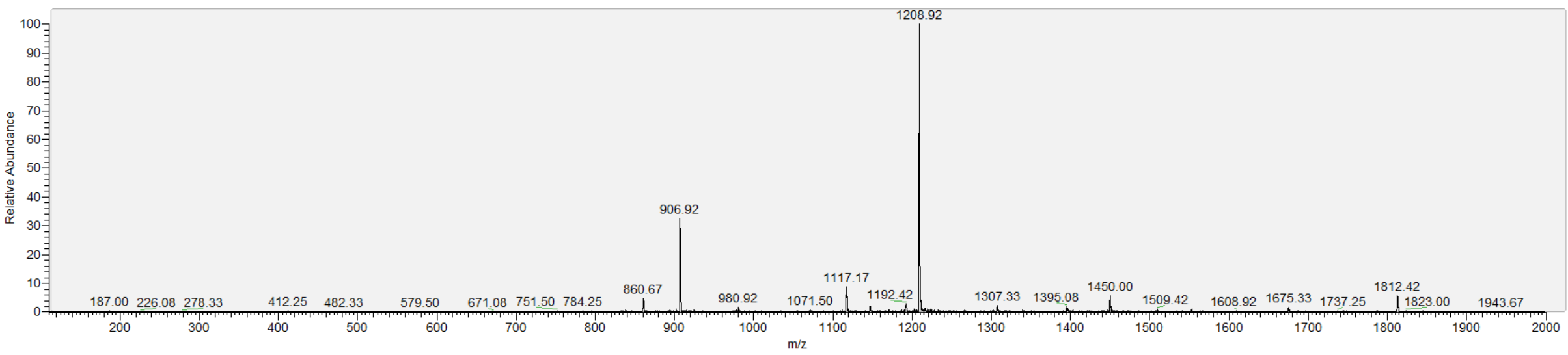

<u>**Ex2-1-TH**</u>

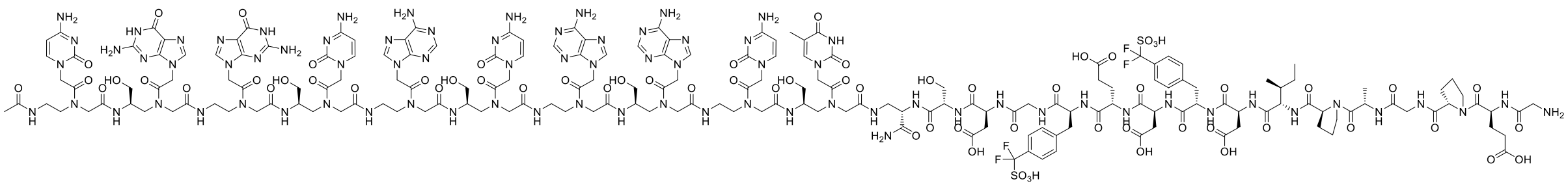

Chemical Formula: $C_{186}H_{243}F_4N_{77}O_{67}S_2$, **Exact Mass**: 4766.74, **Molecular Weight**: 4769.58.

MALDI-TOF; m/z found: 4770.435.

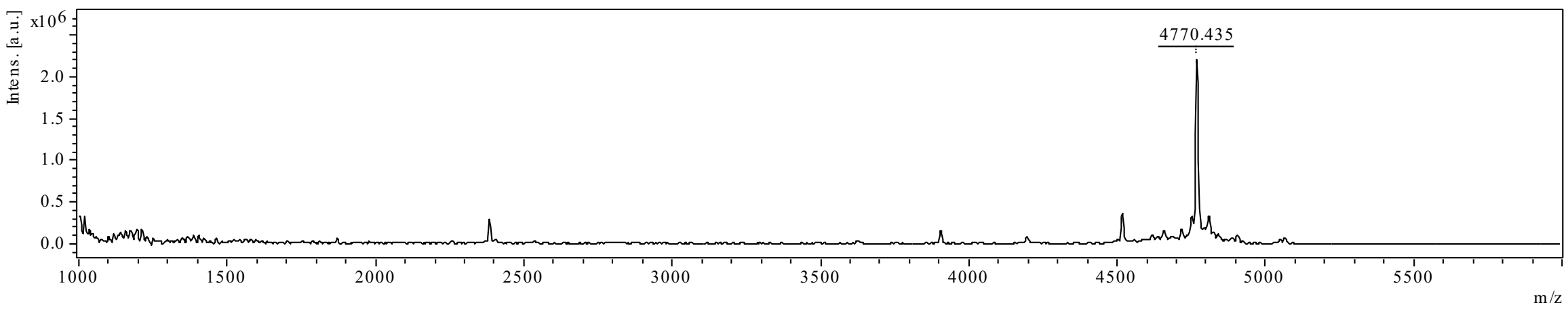

LCMS (ESI); RT= 1.36 min, $[M+3H]^{3+}$: 1590.75, $[M+4H]^{4+}$: 1193.42, $[M+5H]^{5+}$: 954.92.

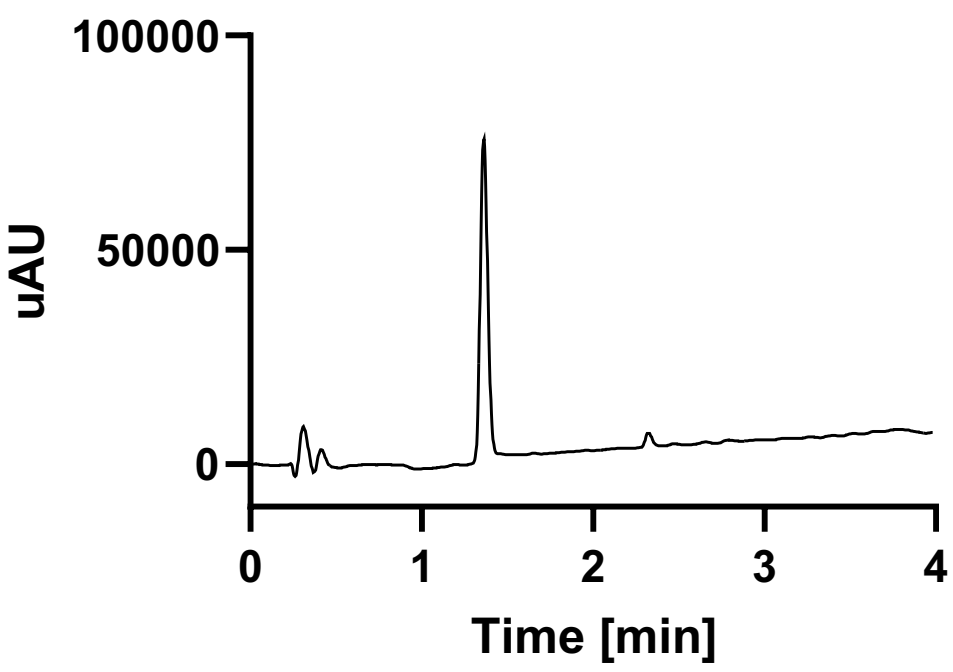

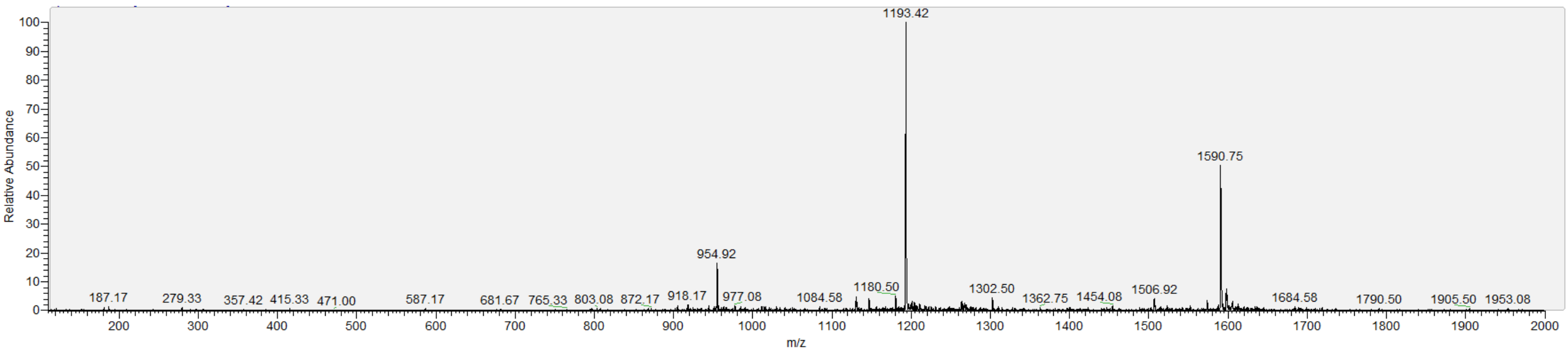

AS31

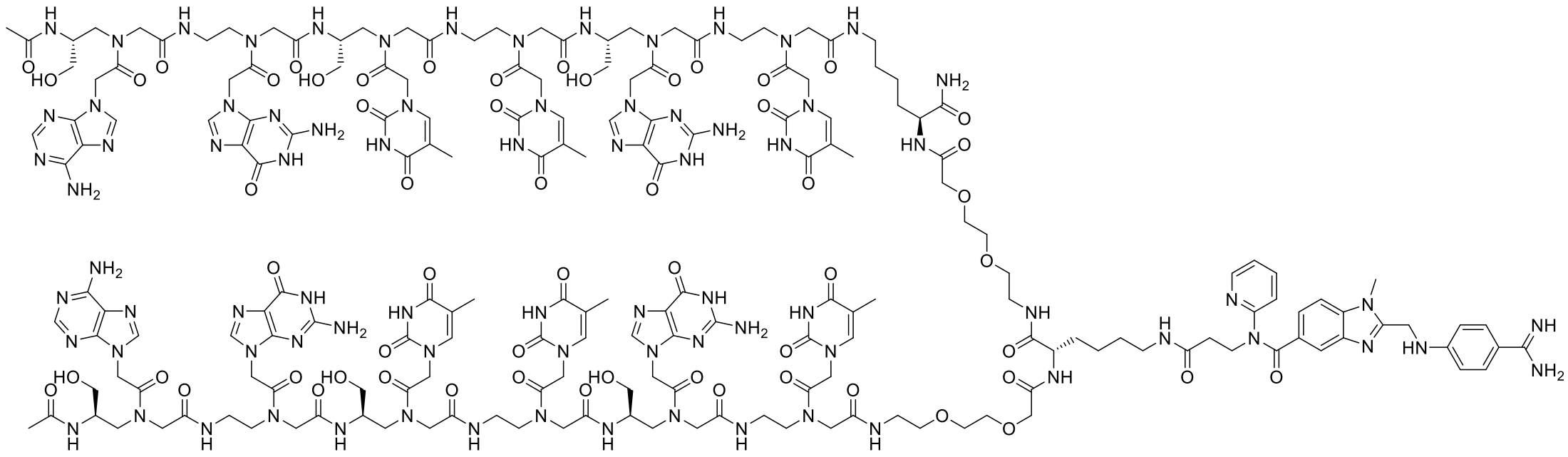

Chemical Formula: $C_{191}H_{250}N_{80}O_{58}$, **Exact Mass**: 4591.91, **Molecular Weight**: 4594.60.

MALDI-TOF; m/z found: 4593.295.

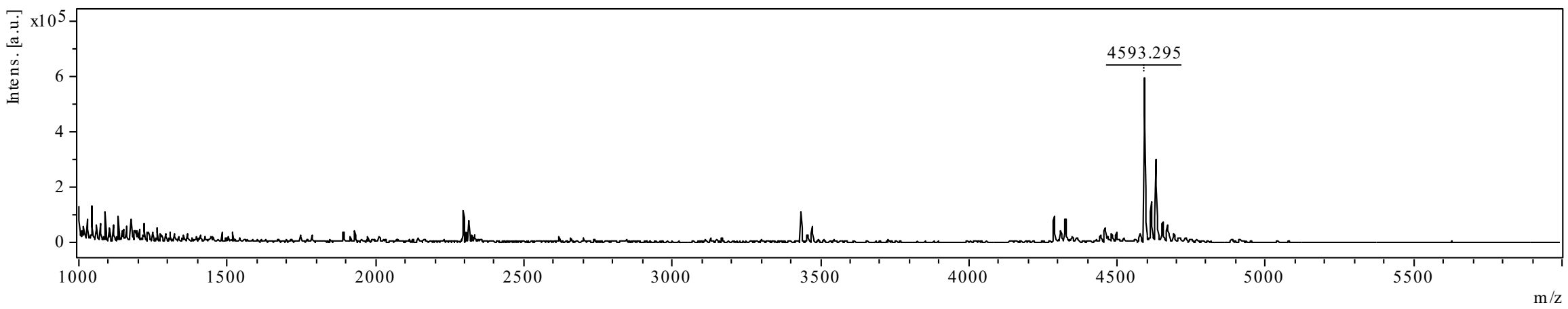

LCMS (ESI); RT= 1.32 min, $[M+3H]^{3+}$: 1532.17, $[M+4H]^{4+}$: 1149.58, $[M+5H]^{5+}$: 919.92, $[M+6H]^{6+}$: 767.00.

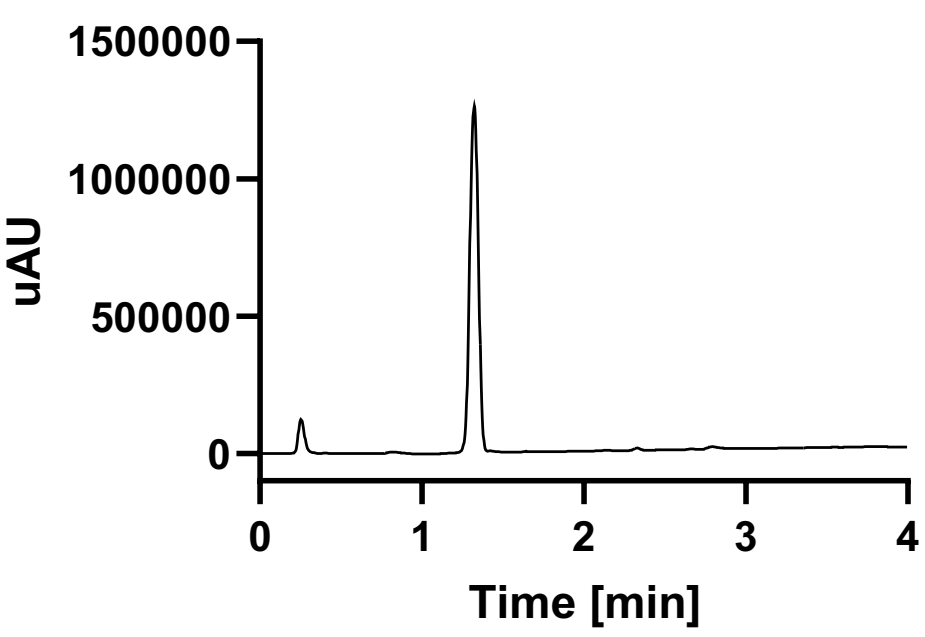

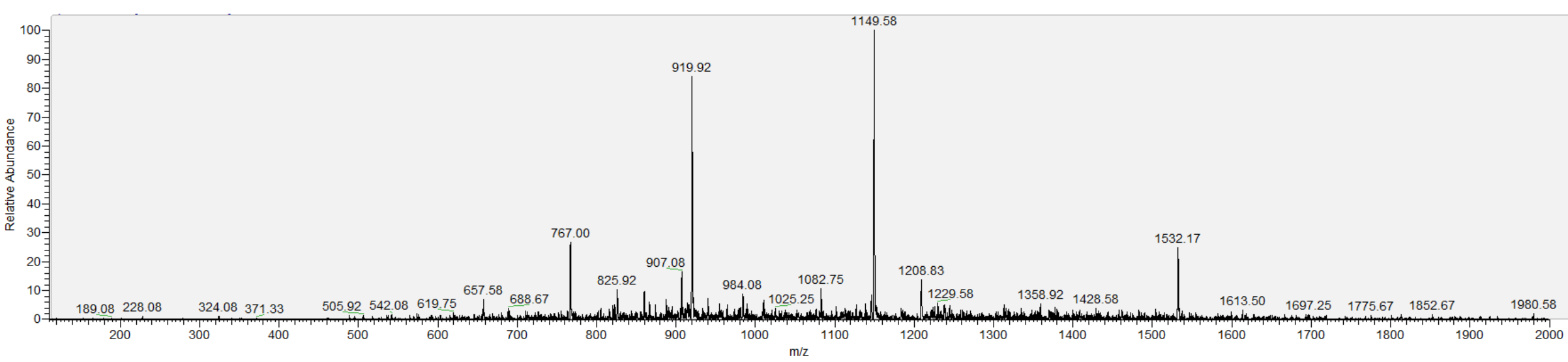

Antidote (AD)

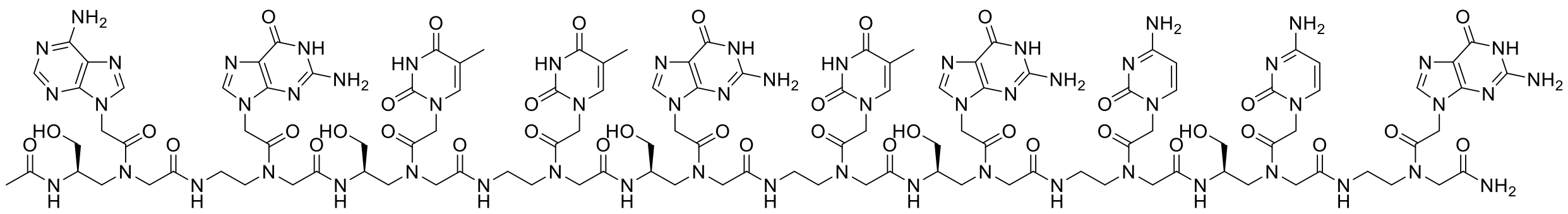

Chemical Formula: $C_{115}H_{148}N_{58}O_{38}$, **Exact Mass**: 2949.14, **Molecular Weight**: 2950.82.

MALDI-TOF; m/z found: 2949.158.

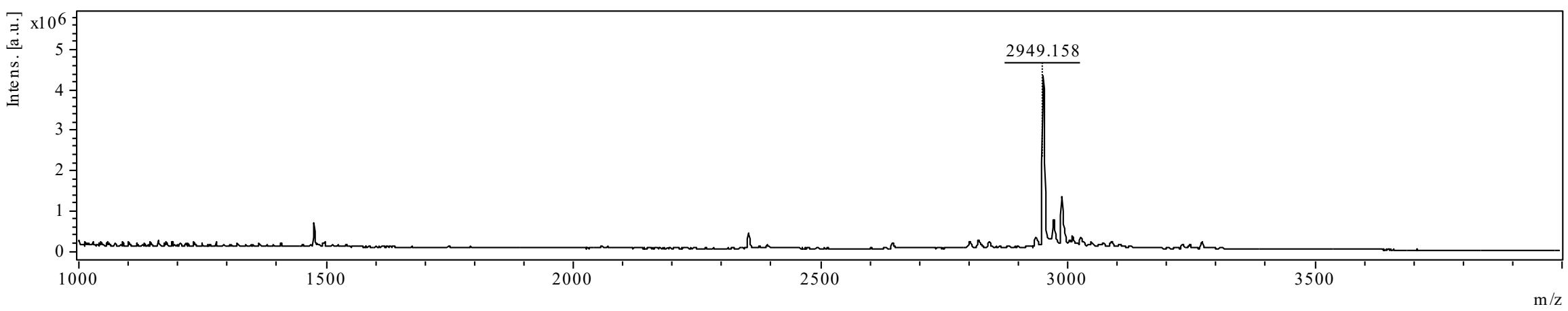

LCMS (ESI); RT= 1.10 min, $[M+2H]^{2+}$: 1476.08, $[M+3H]^{3+}$: 984.58, $[M+4H]^{4+}$: 738.75.

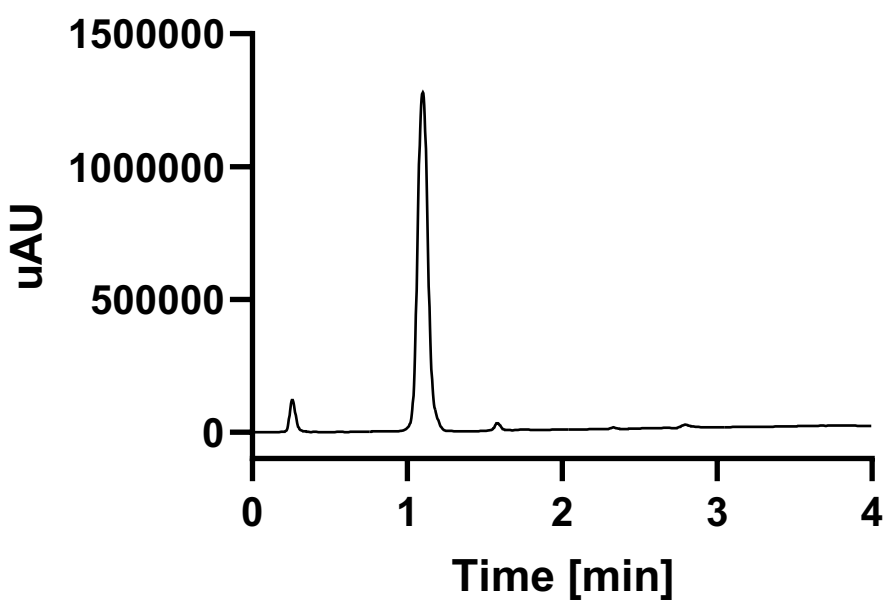

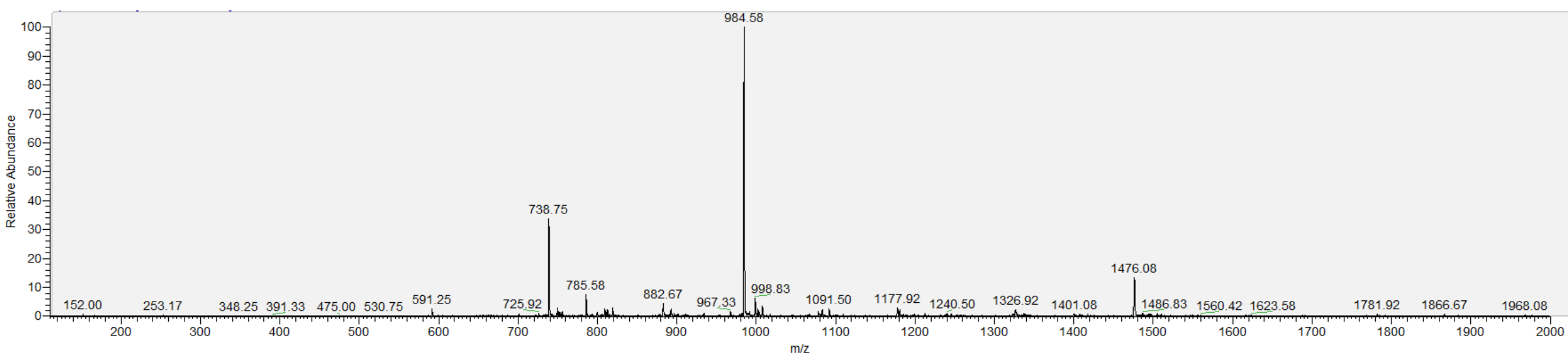

Ex1-16-Biotin (For SPR)

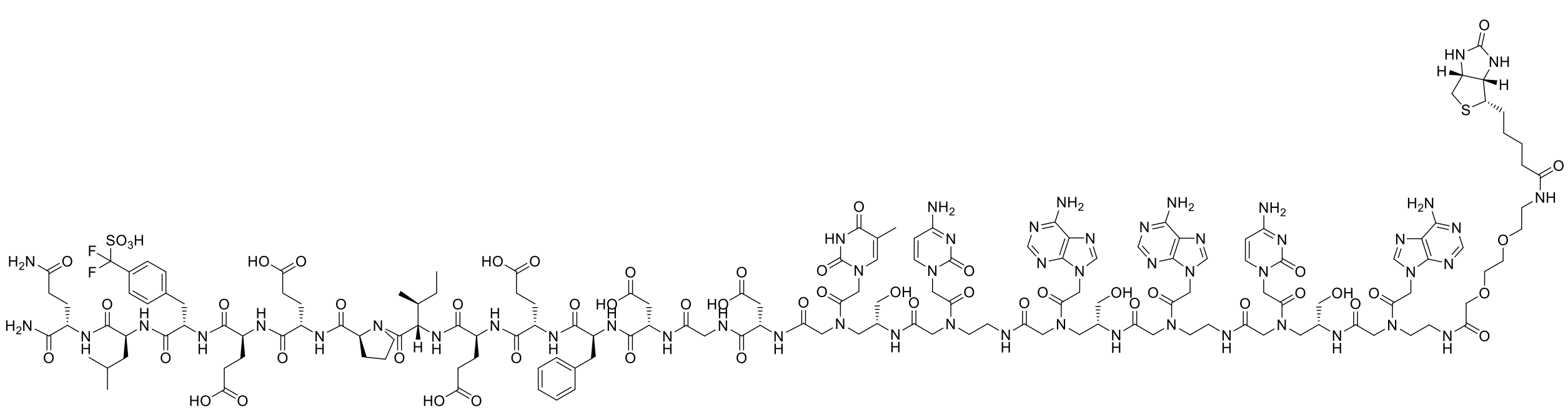

Chemical Formula: $C_{154}H_{209}F_2N_{53}O_{53}S_2$, **Exact Mass**: 3750.47, **Molecular Weight**: 3752.80.

MALDI-TOF; m/z found: 3751.71.

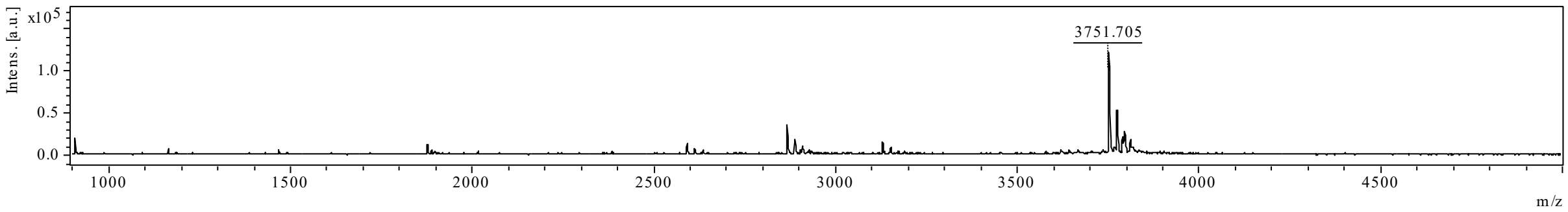

LCMS (ESI); RT= 1.64 min, $[M+2H]^{2+}$: 1876.58, $[M+3H]^{3+}$: 1251.75, $[M+4H]^{4+}$: 939.08.

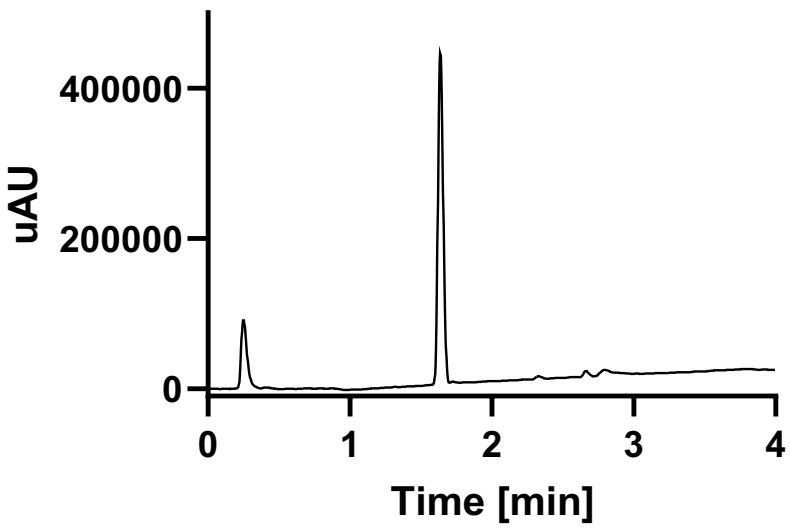

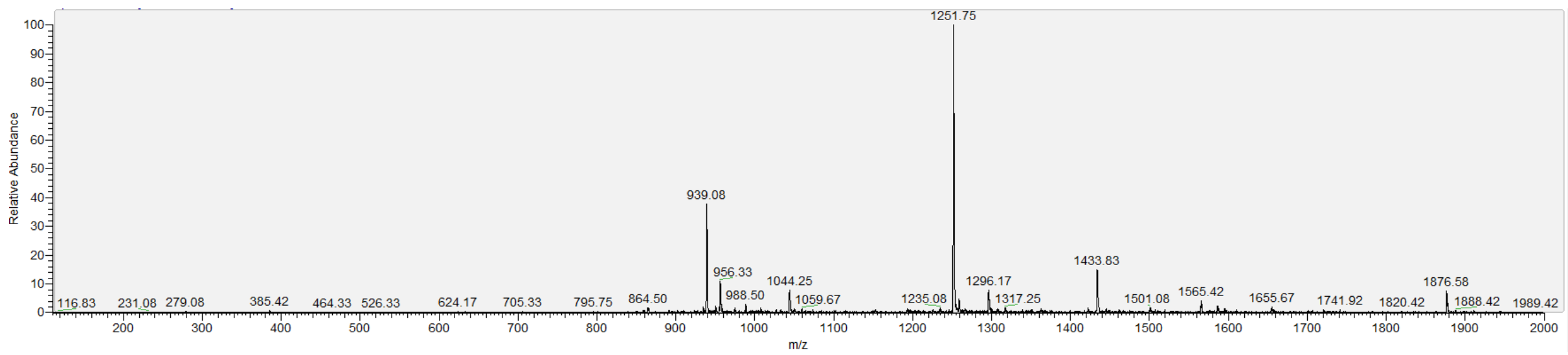

<u>**Ultravariegin**</u> **(For benchmarking in assay)**

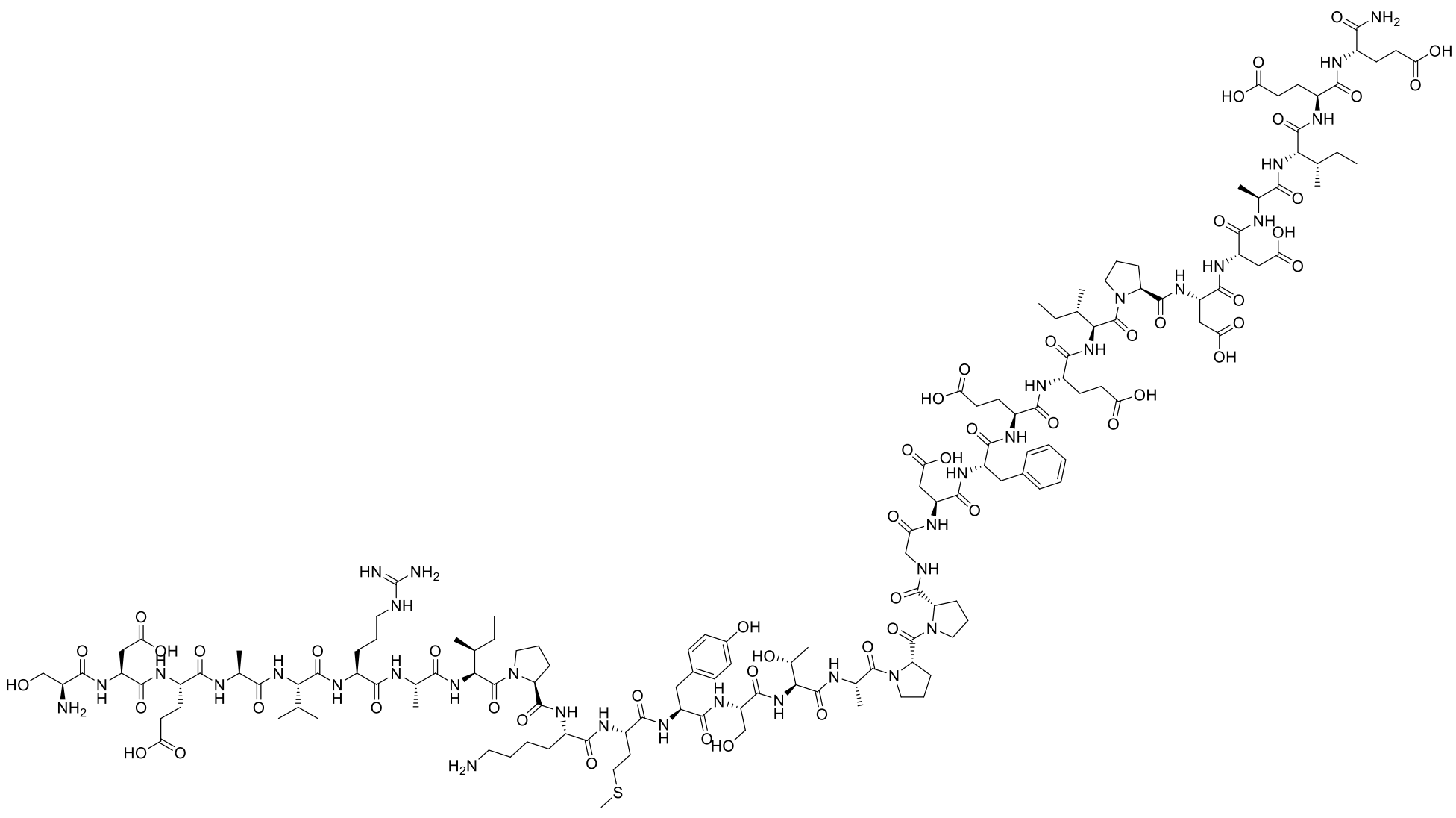

Chemical Formula: $C_{143}H_{219}N_{35}O_{52}S$, **Exact Mass**: 3290.53, **Molecular Weight**: 3292.58.

MALDI-TOF; m/z found: 3291.85.

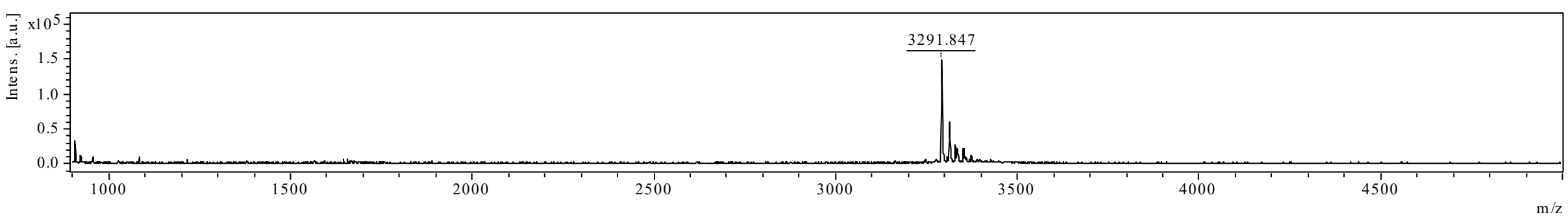

LCMS (ESI); RT= 1.64 min, $[M+2H]^{2+}$: 1646.92, $[M+3H]^{3+}$: 1098.33.

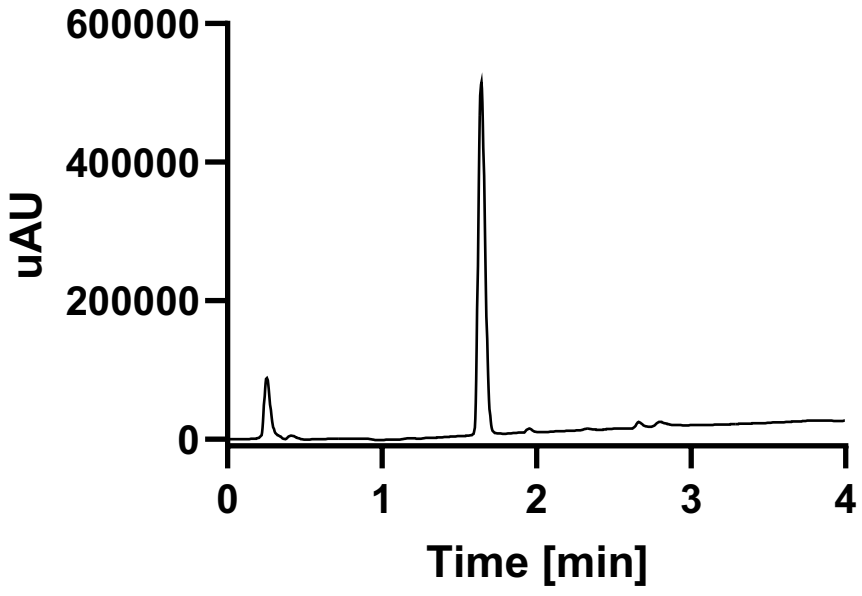

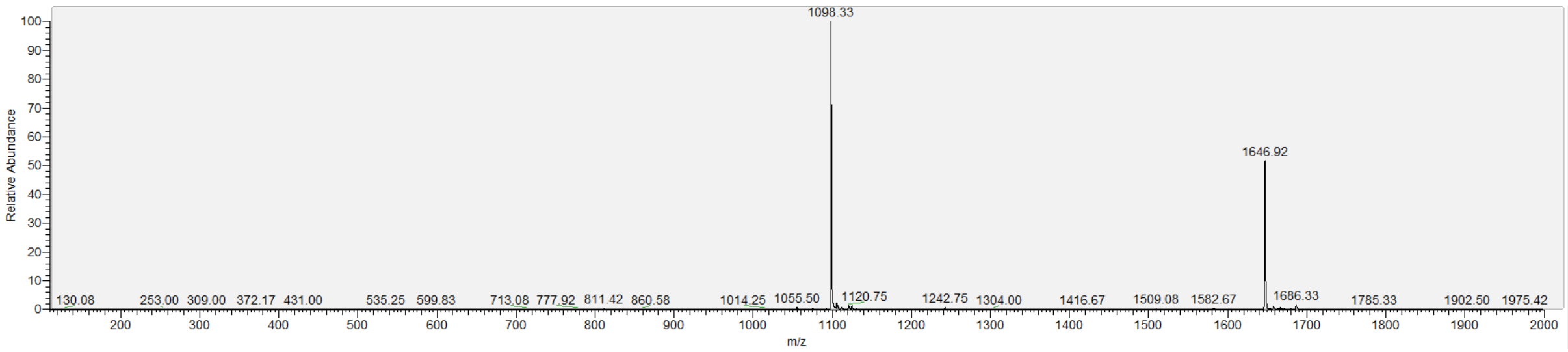

<u>Ultravariegin-Biotin</u> **(For benchmarking on SPR)**

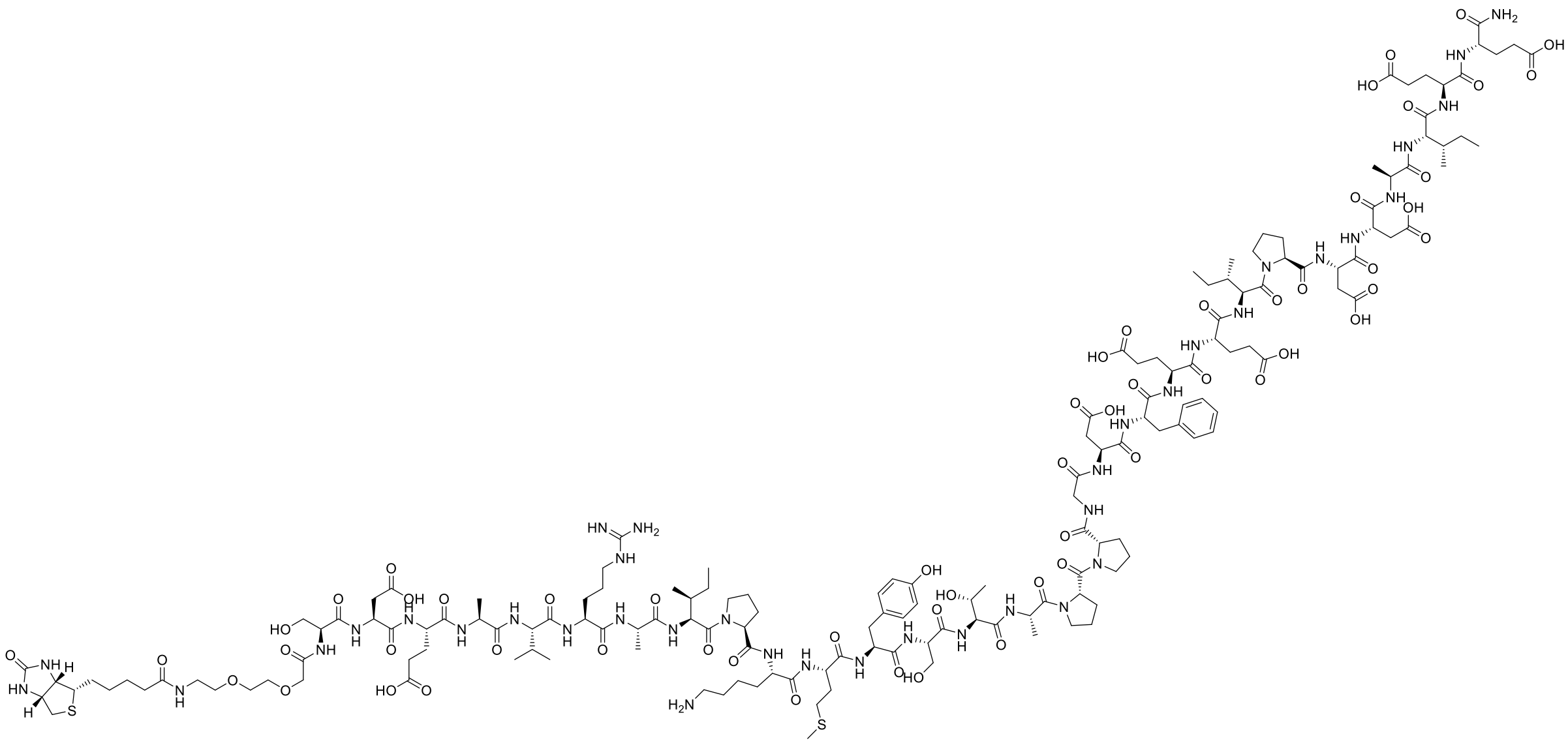

Chemical Formula: $C_{159}H_{244}N_{38}O_{57}S_2$, **Exact Mass**: 3661.68, **Molecular Weight**: 3664.03.

MALDI-TOF; m/z found: 3664.17.

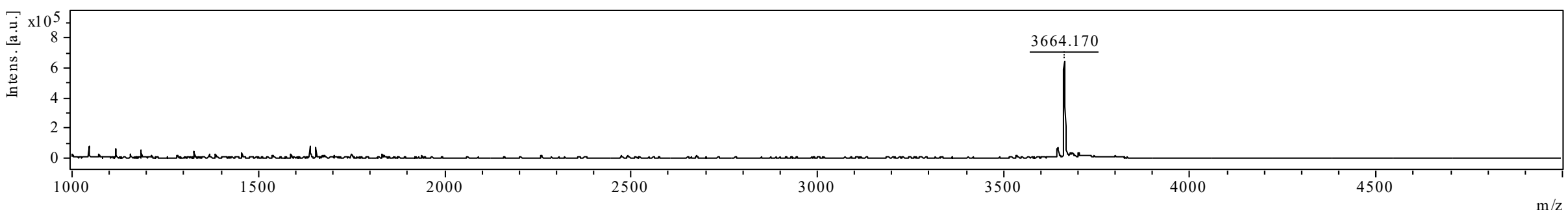

LCMS (ESI); RT= 1.73 min, $[M+2H]^{2+}$: 1832.67, $[M+3H]^{3+}$: 1222.08.

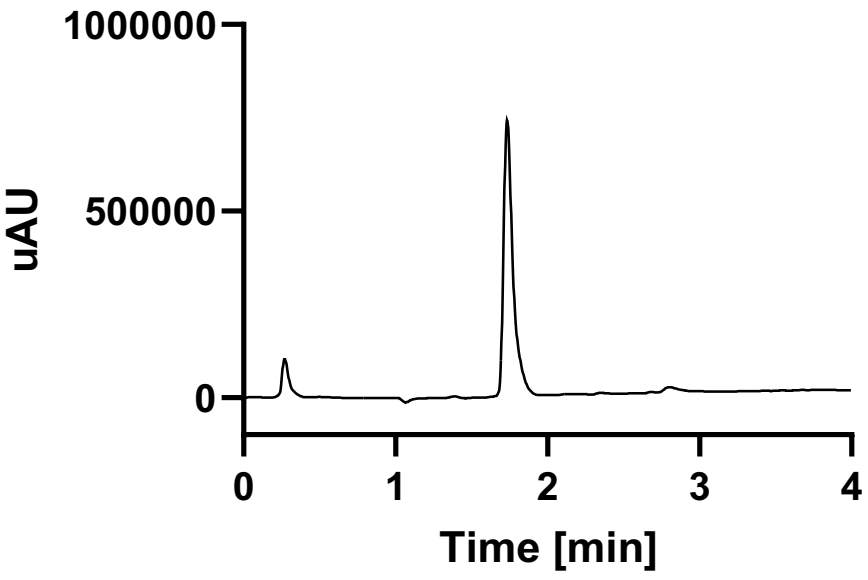

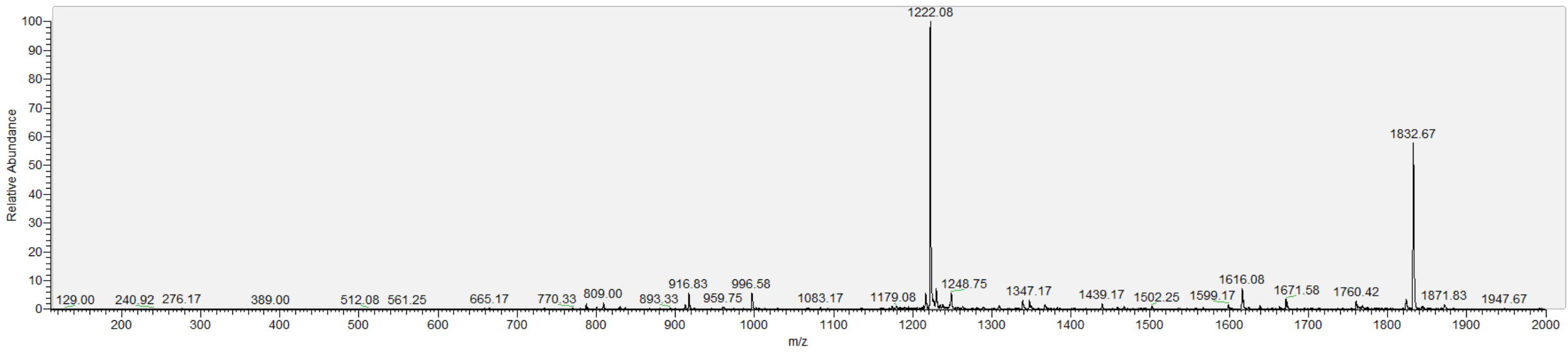

Bivalirudin (For benchmarking in assay)

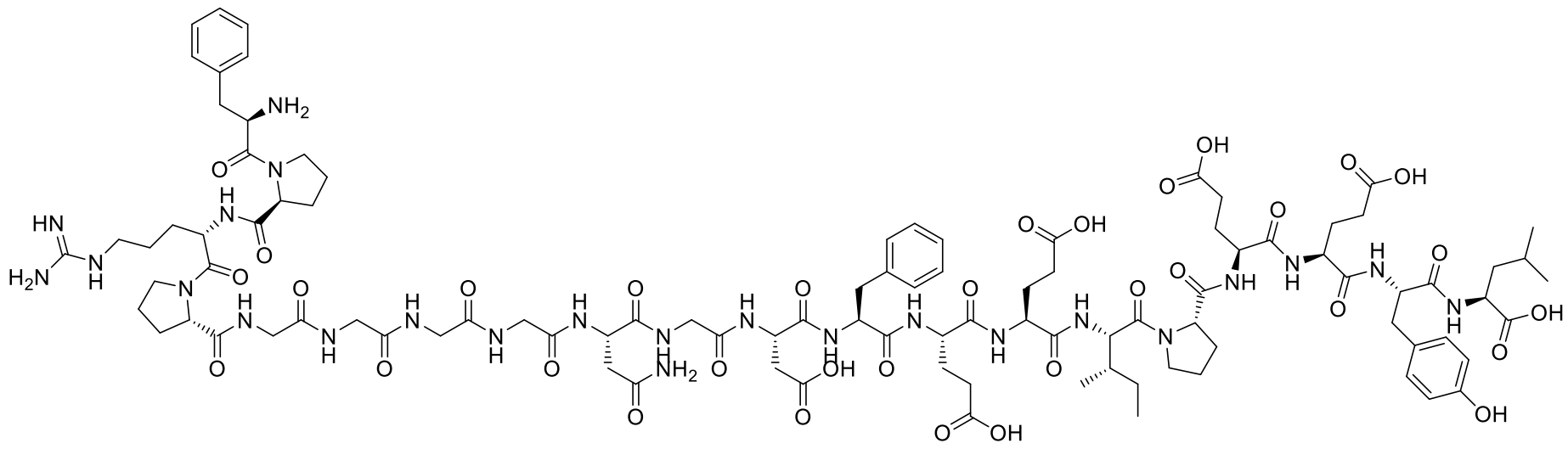

Chemical Formula: $C_{98}H_{138}N_{24}O_{33}$, **Exact Mass**: 2178.99, **Molecular Weight**: 2180.32.

MALDI-TOF; m/z found: 2179.70.

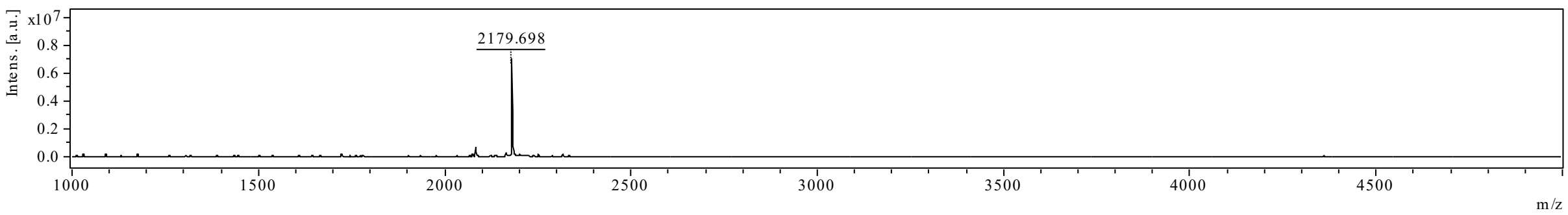

LCMS (ESI); RT= 1.84 min, $[M+2H]^{2+}$: 1090.83.

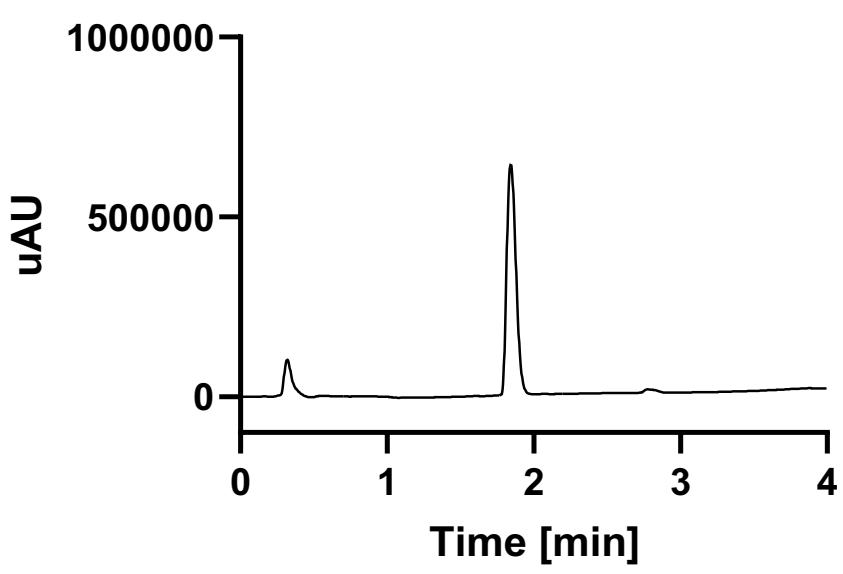

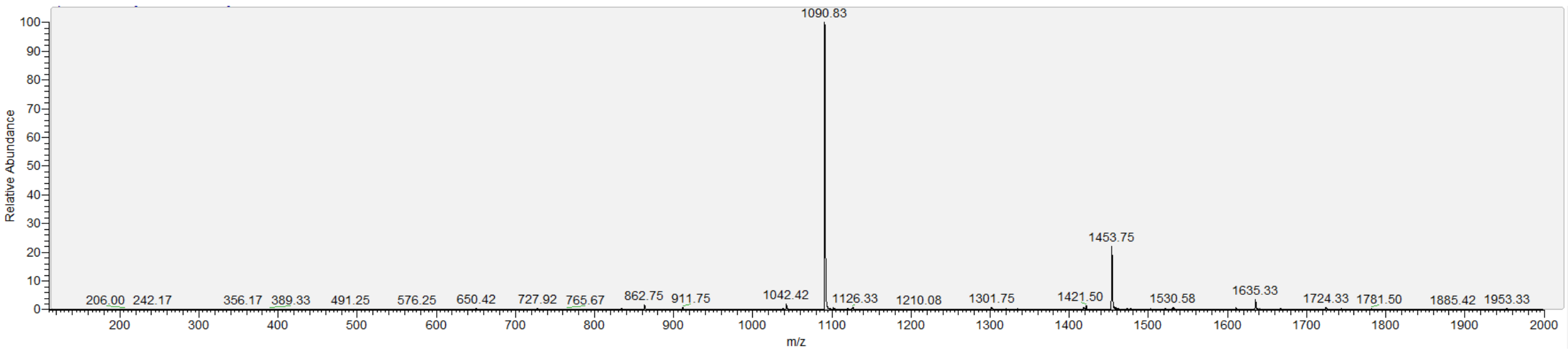

PNA-Biotin(AS sequence) (For SPR – immobilisation of Ex2-1-8mer)

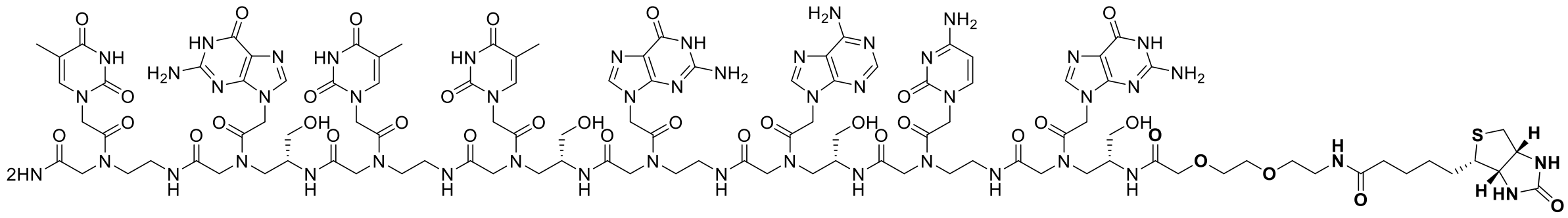

Chemical Formula: $C_{107}H_{143}N_{49}O_{35}S$, **Exact Mass**: 2707.69, **Molecular Weight**: 2707.69

MALDI-TOF; m/z found: 2708.25.

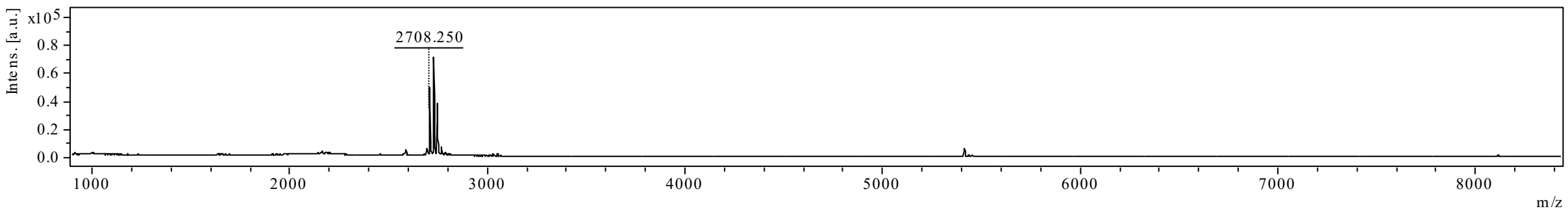

LCMS (ESI); RT= 1.26 min, $[M+2H]^{2+}$: 1354.58, $[M+3H]^{3+}$:903.58.

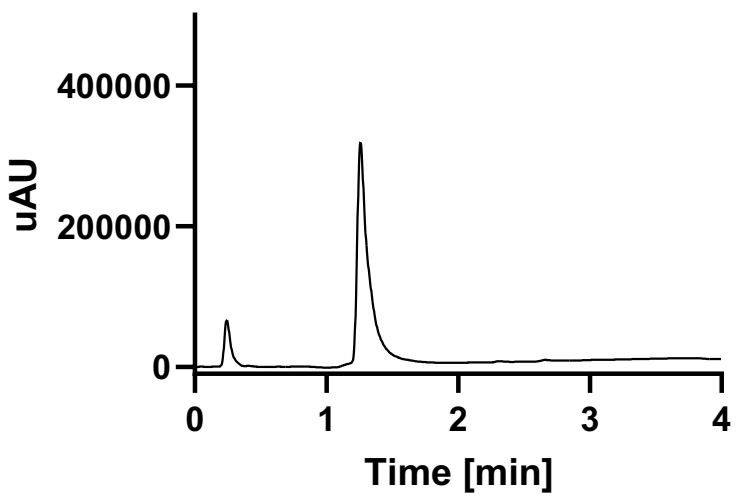

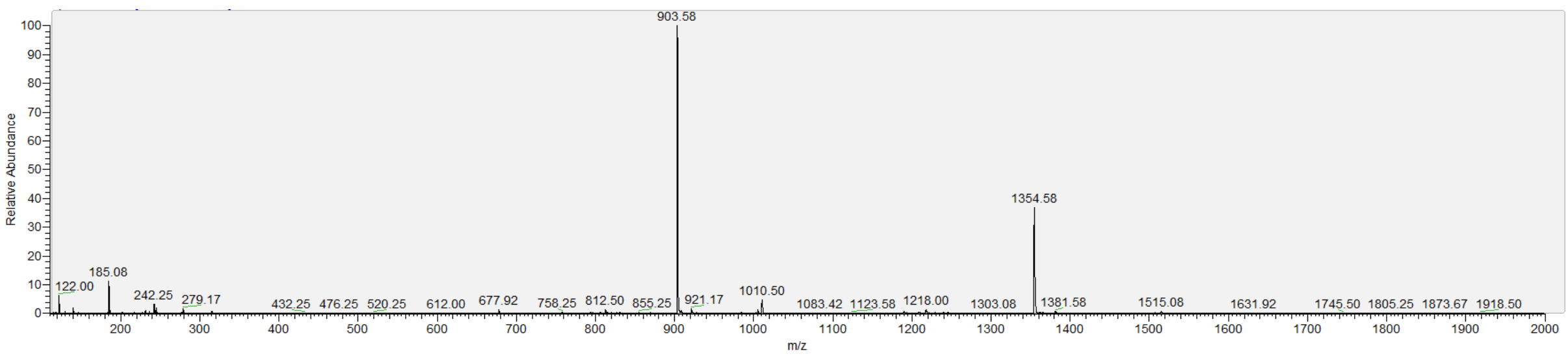

Ex2-1-8mer (For SPR)

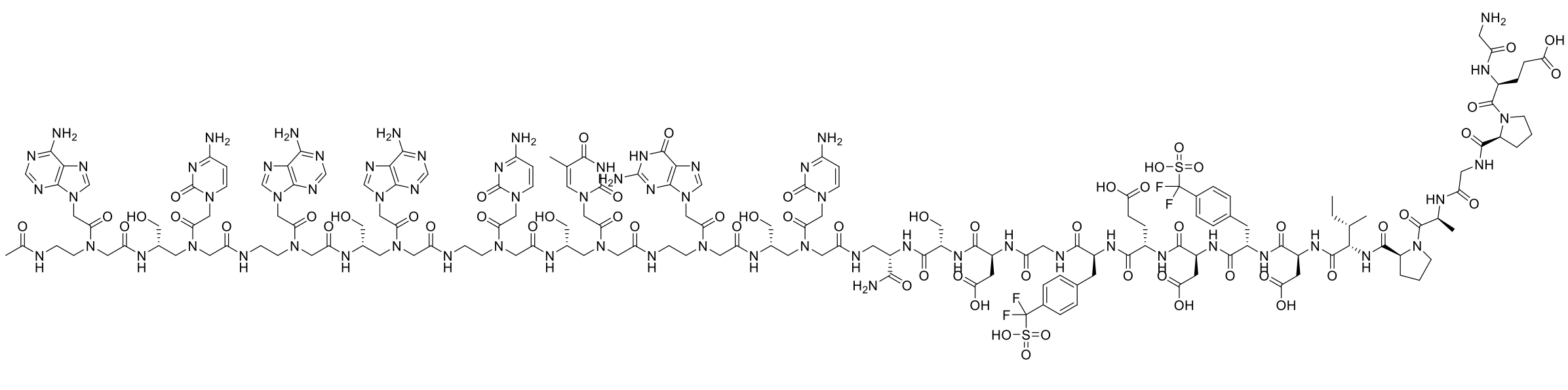

Chemical Formula: $C_{164}H_{215}F_4N_{65}O_{60}S_2$, **Exact Mass**: 4194.52, **Molecular Weight**: 4197.03.

MALDI-TOF; m/z found: 4197.52.

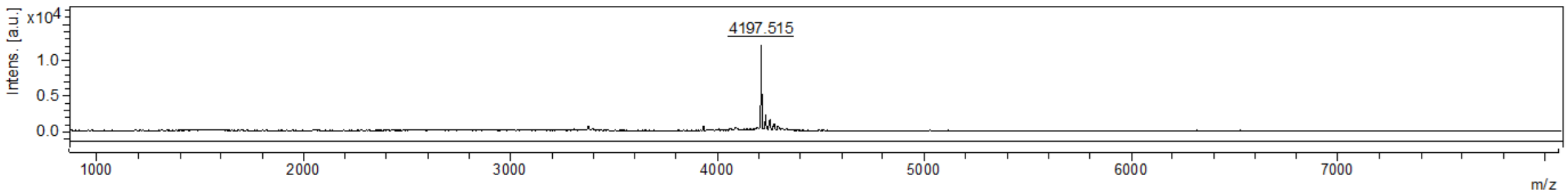

LCMS (ESI); RT= 1.38 min, $[M+3H]^{3+}$: 1399.75, $[M+4H]^{4+}$: 1050.25.

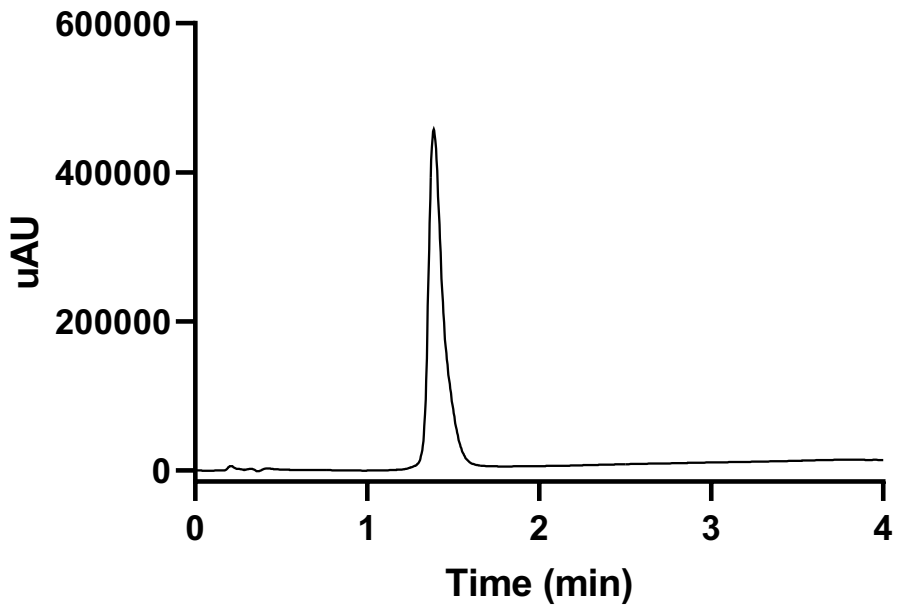

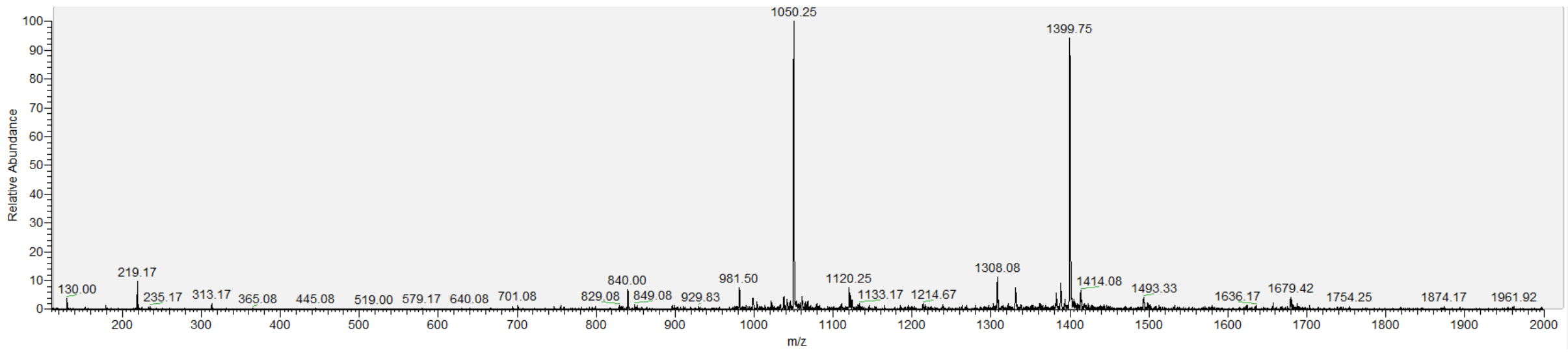

<u>**PNA-Biotin (Exosite Sequence)**</u> **(For SPR – immobilisation of AS31-8mer1)**

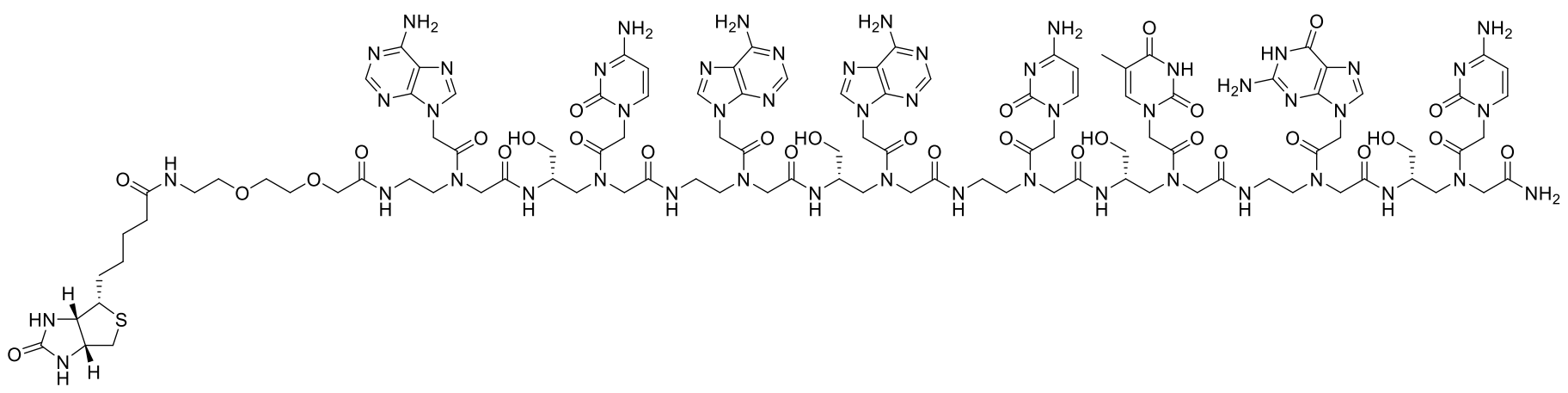

Chemical Formula: $C_{105}H_{141}N_{51}O_{31}S$, **Exact Mass**: 2644.08, **Molecular Weight**: 2645.67.

MALDI-TOF; m/z found: 2645.35.

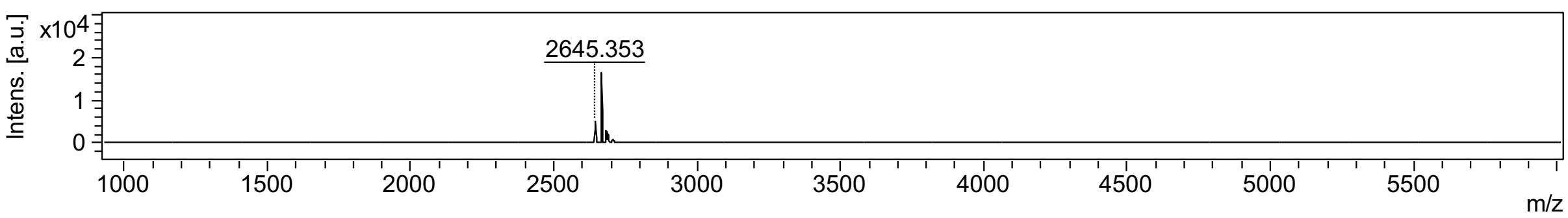

LCMS (ESI); RT= 1.26 min, $[M+2H]^{2+}$: 1323.25, $[M+3H]^{3+}$: 882.67, $[M+4H]^{4+}$: 662.25.

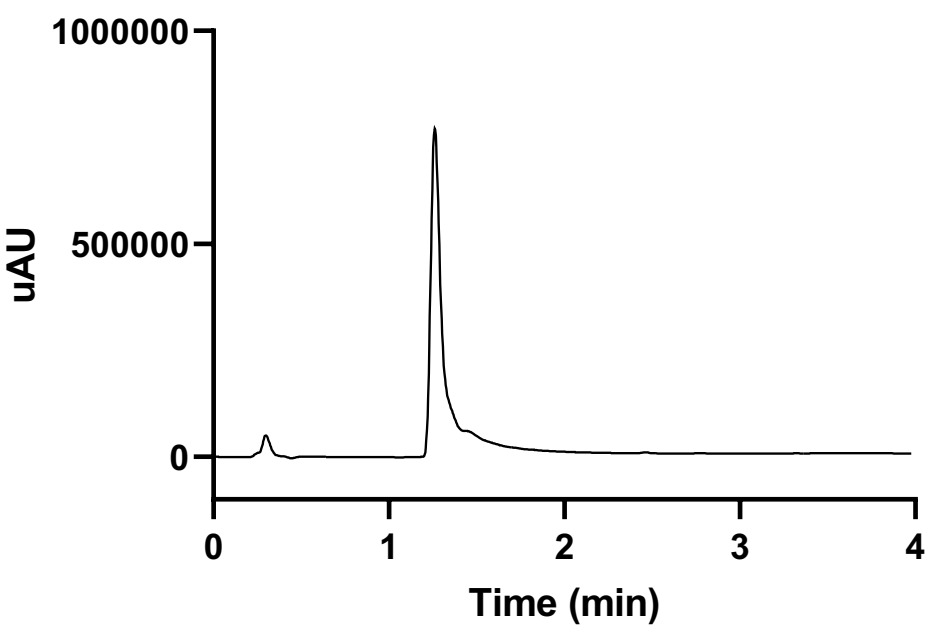

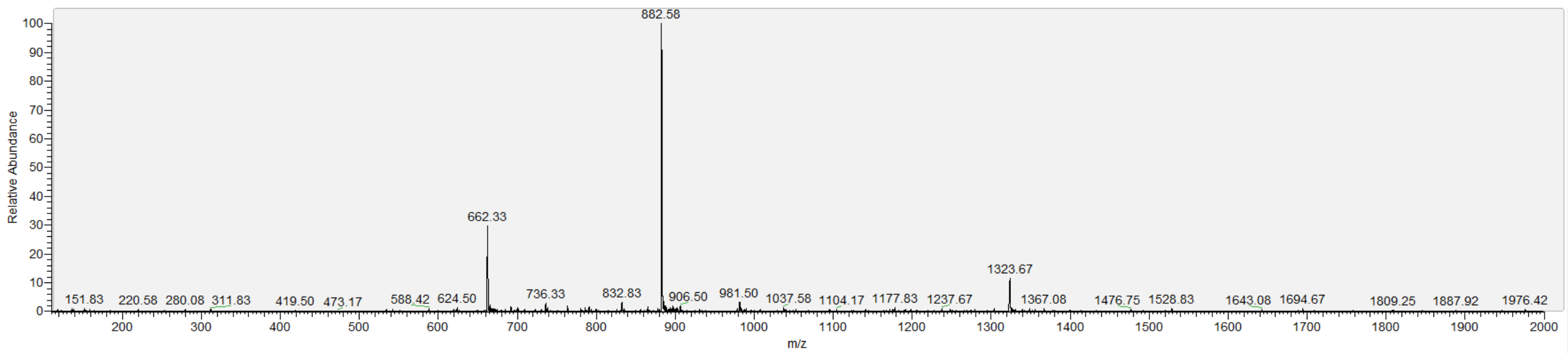

<u>AS31-8mer1</u> (For SPR)

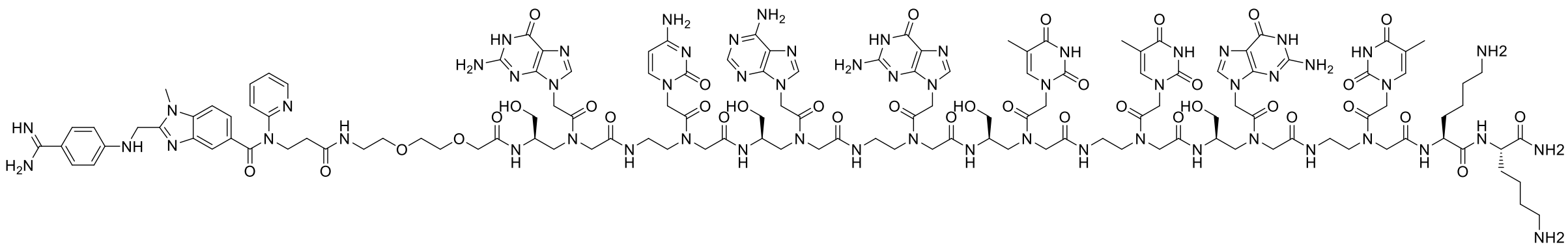

Chemical Formula: $C_{134}H_{176}N_{58}O_{37}$, **Exact Mass**: 3189.67, **Molecular Weight**: 3191.25.

MALDI-TOF; m/z found: 3191.58.

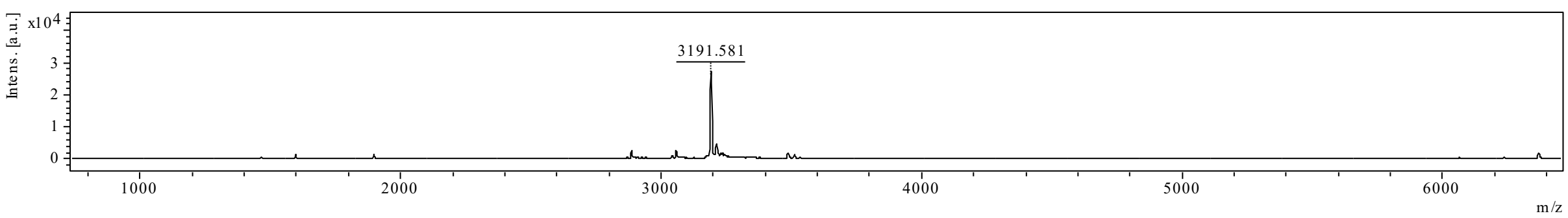

LCMS (ESI); RT= 1.23 min, $[M+3H]^{3+}$: 1064.75, $[M+4H]^{4+}$:798.92.

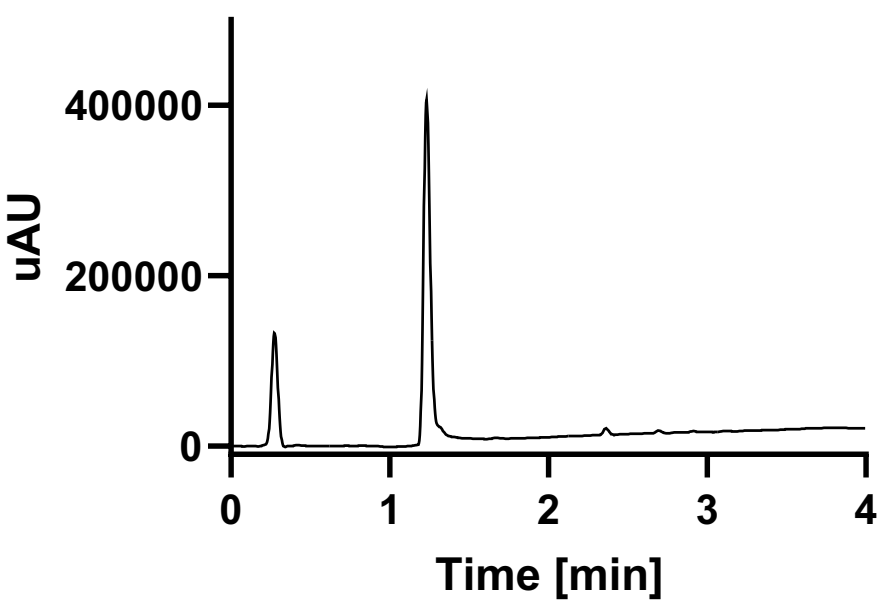

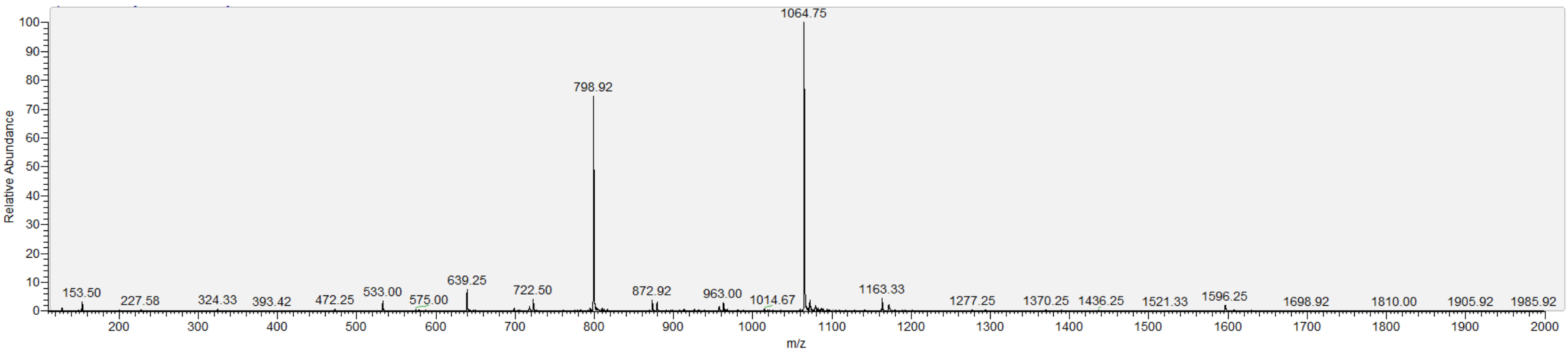

14. References

(1) M. Dockerill, D. J. Ford, S. Angerani, I. Alwis, L. J. Dowman, J. Ripoll-Rozada, R.E. Smythe, J. S. T. Liu, P. J. B. Pereira, S. P. Jackson, R. J. Payne, N. Winssinger, Development of supramolecular anticoagulants with on-demand reversibility. *Nature Biotechnology,* 43, 186–193 (2025). https://doi.org/10.1038/s41587-024-02209-z

(2) L. Dowman, L. J., S. M. Agten, J. Ripoll-Rozada, B. M. Calisto, P. J. B. Pereira, R. J. Payne, Synthesis and evaluation of peptidic thrombin inhibitors bearing acid-stable sulfotyrosine analogues. *Chem Commun* 57(83), 10923-10926 (2021). https://doi.org/10.1039/D1CC04742F
